# The Reactivity of Diiron(I) Bis-cyclopentadienyl Tricarbonyl Aminocarbyne Complexes in Aqueous Media: A Case Study for Iron-Based Anticancer Agents

**DOI:** 10.1021/acs.inorgchem.6c01829

**Published:** 2026-06-11

**Authors:** Eleonora Dolcher, Federico Simonelli, Fatima Nigro, Stefano Zacchini, Beatrice Campanella, Gianluca Ciancaleoni, Fabio Marchetti, Lorenzo Biancalana

**Affiliations:** † Department of Chemistry and Industrial Chemistry, 9310 University of Pisa, Via Giuseppe Moruzzi 13, 56124 Pisa, Italy; ‡ Department of Industrial Chemistry “Toso Montanari”, 9296 University of Bologna, Via Piero Gobetti 85, 40129 Bologna, Italy; § Istituto di Chimica dei Composti Organometallici, 9327 Consiglio Nazionale delle Ricerche, Via G. Moruzzi 1, 56124 Pisa, Italy

## Abstract

The biological effects of diiron­(I) aminocarbyne complexes [Fe_2_Cp_2_(CO)­(L)­(μ-CO)­{μ-CNR­(R’)}]^+^ originate from their intracellular disassembly, releasing reactive iron species. Herein, a systematic multitechnique investigation of the reactivity of tricarbonyl (L = CO) complexes in aqueous media was carried out. Well-soluble nitrate salts were prepared on a (multi)­gram scale and characterized by IR, NMR, and XRD. The degradation process in water or DMEM was assessed after 72 h at 37 °C over a wide concentration range via ^1^H NMR and UV–vis. Results were integrated by pH, conductivity, UV–vis and ^1^H NMR measurements at 24 h intervals and ICP-OES. The process follows zero-order kinetics above mM concentration, with partial formation of the corresponding secondary amine (RR′NH) and cyclopentadiene. The slowly forming brown precipitates contain iron­(III)-oxy­(hydroxides) and minor organic/organometallic components as shown by CHNS analyses, IR, Raman and ESI-MS. Evaluating the effects of O_2_, ambient light, temperature, pH, Me_3_NO on the process via ^1^H NMR and UV–vis provided key mechanistic insights. Addition of 1,3,5-triaza-7-phosphadamantane (PTA) enabled trapping of the CO-substituted intermediate [Fe_2_Cp_2_(CO)­(PTA)­(μ-CO)­{μ-CNR­(R’)}]^+^. The pathway leading to total disruption of the coordination sphere was elucidated by DFT. Overall, these results lay the foundations for understanding the behavior of this promising class of anticancer metallodrugs in physiological settings.

## Graphical abstract

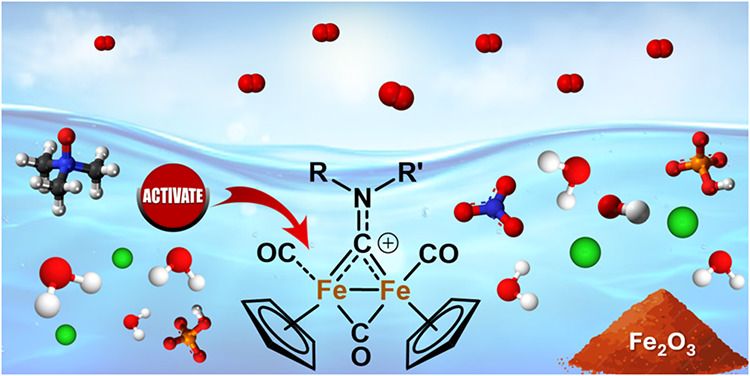

## 1 Introduction

A considerable diversity of transition metal complexes has been extensively investigated as anticancer agents, often aimed to outperform widely used platinum-based chemotherapeutics.^1^ Their behavior in physiological conditions is a central issue, encompassing solubility, lipophilicity and reactivity. Unknown/uncontrolled speciation underlies the toxic side effects of cisplatin^2^ and contributed to the clinical failure of titanocene dichloride.^3^ Before examining metallodrug candidates in complex environments, such as cellular media and biological fluids,^4–8^ it is fundamental to understand their reactivity in aqueous solution^9–15^ and in culture media.^16,17^ Several components of the latter may influence the speciation of metal complexes, including pH buffering agents, coordinating species such as chloride (*ca.*, 0.12 mol/L), α-amino acids (*ca.*, 10 mM in total), nicotinamide, biotin and reducing agents such as glucose and glutathione.^18,19^ Despite the fundamental importance, the behavior of metal compounds in aqueous and culture media is often overlooked or not studied in detail, including different techniques and across multiple concentration ranges.

Bis-cyclopentadienyl tricarbonyl diiron­(I) aminocarbyne complexes of general formula [Fe_2_Cp_2_(CO)_2_(μ-CO)­(μ-CNRR’)]^+^, [**1**]^+^ (Figure 1; R, R′ = alkyl or aryl group) are easily accessible from commercial [Fe_2_Cp_2_(CO)_4_], isocyanides and alkylating agents.^20^ After pioneering organometallic investigations 30–40 years ago,^21^ they returned to the spotlight as an emerging class of anticancer compounds,^22–25^ together with their carbonyl-substituted derivatives, [Fe_2_Cp_2_(CO)­(L)­(μ-CO)­(μ-CNRR’)]^+^, [**2**]^+^ (Figure 1; L = neutral monodentate ligand ≠ CO).^26–31^ Both [**1**]^+^ and [**2**]^+^ display favorable prerequisites for drug development such as straightforward and multigram-scalable synthesis, huge structural variability given by the option of substituents and ligands (R, R′, L) and the associated counteranion (usually CF_3_SO_3_^–^), and a range of physicochemical properties suitable for biological applications. These comprise water solubility in the low mM range, a balanced lipophilic-hydrophilic character (as per octanol–water partition coefficient; −1 < Log *P*_ow_ < +1) and a considerable inertness in aqueous solution. The most performing tricarbonyl compound, **FEAMP** (Figure 1), displayed cytotoxicity in six different human cancer cell lines, with an average 6-fold selectivity with respect to normal cells which is not correlated with the intracellular iron uptake. Moreover, **FEAMP** outperformed cisplatin in more challenging 3D models of ovarian (2008) and pancreatic (PSN-1) cancer cells.^23^

**Figure 1:**
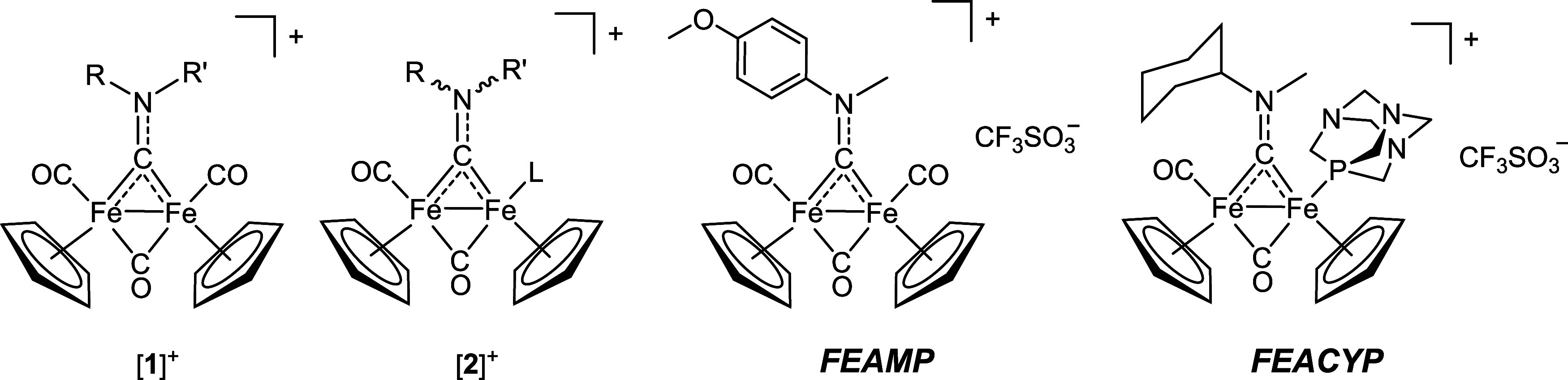
General structure of tricarbonyl [**1**]^+^ and dicarbonyl [**2**]^+^ diiron­(I) bis-cyclopentadienyl aminocarbyne complexes (R, R′ = alkyl, aryl group; L = monodentate ligand ≠ CO; wavy bonds represent *E*/*Z* isomerism) and lead compounds with prominent anticancer activity *in vitro* (**FEAMP**) and/or *in vivo* (**FEACYP**). In this manuscript **FEAMP** = [**1f**]­CF_3_SO_3_ and **FEACYP** = [**2b**]­CF_3_SO_3_.

Replacement of a terminal carbonyl ligand with isocyanides,^26,27^ amines,^28^ pyridines^29^ or phosphanes^30,31^ modulates the anticancer activity. Among the dicarbonyl derivatives [**2**]^+^, **FEACYP** (Figure 1) exhibited potent cytotoxicity against a panel of human cancer cell lines, with IC_50_ values in the low micromolar range, including 3D spheroid models, coupled with a 6-fold selectivity with respect to noncancerous cells. Moreover, **FEACYP** showed excellent suppression of Lewis lung carcinoma in a murine model associated with lower adverse effects compared to cisplatin.

The anticancer activity of the tricarbonyl complexes [**1**]^+^ has been associated with elevation of reactive oxygen species (ROS) levels, disruption of mitochondrial membrane potential, inhibition of Thioredoxin Reductase (TxrR), and release of carbon monoxide.^22,23^ However, these complexes do not offer straightforward reactivity pathways in aqueous solution such as ligand substitutions, reductions/oxidations or nucleophilic additions,^20^ at variance to the most common metallodrugs.^10^

Indeed, very weak interactions were observed with a DNA model^22^ and some proteins, except for a case of protein methylation.^23^ Hence the biological effects do not arise from the complexes themselves, acting as pro-drugs. It has been hypothesized that the compounds undergo a rapid disruption intracellularly that liberates reactive iron species.^22–25^ Such process was conceived based on the irreversible transformation that typically occurs in aqueous solutions of [**1**]^+^ exposed to air, consisting in gradual increase of turbidity, ultimately leading to orange-brown precipitates. This process is relatively slow at a millimolar concentration level so that a substantial fraction^32^ (*e.g.*, 70–80%) of the starting organometallic cation is found in solution after 72 h at 37 °C (^1^H NMR and IR data), highlighting the robustness of the diiron scaffold.

Investigating such a slow and low-yielding process in dilute aqueous solutions is not an easy task. Some scattered data concerning the products of this reaction are compiled in Table S1. In selected cases, the precipitate was collected and iron­(III) (hydr)­oxides were identified by Raman analyses. Their formation points to a role of dioxygen, at least as ultimate oxidizing agent, and implies complete loss of ligands from the starting organodiiron cation. Consistently, release of carbon monoxide was ascertained by GC-TCD (Thermal conductivity detector) analyses and myoglobin assay while the fate of the other ligands remains unclear. Possible formation of the secondary amine deriving from the cleavage of the carbyne-nitrogen bond has been reported in a few cases. Other soluble organo-iron complexes besides the starting material have never been detected. Based on the currently available evidence, the process underlying the total disruption of the coordination sphere is summarized in eq 1.

1 $${\left[{\mathrm{Fe}}_{2}{\mathrm{Cp}}_{2}{\left(\mathrm{CO}\right)}_{3}\left({\mathrm{CNR}}_{2}\right)\right]}_{\left(\mathrm{aq}\right)}^{+}+O_{2\left(g/\mathrm{aq}\right)}\left(?\right)+H_{2}O_{\left(l\right)}\to {\mathrm{FeO}}_{x}\cdot nH_{2}O_{\left(s\right)}+{\mathrm{CO}}_{\left(g\right)}+\mathrm{CpH}{\left(?\right)}_{\left(g/\mathrm{aq}\right)}+R_{2}\mathrm{NH}{\left(?\right)}_{\left(\mathrm{aq}\right)}$$

A slow decrease of [**1**]^+^ was also ascertained in RPMI-1640 or DMEM, accompanied by the formation of an orange-brown precipitate, without soluble FeCp products detectable by ^1^H NMR. However, further data are needed to establish to what extent the process in cell culture medium is similar to that occurring in water. For instance, the precipitates obtained in these conditions were not analyzed.

The behavior of dicarbonyl derivatives [**2**]^+^ in aqueous solution and cell culture medium shows parallels with that of the tricarbonyl derivatives [**1**]^+^ (Table S1).^26–31^ In this case, the inertness of the starting material heavily depends on the nature of the monodentate noncarbonyl ligand L. Compared to [**1**]^+^, the scenario is complicated by the *E*/*Z* isomerism across the μ-C=N bond and consequent steric/electronic effects with the substituents on the aminocarbyne ligand.

In summary, a systematic study is needed to elucitate the kinetics, the mechanism and the products of degradation processes occurring in water and cell culture media of diiron­(I) aminocarbyne complexes.

In this work, we present a comprehensive investigation of the aqueous reactivity of a selection of tricarbonyl complexes [**1**]^+^, combining complementary techniques across a wide concentration range and different conditions, including temperature, pH, oxygen, ambient light and the cell culture medium. The results lay the groundwork to rationalize the mechanism of action of this class of anticancer compounds and the related CO-substituted derivatives [**2**]^+^.

## 2 Results and Discussion

### 2.1 Synthesis and Characterization of Nitrate Salts

The aqueous solubility of tricarbonyl aminocarbyne compounds [**1**]­CF_3_SO_3_ is limited to the low mM range, especially with aromatic groups in the *N*-substituents (*e.g.*, benzyl, xylyl). A higher solubility would allow comparison of different complexes over a wider concentration range, while avoiding the use of organic cosolvents. As the biological activity is associated with the organometallic cation, a strategy to increase its concentration in aqueous solution is the association of an appropriate counteranion, such as nitrate.^24,33–35^

Therefore, the nitrate salts of the target aminocarbyne complexes were prepared. Compound [Fe_2_Cp_2_(CO)_2_(μ-CO)­(μ-CNMe_2_)]­NO_3_, [**1a**]­NO_3_, was previously obtained by a three-step procedure involving methyl isocyanide/carbonyl exchange on [Fe_2_Cp_2_(CO)_4_] followed by isocyanide *N*-alkylation with methyl iodide and ion metathesis with silver nitrate.^24^ Herein, a new one-pot procedure was developed for the gram and multigram scale preparation of the unprecedented nitrate salts of [Fe_2_Cp_2_(CO)_2_(μ-CO)­{μ-CNR­(R′)}]^+^ (R′ = Me, R = Cy, [**1b**]^+^, R = Bn, [**1c**]^+^, R = Xyl, [**1e**]^+^; R′ = R = Bn, [**1d**]^+^) in MeCN as solvent (Scheme 1). The time/temperature conditions for the isocyanide/carbonyl exchange on [Fe_2_Cp_2_(CO)_4_] in the first step were considerably reduced with respect to the literature.^22,23,36^ The subsequent reaction with methyl iodide or benzyl bromide required forced conditions in terms of molar amount, temperature and reaction time, reflecting the lower alkylating ability as compared to methyl triflate (1 equiv, CH_2_Cl_2_, room T).^21,37^ However, further excess of MeI decreased the yield of the desired product and promoted the formation of piano-stool Fe­(II) compounds. In the third step, a slight excess of AgNO_3_ was necessary to achieve a quantitative anion exchange. Compounds [**1b**-**e**]­NO_3_ were purified by alumina chromatography and isolated as air-stable bright red solids in 63–80% yield.

**Scheme 1:**
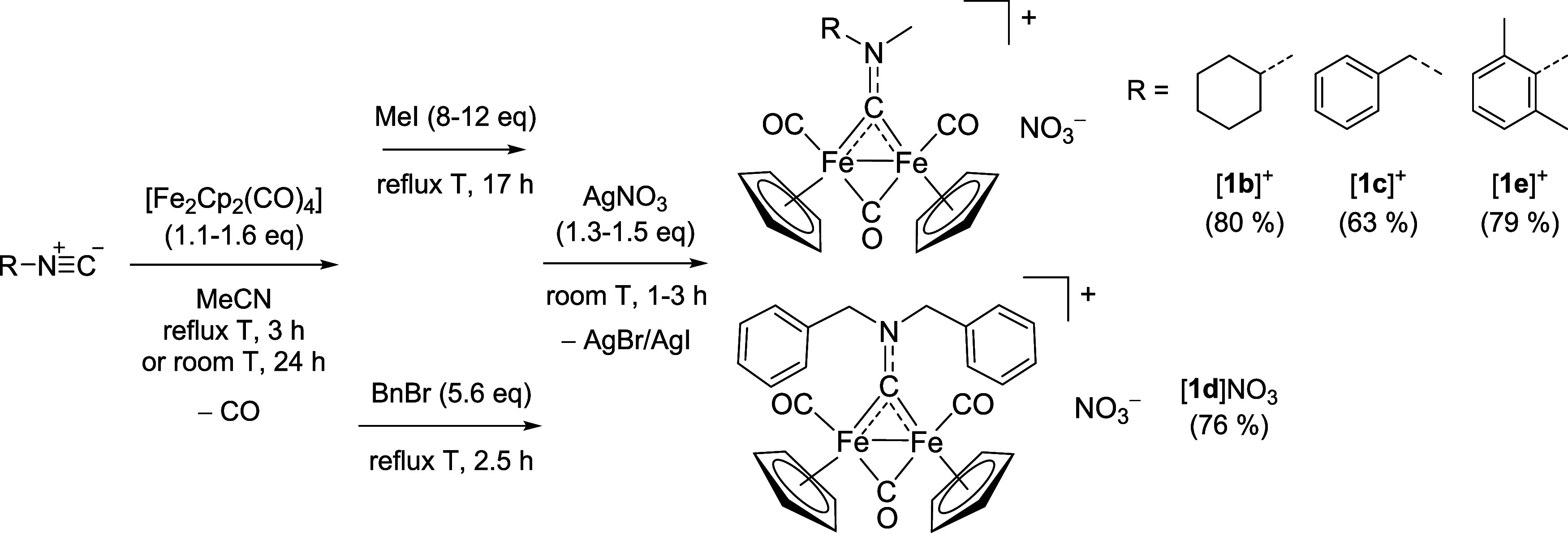
One-Pot, Three-Step (Multi)­gram-Scale Preparation of [Fe_2_Cp_2_(CO)_2_(μ-CO)­{μ-CNR­(R′)}]­NO_3_ from [Fe_2_Cp_2_(CO)_4_], the Selected Isocyanide (CNR), Alkyl Halide and Silver Nitrate in MeCN^a^

a Isolated yields in parentheses.

Compounds [**1b**-**e**]­NO_3_ were characterized by CHNS analyses, IR (solid state and CH_2_Cl_2_) and NMR spectroscopy (CDCl_3_, CD_3_CN or acetone-d_6_); IR and NMR spectra are shown in Figures S1–S16. IR absorptions, ^1^H and ^13^C NMR signals arising from the organodiiron cations are in agreement with those of the previously reported triflate salts.^22^ In addition, a relatively broad IR absorption around 1350 cm^–1^ accounts for the nitrate anion. The water solubility of [**1a**-**e**]­NO_3_ was determined by ^1^H NMR on saturated D_2_O solutions at ambient temperature (*ca.*, 22 °C) using Me_2_SO_2_ as internal standard (Table S2). Solubility values increase in the series [**1d**]^+^ (≈4 mM) < [**1e**]^+^ ≤ [**1c**]^+^ (≈15–16 mM) < [**1b**]^+^ (≈40 mM) < [**1a**]^+^ (≈0.10 M), depending on the type of *N-*substituents on the aminocarbyne ligand. Compounds [**1a**-**e**]­NO_3_ are 5- to 16-fold more water-soluble than the corresponding triflates (Figure S17).^22^

The crystal structures of [**1a**]­NO_3_, [**1b**]­NO_3_ and [**1e**]­NO_3_ were ascertained by X-ray diffraction (Figures 2, 3, and 4). Comparison with [**1a**,**b**]­CF_3_SO_3_ ^22,23^ and [**1b**]­Cl^23^ shows that different anions do not appreciably affect the structural parameters of the organodiiron cations. Complexes [**1a**]^+^, [**1b**]^+^ and [**1e**]^+^ display a *cis*-arrangement of the Fe_2_Cp_2_(CO)_2_ core and the μ-CNMe­(R)^+^ ligand may be described as an almost perfect aminocarbyne-iminium hybrid structure.

**Figure 2:**
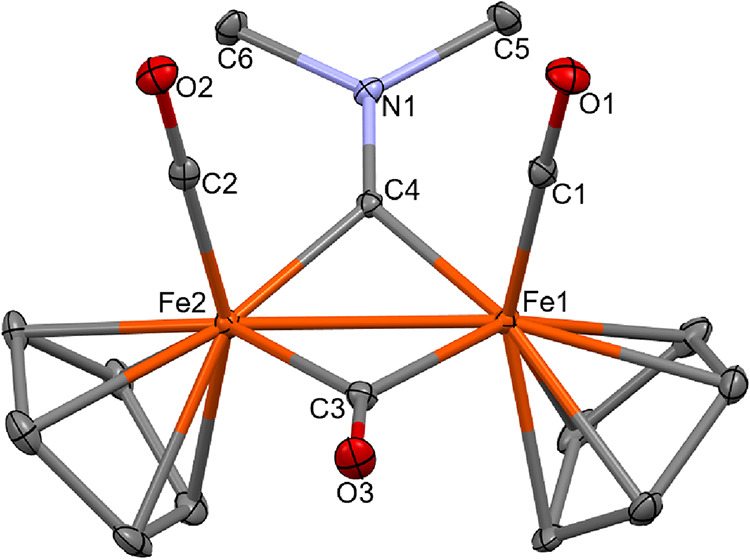
View of the structure of [**1a**]^+^ in [**1a**]­NO_3_. Displacement ellipsoids are at the 30% probability level. H-atoms have been omitted for clarity. Main bond distances (Å) and angles (°): Fe1–Fe2 2.5146(5), Fe1–C1 1.772(3), Fe2–C2 1.763(3), Fe1–C3 1.937(3), Fe2–C3 1.933(3), Fe1–C4 1.880(3), Fe2–C4 1.873(3), C1–O1 1.144(4), C2–O2 1.142(4), C3–O3 1.175(4), N1–C4 1.294(4), N1–C5 1.468(4), N1–C6 1.472(4), Fe1–C1–O1 179.0(3), Fe2–C2–O2 179.7(3), Fe1–C3–Fe2 81.05(11), Fe1–C4–Fe2 84.15(11), C4–N1–C5 123.4(2), C4–N1–C6 123.2(3), C5–N1–C6 113.3(2).

**Figure 3:**
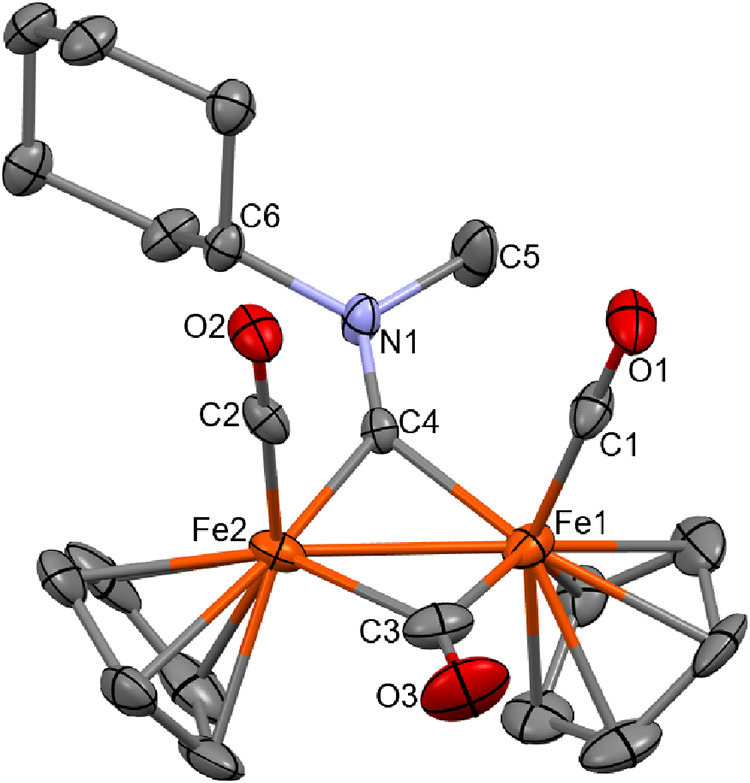
View of the structure of [**1b**]^+^ in [**1b**]­NO_3_·0.5CH_2_Cl_2_. Displacement ellipsoids are at the 30% probability level. H-atoms have been omitted for clarity. Main bond distances (Å) and angles (°): Fe1–Fe2 2.4979(12), Fe1–C1 1.772(6), Fe2–C2 1.758(6), Fe1–C3 1.918(7), Fe2–C3 1.938(7), Fe1–C4 1.878(5), Fe2–C4 1.878(5), C1–O1 1.133(7), C2–O2 1.138(6), C3–O3 1.171(7), N1–C4 1.280(6), N1–C5 1.484(7), N1–C6 1.492(6), Fe1–C1–O1 179.1(6), Fe2–C2–O2 178.4(5), Fe1–C3–Fe2 80.8(2), Fe1–C4–Fe2 83.35(19), C4–N1–C5 121.5(5), C4–N1–C6 122.7(4), C5–N1–C6 115.8(4).

**Figure 4:**
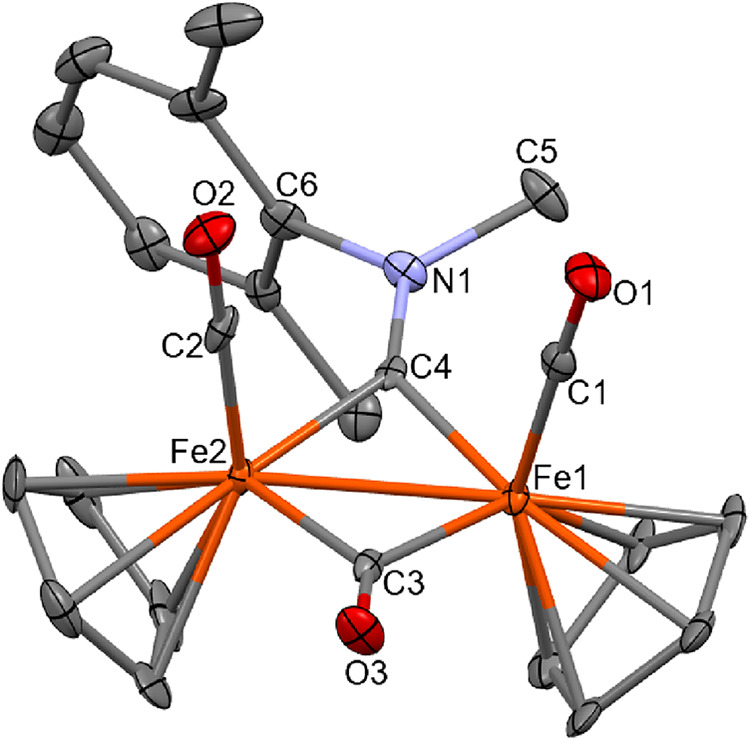
View of the structure of [**1e**]^+^ in [**1e**]­NO_3_. Displacement ellipsoids are at the 30% probability level. H-atoms have been omitted for clarity. Main bond distances (Å) and angles (°): Fe1–Fe2 2.5183(13), Fe1–C1 1.774(8), Fe2–C2 1.766(8), Fe1–C3 1.929(8), Fe2–C3 1.933(8), Fe1–C4 1.878(8), Fe2–C4 1.894(8), C1–O1 1.133(9), C2–O2 1.143(10), C3–O3 1.173(10), N1–C4 1.269(10), N1–C5 1.480(10), N1–C6 1.482(10), Fe1–C1–O1 178.5(7), Fe2–C2–O2 179.6(8), Fe1–C3–Fe2 81.4(3), Fe1–C4–Fe2 83.8(3), C4–N1–C5 124.2(7), C4–N1–C6 122.8(7), C5–N1–C6 113.0(6).

### 2.2 Overview of the Investigation of Reactivity in Aqueous Media

The activation process of diiron­(I) bis-cyclopentadienyl aminocarbyne complexes [**1**]^+^ in aqueous media involves a variety of compounds differing in physical state (gaseous, soluble in water, solid) and chemical nature (organic, inorganic, organometallic) (Introduction). The study of the kinetics/mechanism and the identification and quantitation of the products of this process is complicated by its slow rate, resulting in low conversion of the starting material even after 72 h. Based on a prior general knowledge of the system, different protocols were developed for the characterization and/or quantitation of each component using different techniques (Figure 5), with the aim of minimizing the consumption of materials/solvents. All procedures begin with the preparation of solutions of [**1**]^+^ in water or cell culture medium (DMEM) (using deuterated versions for NMR experiments) which were kept at 37 °C for 72 h (or other temperature/time) and then analyzed.

**Figure 5:**
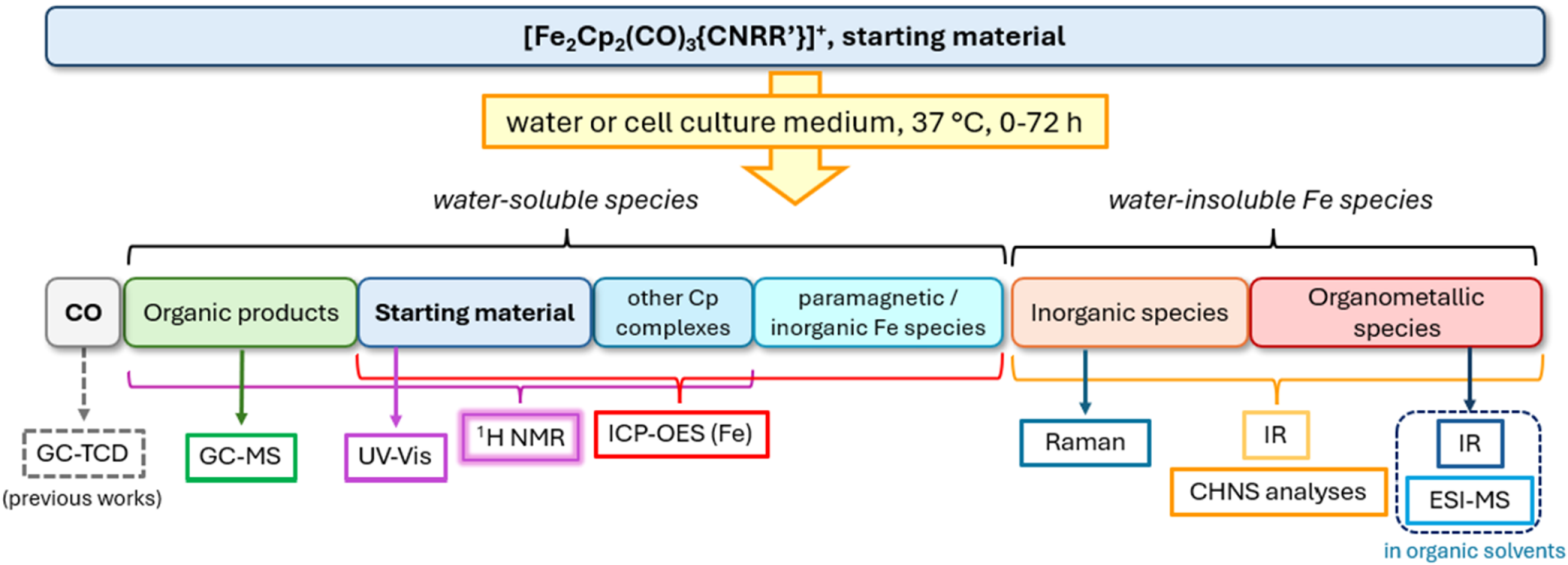
Techniques employed to analyze the speciation of diiron­(I) tris-carbonyl aminocarbyne complexes in aqueous media.

Bicarbonate was preferred to the commonly used phosphate buffer for regulating the pH of the cell culture medium because it is more relevant for cell cultures (bicarbonate/phosphate molar ratio ≈48 in DMEM formulations)^18^ and in biological systems.^38^ Except where otherwise noted (Section 2.6), all experiments were carried out in closed systems (NMR or test tubes) exposed to ambient light/air. The main technique used for assessing the consumption of the starting material and analyzing some of the products is ^1^H NMR, which was optimized in terms of selection of internal standards, acquisition parameters and spectral processing (Section 4 and Figures S18–S20 for details). This method has a threshold concentration of *ca* 1 mM, below which the final spectra suffer from an insufficient *S*/*N* ratio and/or a disproportionate intensity of the HDO/H_2_O signal. Hence, UV–vis was adopted for monitoring the process in the 1.0–0.1 mM range, the latter representing the lower limit of this technique considering the absorption of [**1**]^+^ at 340 nm (ε ≈ 5·10^3^ M^–1^·cm^–1^).

### 2.3 Extent and Kinetics of the Process in Water and Cell Culture Medium

First, the reactivity of the tricarbonyl complexes [**1a**-**e**]^+^ in aqueous solution was examined by ^1^H NMR and UV–vis. Most experiments were conducted with the nitrate salts, covering a wider concentration range (from *ca.* 1·10^–4^ to 1–6·10^–2^ mol/L) than the corresponding triflates, thanks to their higher water solubility. The % residual amount of the starting organometallic cation after 72 h at 37 °C, compared to the freshly prepared solution, is reported in Tables S3–S7; representative NMR and UV–vis spectra are shown in Figures S21–S26.

The initial concentration (*c*_Fe2_^0^) has a remarkable influence on the extent of decomposition of the diiron complex. For instance, a 22 mM solution of [**1a**]­NO_3_ undergoes negligible changes over 72 h at 37 °C (≈95% starting material) while the same compound is much less inert in a hundred-fold more dilute solution (≈58% starting material at *c*_Fe2_^0^ ≈ 0.15 mM). The % residual amount of [**1a**]^+^ shows an approximately exponential decrease when plotted against the decreasing initial concentration (increasing dilution, −*c*_Fe2_^0^), which corresponds to a linear trend on a logarithmic scale (*p*Fe^0^ = −Log *c*_Fe2_^0^; Figure 6).

**Figure 6:**
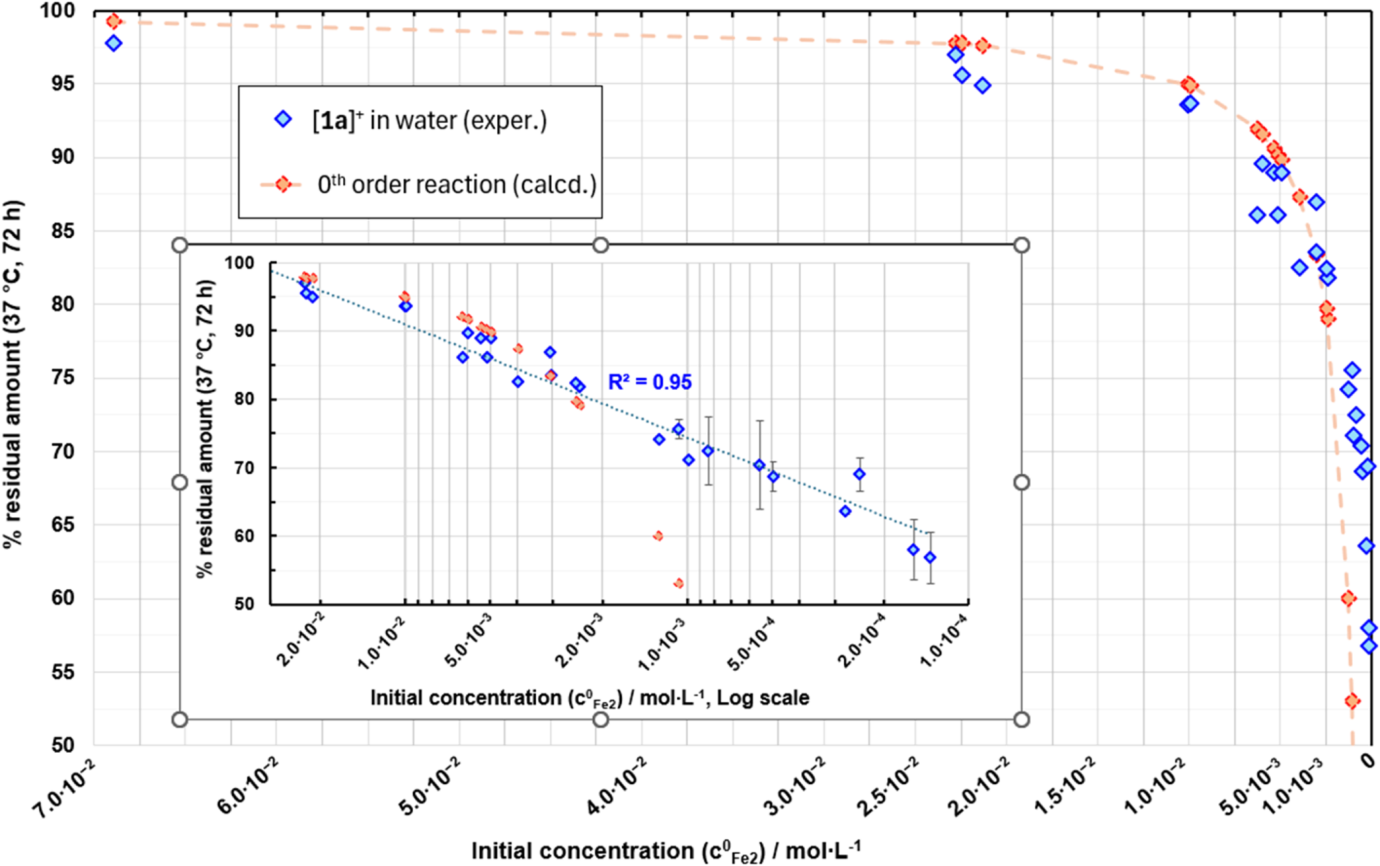
Blue squares: % residual amount of [**1a**]^+^ after 72 h at 37 °C vs decreasing initial molar concentration (−*c*_Fe2_^0^) of the aqueous solution. Data refer to Table S3. Dashed orange squares and line: calculated values according to zero-order kinetics with rate constant *k* = 7 μM/h. Inset shows a logarithmic plot (−Log *c*_Fe2_^0^ = *p*Fe^0^); the dotted blue line represents a linear fitting of the experimental data.

Complexes [**1b**–**e**]^+^ exhibited a qualitatively similar trend of increased extent of decomposition after 72 h at 37 °C upon dilution of the initial solution in the concentration range explored. Log plots of the % residual amount vs *c*_Fe2_^0^ data and the associated linear fittings (*R* ^2^ = 0.91–0.96) are shown in Figures 7 and S27–S31. All complexes are remarkably inert in ≥4 mM solutions as indicated by ≥85% starting material at the end of the experiments. At the lowest concentration range tested (*c*_Fe2_^0^ = 1–2·10^–4^ mol/L), the residual amount of [**1a**–**d**]^+^ varies between 64 and 54%. However, the scattering (and associated uncertainty) of the data prevents to appreciate any significant difference related to the substituent(s) of the aminocarbyne ligand. The similar reactivity of [**1a**–**d**]^+^ is substantiated by an almost identical slope of the linear fitting (Figure 7). Conversely, [**1e**]^+^ shows an increasing deviation from the other compounds below 2 mM, highlighting a higher reactivity. The residual amount of [**1e**]^+^ is *ca.* 30% at the lowest concentration tested (*ca.* 1·10^–4^ mol/L).

**Figure 7:**
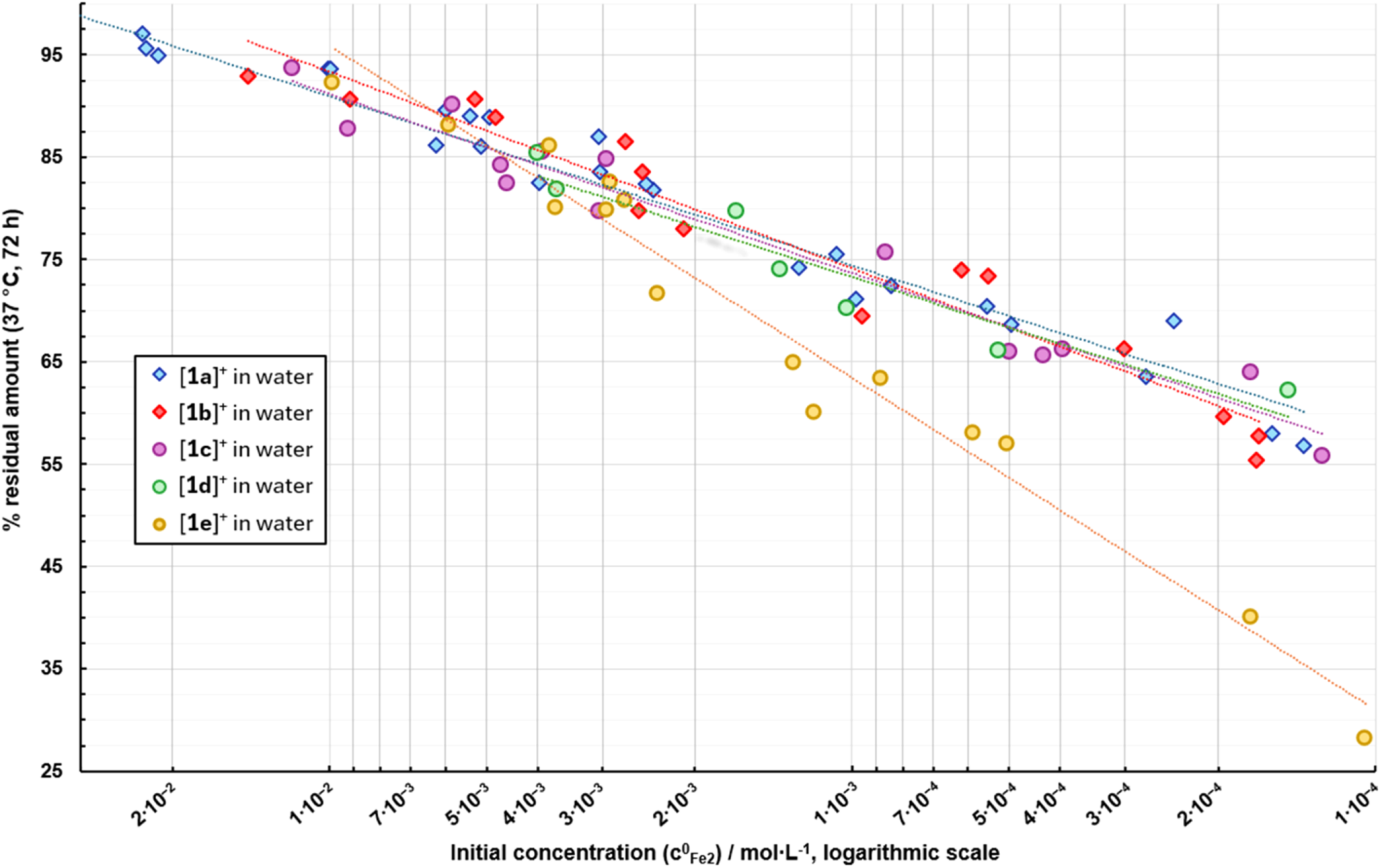
% Residual amount of [**1a**–**e**]^+^ after 72 h at 37 °C vs decreasing initial molar concentration of the aqueous solution (logarithmic scale, −Log  *c*_Fe2_^0^ = *p*Fe^0^). Data refer to Tables S3–S7. Dotted lines represent a linear fitting of the data.

A zero-order kinetic model^39^ with a rate constant of 7 μM/h provides a good fit of the data for *c*_Fe2_^0^ > 2.5 mM solutions (Figures 6 and S32). On the other hand, experimental data show an increasing deviation from the calculated values below 2 mM due to a lower consumption rate of [**1**]^+^. Collectively, results in the 2.0–0.1 mM range no longer follow a zero-order kinetics, regardless of the rate constant (Figure S33). This change in kinetic behavior limits the predictive ability of the regression of residual amount vs. initial concentration data to the millimolar range.

Subsequently, experiments were carried out on solutions of [**1a**–**c**]^+^ and [**1e**]^+^ in deuterated cell culture medium (DMEM-*d*) spiked with NaHCO_3_ to reach a near-physiological pH (pH = 7.7 for the analogous nondeuterated solution). Different initial concentrations (*c*_Fe2_^0^) were tested, ranging from the solubility limit of each compound to the quantitation limit of ^1^H NMR (≈1 mM). The reactivity at lower concentrations could not be explored since the UV–vis technique proved unsuitable with the final solution (Experimental). Data are collected in Tables S3–S5, S7 and refer to 37 °C, 72 h. The behavior of all tested compounds at high concentrations (*c*_Fe2_^0^ ≈ 1–2·10^–2^ mol/L) is characterized by a marked inertness (85–90% starting material). Then, as observed in pure water, the % residual amount progressively decreases upon dilution. Data were plotted against a decreasing logarithmic initial concentration (*p*Fe^0^) and were fitted with a linear regression (Figures S27–S29 and S31). For each complex, the % residual amount values are significantly lower than those collected in aqueous solution at similar concentration and the linear fitting is steeper, indicating a modest acceleration of the decomposition process in cell culture medium. However, compared to pure water, the greater data scattering (Figure S34), combined with the lack of information in the submillimolar range, prevents a clear reactivity order of the complexes from being established.

Overall, the experiments carried out with the triflate salts gave results in accordance with the general behavior of the nitrate analogues in both water and cell culture medium. Any possible influence of the counteranion (CF_3_SO_3_^–^ or NO_3_^–^) in the conditions explored is within the experimental error.

Solutions of [**1b**,**c**,**e**]­NO_3_ in D_2_O or in DMEM-*d* (*c*_Fe2_^0^ ≈ 4–6 mM) at 37 °C were also analyzed at 24 h time points up to 72 h (Tables S8–S10). In each case, the concentration of the starting cation [**1**]^+^ showed an approximately linear decrease over time (Figures S35, S37, and S39). Despite the limited concentration range explored (*i.e.*, the low conversion of the process within the investigated time frame), these data are consistent with zero-order kinetics. Kinetic constants of 7–11 μM/h and 13–18 μM/h in D_2_O or DMEM-d, respectively, were calculated by linear regression (Figures S36, S38, and S40), confirming a higher reactivity in cell culture medium.

### 2.4 Identification and Quantitation of Water-Soluble Products

The ^1^H NMR spectra recorded after 72 h at 37 °C (Section 2.3) were carefully examined in search of species deriving from the cyclopentadienyl or aminocarbyne ligands of [**1a**–**e**]^+^. The analysis was assisted by ^1^H–^13^C HMBC experiments and a collection of ^1^H NMR data in D_2_O of possible target compounds (SI). Results are included in Tables S3–S7. The secondary amine/ammonium ion arising from the hydrolysis of the *N*-carbyne bond in [**1**]^+^ (*i.e.*, Me_2_NH_2_^+^ for [**1a**]^+^, CyMeNH_2_^+^ for [**1b**]^+^, BnMeNH_2_^+^ for [**1c**]^+^, Bn_2_NH_2_^+^ for [**1d**]^+^, XylMeNH_2_^+^ for [**1e**]^+^) was identified for each diiron complex in both water and cell culture medium. Their formation is also evident from the ^1^H NMR spectra of D_2_O solutions of [**1a**–**e**]^+^ stored at room temperature for two months (Figures S41–S45). Primary amines/ammonium ions potentially deriving from the heterolytic cleavage of the other N–C bonds of [**1**]^+^ (*i.e.*, MeNH_3_^+^ for [**1a**–**c**,**e**]^+^, CyNH_3_^+^ for [**1b**]^+^ BnNH_3_^+^ for [**1c**,**d**]^+^, XylNH_3_^+^ for [**1e**]^+^) were not detected except in two isolated cases (from [**1c**]^+^ and [**1e**]^+^). Cyclopentadiene (CpH) was often observed in the final solutions of [**1a**–**e**]^+^ in both water and cell culture medium (Figure S46). The secondary amine, cyclopentadiene and carbon monoxide (Table S1) are the products of the total disruption of the coordination sphere of [**1**]^+^, leading to iron (hydr)­oxides (eq 1, Introduction). Considering the involvement of water and oxygen, the overall reaction can be described by eq 2 or by variants with one or more CO converted into CO_2_. The process comprises: (i) protonation of cyclopentadienyl ligands, (ii) hydrolysis of the *N*-carbyne bond with release of the ammonium ion, (iii) dissociation of carbonyl ligands, (iv) formal 4-electron reduction of one molecule of O_2_ coupled to the oxidation of Fe­(I) centers to Fe­(III).

2 $${\left[{\mathrm{Fe}}_{2}{\mathrm{Cp}}_{2}{\left(\mathrm{CO}\right)}_{3}\left({\mathrm{CNR}}_{2}\right)\right]}_{\left(\mathrm{aq}\right)}^{+}+O_{2\left(g/\mathrm{aq}\right)}+\left(2+n\right)H_{2}O_{\left(l\right)}\to 2{\mathrm{CpH}}_{\left(g/\mathrm{aq}\right)}+4{\mathrm{CO}}_{\left(g/\mathrm{aq}\right)}+{\left[R_{2}{\mathrm{NH}}_{2}\right]}_{\left(\mathrm{aq}\right)}^{+}+{\mathrm{Fe}}_{2}O_{3}\cdot nH_{2}O_{\left(s\right)}$$

However, the picture needs to be complemented by quantitative data. The ^1^H NMR yield of the secondary amines is rather variable and shows no correlation with the initial concentration of the solution (*c*_Fe2_^0^), and hence with the extent of decomposition of [**1**]^+^ after 72 h (Figure S47a). Specifically, formation of the secondary amine accounts for 10–40% of the consumed [**1a**–**d**]^+^ and less than 15% for [**1e**]^+^ (Figure S46b). Cyclopentadiene, when present, represents 0.5–2.5% of the starting organodiiron cation, corresponding to 3–15% of the conversion of [**1**]^+^ (Figure S46c). These figures are much lower than expected if the decomposition of [**1**]^+^ took place only via eq 2 (1:1 and 1:2 ratio between [**1**]^+^ and amine or CpH, respectively). While the yield of CpH is affected by its volatility (*T*_eb_ *ca.*, 41 °C), loss of the secondary amines in the gas phase should be minimal given their high boiling point and/or p*K*_a_ of the ammonium ions.^40^ In fact, experiments carried out with [**1a**]^+^ at higher temperature (50 and 70 °C, Section 2.6) actually recorded the highest yield of Me_2_NH_2_^+^ (50–75%; *T*_eb_ ≈ 8 °C for Me_2_NH). Overall, these data indicate that the total decomposition pathway (eq 2) accounts for a fraction of the observed decrease of [**1**]^+^.

The behavior in solution was integrated by monitoring the conductivity, pH and UV–vis spectra of [**1b**,**c**,**e**,**f**]­CF_3_SO_3_ (*c*_Fe2_^0^ ≈ 1.2·10^–3^ mol/L) at 37 °C at 24 h intervals up to 72 h. Data are compiled in Table S11 together with [RMeNH_2_]­X (R = Me or Xyl, X = I; R = Cy or Bn, X = Cl) and NaCF_3_SO_3_ as reference compounds. At these concentration levels, the conversion of [**1**]^+^ is still approximately linear over time. Kinetic constants of 5 and 9 μM/h were determined for [**1b**,**c**,**f**]^+^ and [**1e**]^+^, respectively (Figure S48), which are lower than those measured for 4–6 mM solutions (Section 2.3). The freshly prepared solutions of [**1**]­CF_3_SO_3_ are characterized by a near-neutral pH and a molar conductivity around 75–80 S·cm^2^·mol^–1^, as expected from a 1:1 electrolyte lacking Brønsted acid/base properties.^41^ A modest acidification and an increase in conductivity were observed over time (Figure S49), with the pH of the final solutions (72 h) ranging from 5.5 to 5.9. The partial [**1**]^+^ → [R_2_NH_2_]^+^ conversion described by eq 2 could in part explain these aspects, especially for aromatic amines. Nevertheless, a pH around 3.2–3.4 would be expected if the decomposition process was accompanied by the net release of one equivalent of H^+^ (calculated based on the conversion of [**1**]^+^ after 72 h assessed by UV–vis—Table S11).

In previously reported investigations, no evidence of FeCp complexes other than the starting material was found in the ^1^H NMR spectra of aqueous solutions of di- and tricarbonyl diiron­(I) aminocarbyne complexes [**1**]^+^ and [**2**]^+^ (Introduction). The ^1^H NMR spectra analyzed in this work make no exception. Raman spectroscopy could provide useful information in this respect, directly monitoring carbonyl or isocyanide species in solution,^42^ including [**1**]^+^, but relatively high concentrations are required (see the spectrum of 2·10^–2^ mol/L [**1a**]­NO_3_ in Figure S50). Moreover, soluble iron species may form that escape detection by ^1^H NMR, *e.g.*, paramagnetic and/or inorganic Fe­(II)/Fe­(III) complexes. Therefore, an indirect assessment of their relative amount was carried out. Specifically, solutions of [**1c**]­NO_3_ or [**1e**]­X (X = NO_3_, CF_3_SO_3_) in water or cell culture medium (*c*_Fe2_^0^ = 2.4–5.8 mM) were incubated at 37 °C for 72 h, then filtered and analyzed by ICP-OES (Table S12). The fraction of soluble iron was calculated with respect to the starting material and was found to be in good agreement with the % residual [**1**]^+^ determined by ^1^H NMR analyses at similar concentrations (based on Figures S29 and S31). If water-soluble Fe products were formed, the total iron content of the solution would remain constant regardless of the extent of decomposition of [**1**]^+^. Therefore, the tricarbonyl complexes [**1**]^+^ are the largely predominant Fe species in solution at these concentration levels, and most of the iron-containing products of the degradation process are expected to be found in the brown precipitate.

### 2.5 Characterization of Water-Insoluble Products

The next goal was the characterization of the insoluble products of the investigated process. Solutions of [**1a**–**c**,**e**,**f**]­CF_3_SO_3_ or [**1b**,**c**,**e**]­NO_3_ (2–8·10^–3^ mol/L initial concentration) in water or DMEM were filtered and kept at 37 °C. The pH of the culture medium was regulated either via the traditional phosphate buffer (DMEM-P; 100 mM H_2_PO_4_^–^/HPO_4_^2–^, pH ≈ 7.2) or with sodium bicarbonate. The latter medium was tested at the standard concentration (DMEM-C; 44 mM NaHCO_3_, pH ≈ 8.3)^18^ and 10-fold diluted (DMEM-C-dil). The decomposition of [**1**]^+^ is higher at lower concentration but the amount of precipitate decreases accordingly, making its isolation more difficult. To collect a larger amount of material, the incubation time was increased to 7 days or more, compared to the standard 3 days (72 h). The resulting brown solids were separated by centrifugation, washed thoroughly with water and dried under vacuum. Related experiments were carried out with FeSO_4_ and (NH_4_)_2_Fe­(SO_4_)_2_·6H_2_O (Mohr’s salt) as benchmark inorganic iron­(II) compounds. The collected solids, together with reference Fe compounds, were analyzed by IR, Raman and for the CHNS content (Figures S51–S63 and Table S13).

Inorganic components predominate in all samples, regardless of the starting material and incubation conditions, as elements other than CHNS–mostly Fe and O–account for 75–95% by mass. All samples show broad IR absorptions in the 3200–3400 and 1590–1630 cm^–1^ regions, corresponding to vibrations of H_2_O and OH^–^ ligands of iron­(III) oxyhydroxides.^43,44^ Additional Fe–OH bands are sometimes present in the 750–1000 cm^–1^ range. Samples of FeO­(OH)·*n*H_2_O obtained by treating aqueous solutions of FeSO_4_ or Fe_2_(SO_4_)_3_ with NaOH^45^ show a strong IR absorption at 905 cm^–1^, which is not observed in the solids collected from [**1**]^+^ (Figure 8a). Raman spectra of selected solids obtained from the incubation of [**1a**–**c**]­CF_3_SO_3_ in water or DMEM-C-dil are relatively similar in the 175–1250 cm^–1^ region, resembling α-Fe_2_O_3_,^44^ while the Raman profile of solids precipitated from Mohr’s salt in DMEM-C-dil or Fe_2_(SO_4_)_3_ + NaOH are comparable to ferrihydrite, a type of iron­(III) oxyhydroxide (Figure S54).^46^

Solids collected from DMEM-P are characterized by a strong IR absorption around 985–1020 cm^–1^ matching that of iron­(III) phosphate (Figure 8b).^47^ Reference samples of FePO_4_·*n*H_2_O were obtained by addition of either FeSO_4_ or Fe_2_(SO_4_)_3_ to a phosphate buffer (NaH_2_PO_4_/Na_2_HPO_4_) solution. A similar medium-strong IR band around 1000 cm^–1^ is also observed in the products of incubation of FeSO_4_, Mohr’s salt and some of the tested [**1**]^+^ in DMEM-C (0.77 mM phosphate).^18^ The precipitation of iron­(III) phosphate represents a *direct* intervention of pH-controlling species in the speciation of a transition metal, which can be mitigated by avoiding the use of phosphate buffer.

On the other hand, solids obtained from bicarbonate-treated DMEM do not contain carbonate as indicated by the absence of the characteristic IR bands at 1360 and 853 cm^–1^ (Figure 8c).^48^ IR spectra and carbon content of the solids obtained from FeSO_4_/NaHCO_3_, FeSO_4_/Na_2_CO_3_ and Fe_2_(SO_4_)_3_/Na_2_CO_3_ confirm that iron carbonate species are neither kinetically nor thermodynamically favored over iron­(III) oxyhydroxides in aerobic aqueous solution.

In addition to these aspects, samples obtained from [**1**]^+^ feature a variable carbon and nitrogen content (C + N 6–33% by mass) and peculiar IR absorptions, which are mainly determined by the starting aminocarbyne complex. Changing the counteranion (CF_3_SO_3_^–^ *vs*. NO_3_^–^) or the incubation medium gave relatively similar results in this context, with few exceptions. Specifically, solids derived from [**1e**,**f**]^+^ feature a relatively high C (18–30%), H (2.5–4.0%) and N (1.1–3.0%) content, often associated with a pattern of five IR bands in the 1700–2200 cm^–1^ region attributed to carbonyl and isocyanide ligands (*e.g.*, Figure 8d). On the other hand, the precipitates derived from [**1a**,**b**]^+^ contain lower amounts of C (typically 7–13%) and N (typically 0.2–1.5%), and show no IR bands in the same region. The *N*-benzyl derivative [**1c**]^+^ is peculiar as it behaves like the dialkyl aminocarbyne analogues [**1a**,**b**]^+^ in water and like the *N*-aryl counterparts [**1e**,**f**]^+^ in DMEM. Save for the above-mentioned iron­(III) phosphate precipitation, this is the only case - at the investigated concentration levels—where the incubation medium affects, at least indirectly, the insoluble decomposition products.

**Figure 8:**
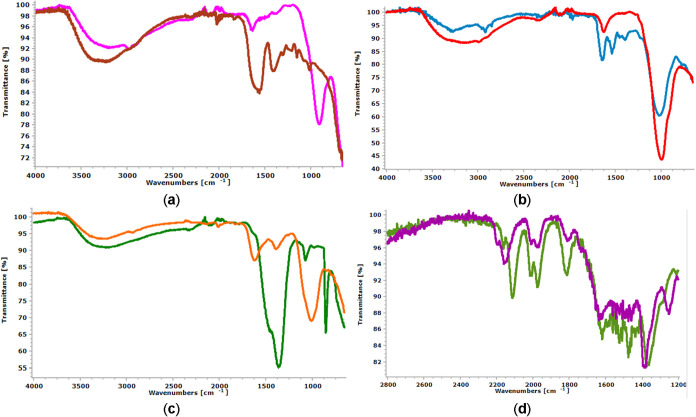
Comparison of IR spectra of solids precipitated from aqueous solutions. (a) Incubation of [**1c**]­NO_3_ in water (brown line) vs FeO­(OH)·*n*H_2_O obtained from Fe_2_(SO_4_)_3_ + NaOH (pink line). (b) Incubation of [**1a**]­CF_3_SO_3_ in DMEM-P (red line) vs FePO_4_·*n*H_2_O (cyan line) obtained from Fe_2_(SO_4_)_3_ + phosphate buffer. (**c**) Incubation of [**1b**]­NO_3_ in DMEM-C (orange line) vs FeO­(OH)·*n*H_2_O·Fe­(CO_3_) obtained from FeSO_4_ + NaHCO_3_. (d) Incubation of [**1e**]­NO_3_ in water (olive green line) vs incubation of [**1c**]­CF_3_SO_3_ in DMEM-C-dil (violet line).

Interesting considerations emerge after comparative experiments with FeSO_4_ and Mohr’s salt. First, no appreciable amount of precipitate was formed when these Fe­(II) compounds were incubated in water. The oxidation of Fe_(aq)_^2+^ to Fe­(OH)_3_ by O_2_ is thermodynamically favored at 25 °C except in highly acidic solution,^49,50^ meaning that the reaction is kinetically hampered. Therefore, the decomposition process of [**1**]^+^ does not generate significant amounts of Fe_(aq)_^2+^ ions. Second, the brown solids formed from DMEM-C solutions of both FeSO_4_ and Mohr’s salt at 37 °C also contain a substantial fraction of C (11–13%) and N (1.3–2.3%). Such organic content probably arises from components of the culture medium that are absorbed or trapped in the precipitate,^51^ since it decreases upon dilution (from DMEM-C to DMEM-C-dil). This phenomenon appears to be less important for [**1**]^+^, as a certain amount of C and N is already present in the precipitates collected from water, and is often comparable to that in cell culture media (DMEM-C, DMEM-P or DMEM-C-dil). Moreover, organometallic species are present in the solids obtained from [**1c**,**e**,**f**]^+^. The hypothesis that most of the CN content of the precipitates derives from [**1**]^+^ is consistent with the observation that only a fraction of the aminocarbyne complexes undergoes full decomposition (eq 2) releasing inorganic iron species, the corresponding amine and CpH (Section 2.4). A test carried out with [**1e**]­NO_3_ in DMEM-C at 60 °C led to a reduction of *ca.*, 30% of the CHN content of the resulting solid, in agreement with the higher yield of Me_2_NH_2_^+^ obtained from [**1a**]^+^ upon increasing the temperature (*vide supra*).

In order to have more insights on their organometallic components, the samples obtained from [**1c**,**e**,**f**]^+^ were extracted with CH_2_Cl_2_ and analyzed by IR and ESI-MS (Figures S64–S70). The IR spectra show the same five-band pattern as observed in the solid state, with changes in the relative intensities depending on the starting material and the conditions. The most intense MS peaks correspond to tris-isocyanide piano stool iron­(II) complexes [CpFe­(CNR)_3_]^+^ ^52^ and monoisocyanide aminocarbyne diiron­(I) complexes [Fe_2_Cp_2_(CO)­(CNR)­(μ-CO)­{μ-CNMe­(R)}]^+^ (R = Bn, Xyl, 4-C_6_H_4_OMe).^27,36,53^ Minor pattern of peaks account for other Fe_2_, Fe_3_ ^54^ and possibly Fe_4_ species.

In summary, the complexity of the water-insoluble products indicates that multiple pathways are operative during the breakup of [**1**]^+^ in water or cell culture medium. Iron­(III) oxyhydroxides, resulting from the total disruption of the coordination sphere, are unlikely to have *direct* biological implications, as their formation in a cellular environment at μM concentration would be negligible. The inorganic Fe species released from the organodiiron scaffold would instead be intercepted by the biological milieu and may play an important role in the cytotoxic mechanism and other biological effects. It has been demonstrated that ferrocene (*in vivo*)^55^ as well as ferrocenium salts (in aqueous or organic solution)^56^ are metabolized by O_2_ to ferrocenol (hydroxyferrocene) which then decomposes to inorganic Fe. Similarly, ferrocene derivatives are decomposed by H_2_O_2_ with liberation of Fe_(aq)_^2+^ ions.^57^ The formation of poorly soluble organometallic species from the rearrangement of [**1**]^+^ in aqueous media may represent an alternative process relevant to biological applications, which will be the subject of a future investigation.

### 2.6 Mechanistic Experiments

The decomposition of aminocarbyne complexes in aqueous solution was further investigated in the presence of different species and applying different conditions, such as oxidizing or decarbonylating agents, temperature, pH, light. UV–vis and ^1^H NMR experiments with [**1a**,**c**,**e**]^+^ as representative complexes were carried out as previously described (Sections 2.3–2.4). Results are expressed in terms of % residual starting material after 72 h (Tables S14–S16; plotted vs *p*Fe^0^ in Figures 9 and S71–S72) and are compared with the established reactivity trend in water (or DMEM) in standard conditions (ambient air, light, 37 °C).

**Figure 9:**
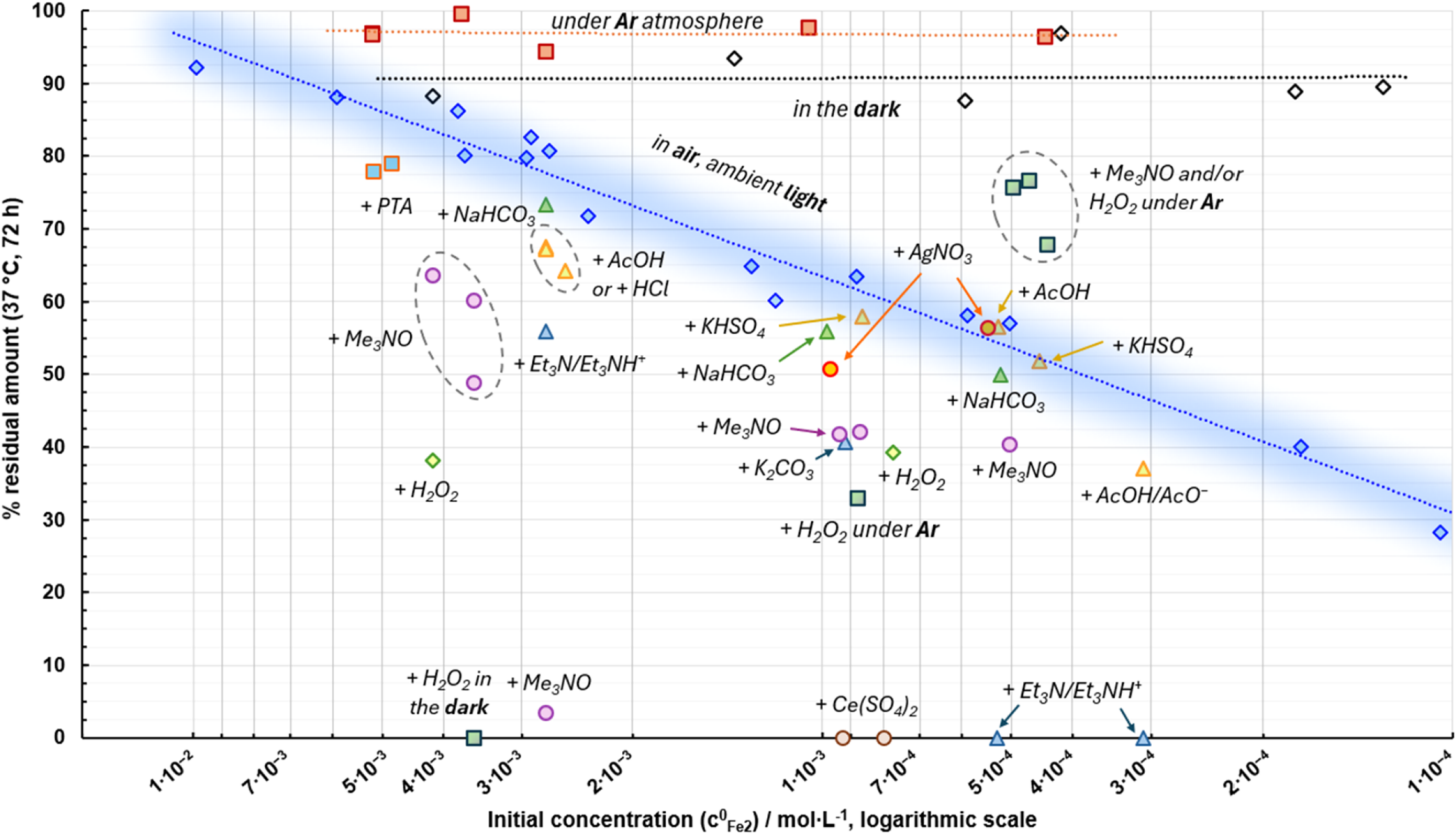
% Residual amount of [**1e**]^+^ after 72 h at 37 °C vs decreasing initial molar concentration of the aqueous solution (logarithmic scale, −Log *c*_Fe2_^0^ = *p*Fe^0^) in different conditions, compared to the standard system (H_2_O, air/ambient light, 37 °C; blue points and linear fit). Data refer to Tables S16 and S19.

Tests under N_2_ or Ar atmosphere were carried out using an alkaline pyrogallol solution as a visual sensor to check for the complete exclusion of air in the system.^58^ Complexes [**1a**,**c**,**e**]^+^ were found to be remarkably inert under anaerobic conditions at 37 °C over a wide concentration range (1.1·10^–2^–4.4·10^–4^ mol/L), in both water and DMEM (generally ≥93%), providing a strong indirect evidence of the role of O_2_ as oxidizing agent. It should be noted that the change in the kinetic behavior of the decomposition process (Figures 6 and S32) occurs as the diiron complex approaches the concentration of dissolved oxygen (*c*_Fe2_^0^ < 1.5 mM; *c*_O_2__ ≈ 0.20 mM at 37 °C with *p*_O_2__ = 0.21 atm).^59^

For comparison, silver nitrate, cerium­(IV) sulfate and hydrogen peroxide were tested as alternative oxidizing agents. While AgNO_3_ (*E*°_Ag^+^/Ag_ = +0.79 V vs SHE) had no remarkable effect on the extent of decomposition of [**1e**]^+^ after 72 h at 37 °C in the sub mM range, Ce­(SO_4_)_2_ (*E*°_Ce^4+^/Ce^3+^_ = +1.61 V vs SHE) caused an immediate and quantitative decomposition. These outcomes agree with the potential for the irreversible monoelectron oxidation of [**1b**,**f**]^+^ in aqueous solution (*E*_ox_ ≈ +1.3 V vs SHE)^25^ as well as the first oxidation potential of [**1a**,**e**]^+^ in MeCN (*E*_ox_ ≈ +1.5–1.6 V vs SHE).^60^

The addition of H_2_O_2_ (1.0–2.5 equiv) resulted in a 1.5- to 3.5-fold increase in the consumption of [**1a**,**c**,**e**]^+^ after 72 h. The effect appears to be concentrated within the first 24 h (Figures S73–S74, with [**1e**]­NO_3_), presumably due to the progressive decomposition of H_2_O_2_ promoted by temperature and catalyzed by traces of inorganic iron species formed during the process.^61^

Temperature variation in the 25–70 °C range at different initial concentrations (*c*_Fe_^0^ ≈ 2.0·10^–2^, 5.5·10^–3^ and 3.7·10^–3^ mol/L) had a marginal effect on the % residual amount of [**1a**]^+^ after 72 h, as compared to data obtained at 37 °C. The lack of acceleration with increasing temperature could also be related to the decreasing solubility of O_2_ in water (*ca.* 0.26 mM at 25 °C *vs*. 0.17 mM at 70 °C with *p*_O_2__ = 0.21 atm).^59^

The effect of pH was checked on aqueous (H_2_O or D_2_O) solutions of [**1a**,**c**,**e**]­NO_3_ treated with hydrochloric acid (1.6), potassium hydrogen sulfate (2.4), acetic acid (3.0), acetate buffer (4.8), sodium bicarbonate (8.3), triethylammonium buffer (10.8), triethylamine (11.7) or potassium carbonate (11.1) (approximate pH in H_2_O in parentheses). The residual starting material after 72 h at 37 °C in acidic or near-neutral solutions is comparable to that assessed in water. Conversely, the decomposition process of [**1**]^+^ is greatly accelerated in basic solution (pH ≥ 11). In these conditions, the hydroxide ion (OH^–^/OD^–^) has a similar concentration (1–5 mM) to the diiron complex, and their interaction is kinetically favored by electrostatic reasons. Moreover, on thermodynamic grounds, the equilibrium concentration of soluble Fe­(III) species decreases with increasing pH due to the limiting solubility of the corresponding hydroxide.^49^

The robustness of the diiron scaffold in acidic aqueous solution was also verified on solutions of [**1d**–**f**]­CF_3_SO_3_ and HCl (*ca.*, 10 mM and 0.1 mol/L, respectively) kept at 80 °C for 48 h. It is noteworthy that neutral, water-insoluble chloride complexes [Fe_2_Cp_2_Cl­(CO)­(μ-CO)­{μ-CNR­(R′)}], **3**,^62^ were not detected, either in this case or in the solids precipitated from DMEM solutions (0.12 mol/L Cl^–^,^18^ Section 2.5). These results indicate that direct CO/Cl^–^ exchange on [**1**]^+^ (eq 3) is not a viable pathway.

3 $${\left[{\mathrm{Fe}}_{2}{\mathrm{Cp}}_{2}{\left(\mathrm{CO}\right)}_{2}\left(\mu ‐\mathrm{CO}\right)\left(\mu ‐{\mathrm{CNRR}}^{\prime }\right)\right]}_{\left(\mathrm{aq}\right)}^{+}+{\mathrm{Cl}}_{\left(\mathrm{aq}\right)}^{-}\to {\left[{\mathrm{Fe}}_{2}{\mathrm{Cp}}_{2}\mathrm{Cl}\left(\mathrm{CO}\right)\left(\mu ‐\mathrm{CO}\right)\left(\mu ‐{\mathrm{CNRR}}^{\prime }\right)\right]}_{\left(s\right)}+{\mathrm{CO}}_{\left(g\right)}$$

Addition of trimethylamine *N*-oxide (Me_3_NO; 1–12 equiv), a well-known decarbonylating agent for metal complexes,^63^ resulted in an increased consumption of [**1a**,**c**,**e**]^+^ after 72 h at 37 °C as compared to the same initial concentration in pure water. These results uphold the dissociation of carbonyl ligand(s) as a key step in the decomposition mechanism of [**1**]^+^ in aqueous media. However, the process remains relatively slow despite the large excess of Me_3_NO in solution, resulting in a significant fraction of [**1**]^+^ at the end of the experiment (*e.g.*, *ca.*, 55% [**1a**]^+^ with *c*_Fe2_^0^ ≈ 4 mM and 3 equiv of Me_3_NO). In other words, Me_3_NO is not particularly effective for carbonyl removal in neat aqueous solution. Conversely, reactions of [**1**]^+^, Me_3_NO and monodentate ligands such as pyridines, phosphanes, isocyanides and nitriles are usually rapid and quantitative at room temperature under near-stoichiometric conditions (typically 1.2–1.3 equiv Me_3_NO),^27,26,29,30^ suggesting that attack of Me_3_NO on a terminal carbonyl is assisted by the entering ligand.

Overall, the collected data (Table S17) indicate that the contribution of Me_3_NO to the process depends on the specific aminocarbyne complex and the experimental conditions. The % residual amount of [**1a**,**c**]^+^ decreases with increasing initial amount of Me_3_NO, while [**1e**]^+^ is relatively less influenced. The ratio of Me_3_NH^+^ to decomposed [**1**]^+^ ranges from 0.5 to 2.0, most often between 0.7 and 1.2, without any clear correlation with the initial concentration of [**1**]^+^ or the initial Me_3_NO/[**1**]^+^ molar ratio. Since Me_3_NO-free and Me_3_NO-promoted decomposition processes occur simultaneously, these values do not define a reaction stoichiometry. Selected experiments were monitored at 24 h time points, showing a linear decrease of [**1a**]^+^ and formation of Me_3_NH^+^, thus indicating a constant ratio of Me_3_NH^+^ to decomposed [**1a**]^+^ over time (Figure S75).

In order to confirm that Fe–CO dissociation is pivotal in the activation process of diiron aminocarbyne complexes in aqueous solution, as well as to trap the intermediate(s) generated, targeted experiments were carried out with [**1a**,**c**,**e**]­NO_3_ and 1,3,5-triaza-7-phosphadamantane (PTA) in D_2_O or DMEM-*d*. PTA is water-soluble, air stable and imparts water-solubility to related metal complexes.^64^ Thus, D_2_O or DMEM solutions of [**1a**,**c**,**e**]­NO_3_ and PTA (3–4 equiv) were incubated at 37 °C up to 144 h (6 days) and analyzed by ^1^H and ^31^P NMR (Table S18 and Figures S76–S78). The consumption of [**1a**,**c**,**e**]^+^ was accompanied by the rise of new ^1^H and ^31^P signals belonging to the corresponding mono-PTA derivative of formula [Fe_2_Cp_2_(CO)­(PTA)­(μ-CO)­{μ-CNMe­(R)}]^+^ (R = Me, [**2a**]^+^, Bn, [**2c**]^+^, Xyl, [**2e**]^+^).^22,24,29^ The formation of [**2**]^+^ from [**1**]^+^ and PTA is the result of carbonyl/phosphane ligand substitution (Scheme 2a). The sum of residual [**1**]^+^ and [**2**]^+^ after 72 h accounts for 95–99% of the starting material, decreasing slightly in the successive 72 h (90–95% after 144 h). The higher inertness of the PTA derivatives [**2**]^+^ in aqueous solution with respect to the tricarbonyl complexes^22,29^ is essential to demonstrate that the monodecarbonylated species generated from [**1**]^+^ is almost quantitatively trapped by PTA coordination. The residual amount of [**1**]^+^ in solution after 72 h is only slightly lower than expected in the absence of PTA. A modest acceleration upon introduction of a good entering ligand (PTA in place of H_2_O) is consistent with a dissociative mechanism.^65^

**Scheme 2:**
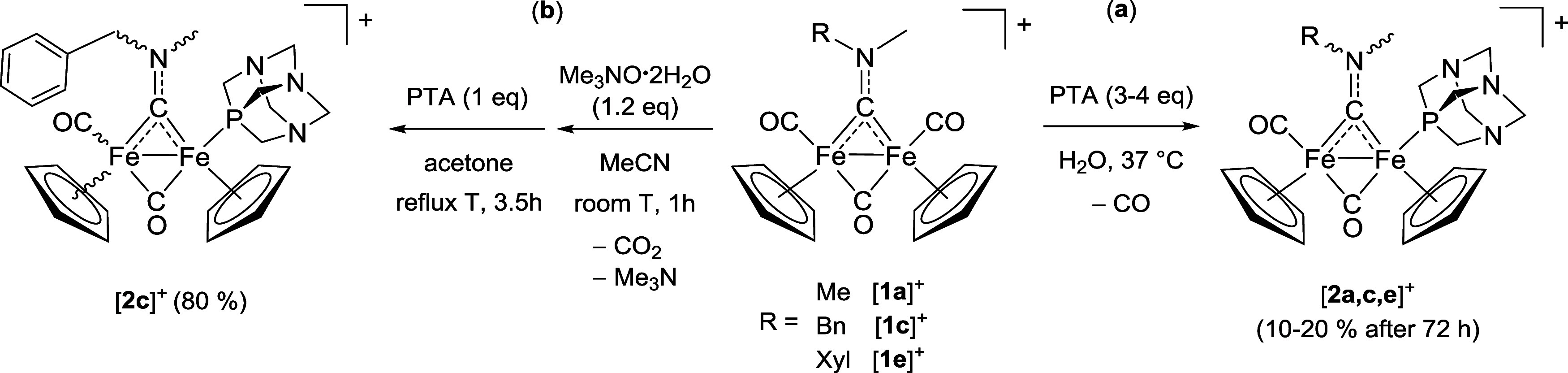
Formation of the PTA Complexes [**2**]^+^ from [**1**]^+^ and PTA upon Slow Decarbonylation in Aqueous or DMEM Solution at 37 °C (Path **a**, NO_3_^–^ as Anion) or with Me_3_NO via a Two-Step Process (Path **b**, CF_3_SO_3_^–^ as Anion)^a^

a Wavy bonds represent *cis*/*trans* or *E*/*Z* isomerism. NMR or isolated yield in parentheses.

Compounds [**2a**,**e**]­CF_3_SO_3_ were previously synthesized from [**1a**,**e**]­CF_3_SO_3_ by a two-step procedure involving Me_3_NO-assisted CO/MeCN replacement followed by MeCN/PTA exchange in refluxing acetone.^24,29^ The unprecedented [**2c**]­CF_3_SO_3_ was prepared from [**1c**]­CF_3_SO_3_ according to the same method (Scheme 2b). Following alumina chromatography, the compound was isolated as a green-brown solid in 80% yield and characterized by IR (solid state, CH_2_CH_2_) and ^1^H, ^13^C and ^31^P NMR (acetone-*d*_6_); spectra are shown in Figures S79–S84. Two major sets of NMR signals of [**2c**]^+^ correspond to isomers featuring a *cis* arrangement of the cyclopentadienyl ligands and an *E* or *Z* stereochemistry of the carbyne-nitrogen partial double bond, with the former slightly prevalent. Minor (<10%) amounts of *trans* isomers were later removed through a more careful chromatography. The crystal structure of *cis*-*E*-[**2c**]­CF_3_SO_3_ was ascertained by X-ray diffraction (Figure 10). The introduction of a stronger σ-donor/weaker π-acceptor PTA ligand generates a marked asymmetry of the μ-CO ligand [Fe1–C2 2.001(4) Å, Fe2–C2 1.888(4) Å] and a less pronounced one for the bridging aminocarbyne [Fe1–C3 1.892(4) Å, Fe2–C2 1.859(4) Å]. Similar bonding parameters have been found in related [Fe_2_Cp_2_(CO)­(PTA)­(μ-CO)­{μ-CNR­(R’)}]^+^ complexes.^22,24,29^ The PTA ligand of [**2c**]^+^ and the aminocarbyne substituents display *E* stereochemistry, as in the related [Fe_2_Cp_2_(2-thienylcarbonyl)­(CO)­(μ-CO)­{μ-CNMe­(Bn)}].^66^

**Figure 10:**
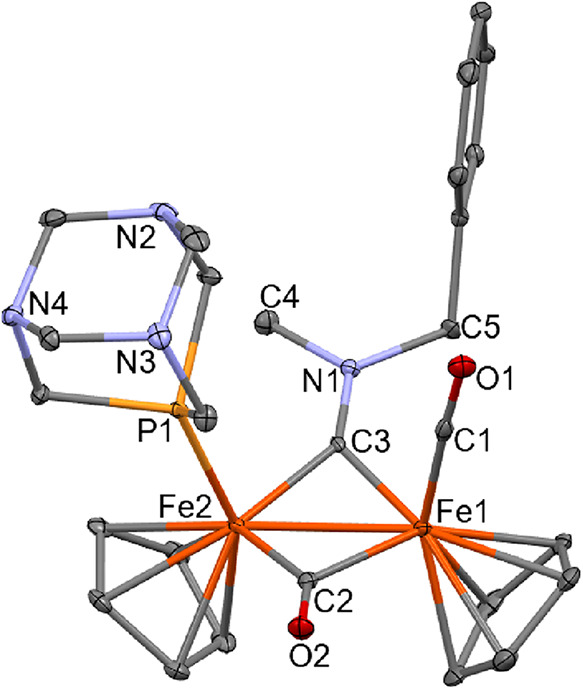
View of the structure of *cis*-*E*-[**2c**]^+^ in [**2c**]­CF_3_SO_3_. Displacement ellipsoids are at the 30% probability level. H atoms have been omitted for clarity. Main bond distances (Å) and angles (°): Fe1–Fe2 2.5156(8), Fe1–C1 1.764(5), Fe2–P1 2.2140(12), Fe1–C2 2.001(4), Fe2–C2 1.888(4), Fe1–C3 1.892(4), Fe2–C3 1.859(4), C1–O1 1.113(6), C2–O2 1.158(5), N1–C3 1.301(5), N1–C4 1.472(6), N1–C5 1.496(5), Fe1–C1–O(1) 177.2(4), Fe1–C2–Fe2 80.54(16), Fe1–C3–Fe2 84.23(17), C3–N1–C4 122.6(4), C3–N1–C5 122.9(4), C4–N1–C5 114.2(3).

Based on the collected data, the dissociation of one carbonyl ligand represents the first step as well as the rate-determining step of the activation process for diiron aminocarbyne complexes [**1**]^+^. Coordination of water to the vacant site should provide an elusive aqua-complex of the type [Fe_2_Cp_2_(H_2_O)­(CO)­(μ-CO)­{μ-CNRR′}]^+^, [**1** ^**H_2_O**^]^+^ (eq 4), which then interacts with O_2_. Otherwise, this reactive intermediate is trapped by coordinating species such as PTA (Scheme 2a).

4 $$[{\mathrm{Fe}}_{2}{\mathrm{Cp}}_{2}{\left(\mathrm{CO}\right)}_{2}\left(\mu ‐\mathrm{CO}\right)\left({\mu ‐\mathrm{CNRR}}^{\prime }\right){]}^{+}+H_{2}O\to \{{\mathrm{Fe}}_{2}{\mathrm{Cp}}_{2}\left(\mathrm{CO}\right)\left(H_{2}O\right)\left(\mu ‐\mathrm{CO}\right)\left(\mu ‐{\mathrm{CNRR}}^{\prime }\right){\}}^{+}+\mathrm{CO}$$

Such simple ligand substitutions have been rarely reported for this class of compounds, as the carbonyl ligands are tightly bound to the iron­(I) centers. Prior examples include a CO/CNBn exchange in refluxing benzene^53^ and CO/solvent exchange in MeCN or DMSO promoted by UV irradiation (all monosubstitutions).^24,67^ In this regard, the role of ambient light in the process was assessed by keeping aqueous solutions of [**1a**–**f**]^+^ (NO_3_^–^ or CF_3_SO_3_^–^ salts) in the dark at 37 °C (Table S19). A remarkable (89–96%) amount of starting material was detected after 72 h across the entire concentration range investigated (*c*_Fe2_^0^ from 0.13 to 4.2 mM). The stark difference with respect to the extent of the decomposition under ambient light (Figures 9 and S70–S71) clearly indicates that the carbonyl removal step is photoactivated. The photodissociation of a Fe–CO bond and the subsequent coordination of a solvent molecule is a known phenomenon.^68^ In this regard, piano-stool Fe­(II) complexes of the type [FeCp­(CO)_2_X] (X = Cl, Br, I, maleimidato/succinimidato, CH_2_CONH_2_) undergo CO release upon visible light illumination in aqueous solution, followed by decomposition.^69^

Interestingly, a significant decomposition after 72 h at 37 °C was observed for [**1c**,**e**]^+^ in the presence of H_2_O_2_ and/or Me_3_NO under Ar as well as in the dark (Tables S16 and S17). Therefore, H_2_O_2_ and Me_3_NO do not only accelerate the process under ambient conditions but also enable alternative decomposition pathways in the absence of O_2_ or light.

### 2.7 DFT Investigation

The fragmentation pattern leading to the formation of secondary amines and iron­(III) (oxy)­hydroxides (eq 2) is a fundamental component of the activation process of [**1**]^+^ in aqueous media. This complex transformation begins with dissociation of a CO ligand, promoted by ambient light, followed by the interaction with O_2_. The remainder of the mechanism is largely unknown, as no intermediate Fe species has ever been detected in solution.

In order to gain insights on this process, a DFT study was carried out using the dimethylaminocarbyne complex [**1a**]^+^ as representative compound. Results are summarized in Figure 11 and Tables S20–S21. The DFT-optimized transition state for the substitution of a terminal carbonyl of [**1a**]^+^ with O_2_ reveals that the associated activation energy (**TS1alt**) is too high to be a viable option (Δ*G* ^⧧^ = 53.9 kcal/mol). Likewise, the substitution of a terminal carbonyl of [**1a**]^+^ with a water molecule is kinetically hindered (**TS1_s**, Δ*G* ^⧧^ = 48.9 kcal/mol). An alternative possibility is that the diiron complex absorbs a photon, changing its spin state from a singlet to a high energy triplet (**MeMe_t**, Δ*G* = 29.1 kcal/mol). In **MeMe_t**, the spin density is distributed asymmetrically over the two iron centers. One iron atom exhibits a spin population of 0.70 and an Fe–CO bond length of 1.76 Å, while the other shows a higher spin population of 1.31, accompanied by an elongation of the Fe–CO bond to 1.85 Å. This asymmetry weakens the strong-field character of the CO ligand, thereby stabilizing higher-spin electronic configurations. Furthermore, the Cp ring is no longer planar, as evidenced by the unequal Fe–C bond distances. This distortion further lowers the local symmetry around the metal center, facilitating the stabilization of the triplet state. Using **MeMe_t** as the new energy reference, the activation barrier of the CO/H_2_O substitution becomes smaller and reasonable for a slow reaction (**TS1_t**, Δ*G* = 21.0 kcal/mol). Also **TS1_t** was optimized as a triplet state, as well as the aqua-complex **A_t** (Δ*G* = 6.1 kcal/mol), which then relaxes to the more stable singlet state **A_s** (Δ*G* = −2.5 kcal/mol). At this stage, water can be substituted by a dioxygen molecule through **TS2_t** (Δ*G* = 25.5 kcal/mol), leading to the Fe–O_2_ intermediate **B_t** (Δ*G* = 1.0 kcal/mol). The spin of the transition state is dictated by the presence of dioxygen, which is stable in the triplet state. The coordinated dioxygen forms a reactive peroxide moiety, which attacks the aminocarbyne carbon (**TS3_t**, Δ*G* = 20.6 kcal/mol), yielding intermediate **C_t** (Δ*G* = −41.3 kcal/mol) containing a bridging dimethylaminoacyl ligand. A similar sequence of steps (O_2_ coordination and intramolecular attack of a Fe–O_2_ moiety on a cyclopentadienyl ligand) has been proposed for the decomposition of [FeCp_2_]^+^.^56^ The bridging ligand can then be readily hydrolyzed (**TS4_t**, Δ*G* = −29.0), liberating dimethylcarbamic acid and generating the diiron intermediate **D_t** (Δ*G* = −44.4 kcal/mol), which contains a terminal carbonyl, a bridging carbonyl and a bridging oxide. Dimethylcarbamic acid is expected to decompose into CO_2_ and dimethylamine. The remaining steps likely involve further oxidation of the diiron residue and hydrolysis of the cyclopentadienyl ligands.

**Figure 11:**
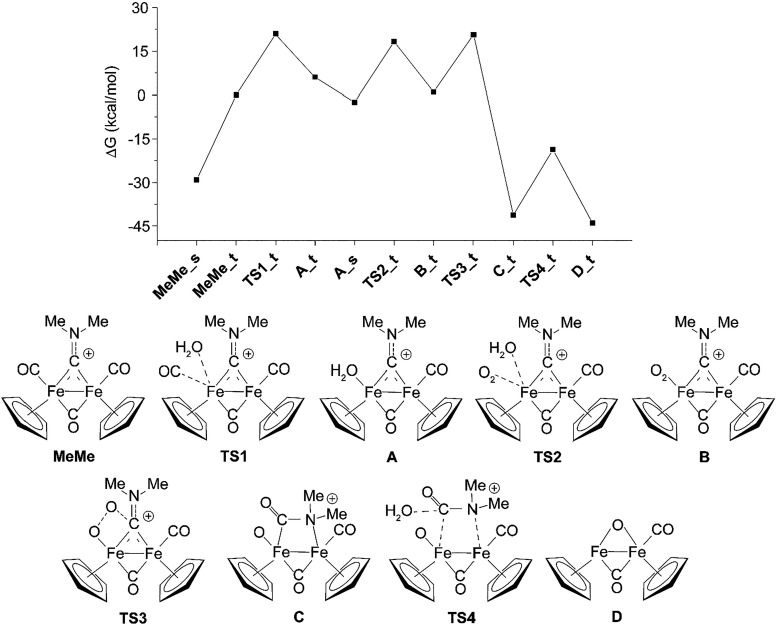
Optimized geometries and relative Gibbs free energies for the light-induced decomposition of [**1a**]^+^ (**MeMe**) with O_2_ and H_2_O.

Overall, these results align with previous DFT investigations in indicating an intrinsic stability of aminocarbyne complexes, and particularly of the Fe–Fe core, in the ground state.^36,70^

### 2.8 Decomposition of Chloro-Complexes

The first step of the decomposition process of [**1**]^+^ in aqueous solution is the dissociation of a carbonyl ligand with presumable concomitant formation of an aqua-complex [**1** ^**H_2_O**^]^+^ (Scheme 3a). This elusive species is substantially less stable than the tricarbonyl precursor (*ca.* +27 kcal/mol for R = R′ = Me, Section 2.7). Very few FeCp complexes containing a carbonyl and an aqua ligand have been reported, among which [Fe­(η^5^-C_5_R_5_)­(CO)_2_(H_2_O)]^+^ (R = H, Me, Ph and R_5_ = Me_4_Et).^71^ Chloride displacement from dicarbonyl compounds [Fe_2_Cp_2_Cl­(CO)­(μ-CO)­{μ-CNR­(R’)}], **3**, promoted by silver salts,^72^ may represent an alternative pathway to generate the same reactive species (Scheme 3b).

In order to test this hypothesis, complexes [Fe_2_Cp_2_Cl­(CO)­(μ-CO)­{μ-CNR­(R′)}] (R = Me, R′ = Bn, **3c**; R′ = Xyl, **3e**; R′ = 4-C_6_H_4_OMe, **3f**; R = R′ = Bn, **3d**) were prepared from the respective tricarbonyl precursors, an excess of trimethylamine *N*-oxide and LiCl in refluxing acetone (Scheme 4). Compounds **3c**–**f** were purified by alumina chromatography and isolated as brown solids in 50–76% yield. This one-pot method is novel for **3c**,**f**,**e**, which were previously prepared using two-step procedures.^62,73,74^ On the other hand, the bis-benzyl complex **3d** is unprecedented.

Compounds **3d**–**f** were characterized by ^1^H NMR (acetone-*d*_6_) and IR (solid state and CH_2_Cl_2_) while **3d** was further characterized by CHN analyses and ^13^C NMR. IR and NMR spectra are reported in Figures S85–S96. Two sets of signals are present in the NMR spectra of **3d**, related to *cis* and *trans* diastereomers, with the former largely prevalent (*cis*/*trans* ratio ≈ 6). On the other hand, **3c**,**e**,**f** were isolated as mixtures of *cis*-*E* and *cis*-*Z* isomers. Interestingly, the solid-state IR spectrum of **3d** shows two pairs of bands for terminal and bridging carbonyls, at 1974/1952 cm^–1^ and 1806/1794 cm^–1^, respectively, whereas **3c**,**e**,**f** display two absorptions at lower wavenumbers (1940–1947 and 1756–1776 cm^–1^, respectively). On the other hand, the IR profiles of the carbonyl bands of **3c**–**f** are superimposable in CH_2_Cl_2_ (Figure S96).

The X-ray structure of *cis*-**3d** was ascertained by X-ray diffraction (Figure 12). Its bonding parameters are comparable to those reported for analogous complexes,^23,74^ with a marked asymmetry of the μ-CO ligand [Fe1–C2 1.942(4) Å, Fe2–C2 1.882(4) Å] and a slight asymmetry of the μ-aminocarbyne [Fe1–C2 1.877(4) Å, Fe2–C2 1.855(4) Å].

Next, a procedure was developed to trigger the degradation process of chlorido complexes **3** in aqueous medium, with assistance of an organic cosolvent due to their insolubility in water. Specifically, a stoichiometric amount of silver triflate was added to water/acetone solutions of **3c**–**f** at room temperature. The reaction was faster than the decarbonylation of [**1**]^+^ in aqueous media at 37 °C, as appreciated by the progressive formation of AgCl. However, in most cases a trace of **3** was still present after 20 h (IR analyses). Following volatiles removal under vacuum, the residue was treated with Me_2_SO_2_ (internal standard) and analyzed by ^1^H NMR (D_2_O). In each case, the corresponding secondary ammonium ion (RR′NH_2_^+^) was identified by comparison with authentic samples (Table S22). The formation of xylyl­(methyl)­amine from **3e** was confirmed by GC-MS. A similar procedure carried out with [Fe_2_Cp_2_Cl­(CO)­(μ-CO)­{μ-CNMe_2_}], **3a**, and Ag­(CF_3_SO_3_) in a biphasic medium (H_2_O/CDCl_3_) allowed to identify cyclopentadiene. Despite the near-quantitative conversion of **3**, ^1^H NMR yields of the amines represent 18–33% of the starting diiron compound. One test carried out by acidifying (HCl) the final mixture indicated that a small portion of the amines could have been lost during the vacuum treatment (yield of Anis­(Me)­NH_2_^+^ increased from 18% to 27%). Nevertheless, these figures resemble those found for the tricarbonyl complexes [**1**]^+^ in aqueous media. Both systems indicate that cleavage of the carbyne-nitrogen bond only accounts for a fraction of the decomposed aminocarbyne complex.

**Scheme 3:**
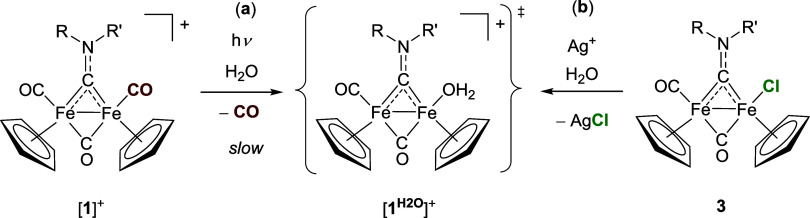
Formation of the Reactive Diiron­(I) Bis-cyclopentadienyl Aqua-Complex [**1** ^**H_2_O**^]^+^ by Photoactivated CO Dissociation from the Tricarbonyl Precursor [**1**]^+^ (Path **a**) or by Silver­(I)-Promoted Chloride Dissociation from the Dicarbonyl Complex **3** (Path **b**)­^a^

a Isomerism is not depicted.

**Scheme 4:**
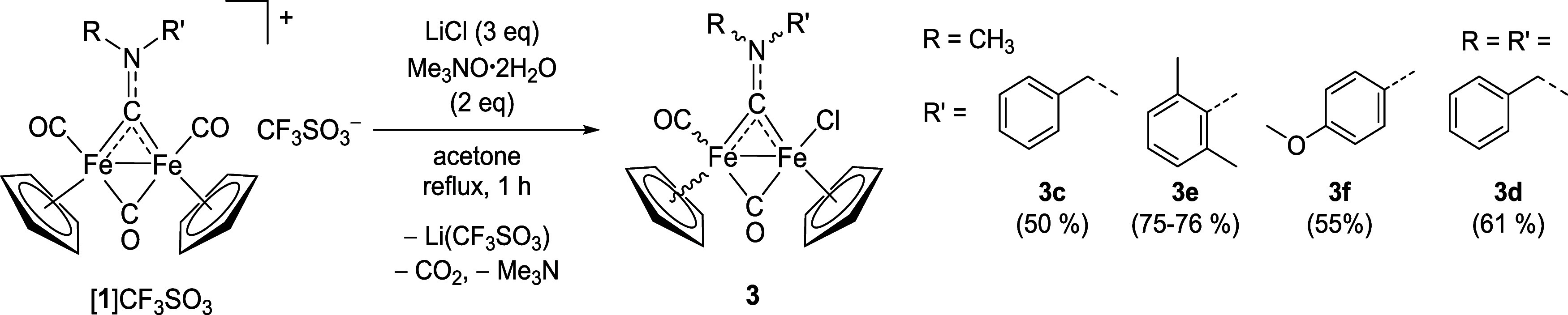
One-Pot Preparation of Chlorido-Dicarbonyl Compounds **3** from the Respective Tricarbonyl Precursors [**1**]­CF_3_SO_3_, LiCl, and Me_3_NO in Refluxing Acetone; Isolated Yields in Parentheses^a^

a Wavy bonds represent *cis*/*trans* or *E*/*Z* isomerism.

**Figure 12:**
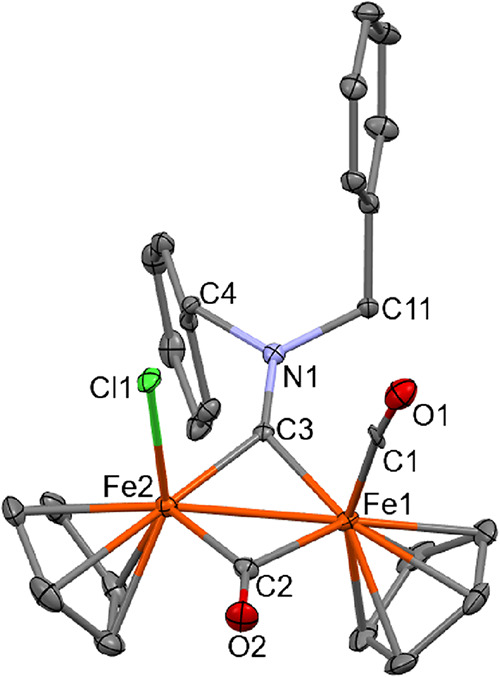
View of the structure of **3d**. Displacement ellipsoids are at the 30% probability level. H-atoms have been omitted for clarity. Main bond distances (Å) and angles (°): Fe1–Fe2 2.5063(8), Fe1–C1 1.847(5), Fe2–Cl1 2.3370(11), Fe1–C2 1.942(4), Fe2–C2 1.882(4), Fe1–C3 1.877(4), Fe2–C3 1.855(4), C1–O1 1.041(6), C2–O2 1.170(6), N1–C3 1.299(5), N1–C4 1.476(5), N1–C11 1.475(5), Fe1–C1–O1 173.4(4), Fe1–C2–Fe2 81.88(17), Fe1–C3–Fe2 84.38(16), C3–N1–C4 122.0(3), C3–N1–C11 124.1(3), C4–N1–C11 113.8(3).

## 3 Conclusions

Diiron­(I) bis-cyclopentadienyl aminocarbyne complexes represent an emerging class of anticancer agents that benefit from a considerable inertness in aqueous media. The biological activity of these prodrugs relies on an intracellular disassembly and release of reactive iron species. In this respect, investigating the slow degradation process occurring in aqueous media is fundamental to rationalize their behavior in a physiological setting. The process begins with a photoactivated, rate-determining dissociation of one CO ligand, followed by rapid evolution through multiple pathways. The major one involves interaction with dioxygen and leads to complete disruption of the coordination sphere, yielding the corresponding secondary amine, cyclopentadiene and iron­(III) oxyhydroxides. The process is strongly concentration-dependent and follows zero order kinetics above the millimolar range. Specifically, the extent of decomposition after a given time (*e.g.*, 72 h) is inversely related to the initial concentration of the compounds with an exponential trend. The organodiiron complexes are almost totally unaffected under anaerobic conditions or in the dark, even in very dilute solutions (*ca.* 10^–4^ mol/L). Hence, stock solutions of the aminocarbyne prodrugs should be stored as such. Phosphate buffers should be avoided as they promote the precipitation of FePO_4_. Within the investigated concentration range, increasing the temperature up to 70 °C or varying the pH in the 2–8 interval has a limited impact on the decomposition process, which is instead accelerated in basic solutions (pH ≥ 10). Trimethylamine *N*-oxide and hydrogen peroxide can also promote disruption of the diiron compounds, even in the absence of oxygen or light. A slight acceleration is observed in DMEM relative to water, without evidence of direct involvement of its components in the formation of new soluble or insoluble species (*e.g.*, chlorocomplexes via CO/Cl^–^ exchange). As in water, the resulting precipitate mainly consists of iron­(III) oxyhydroxides together with some carbonyl/isocyanide species derived from complexes with an aromatic group in the aminocarbyne ligand. Save for this aspect, the reactivity of different complexes is comparable under identical conditions and the counteranion (CF_3_SO_3_^–^ or NO_3_^–^) takes no part in it.

In summary, this work underscores fundamental aspects of the reactivity of a class of iron-based anticancer agents in aqueous solution and shed light on a complex activation process. Further studies will focus on identifying the wavelength(s) responsible for photoactivation and extending the analysis to a more biologically relevant concentration range. As a matter of fact, the present kinetic and speciation data provide an important model framework, but extrapolation to low-micromolar conditions should be made with care. Beyond the specific system investigated, this study establishes a general framework for the analysis of organometallic reactivity in aqueous and biological environments, where slow, multipathway transformations, concentration effects, and heterogeneous conditions are often overlooked.

## 4 Experimental Section

### 4.1 General Experimental Details

#### 4.1.1 Materials and Methods

Except where otherwise noted, reactants and solvents were obtained from Merck or TCI Chemicals and were used as received. LiCl and 1,3,5-triaza-7-phosphaadamantane (PTA) were stored under N_2_. Isocyanides were stored at −20 °C (*m*-xylyl isocyanide, benzyl isocyanide and 4-methoxyphenyl isocyanide) or at 4 °C (cyclohexyl isocyanide, CyNC); contaminated labware was treated with HCl/EtOH. The concentration of H_2_O_2_ (9.31 mol/L) was determined by ^1^H NMR (*d*1 = 30 s, 8 scans) on a THF-*d*_8_ solution prepared with the concentrated aqueous solution (20 μL) and Me_2_SO_2_ (20 mg) as internal standard (δ/ppm = 9.75 for H_2_O_2_, 3.32 for Me_2_SO_2_, 2.90 for H_2_O).^75^

All synthetic work was carried out under a N_2_ atmosphere using standard Schlenk techniques. Reactions were monitored by IR spectroscopy on aliquots of the reaction mixture. Compounds [**1a**–**f**]­CF_3_SO_3_,^23,36^ [**1a**]­NO_3_ ^24^ and **3a** ^76^ were prepared according to the literature. Acetonitrile used for the preparation of [**1b**–**e**]­NO_3_ was distilled from CaH_2_ and stored over 3 Å MS. Acetone used for the preparation of [**2c**]­CF_3_SO_3_ and **3c**–**f** was deaerated by bubbling Ar for 30′. All the other operations were carried out in air with common laboratory glassware. Chromatographic separations were carried out on neutral alumina columns (Merck). All isolated compounds are air- and moisture-stable in the solid state.

#### 4.1.2 Instruments and Measurements

NMR spectra were recorded at ambient temperature on Jeol JNM-ECZR 400 and 500 MHz instruments equipped with Royal HFX Broadband probes. Chemical shifts (ppm) are referenced to the residual solvent peaks (^1^H, ^13^C) or to external standards (^19^F to CFCl_3_, ^31^P to 85% H_3_PO_4_).^77^ ^1^H and ^13^C NMR spectra were assigned with the support ^1^H–^13^C *gs*-HSQC and/or HMBC experiments. Signals corresponding to the minor isomer—if present—are *italicized*; the cyclopentadienyl ligand bonded to the Fe­(PTA) fragment is labeled as Cp^P^. CDCl_3_ used for NMR analysis was filtered on alumina and stored in the dark over Na_2_CO_3_. D_2_O was stored under N_2_ at 4 °C. IR spectra of solid samples (650–4000 cm^–1^) were recorded on an Agilent Cary 630 instrument equipped with a UATR sampling accessory (ZnSe crystal). IR spectra of solutions were recorded using a CaF_2_ liquid transmission cell (1500–2300 cm^–1^) on a PerkinElmer Spectrum 100 FT-IR spectrometer. IR bands and NMR signals due to terminal or bridging carbonyls are denoted as t-CO and μ-CO, respectively. UV–vis spectra (200–800 nm) were recorded on a Ultraspec 2100 Pro spectrophotometer using quartz cuvettes (0.2, 0.5, or 1.0 cm path length). IR and UV–vis spectra were processed with Spectragryph.^78^ CHNS analyses were performed on a Vario MICRO cube instrument (Elementar); the reported data is the average of two independent measurements. Conductivity measurements were carried using an XS COND 8 instrument (cell constant = 1.0 cm^–1^) equipped with NT 55 temperature probe (measurements automatically adjusted to 25 °C) and was calibrated using standard KCl solutions in ultrapure water.^49^ pH measurements were performed with an Orion pH meter equipped with a Hamilton glass pH electrode, calibrated with pH = 4.0 and pH = 7.0 buffer solutions.

ICP-OES analyses were carried out with Optima 8000 ICP-OES (PerkinElmer) operating at 1500 W and equipped with autosampler S10, MiraMist Nebulizer (PerkinElmer) and cyclonic chamber. Argon (420.069 nm) was used as the internal standard. Fe was examined at 238.204 nm wavelength.

Iron content was determined by comparison with calibration curves obtained by dilution in 2% HNO_3_ of a commercial standard solution (1000 mg/L, Fluka TraceCERT).

Raman analyses were conducted with a Renishaw Invia micro-Raman instrument equipped with a multichannel air-cooled CCD detector, a Nd:YAG laser working at 532 nm and an Olympus 50× objective. Spectra of solids were acquired at 0.1 mW: keeping a low laser power is mandatory when working with iron compounds to avoid any laser-induced thermal effect on samples.^79^ The spectral resolution was 5 cm^–1^ and the spectral range analyzed between 100 and 1000 cm^–1^. In order to improve the signal-to-noise ratio, the data were accumulated over 10 acquisitions with an exposure time of 10 s per acquisition. There are no significant discrepancies between the Raman band positions reported in the literature for α-Fe_2_O_3_ ^79^ and those found analyzing our reference compound (Figure S54). The Raman spectrum of the aqueous solution of [**1a**]­NO_3_ (Figure S50) was recorded using a 96-well plate well as the sample holder, focusing the laser into the solution with a 20× objective. The instrumental parameters were as follows: 633 nm laser, 14 mW power, and 10 accumulations of 10 s each.

ESI-Q/ToF flow injection analyses were carried out using a 1200 Infinity HPLC coupled to a Jet Stream ESI interface with a Quadrupole-Time of Flight tandem mass spectrometer 6530 Infinity Q-TOF (Agilent Technologies). Ca. 1 mg of sample was weighed and dissolved in 1 mL MeOH (LC-MS grade, Carlo Erba) and then diluted to 10 ppm. Injection volume: 0.1 μL. The flow rate was 0.2 mL/min (total run time 3 min). The ESI operating conditions were: *drying gas* (N_2_, purity >98%): 350 °C and 10 L/min; *capillary voltage* 4.5 kV; nozzle voltage: 1 kV; *nebulizer gas* 35 psig; *sheath gas* (N_2_, purity >98%): 375 °C and 11 L/min. The fragmentor was kept at 50 V, the skimmer at 65 V and the OCT 1 RF at 750 V. High resolution MS spectra were achieved in positive mode in the range 100–1700 *m*/*z*; the mass axis was calibrated daily using the Agilent tuning mix HP0321 prepared in acetonitrile and water.

### 4.2 Synthesis and Characterization of Compounds

#### 4.2.1 [Fe_2_Cp_2_(CO)_2_(μ-CO)­{μ-CNR­(R′)}]­NO_3_, [1]­NO_3_

##### 4.2.1.1 General Procedure

In a Schlenk flask under N_2_, a suspension of [Fe_2_Cp_2_(CO)_4_] in MeCN (1.0–1.4 g in 25–30 mL or 5.6 g in 75 mL) was treated with the selected isocyanide (CNR) and was stirred under reflux for 3–3.5 h (R = cyclohexyl, benzyl) or at room temperature for 24 h (R = xylyl). For [**1b**,**c**,**e**]^+^: the mixture was treated with MeI and stirred under reflux overnight (*ca.*, 17 h) [the tap of the vessel was closed to avoid loss of alkylating agent]. For [**1d**]^+^: the mixture was treated with BnBr and stirred under reflux for 2.5 h. The resulting dark red solution ([**1b**,**c**,**e**]^+^) or suspension ([**1d**]^+^) was allowed to cool to room temperature and treated with AgNO_3_, with immediate precipitation of a pale-yellow solid (AgBr or AgI). The suspension was stirred at room temperature for 1–3 h under protection from the light, then filtered over a Celite pad. The filtrate solution was taken to dryness under vacuum. The residue was dissolved in CH_2_Cl_2_ and moved on top of an alumina column (1 g scale: h 4–5 cm, d 4.3 cm; 6 g scale: h 8 cm, d 7 cm). A dark brown band containing [FeCpX­(CO)_2_] (X = Br, I; IR (CH_2_Cl_2_): ṽ/cm^–1^ = 2039s, 1995s for X = I^80^ was eluted with CH_2_Cl_2_, other impurities were eluted as yellow/orange/brown bands using THF and then MeCN. A red band containing the title compound started trailing with MeCN and its elution was completed with MeCN/MeOH 10:1 *V*/*V*. The eluate was taken to dryness under vacuum, redissolved in CH_2_Cl_2_ and filtered over a Celite pad. The filtrate was evaporated under vacuum affording a foamy red solid. The solid was suspended in Et_2_O/EtOAc (2:1 *V*/*V* for [**1b**]^+^, 1:1 *V*/*V* for [**1c**]^+^) or Et_2_O/toluene (4:1 *V*/*V* for [**1d**]^+^, 1:1 *V*/*V* for [**1e**]^+^) under stirring. The suspension was filtered and the resulting red solid was washed with Et_2_O, hexane and dried under vacuum. All compounds were kept under N_2_ for long time storage as a precaution.

##### 4.2.1.2 Notes

Reactions carried out with MeI at room temperature led to negligible conversion. On the other hand, a large excess of MeI (20 equiv) under reflux drastically lowered the yield of [**1e**]­NO_3_ (*ca.*, 54%) with elution of additional bands containing piano-stool Fe­(II) compounds. However, most of [FeCpI­(CO)_2_] probably results from the oxidative cleavage of unreacted [Fe_2_Cp_2_(CO)_4_] from the first step.^81^ The absence of halides in the final product was qualitatively checked with AgNO_3_ in MeOH and corroborated by CHN analyses. Compounds [**1**]­NO_3_ were isolated as single isomers with *cis* stereochemistry; *trans* isomers, if present, are found in trace amounts (<1%).

#### 4.2.2 [Fe_2_Cp_2_(CO)_2_(μ-CO)­{μ-CNMe­(Cy)}]­NO_3_, [1b]­NO_3_ (Chart 1)

Prepared according to the general procedure using [Fe_2_Cp_2_(CO)_4_] (1.393 g, 3.933 mmol, 1.5 equiv), cyclohexyl isocyanide (0.33 mL, 2.65 mmol; 1 equiv), MeI (2.0 mL, 32 mmol, 12 equiv) and AgNO_3_ (595 mg, 3.50 mmol, 1.3 equiv). Yield: 1.088 g, 80% vs. isocyanide. Soluble in H_2_O, CH_2_Cl_2_, CHCl_3_, MeOH, poorly soluble in EtOAc, insoluble in toluene, Et_2_O, hexane. X-ray quality crystals of [**1b**]­NO_3_·CH_2_Cl_2_ were obtained from a CH_2_Cl_2_ solution layered with Et_2_O and settled aside at −20 °C. Anal. Calcd for C_21_H_24_Fe_2_N_2_O_6_: C, 49.25; H, 4.72; N, 5.47. Found: C, 47.84; H, 4.88; N, 5.69. IR (solid state): ṽ/cm^–1^ = 3080w, 2930w, 2855w, 2138w; 1999s, 1972s (t-CO); 1812s (μ-CO); 1560s (μ-CN); 1541m, 1507w, 1449w, 1399m-sh, 1330s-br (NO_3_), 1319s, 1298s, 1248m-sh, 1170w, 1153w, 1052m, 1014w, 990w, 851m-br, 795m, 745s. IR (CH_2_Cl_2_): ṽ/cm^–1^ = 2019s, 1987w-sh (t-CO); 1834m (μ-CO); 1605w, 1568w (μ-CN), 1554w, 1451w, 1350m-br (NO_3_). ^1^H NMR (CDCl_3_): δ/ppm = 5.42 (s, 5H, Cp), 5.31 (s, 5H, Cp’), 4.73–4.62 (m, 1H, NCH^Cy^), 4.10 (s, 3H, NCH_3_); 2.86 (d, *J* = 11 Hz, 1H), 2.15 (d, *J* = 13 Hz, 1H), 2.06–1.95 (m, 2H), 1.89–1.74 (m, 3H), 1.54–1.37 (m, 2H), 1.36–1.20 (m, 1H) (CH_2_^Cy^). ^1^H NMR (acetone-d_6_): δ/ppm = 5.59 (s, 5H, Cp), 5.53 (s, 5H, Cp’), 4.91 (tt, ^3^ *J*_HH_ = 12.1, 3.5 Hz, 1H, NCH^Cy^), 4.19 (s, 3H, NCH_3_); 2.61 (d, *J* = 12.4 Hz, 1H), 2.13 (ddd, *J* = 25.5, 12.9, 3.9 Hz, 1H), 2.05–1.98 (m, 1H), 1.93–1.87 (m, 1H), 1.83–1.55 (m, 5H), 1.38–1.27 (m, 1H) (CH_2_^Cy^). ^13^C­{^1^H} NMR (acetone-d_6_): δ/ppm = 317.3 (μ-CN), 256.7 (μ-CO); 210.3, 209.6 (t-CO); 91.5, 91.3 (Cp); 79.7 (NCH^Cy^); 47.3 (NCH_3_), 31.4, 31.2, 26.1, 25.7 (CH_2_^Cy^).

**Chart 1:**
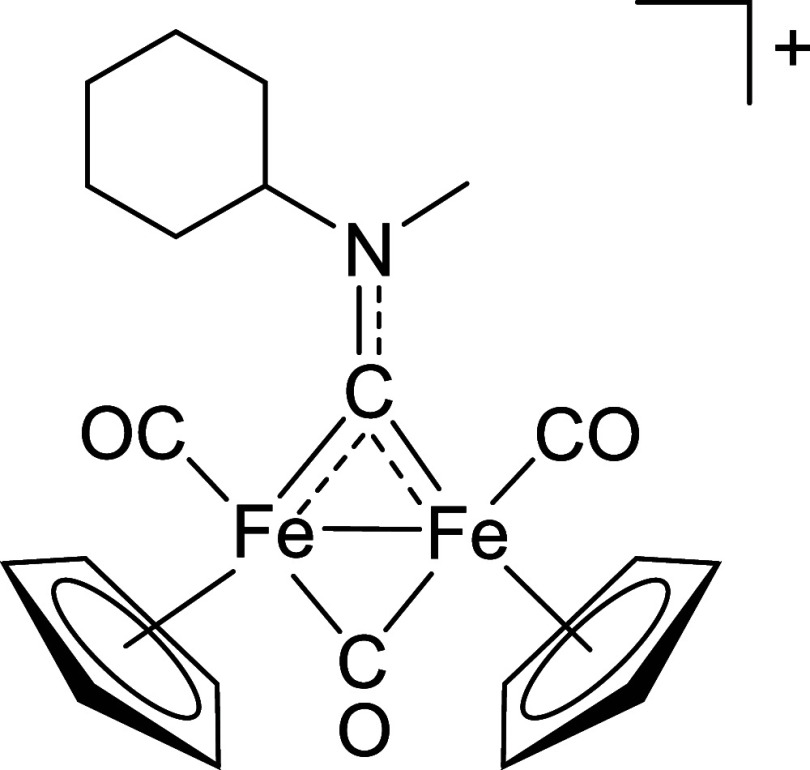
Structure of [**1b**]^+^

#### 4.2.3 [Fe_2_Cp_2_(CO)_2_(μ-CO)­{μ-CNMe­(Bn)}]­NO_3_, [1c]­NO_3_ (Chart 2)

Prepared according to the general procedure using [Fe_2_Cp_2_(CO)_4_] (1.00 g, 2.825 mmol, 1.1 equiv), benzyl isocyanide (0.315 mL, 2.59 mmol, 1.0 equiv), MeI (1.25 mL, 20.1 mmol, 7.76 equiv) and AgNO_3_ (525 mg, 3.11 mmol, 1.2 equiv). Yield: 858 mg, 1.65 mmol, 63% vs. isocyanide. The residence time of [**1c**]^+^ on the alumina column must be minimized, otherwise dark brown byproducts begin to accumulate on the front of the eluting red band. Soluble in H_2_O, MeOH, CH_2_Cl_2_, less soluble in acetone, THF, ^i^PrOH, poorly soluble in EtOAc, Et_2_O, insoluble in toluene, hexane. Anal. Calcd for C_22_H_20_Fe_2_N_2_O_6_: C, 50.81; H, 3.88; N, 5.39. Found: C, 50.95; H, 3.74; N, 5.20. IR (solid state): ṽ/cm^–1^ = 3082w, 3062w; 1994s, 1969s, 1959s (t-CO); 1821s (μ-CO); 1585w-sh, 1566m, 1561m (μ-CN), 1542w-sh, 1508w, 1490w, 1447w, 1419w, 1391w-sh, 1364m-sh, 1333s-br (NO_3_), 1222w, 1209w, 1159w, 1081w, 1053w, 1013w, 1004w, 946w, 858m, 829m, 756s, 746m-sh, 701s. IR (CH_2_Cl_2_): ṽ/cm^–1^ = 2020s, 1988m (t-CO); 1835m (μ-CO), 1590w, 1577w (μ-CN), 1397w, 1351m-br (NO_3_). ^1^H NMR (acetone-*d*_6_): δ/ppm = 7.52–7.48 (m, 4H), 7.47–7.41 (m, 1H) (Ph); 6.01, 5.91 (d, ^2^ *J*_HH_ = 14.8 Hz, 1H+1H, NCH_2_), 5.65 (s, 5H, Cp), 5.61 (s, 5H, Cp’), 4.16 (s, 3H, NCH_3_). ^13^C­{^1^H} NMR (acetone-d_6_): δ/ppm = 321.0 (μ-CN), 256.3 (μ-CO); 210.2, 209.6 (t-CO); 135.2 (Ph/C_ipso_), 130.2 (Ph/C_ortho_), 129.5 (Ph/C_para_), 128.8 (Ph/C_meta_); 91.6, 91.5 (Cp); 72.3 (NCH_3_), 51.8 (NCH_2_).

**Chart 2:**
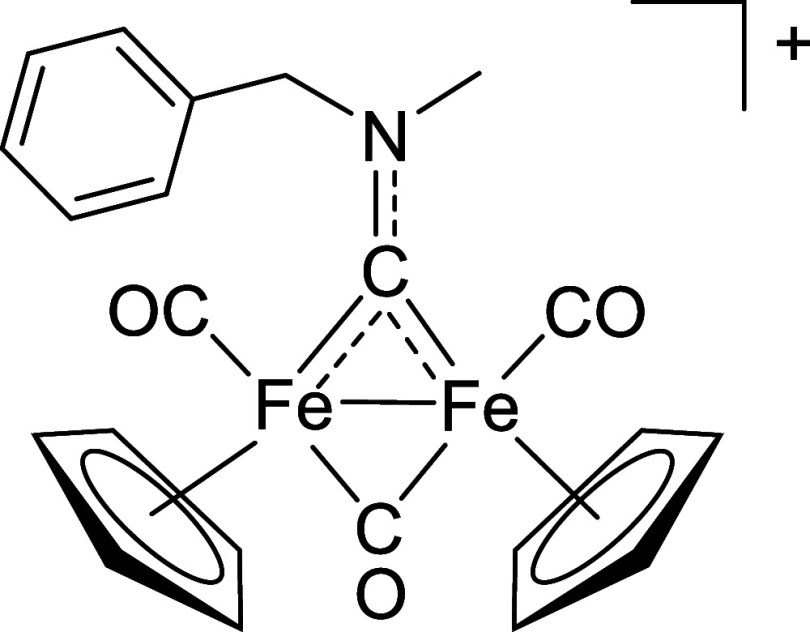
Structure of [**1c**]^+^

#### 4.2.4 [Fe_2_Cp_2_(CO)_2_(μ-CO)­(μ-CNBn_2_)]­NO_3_, [1d]­NO_3_ (Chart 3)

Prepared according to the general procedure using [Fe_2_Cp_2_(CO)_4_] (1.121 g, 3.167 mmol, 1.1 equiv), benzyl isocyanide (0.35 mL, 2.87 mmol, 1 equiv), benzyl bromide (1.90 mL, 16 mmol, 5.56 equiv) and AgNO_3_ (658 mg, 3.87 mmol, 1.35 equiv). Yield: 1.306 g, 2.19 mmol, 76% vs. isocyanide. Soluble in acetone, MeOH, H_2_O, poorly soluble in toluene, insoluble in Et_2_O, hexane. Anal. Calcd for C_28_H_24_Fe_2_N_2_O_6_: C, 56.41; H, 4.06; N, 4.70. Found: C, 55.51; H, 3.83; N, 4.65. IR (solid state): ṽ/cm^–1^ = 3067w, 3026w, 2151w; 2002s, 1973s (t-CO); 1813s (μ-CO); 1583w, 1541m, 1534m, 1527m (μ-CN), 1522m, 1507m, 1496w, 1491w, 1451m, 1430m, 1419m, 1363m-sh, 1331s-br (NO_3_), 1241m, 1214m, 1157w, 1093w, 1076w, 1027w, 1000w, 854m, 827m, 763s, 734m, 695s. IR (CH_2_Cl_2_): ṽ/cm^–1^ = 2021s, 1990m (t-CO); 1838m (μ-CO); 1605w, 1589w, 1550w, 1535w (μ-CN), 1455w, 1433w, 1421w, 1351m-br (NO_3_). ^1^H NMR (CDCl_3_): δ/ppm = 7.49–7.43 (m, 4H, Ph/C_meta_), 7.42–7.37 (m, 2H, Ph/C_para_), 7.22 (d, ^3^ *J*_HH_ = 7.3 Hz, 4H, Ph/C_ortho_), 5.69 (d, ^2^ *J*_HH_ = 14.8 Hz, 2H, NCH), 5.52 (d, ^2^ *J*_HH_ = 14.9 Hz, 2H, NCH’), 5.44 (s, 10H, Cp). ^1^H NMR (acetone-d_6_): δ/ppm = 7.49 (t, ^3^ *J*_HH_ = 7.4 Hz, 4H, Ph/C_meta_), 7.42 (t, ^3^ *J*_HH_ = 7.3 Hz, 2H, Ph/C_para_), 7.37 (d, ^3^ *J*_HH_ = 7.4 Hz, 4H, Ph/C_ortho_), 5.88 (d, ^2^ *J*_HH_ = 15.3 Hz, 2H, NCH), 5.70 (s, 10H, Cp), 5.68 (d, ^2^ *J*_HH_ = 15.4 Hz, 2H, NCH’). ^13^C­{^1^H} NMR (acetone-d_6_): δ/ppm = 325.9 (μ-CN), 255.5 (μ-CO), 210.0 (t-CO), 134.4 (Ph/C_ipso_), 130.3 (Ph/C_ortho_), 129.4 (Ph/C_para_), 128.6 (Ph/C_meta_), 91.9 (Cp), 69.3 (NCH_2_).

**Chart 3:**
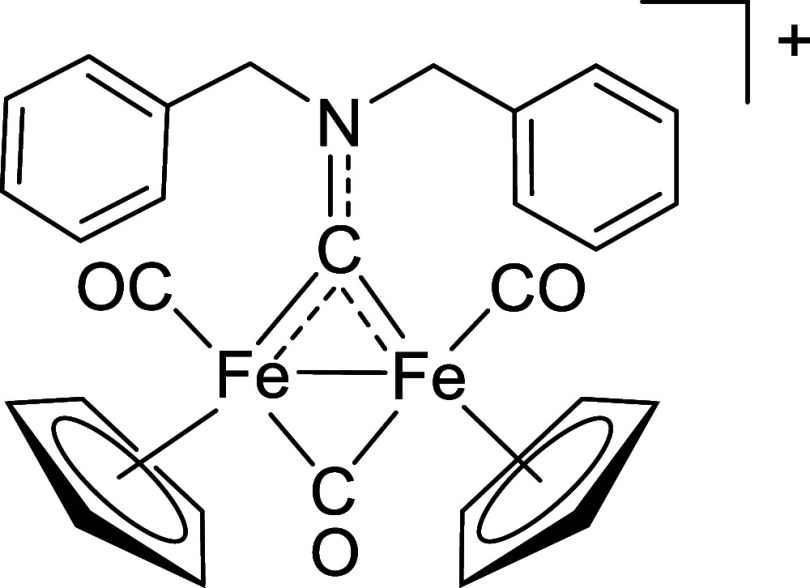
Structure of [**1d**]^+^

#### 4.2.5 [Fe_2_Cp_2_(CO)_2_(μ-CO)­{μ-CNMe­(Xyl)}]­NO_3_, [1e]­NO_3_ (Chart 4)

Prepared according to the general procedure, using [Fe_2_Cp_2_(CO)_4_] (5.640 g, 15.94 mmol, 1.6 equiv), xylyl isocyanide (1.306 g, 9.96 mmol, 1 equiv), methyl iodide (7.4 mL, 116 mmol, 12 equiv) and AgNO_3_ (2.538 g, 14.94 mmol, 1.5 equiv). Yield: 4.124 g, 7.72 mmol, 79% vs. isocyanide. Soluble in CH_2_Cl_2_, CHCl_3_, acetone, CH_3_CN, H_2_O, insoluble in Et_2_O, hexane. X-ray quality crystals of [**1e**]­NO_3_ were collected from a CH_2_Cl_2_ solution layered with hexane and settled aside at −20 °C. Anal. Calcd for C_23_H_22_Fe_2_N_2_O_6_: C, 51.72; H, 4.15; N, 5.24. Found: C, 51.9; H, 4.13; N 5.20. IR (solid state): ṽ/cm^–1^ = 3081w; 2009s, 1968s (t-CO); 1825s (μ-CO); 1543m, 1526m (μ-CN), 1471w, 1418w, 1383m-sh, 1365m-sh, 1332s-br (NO_3_), 1206w, 1111w, 1084w, 1006m, 887w, 856w, 848m, 829w, 851w, 784m, 767s, 731s, 665s. IR (CH_2_Cl_2_): ṽ/cm^–1^ = 2022s, 1990w (t-CO); 1839m (μ-CO), 1547w, 1530w (μ-CN), 1422w, 1349m-br (NO_3_). ^1^H NMR (CDCl_3_): δ/ppm = 7.35 (m-br), 7.18–7.08 (m) (3H, C_6_H_3_); 5.59 (s-br, 5H, Cp), 4.90 (s-br, 5H, Cp’), 4.51 (s-br, 3H, NCH_3_); 2.83 (s-br, 3H), 2.11 (s, 3H) (CCH_3_). The bright red CDCl_3_ solution turned dark red-brown after 15 h at room temperature, showing partial formation of **2e** (IR analysis). ^1^H NMR (acetone-*d*_6_): δ/ppm = 7.50–7.44 (m, 2H), 7.44–7.37 (m, 1H) (C_6_H_3_); 5.75 (s, 5H, Cp), 5.08 (s, 5H, Cp’), 4.63 (s, 3H, NCH_3_); 2.78 (s, 3H), 2.20 (s, 3H) (CCH_3_). ^1^H NMR (CD_3_CN): δ/ppm = 7.48–7.31 (m, 3H, C_6_H_3_); 5.45 (s, 5H, Cp), 4.82 (s, 5H, Cp’), 4.40 (s, 3H, NCH_3_); 2.64 (s, 3H), 2.13 (s, 3H) (CCH_3_). ^13^C­{^1^H} NMR (CD_3_CN): δ/ppm = 327.8 (μ-CN), 255.1 (μ-CO); 209.7, 209.2 (t-CO); 149.1 (Xyl/C_ipso_), 134.1, 133.2 (Xyl/C_meta_); 131.2; 130.1 (s-br; Xyl/C_ortho_); 130.44, 130.41 (s-br; Xyl/C_para_); 91.6 (Cp); 90.9 (m, Cp’); 56.4 (NCH_3_), 18.9 (m, CCH_3_), 17.9 (CCH_3_).

**Chart 4:**
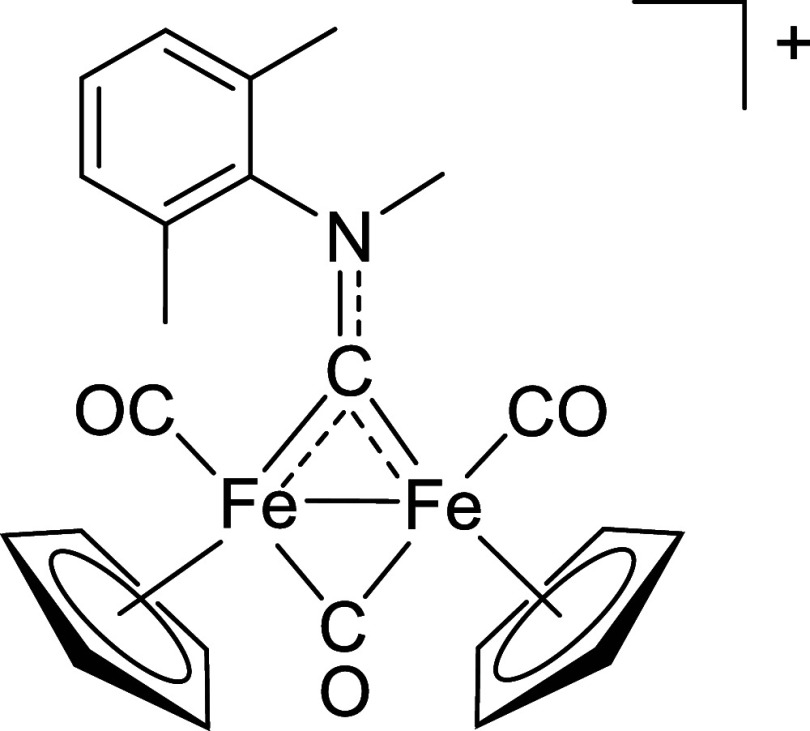
Structure of [**1e**]^+^

#### 4.2.6 [Fe_2_Cp_2_(CO)­(PTA)­(μ-CO)­{μ-CNMe­(Bn)}]­CF_3_SO_3_, [2c]­CF_3_SO_3_ (Chart 5)

Compound [**1c**]­CF_3_SO_3_ (86 mg, 0.14 mmol), Me_3_NO·2H_2_O (20 mg, 0.18 mmol) and MeCN (5 mL) were introduced into a 25 mL Schlenk flask under N_2_. The mixture was stirred at room temperature for 1 h and the complete conversion of [**1c**]^+^ was checked by IR (CH_2_Cl_2_). Next, volatiles were removed under vacuum and the brown residue was treated with PTA (22 mg, 0.14 mmol, 1.0 equiv) and acetone (10 mL). The mixture was stirred under reflux for 3.5 h, affording a dark green-brown solution. The conversion was checked by IR (MeCN). Volatiles were removed under vacuum. The residue was dissolved in few mL of CH_2_Cl_2_ and moved on top of an alumina column (d 2.3 cm, h 5 cm). Impurities were eluted with CH_2_Cl_2_, THF (pale yellow band) and then MeCN (pale orange-brown band). A dark green band was eluted with MeCN/MeOH 25:1 *V*/*V* and then taken to dryness under vacuum. The residue was redissolved in CH_2_Cl_2_, the solution was filtered over a Celite pad and volatiles were removed under vacuum. The dark green-brown solid was triturated in a Et_2_O/hexane 1:1 *V*/*V* mixture. The suspension was filtered, the solid was washed with Et_2_O/hexane 1:1 *V*/*V*, followed by hexane and then dried under vacuum. Yield: 83 mg, 80% of a mixture of *cis*/*trans* and *E*/*Z* isomers (*cis*-*E*/*cis*-*Z* ratio = 1.3; *cis*/*trans* ratio ≈ 10; ^1^H NMR). A dark green-brown solid consisting of *cis*-*E*/*Z* isomers only (*cis*-*E*/*cis*-*Z* ratio = 1.3) was recovered from a green band slowly eluted with neat MeCN on a subsequent alumina column. Yield: 35 mg, 30%. Soluble in CH_2_Cl_2_, acetone, MeOH, MeCN; poorly soluble in EtOAc, insoluble in toluene, Et_2_O, hexane. X-ray quality crystals of *cis*-*E*-[**2c**]­CF_3_SO_3_ were obtained from an acetone solution layered with Et_2_O and settled aside at −20 °C. Anal. Calcd for C_28_H_32_F_3_Fe_2_N_4_O_5_PS: C, 45.67; H, 4.38; N, 7.61; S, 4.35. Found: C, 44.63; H, 4.42; N, 7.38; S, 4.12. IR (solid state): ṽ/cm^–1^ = 3093w, 3070w, 3036w, 2920w, 2873w, 1947s (t-CO), 1781s (μ-CO), 1545m (μ-CN), 1526m-sh, 1495w, 1447m, 1421m, 1391m, 1352w; 1269s-sh, 1257s, 1243s, 1222m-sh (SO_3_); 1149m, 1101m, 1078m-br, 1045w, 1028s, 1016s, 971s, 948s, 892w, 848m, 806m, 762s, 743s, 698m. IR (CH_2_Cl_2_): ṽ/cm^–1^ = 1968s (t-CO), 1802s (μ-CO), 1550m (μ-CN), 1530w-sh, 1454w, 1444w, 1421w, 1393w. IR (MeCN): ṽ/cm^–1^ = 1968s (t-CO), 1801s (μ-CO), 1552m (μ-CN); no significant differences were observed between the various isomeric compositions. ^1^H NMR (acetone-d_6_): δ/ppm for *cis-E*/ *cis-Z* isomers = 7.76–7.43 (m, 5H, Ph); *6*. *08*, 6.02 (d, ^2^ *J*_HH_ = 15 Hz, 1H, CHPh); 5.81, *5*. *78* (d, ^2^ *J*_HH_ = 15 Hz, CH’Ph); *5*. *40*, 5.29 (s, 5H, Cp); 5.23, *5*. *13* (d, ^3^ *J*_HP_ = 1.5 Hz, 5H, Cp^P^); 4.46–4.28 (m, 6H, NCH_2_); 4.19, *4*. *15* (s, 3H, NCH_3_); 4.02–3.88 (m), 3.91 (s) (6H, PCH_2_); δ/ppm for for *trans-E*/ *trans*-Z isomers = 6.16, 5.76 (d, ^2^ *J*_HH_ = 14 Hz, 2H, NCH_2_Ph); *5*. *06*, 5.03 (d, ^3^ *J*_HP_ = 1.1 Hz, 5H, Cp^P^), 4.17 (s, 3H, NCH_3_). ^31^P­{^1^H} NMR (acetone-d_6_): δ/ppm = −16.4 (*trans*-*Z*), −18.2 (*trans*-*E*), −19.9 (*cis*-*Z*), −22.8 (*cis*-*E*). ^1^H NMR (CD_3_OD): δ/ppm for *cis*-*E*/*cis* - *Z* isomers = 7.63–7.59, 7.58–7.53, 7.52–7.44 (m, 5H, Ph); *5*. *90*, 5.88, 5.69, *5*. *61* (d, *J* = 15.0 Hz, 2H, NCH_2_Ph); *5*. *28*, 5.17 (s, 5H, Cp); 5.11, *4*. *99* (s, 5H, Cp^P^); 4.49–4.26 (m, 6H, NCH_2_N); 4.06 (s, 3H, NCH_3_); 3.90–3.77 (m, 6H, NCH_2_P); *E* → *Z* isomerization occurred at room temperature (*E*/*Z* ratio: 1.3 at 0 h and 0.55 at 24 h). ^13^C­{^1^H} NMR (CD_3_OD): δ/ppm for *cis*-*E*/*cis* - *Z* isomers = *329*. *2*, 328.8 (d, ^2^ *J*_CP_ = 16 Hz, μ-CN); 263.3, *262*. *4* (d, ^2^ *J*_CP_ = 18 Hz, μ-CO); 216.7, *216*. *3* (d, ^2^ *J*_CP_ = 3 Hz); 136.1, *135*. *8* (Ph/C_ipso_); *130*. *8*, 130.6 (Ph/C_ortho_); 129.9, *129*. *7* (Ph/C_para_); 129.0, *128*. *0* (Ph/C_meta_); 121.8 (d, *J* = 319 Hz, CF_3_), *90*. *5*, 90.4 (Cp), 88.4, *88*. *2* (Cp^P^); 72.8 (d, ^3^ *J*_CP_ = 6.7 Hz, NCH_2_N); 72.3, *72*. *2* (NCH_2_Ph), *54*. *4*, 54.3 (s, ^1^ *J*_CP_ = 13 Hz, PCH_2_); *52*. *3*, 52.0 (s, NCH_3_). ^31^P­{^1^H} NMR (CD_3_OD): δ/ppm = −18.3 (*cis*-*Z*), −21.1 (*cis*-*E*). ^1^H NMR (D_2_O): δ/ppm for *cis*-*E*/*cis* - *Z* isomers = 7.66–7.48 (m, 5H, Ph); 5.92–5.82 (m, 1H), 5.69, *5*. *61* (d, ^2^ *J*_HH_ = 15.0 Hz, 1H) (NCH_2_Ph); *5*. *31*, 5.19 (s, 5H, Cp); 5.09, *4*. *99* (d, ^3^ *J*_HP_ = 1.6 Hz, 5H, Cp^P^); 4.42–4.33, 4.31–4.23 (m, 6H, NCH_2_P); 4.07, *4*. *06* (s, 3H; NCH_3_); 3.84–3.69 (m), 3.75 (s) (6H, PCH_2_). ^31^P­{^1^H} NMR (D_2_O): δ/ppm = −*13*. *6* (*cis*-*Z*), −16.4 (*cis*-*E*).

**Chart 5:**
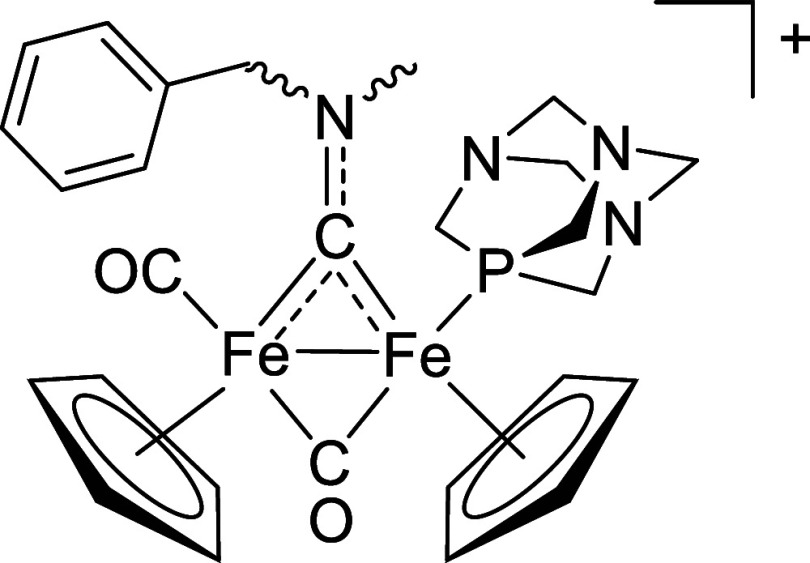
Structure of [**2c**]^+^ (Wavy Bonds Refer to *E*/*Z* Isomerism)

#### 4.2.7 [Fe_2_Cp_2_Cl­(CO)­(μ-CO)­{μ-CNR­(R′)}], 3c-f (Chart 6)

##### 4.2.7.1 General Procedure

In a 100 mL Schlenk tube under N_2_, a dark red suspension of the appropriate tricarbonyl precursor [Fe_2_Cp_2_(CO)_3_{μ-CNR­(R’)}]­CF_3_SO_3_, [**1**]­CF_3_SO_3_ (200–600 mg, 1.0 equiv), LiCl (3.0 equiv) and Me_3_NO·2H_2_O (2.0 equiv) in deaerated acetone (10–15 mL) was stirred at reflux for 1 h. The resulting brown suspension was allowed to cool to room temperature and then taken to dryness under vacuum. The residue was dissolved in a small volume of CH_2_Cl_2_ and moved on top of an alumina column (d 3.4, h 3 cm for 200–250 mg scale; d 4.3, h 5 cm for 300–600 mg scale). Impurities were eluted with CH_2_Cl_2_ then a brown band containing the title product was eluted with DCM/THF 1:1 *V*/*V* (**3d**) or THF (**3c**,**e**,**f**). Volatiles were removed under vacuum. The residue was triturated in a Et_2_O/petroleum ether 1:1 *V*/*V* (**3c**,**e**) or 1:2 *V*/*V* mixture (**3d**,**f**). The suspension was filtered and the resulting brown solid was washed with hexane and dried under vacuum. Compounds **3c**, **3e**, **3f** were previously prepared by two-step procedures (yields 61–73%)^62,73,74^ while **3d** is unprecedented.

**Chart 6:**
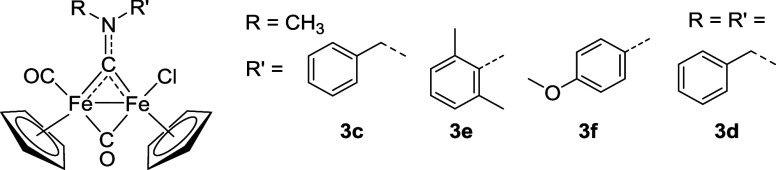
General Structure of **3c**–**f**

#### 4.2.8 [Fe_2_Cp_2_Cl­(CO)­(μ-CO)­{μ-CNMe­(Bn)}], 3c

Prepared from [**1c**]­CF_3_SO_3_ (250 mg, 0.412 mmol) according to the general procedure. Yield: 96 mg, 50%. IR (solid state): ṽ/cm^–1^ = 3102w, 3069w, 2923w, 2862w, 1946 (t-CO); 1766 (μ-CO); 1584w, 1559m, 1535m, 1497w, 1452w, 1431w, 1418w, 1392w, 1357w, 1346w, 1233w, 1169w, 1181w, 1081w, 1066w, 1011w, 1001w, 969w, 873w, 840m, 817w, 773s, 734m, 697s. IR (CH_2_Cl_2_): ṽ/cm^–1^ = 1979s (t-CO), 1801s (μ-CO), 1561w, 1537m (μ-CN), 1454w. ^1^H NMR (CDCl_3_): δ/ppm = 7.70, 7.60 (d, *J* = 7.6 Hz, 2H), 7.52–7.35 (m, 3H) (Ph); 6.61, 6.21 (d, ^2^ *J*_HH_ = 15.0 Hz, 1H; NCH); 5.99, 5.87 (d, ^2^ *J*_HH_ = 15.1 Hz, 1H, NCH’); 4.97, 4.90 (s, 5H, Cp); 4.75, 4.64 (s, 5H, Cp’); 4.55, 4.11 (s, 3H, NCH_3_); *cis*-*E*/*Z* isomer ratio = 1.0.

#### 4.2.9 [Fe_2_Cp_2_Cl­(CO)­(μ-CO)­{μ-CNBn_2_}], 3d

Prepared from [**1d**]­CF_3_SO_3_ (200 mg, 0.292 mmol) according to the general procedure. Yield: 97 mg, 61%. Soluble in CH_2_Cl_2_, acetone, poorly soluble in Et_2_O, insoluble in hexane. Cube-shaped dark brown crystals of **3d** were obtained from a CH_2_Cl_2_ solution layered with hexane and settled aside at −20 °C. Anal. Calcd for C_27_H_24_ClFe_2_NO_2_: C, 59.87; H, 4.47; N, 2.59. Found: C, 58.57; H, 4.17; N, 2.61. IR (solid state): ṽ/cm^–1^ = 3123w, 3089w, 3057w, 3023w, 2912w; 1974s, 1952m-sh (t-CO); 1806s, 1794s (μ-CO); 1601w, 1584w, 1556w, 1507m, 1451m, 1427m, 1418m, 1348m, 1262w, 1228w, 1214w, 1115w, 1109w, 1078w, 1063w, 1027w, 1013m, 998m, 870w, 845m, 828m, 773s, 742m, 697s. IR (CH_2_Cl_2_): ṽ/cm^–1^ = 1981s (t-CO), 1802s (μ-CO), 1520m (μ-CN), 1509m, 1497w, 1454w. ^1^H NMR (CDCl_3_): δ/ppm = 7.46–7.36 (m, 8H), 7.32–7.28 (m, 2H) (Ph); *6*. *50*, 6.44, 6.14, *5*. *96* (d, ^2^ *J*_HH_ = 15.0 Hz, 1H + 1H, NCH); *5*. *81*, 5.71, 5.64, *5*. *62* (d, ^2^ *J*_HH_ = 15.0 Hz, 1H + 1H, NCH’); *4*. *79*, 4.78 (s, 5H, Cp); 4.68, *4*. *66* (s, 5H, Cp’); *cis*/*trans* isomer ratio = 5.8. ^13^C­{^1^H} NMR (CDCl_3_): δ/ppm = 341.6 (μ-CN); 266.3 (μ-CO); 212.1 (t-CO); 136.0, 134.6 (Ph/C_ipso_); 129.4, 129.3 (Ph/C_ortho_); 128.5, 128.4 (Ph/C_para_); 128.1, *127*. *8*, *127*. *6*, 127.5 (Ph/C_meta_); 86.9, 86.7­(br.), *86.5* (Cp+Cp’); *66*. *8*, 66.4, *66*. *3*, 65.9 (NCH_2_).

#### 4.2.10 [Fe_2_Cp_2_Cl­(CO)­(μ-CO)­{μ-CNMe­(Xyl)}], 3e

Prepared from [**1e**]­CF_3_SO_3_ (602 mg, 0.969 mmol or 300 mg, 0.969 mmol) according to the general procedure. Yields: 315 mg, 75%, *cis*-*E*/*Z* isomer ratio 7.4 or 177 mg, 76%, *cis*-*E*/*Z* isomer ratio ≥30, respectively. IR (solid state): ṽ/cm^–1^ = 3123w, 3082w, 2956w, 2927w, 2859w; 1957s, 1943m-sh (t-CO); 1789s (μ-CO); 1506m, 1466m, 1443m, 1418m, 1377m, 1260w, 1223w, 1167w, 1120w, 1103w, 1085w, 1063w, 1015w, 1000w, 874w, 831m, 808w, 781m, 767m, 737m, 668m. IR (CH_2_Cl_2_): ṽ/cm^–1^ = 1980s (t-CO), 1800s (μ-CO), 1507m (μ-CN). ^1^H NMR (CDCl_3_): δ/ppm = 7.36–7.28 (m, 3H, C_6_H_3_); 4.92, *4*. *51* (s, 3H, NCH_3_); *4*. *86*, 4.77 (s, 5H, Cp); 4.31, *4*. *22* (s, 5H, Cp’); 2.76, *2*. *72* (s, 3H, CCH_3_); *2*. *37*, 2.19 (s, CCH_3_’).

#### 4.2.11 [Fe_2_Cp_2_Cl­(CO)­(μ-CO)­{μ-CNMe­(4-C_6_H_4_OMe)}], 3f

Prepared from [**1f**]­CF_3_SO_3_ (300 mg, 0.481 mmol) according to the general procedure. Yield: 125 mg, 55%. IR (solid state): ṽ/cm^–1^ = 3083w, 2953w, 2935w, 2836w; 1947s (t-CO); 1776s (μ-CO); 1607w, 1556w, 1507m, 1498m, 1456w, 1418w, 1381m, 1296w, 1250m, 1177w, 1112w, 1102w, 1034m, 1024w, 1011w, 1000w, 855w, 841m, 831m, 821m, 780s, 703m. IR (CH_2_Cl_2_): ṽ/cm^–1^ = 1979s (t-CO), 1800m (μ-CO), 1712w, 1688w, 1607w, 1515m-sh (μ-CN), 1506m. ^1^H NMR (CDCl_3_): δ/ppm = *7*. *94*, 7.51 (d, ^3^ *J*_HH_ = 8.8 Hz, 2H), 7.09 (app-t, ^3^ *J*_HH_ = 9.1 Hz, 2H) (C_6_H_4_); 4.99, *4*. *59* (s, 3H, NCH_3_); *4*. *80*, 4.73 (s, 5H, Cp); 4.27, *4*. *16* (s, 5H, Cp’); 3.92 (s, 3H, OCH_3_); *cis*-*E*/*Z* isomer ratio = 2.0.

### 4.3 X-ray Crystallography

Crystal data and collection details for [**1a**]­NO_3_, [**1b**]­NO_3_·0.5CH_2_Cl_2_, [**1e**]­NO_3_, *E*-[**2c**]­CF_3_SO_3_ and **3d** are reported in Table S23. Data were recorded on a Bruker APEX II diffractometer equipped with a PHOTON2 detector using Mo–Kα radiation. Data were corrected for Lorentz polarization and absorption effects (empirical absorption correction SADABS).^82^ The structures were solved by direct methods and refined by full-matrix least-squares based on all data using *F* ^2^.^83^ Hydrogen atoms were fixed at calculated positions and refined by a riding model. All non-hydrogen atoms were refined with anisotropic displacement parameters.

### 4.4 NMR-Based Experiments in Deuterated Aqueous Media

#### 4.4.1 Selection of Internal Standards

Dimethylsulfone (Me_2_SO_2_) and sodium 3-(trimethylsilyl)-1-propanesulfonate (Me_3_Si­(CH_2_)_3_SO_3_Na, DSS) were used as internal standards as nonvolatile, water-soluble and relatively inert solids.^84^ In this respect, both compounds are assumed to be fully stable in the conditions explored. DSS is advantageous over Me_2_SO_2_ due to a shorter relaxation time, absence of overlaps with DMEM components or other species, stability in basic solution. Me_2_SO_2_ was preferred over DSS in D_2_O solution due to its nonionic nature, avoiding unwanted ion–ion interactions and/or metathesis reactions, especially at high concentrations.

#### 4.4.2 Spectra Acquisition

^1^H NMR spectra were acquired with the following parameters: 45° pulse, 25 ppm spectral window, 15 s delay time, 65,536 points (64 K), 3.40 s pulse width, 5.24 s acquisition time, 10 or 16 scans in more dilute solutions. These parameters revealed optimal to obtain reliable data,^85^ in terms of signal-to-noise ratio and accuracy in the relative integrals for both internal standards (Me_2_SO_2_ and DSS) in the freshly prepared solution and at the end of the experiments (Figures S18–S20).

#### 4.4.3 Spectral Processing

All spectra were referenced to Me_2_SO_2_ [δ/ppm = 3.14 (s, 6H, SMe)] or DSS [δ/ppm = 0.00 (s, 9H, SiMe_3_)], manually phased then processed with an exponential apodization function of 1.0 Hz, zero-filled to 262,144 points (256 K). Finally, spectra were baseline-corrected by manually applying a point at the base of each peak of interest.^86^ The multipoint baseline correction was particularly important to obtain reliable and consistent data across different experiments. The limit of quantitation of the technique is approximately 2·10^–4^ mol/L for a freshly prepared solution, but it is sensibly higher (≈5·10^–4^ mol/L) after 72 h due to broadening of the HDO peak due to H/D exchange with moisture, possible suspended particles not removed by filtration and/or traces of paramagnetic species in solution.

#### 4.4.4 Stock Solution Preparation, Storage, and Standardization

A stock solution of Me_2_SO_2_ (*ca.* 40–50 mg) in D_2_O (*ca.* 80 mL) was prepared in a 150 mL Schlenk flask. Another stock solution was prepared with powdered Dulbecco’s Modified Eagle Medium (DMEM; 10 g/L; with 1000 mg/L glucose and l-glutamine, without sodium bicarbonate and phenol red; D2902-Merck), NaHCO_3_ (3.7 g/L; 44 mM), DSS (*ca.* 30 mg) and D_2_O (50 mL). Both solutions were stored under N_2_ at 4 °C. The concentration of the internal standard (*c*_std_) was determined by ^1^H NMR on a weighed aliquot of the solution (assuming *d* = 1.107 g/mL as pure D_2_O) spiked with a known amount (*ca.* 50 mg) of a solution of another reference substance (*ca.* 100 mg in 1.7–2.0 g H_2_O). In this regard, glycine (spin–lattice relaxation time *T*_1_ ≈ 3 s in D_2_O)^87^ was used for the standardization of Me_2_SO_2_ (^1^H NMR spectrum run with *d*1 = 30 s), while sodium formate (*T*_1_ ≈ 9 s in D_2_O)^88^ was used for the standardization of DSS (^1^H NMR spectrum run with *d*1 = 45 s), to avoid overlaps of signals with DMEM components.

#### 4.4.5 Experimental Set Up in Aerobic Conditions

In a 5 mL test tube, the selected amount of [**1**]­NO_3_ or [**1**]­CF_3_SO_3_ and other reactant(s), if present, were dissolved in the appropriate volume of D_2_O or DMEM-*d* stock solutions (method #1). Alternatively, a known amount of [**1**]­NO_3_ or [**1**]­CF_3_SO_3_ (*m*_Fe_2__ > 15 mg) and internal standard (Me_2_SO_2_ or DSS) was dissolved into a weighed amount of D_2_O or DMEM-*d* (*m*_D_2_O_) (method #2). In the latter case, the freshly prepared solution was portioned and each aliquot was treated with different reactants or serially diluted with weighed amounts of for different experiments. The freshly prepared solution (*t*_0_) was filtered over a small Celite pad into an NMR tube. All solutions were exposed to ambient light/air except where otherwise noted (in the dark, Table S19); however NMR tubes were sealed with cap and parafilm to minimize H/D exchange and loss of volatiles over time, including the solvent. The solution was analyzed by ^1^H NMR then the tube was placed in a thermostated, magnetically stirred silicone oil bath at 37 °C for 72 h or any other temperature/time conditions as specified in Tables S3–S10 and S14–S19. Next, the solutions were cooled to room temperature and filtered through Celite to remove any insoluble material. Solutions containing 1,3,5-triaza-7-phosphaadamantane (PTA) were kept for 144 h and were also analyzed by ^31^P NMR (Table S18).

#### 4.4.6 Experimental Set Up in Anaerobic Conditions

H_2_O was deaerated by bubbling argon for 30′. In a 50 mL Schlenk tube under Ar, pyrogallol (1,2,3-trihydroxybenzene; 25 mg, 0.20 mmol) and NaOH (40 mg, 1.0 mmol) were dissolved in deaerated H_2_O (10 mL). In a 25 mL Schlenk tube under Ar, the selected amount of [**1a**,**c**,**e**]­NO_3_ was dissolved in the appropriate volume of D_2_O or DMEM-*d* stock solution. Next, a small test tube was introduced into the larger Schlenk tube and was half-filled with the alkaline pyrogallol solution. The red solution in the bottom of the Schlenk tube was heated at 37 °C by full immersion into an oil bath. A rapid change from a colorless to a brown solution occurred in the presence of O_2_, due to the oxidation and condensation of pyrogallol,^58^ and in this case, experiments were discarded. Aliquots (*ca.*, 0.65 mL) of the freshly prepared (0 h) and of the final solution (72 h) were filtered through Celite and analyzed by ^1^H NMR (Tables S14–S16).

#### 4.4.7 Data Elaboration

The concentration of the diiron compound in the freshly prepared solution (**c**_**Fe2**_^**0**^) was determined according to the preparation method as follows.

Method #1: with respect to the known concentration of internal standard as *c*_Fe_^0^_2_ = [*I*_Fe_2__(*t*_0_)/*N*_Fe_2__]/[*I*_std_(*t*_0_)/*N*_std_]·*c*_std_ where *I*_Fe_2__(*t*_0_)/*N*_Fe2_ and *I*_std_(*t*_0_)/*N*_std_ represent the integrals of a selected signal of [**1**]^+^ and the internal standard, respectively, normalized for the number of protons.

Method #2: by accurate mass measurements as c_Fe_^0^_2_ = (*m*_Fe_2__/*M*_Fe_2__)/(*m*_D_2_O_/*d*) where the volume of the solution was calculated by assuming *d* = 1.107 g/mL as pure D_2_O.

The % amount of compounds in solution was calculated with respect to the internal standard (Me_2_SO_2_ or DSS). In particular, the residual amount (**%R**) of the starting material [**1**]^+^ was calculated as %*R* = [*I*_Fe_2__(*t*)/*I*_std_(*t*)]/[*I*_Fe_2__(*t*_0_)/*I*_std_(*t*_0_)]·100%, where *I*_Fe_2__ and *I*_std_ represent the relative integrals of a selected signal of [**1**]^+^ and the internal standard, respectively, at time *t* (*e.g.*, 72 h) and in the initial spectrum (*t*_0_). The formula simplifies to %*R* = [*I*_Fe_2__(*t*)/*I*_Fe_2__(*t*_0_)]·100% when the integral of the internal standard is given a constant value (preferably *I* = 6 for Me_2_SO_2_ and *I* = 9 for the trimethylsilyl group of DSS). In aqueous solution, the formula above was applied to the C_5_H_5_ (all compounds), NCH_3_ (all compounds), CCH_3_ (xylyl), CH_2_ (cyclohexyl) and NCH_2_ (benzyl) signals of [**1**]^+^ whenever possible (*i.e.*, except in case of overlaps with HDO or other signals, particularly those of components of the cell culture medium). The %*R* values were averaged whenever the relative difference was ≤5%. Otherwise, NCH_2_ and NCH_3_ signals were preferred for the calculations due to their shorter relaxation time.

Data for [**1b**–**e**]­NO_3_ and [**1e**]­CF_3_SO_3_ refer to the *cis* isomer as the only species observed in solution. Data for [**1a**]­NO_3_ and [**1a**–**c**]­CF_3_SO_3_ also take into account the *trans* isomer (*ca.*, 3% or 10%, respectively). No significant changes in the *cis*/*trans* isomer ratios were observed across all experiments, except those with [**1a**,**c**]­CF_3_SO_3_ and HCl.

The % relative amount of a compound Z in solution with respect to the starting organometallic cation was calculated as %*Z* = [*I*_Z_(*t*)/*N*_Z_]/[*I*_Fe_2__(*t*_0_)/*N*_Fe_2__]·100%, wherein *I*_Y_(*t*)/*N*_Z_ represents the integral of a selected signal of Z at time *t* and *I*_Fe_2__(*t*_0_)/*N*_Fe_2__ represents the integral of a selected signal of [**1**]^+^ in the initial solution (*t*_0_), both normalized with respect to the number of protons (*N*) and relative to the internal standard, which is assigned the same integral value in each spectrum. Results are compiled in Tables S3–S10 and S14–S19; NMR data are reported in the SI. Duplicate experiments were not carried out; therefore, data are reported without an experimental uncertainty.

### 4.5 UV–Vis Based Experiments in Aqueous Media

#### 4.5.1 Experimental Set Up in Aerobic Conditions

A known amount of [**1**]­NO_3_ or [**1**]­CF_3_SO_3_ (*m*_Fe_2__ ≥ 12 mg) was introduced in a 25, 50, 100 or 200 mL volumetric flask and magnetically stirred with deionized H_2_O for a sufficient time until completely dissolved, then made up to volume. In the few cases where complete dissolution was not achieved the solution was filtered through a G3 sintered glass filter. Except where otherwise noted (in the dark, Table S19), no special precaution was adopted to exclude ambient light and air. The freshly prepared solution (*t*_0_) was analyzed by UV–vis then partitioned in three to five 15 mL glass or polypropylene test tubes. The partitions were used as replicates (≈10–11 mL from 50–200 mL solutions or ≈8 mL for 25 mL stock solution) for the same experiment or for monitoring the process at 24 h intervals (Table S11). In this case, pH and conductivity were also recorded. Alternatively, each 10 mL aliquot (measured with a volumetric flask) was treated with a different reagent as indicated in Tables S14–S16. Concentrated aqueous solutions of each reagent were prepared in 5 mL volumetric flasks, so that the volume added (20–50 μL) to the solution of [**1**]^+^ is negligible. The test tubes were sealed and placed in a thermostated, magnetically stirred silicone oil bath. The solution was kept at 37 °C for 72 h. After 72 h or any other time interval (24–48–72 h), an aliquot of the solution was filtered through a cotton plug with a little Celite and the UV–vis spectrum was recorded. Before each sampling, the test tube was shaken to homogenize the mixture.

#### 4.5.2 Experimental Set Up in Anaerobic Conditions

H_2_O and DMEM-C (see Section 4.7) were deaerated by bubbling argon for 30′. The required amount/volume of [**1a**,**c**,**e**]­NO_3_, H_2_O or DMEM-C, and any other reagent as specified in Table S16, was transferred into a Schlenk tube. Next, a small test tube was introduced into the larger Schlenk tube and was half-filled with the alkaline pyrogallol solution (Section 4.4). The Schlenk tube was heated at 37 °C for 72 h, ensuring that the solution was fully immersed in the oil bath. UV–vis analyses of the freshly prepared solution and after 24, 48, or 72 h were carried out as described above.

#### 4.5.3 Spectra Acquisition

The method is based on an absorption peak of [**1**]^+^ centered at 340 nm with ε_340_ ≈ 5·10^3^ M^–1^·cm^–1^. UV–vis spectra were measured in quartz cuvettes of appropriate path length (*l* = 2 mm, 5 mm or 10 mm) so that the absorbance at 340 nm (A) of the freshly prepared solution falls within the higher part of the linear range of the Lambert–Beer law (≈1 < *A* < 1.3).^89^ Spectra were collected with a 750 nm/min scan rate and 1 nm resolution in the 250–800 nm range, then blank-subtracted with a spectrum of the solvent (H_2_O, DMEM-C) recorded with the same cuvette and baseline corrected by normalizing to 0 the absorbance around 800 nm (this last step corrects for small light scattering effects, on the order of 0.01 absorbance units. The resulting absorbance at 340 nm (*A*_340_) was used for the calculations.

#### 4.5.4 Data Processing

The concentration of [**1**]^+^ in the freshly prepared solution was determined as *c*_Fe2_^0^ = (*m*_Fe_2__/*M*_Fe_2__)/*V* when both mass and volume were accurately known. The molar absorption coefficient was calculated in the freshly prepared solution as ε_340_ = *A*_340_(*t*_0_)/(*l*·*c*_Fe_2__^0^) and was checked for consistency among different experiments of the same compound, otherwise the initial concentration of the solution was corrected accordingly [*c*_Fe2_^0^ = *A*_340_(*t*_0_)/(*l*·ε_340_)]. Similarly, whenever the mass or the volume were not accurately measured (*e.g.*, experiments in anaerobic conditions), *c*_Fe2_^0^ was determined from *A*_340_(*t*_0_) and ε_340_ known from other experiments.

The % residual amount of [**1**]^+^ in solution (%*R*) was calculated as %*R* = *A*_340_(*t*)/*A*_340_(*t*_0_)·100% where *A*_340_(*t*_0_) and *A*_340_(*t*) are the absorbance at 340 nm of the freshly prepared solution (*t*_0_) and at time *t* (*e.g.*, 72 h) at 37 °C. In case of replicates from the same stock solution, results are expressed as mean ± standard deviation. Results are compiled in Tables S3–S7, S11, S14–S17, S19; ε_340_ data are reported in the SI.

The relation between the relative amount of [**1**]^+^ and the absorbance at 340 nm is based on the assumption that no soluble species absorbing long-wave UV is formed during the thermal treatment. In this scenario, the blank-subtracted absorbance is entirely ascribable to the starting material both in the freshly prepared and final solution. The assumption was not applicable to aerobic experiments in DMEM solution because of the formation of species absorbing at 300 nm in the final solution.

### 4.6 NMR Based Experiments: Additional Data Analyses/Interpretation or Protocols

#### 4.6.1 Zero Order Kinetics (NMR, UV–Vis)

A plot of the concentration over time gives a straight line according to the generic integrated rate law for a zero-order reaction: *c*(*t*) = *c* ^0^ – *k*·t. The rate constant *k*, usually expressed as mol·L^–1^·s^–1^ (M/s), was herein more conveniently expressed as μmol·L^–1^·h^–1^ (μM/h). Data are shown in Figures S36, S38, S40, and S48. A rate constant was also estimated for a set of experiments with a fixed time (72 h) and a variable initial concentration of [**1a**]­NO_3_ (Figure 6). In this case, the combination of integrated rate law and percent residual amount (%*R*) of the starting material provides: %*R*(*t*) = *c*(*t*)/*c* ^0^·100% = (*c* ^0^ – *k*·*t*)/*c* ^0^·100% = (1 – *k*·*t*/*c* ^0^)·100%. At 72 h: %*R* ^72h^ = (1 – 72·*k*·/*c* ^0^)·100%. Data below *c*_Fe_^0^_2_ ≈ 1.5 mM are no longer well fitted by a zero-order kinetic model, regardless of the value set for the rate constant (Figure S33).

#### 4.6.2 Identification and Quantitation of Amines and Cyclopentadiene

Secondary and primary amines/ammonium salts potentially deriving from the heterolytic cleavage of N–C bonds in [**1a**–**f**]^+^ were prepared and/or characterized by ^1^H NMR in D_2_O (SI). Excess NaHCO_3_ was added to each solution to obtain the predominant species at physiological pH (the ammonium ion, the corresponding amine or comparable amounts of both, depending on the p*K*_a_). ^1^H NMR signals of CpH were obtained by addition of a NaCp solution in THF to D_2_O. These data were used as reference for the analysis of NMR spectra of [**1a**–**f**]^+^ in various conditions (Section 4.4); results are included in Tables S3–S10 and S14–S19.

#### 4.6.3 Determination of Solubility in D_2_O

A sufficient amount of the selected compound (at least double the amount required for saturation) and the D_2_O stock solution (*ca.* 0.8 mL) was vigorously stirred at ambient temperature (*ca.* 25 °C) for at least 5 h, allowing to reach saturation. The less soluble compounds, *e.g.*, [**1d**]­CF_3_SO_3_ were kept under stirring for 24 h, due to their presumably lower rate of dissolution.^90^ For the most soluble compounds, *e.g.*, [**1a**,**b**,**c**,**e**]­NO_3_, a smaller volume of saturated solution was prepared (*ca.* 0.4 mL) and subsequently diluted with pure D_2_O in the NMR tube, in order to minimize the consumption of the starting material. Specifically, the very high solubility of [**1a**]­NO_3_ with respect to the concentration of the internal standard in the stock D_2_O solution (Me_2_SO_2_, usually *ca.* 6 mM) resulted in a disproportionate intensity of the related signals. Therefore, [**1a**]­NO_3_ was suspended with an accurately weighted amount of Me_2_SO_2_ (*ca.* 15–20 mg) and D_2_O (0.3–0.4 g) under stirring until saturation.

For each compound, the suspension (saturared solution + solid) was filtered through Celite into an NMR tube. The concentration of the diiron compound (= solubility, *S*) was calculated as *S* = [*I*_Fe_2__/*N*_Fe_2__]/[*I*_Me_2_SO_2__/6]·*c*_std_, where *I*_Fe_2__/*N*_Fe_2__ and *I*_Me_2_SO_2__/6 represent the integrals of a selected signal of [**1**]^+^ and the internal standard, respectively, normalized for the number of protons. NCH_2_ and NCH_3_ signals were preferred for the calculations due to their shorter relaxation time. Solubility data are collected in Table S2.

#### 4.6.4 Thermal Treatment in Hydrochloric Acid Solution

A solution of [**1d**–**f**]­CF_3_SO_3_ (50–60 mg; 0.072–0.097 mmol in EtOH (1.0 mL) was treated with 2.0 M aqueous HCl (0.35–0.50 mL, 0.70–1.0 mmol, *ca.*, 10 equiv) and deionized water up to 10 mL total volume. The resulting red solution or suspension was heated at 80 °C. After 48 h, the conversion of the starting material was low or modest in each case as indicated by the appearance of the reaction mixture: turbid pale red solution + red solid for [**1d**]^+^, turbid red suspension for [**1e**]^+^, clear red solution for [**1f**]^+^. An aliquot (*ca.*, 1 mL) of each mixture was taken to dryness under vacuum and analyzed by ^1^H NMR (CDCl_3_ or D_2_O), showing [**1d**–**f**]^+^.

### 4.7 Analysis of Iron-Containing Compounds in Solution and in the Solid State

#### 4.7.1 Cell Culture Medium Solutions

The experiments described in the following sections were carried out in different solutions prepared from powdered DMEM (D2902-Merck), as specified in the following. All these solutions were stored under N_2_ at 4 °C. **DMEM-C**: Powdered DMEM (10 g/L), NaHCO_3_ (3.7 g/L, 44 mM) in deionized water; pH = 7.7. **DMEM-C-dil**: Powdered DMEM (1 g/L), NaHCO_3_ (3.7 g/L, 44 mM) in deionized water; 7.7 < pH < 8.3. **DMEM-P**: Powdered DMEM (10 g/L), K_2_HPO_4_ and NaHPO_4_ (3:1 molar ratio; 100 mM total phosphate) in deionized water; pH = 7.2.

#### 4.7.2 Iron Quantitation via ICP-OES

Compounds [**1c**,**e**]­NO_3_ and [**1e**]­CF_3_SO_3_ (*m*_Fe_2__ = 30 mg) were stirred in an appropriate volume (*V*_i_) of H_2_O or DMEM-C (10–20 mL, depending on the water solubility of the compound) until complete dissolution. The resulting solutions (*c*_Fe2_^0^ = 5.77, 3.74, 2.41 mM for [**1c**]­NO_3_, [**1e**]­NO_3_ and [**1e**]­CF_3_SO_3_ respectively), together with H_2_O and DMEM-C as blank tests, were kept at 37 °C for 72 h then filtered (G4 sintered glass filter) to remove any insoluble material. The filtrate solution were collected in volumetric flasks (*V*_f_ = 25 or 50 mL), treated with 68% HNO_3(aq)_ (0.75 or 1.50 mL, respectively, to obtain a final 2% HNO_3_ solution) and diluted with water up to the volumetric mark. The nominal amount of iron (*c*_Fe_/ppm) in this solution was calculated as 2·(*m*_Fe_2__·*M*_Fe_/*M*_Fe_2__/*V*_f_)·1000, where *m*_Fe_2__ is the initial amount of the diiron compound (molar mass *M*_Fe_2__), *M*_Fe_ = 55.845 g/mol and *V*_f_ is the volume of the graduated flask. The solutions were analyzed for their total soluble iron content by ICP-OES. Results are expressed in blank-subtracted ppm of iron (mg Fe/L solution; 0.11 ppm Fe in the aqueous solution; 0.18 ppm Fe in the DMEM-C solution). The % relative amount of iron in the final solution was calculated as the ratio of the measured ppm value with respect to the nominal ppm amount. This value would be 100% (nominal Fe = measured Fe) if no insoluble iron compounds were formed over 72 h at 37 °C (removed by filtration). All data and results are reported in Table S12.

#### 4.7.3 Isolation and Characterization of Water-Insoluble Products

In a 50 or 100 mL round-bottom flask, the selected iron compound (FeSO_4_, (NH_4_)_2_FeSO_4_·6H_2_O, [**1a**–**c**,**e**,**f**]­CF_3_SO_3_, [**1b**,**c**,**e**]­NO_3_; 50–90 mg) was vigorously stirred in an appropriate volume of the selected aqueous solution (H_2_O, DMEM-P, DMEM-C or DMEM-C-dil; 25–50 mL). Stirring was prolonged for a few hours in those cases approaching the solubility limit of the compound. The resulting mixture was filtered (sintered glass G4 filter) to remove the undissolved starting material, if present. The resulting clear solution was collected in a 50 or 100 mL tube which was closed with a rubber cap and kept at 37 °C for 7–17 days. In all cases except FeSO_4_ and Mohr’s salt in water, a progressive increase in turbidity was observed over time, followed by the formation of an orange-brown solid. The final mixture was centrifuged (5 min, 2800 rpm) and the solution was discarded. The solid was suspended with water, separated by centrifugation and the aqueous solution was removed (×5). Finally, the brown solid was thoroughly dried under vacuum (40 °C) and collected as a powder, although a fraction remained as a thin coating on the surface of the reaction vessel and the centrifuge tubes. Samples were analyzed by CHNS content, IR and Raman, then were suspended in CH_2_Cl_2_ (≤0.4 mL) and the resulting solution was analyzed by IR and ESI-MS. Details of solution preparation and results are compiled in Table S13. CHNS analyses showed some intrasample variability, possibly related to the heterogeneous nature of the particles; therefore, a relative standard deviation <10% was considered acceptable.

#### 4.7.4 Preparation and Characterization of Reference Fe Compounds

Solutions of FeSO_4_ or Fe_2_(SO_4_)_3_ in H_2_O (15–20 mL) were treated with NaOH, Na_2_CO_3_, NaHCO_3_ or phosphate buffer (pH ≈ 7.4). The resulting precipitates were isolated by filtration, washed with water and characterized by CHNS, IR and Raman analyses. Details are reported in the SI.

### 4.8 Decomposition of Chloride Complexes with Silver Salts in Aqueous Media

#### 4.8.1 General Procedure (Homogeneous Reaction)

A brown solution of **3c**–**f** (*ca.* 50 mg, 0.10 mmol) in acetone (4.5 mL) was treated with water (0.50 mL, 27.8 mmol, *ca.* 280 equiv vs Fe_2_) and Ag­(CF_3_SO_3_) (27 mg, 0.10 mmol, 1.0 equiv). The mixture was stirred at room temperature for 18–21 h under protection from the light, affording a light brown solution and a colorless solid. IR analyses (acetone/water 5:1 *V*/*V* as solvent) indicated complete conversion for **3e** whereas minor amounts of **3c**,**d**,**f** were still present. Next, the suspension was filtered on a Celite pad and the filtrate solution was taken to dryness under vacuum. For **3e**: a small aliquot of the solution was analyzed by GC-MS. In one case, 2 M HCl (0.10 mL, 0.2 mmol) was added to the mixture before taking to dryness under vacuum. The residue was treated with Me_2_SO_2_ (11 mg, 0.12 mmol) and acetone (few mL); the solution was transferred into a test tube and volatiles were removed under vacuum (RT). The brown sticky residue was stirred with D_2_O (0.8 mL) for 2 h. The aqueous solution was filtered through Celite and analyzed by ^1^H NMR. No FeCp compound was detected. The relative amount of compounds in solution with respect to the starting material (**3c**–**f**) was calculated based on the relative integral with respect to Me_2_SO_2_ as internal standard. Results are reported in Table S22.

#### 4.8.2 Alternative Procedure (Biphasic Reaction)

A solution of **3a** (11 mg, 0.020 mmol) in CDCl_3_ (1.5 mL) was treated with a 0.21 mol/L aqueous solution of AgNO_3_ (191 μL, 0.040 mmol, 2 equiv). The mixture was kept under vigorous stirring for 6 h at room temperature then centrifuged. The organic phase was analyzed by ^1^H NMR. Results are reported in Table S22.

### 4.9 DFT Calculations

All geometries were optimized with ORCA 6.1.3 ^91^ using the B97 functional with the zero-order regular approximation (ZORA^92^ to take relativistic effects into account and in conjunction with a single-ζ quality basis set (ZORA-SVP) and the auxiliary basis set SARC/J. The energy was then estimated with a single point calculation using triple-ζ quality basis set (ZORA-def2-TZVP). The dispersion corrections were introduced using the Grimme D3-parametrized correction and the Becke–Johnson damping to the DFT energy.^93^ All the structures were confirmed to be local energy minima (no imaginary frequencies) or saddle points in the case of transition states (one imaginary frequency). The solvent was considered through the continuum-like polarizable continuum model (C-PCM, water).

## Supplementary Material





## Notes

IR and NMR spectra of aminocarbyne complexes (solid state and organic solvents); ^1^H NMR, UV–vis, pH, conductivity and ICP-OES analyses of aqueous solutions of diiron compounds in various conditions; preparation and characterization of reference ammonium salts and inorganic iron compounds; CHNS, IR, Raman, ESI-MS characterization of water-insoluble iron compounds (PDF)
DFT optimized structures (XYZ)

## ⊥

Institute of Functional Materials and Catalysis, Faculty of Chemistry, University of Vienna, Währinger Str. 38, 1090 Vienna, Austria
