# Supplementary material for: The Reactivity of Diiron(I) Bis-cyclopentadienyl Tricarbonyl Aminocarbyne Complexes in Aqueous Media: A Case Study for Iron-Based Anticancer Agents
Source: Inorg Chem. 2026 Jun 11;65(25):14250–73. doi: 10.1021/acs.inorgchem.6c01829 (PMC13321316; doi:10.1021/acs.inorgchem.6c01829)
Supplement: Supplementary file 1 [file ic6c01829_si_001.pdf]

Supporting Information

The reactivity of diiron(I) bis-cyclopentadienyl tricarbonyl aminocarbyne complexes in aqueous media: a case study for iron-based anticancer agents

Eleonora Dolcher,[a,#] *Federico Simonelli,*[a,#,£] *Fatima Nigro,*[a] *Stefano Zacchini,*[b] *Beatrice Campanella,*[c] *Gianluca Ciancaleoni,*[a] *Fabio Marchetti,*[a] *Lorenzo Biancalana*[a*]

[a] Department of Chemistry and Industrial Chemistry, University of Pisa, Via G. Moruzzi 13, 56124 Pisa, Italy.

[b] Department of Industrial Chemistry "Toso Montanari", University of Bologna, Via Piero Gobetti 85, 40129 Bologna, Italy.

[c] Istituto di Chimica dei Composti Organometallici, Consiglio Nazionale delle Ricerche, Via G. Moruzzi 1, 56124 Pisa, Italy

[#] The authors contributed equally to the work

£ Current affiliation: Institute of Functional Materials and Catalysis, Faculty of Chemistry, University of Vienna, Währinger Str. 38, 1090 Vienna, Austria.

* lorenzo.biancalana@unipi.it

Table of contents	Page(s)
Literature data on the products of the decomposition process (**Table S1**)	S3
IR and NMR characterization of tricarbonyl aminocarbyne complexes, nitrate salts (**Figures S1-S16**)	S4-S11
Spectroscopic characterization in aqueous solution (**Table S2, Figure S17**)	S12-S14
Optimization of parameters for ^{1}H NMR experiments (**Figures S18-S20**)	S15-S16
NMR and UV-Vis analyses of solutions of diiron compounds in water or DMEM (**Figures S21–S40**, **Tables S3–S10**)	S17-S34
Preparation, characterization and identification of amines/ammonium salts and cyclopentadiene (**Figures S41-S47**)	S35-S43
Further UV-Vis, conductivity, pH, Raman and ICP-OES analyses of solutions (**Figures S48–S50**, **Tables S11-S12**)	S44-S46
Characterization of water-insoluble iron compounds (**Figures S51-S70**, **Table S13**)	S47-S71
NMR and UV-Vis analyses of aqueous solutions of diiron complexes in various conditions (**Figures S71–S75**, **Tables S14–S17**)	S72-S80
Activation of tricarbonyl complexes in presence of PTA and characterization of a dicarbonyl PTA complex (**Figures S76-S84**, **Table S18**)	S81-S86
NMR and UV-Vis analyses of aqueous solutions of diiron complexes in the dark (**Table S19**)	S87
Computational studies (**Tables S20,S21**)	S88-S89
Chlorido dicarbonyl complexes: IR and NMR characterization and silver-mediated decomposition in aqueous solution (**Figures S85–S97**, **Table S22**)	S90-S96
X-ray crystallography (**Table S23**)	S97
References	S98-S100

Literature data on the products of the decomposition process

Table S1. Identified products of the decomposition process of diiron(I) aminocarbyne compounds of the type $[Fe_2Cp_2(CO)(L)(\mu\text{-}CO)(\mu\text{-}CNRR')]Y$ in aqueous solution ($Y = CF_3SO_3$ unless otherwise specified).

Precursor	Conditions	Technique: product observed	Ref.
R = Me, R' = Me, Bn, Xyl<br>L = CO	D_2O and CD_3OD/D_2O 7:2 *V*/*V*<br>37 °C, 72 h	^{1}H NMR:<br>R(Me)NH (R = Me, Bn, Xyl?)	1
R = Me, R' = Me, Cy, Xyl<br>L = CO	H_2O, 37 °C, 72 h	Raman:<br>Me: α-Fe_2O_3 (hematite)<br>Xyl: α-Fe_2O_3 + Fe_3O_4 (magnetite)<br>Cy: γ-Fe_2O_3 (maghemite)	1,2
R = Me, R' = Me, Cy, Xyl, All<br>or R = R' = Bn<br>L = CO	H_2O + 5% MeOH<br>37 °C, 24-48 h	GC-TCD: CO	2
R = R' = Me<br>L = CO<br>Y = NO_3	10 mM PBS (pH = 7.4), 25 °C, 350 nm irradiation (15 min, 6 mW/cm^2)	Myoglobin assay: CO<br>(2.2 eq.)	3
R = Me, R' = Cy, Xyl, Anis, Naph<br>or R = R' = Bn<br>L = PR''$_3$ (various)<br>14 examples	D_2O or D_2O/CD_3OD 1:1 or 2:5 *V*/*V*,<br>37 °C, 72 h	^{1}H NMR: O=PR''$_3$	1,4,5
R = Me, R' = Cy<br>L = pyridine (various)<br>5 examples	D_2O/CD_3OD 1.3:1–4:1 *V*/*V*, 37 °C, 72 h or DMEM-d/CD_3OD, 37 °C, 24 h	^{1}H NMR: pyridine	6
R = Me, R' = Cy<br>L = NH_2R'' (various)<br>6 examples	D_2O/CD_3OD or DMEM-d/CD_3OD<br>1:1 – 6:1 *V*/*V*, 37 °C, 72 h	^{1}H NMR: NH_2R''	7
R = Me, R' = Me, Xyl<br>L = NCMe	DMEM-d/CD_3OD 6:1 or 5:2 *V*/*V*, 37 °C, 72 h	^{1}H NMR: MeCN	7
R = Me, Cy, Anis, R' = Me<br>L = CNR'' (various)<br>5 examples	D_2O/CD_3OD 1:1 *V*/*V*,<br>37°C, 72-96 h	^{1}H NMR: CpH, R(Me)NH (R = Me, Cy, Anis),	8

Abbreviation list: Cy = cyclohexyl, Xyl = xylyl, 2,6-dimethylphenyl, Bn = benzyl, All = allyl, Anis = 4-methoxyphenyl, Naph = 2-naphthyl, PBS = phosphate buffer saline, DMEM-d = deuterated Dulbecco's modified Eagle Medium, Cp = cyclopentadienyl.

IR and NMR characterization of tricarbonyl aminocarbyne complexes, nitrate salts

Figure S1. Solid-state IR spectrum (650-4000 cm^{-1}) of $[Fe_2Cp_2(CO)_2(\mu\text{-CO})\{\mu\text{-CNMe(Cy)}\}]NO_3$, [**1b**]$NO_3$.

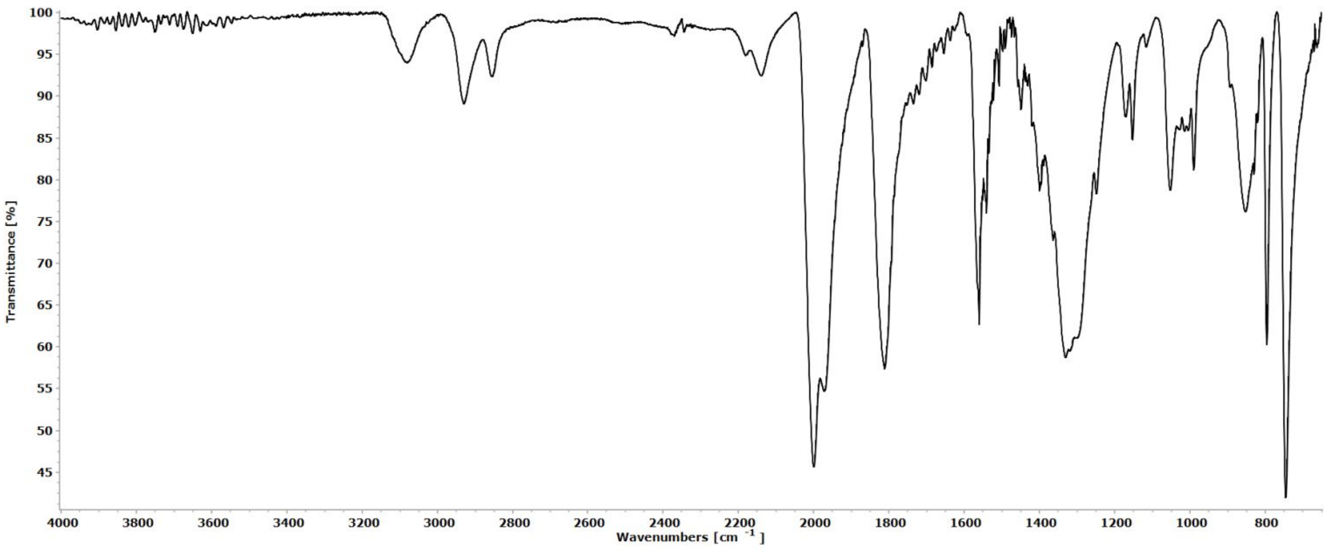

Figure S2. ^1H NMR spectrum (401 MHz, acetone-d_6) of [**1b**]NO_3.

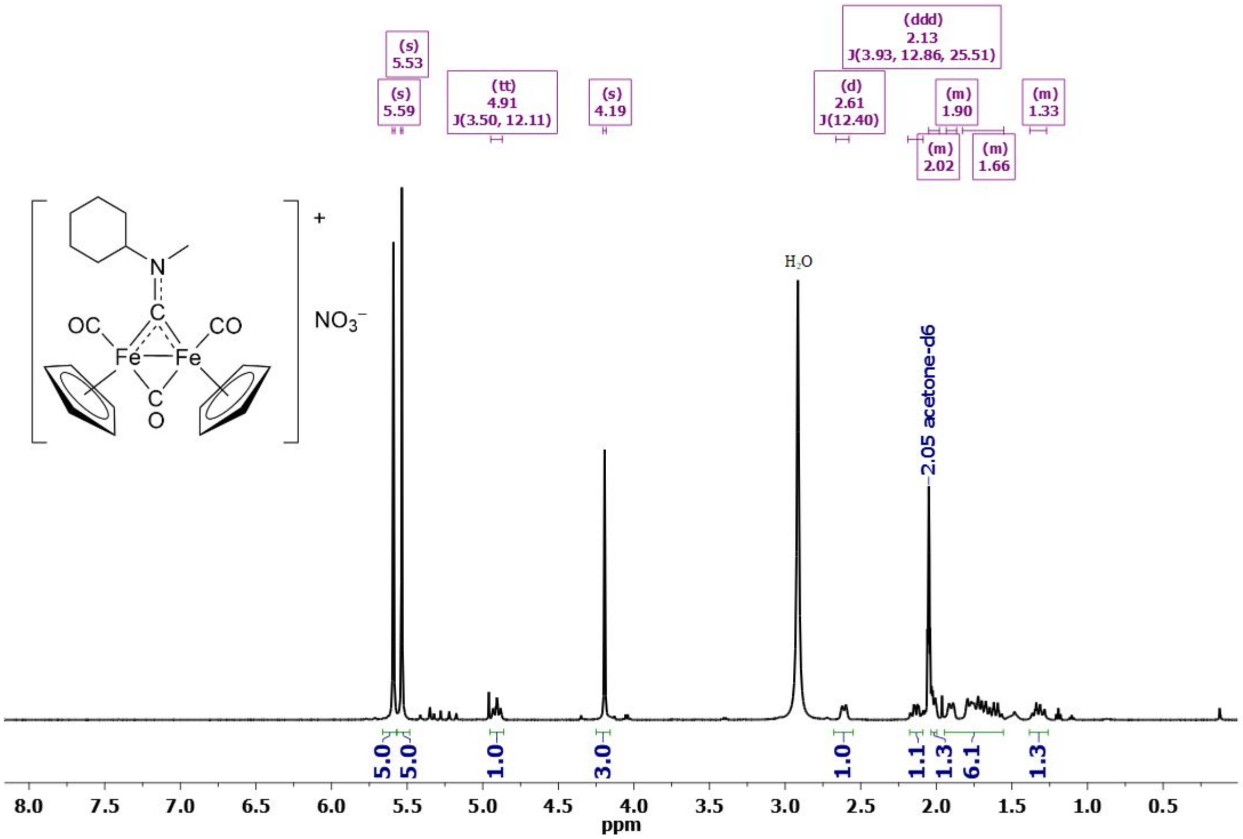

Figure S3. $^{13}C\{^{1}H\}$ NMR spectrum (126 MHz, acetone-d_6) of [**1b**]NO_3.

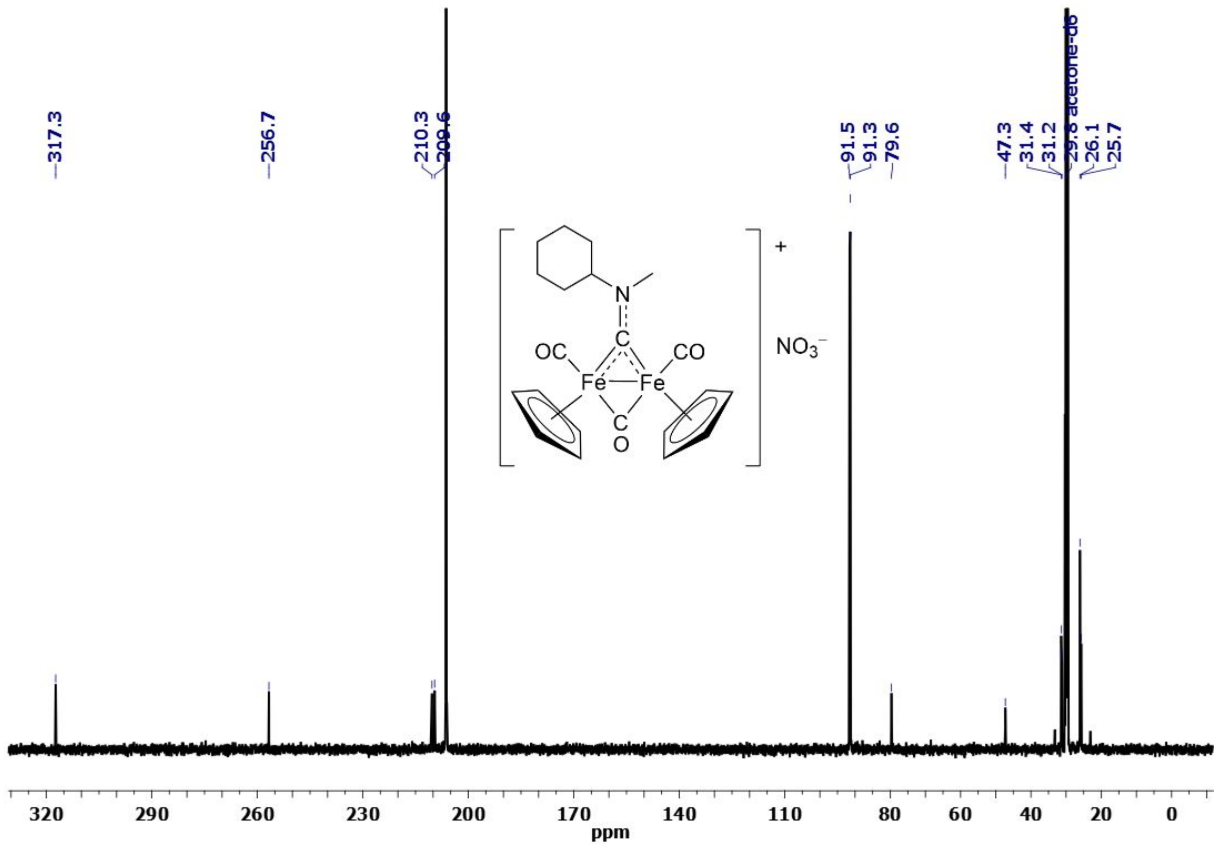

Figure S4. Solvent-subtracted IR spectrum (1300-2300 cm^{-1}) of [**1b**]NO_3 in CH_2Cl_2.

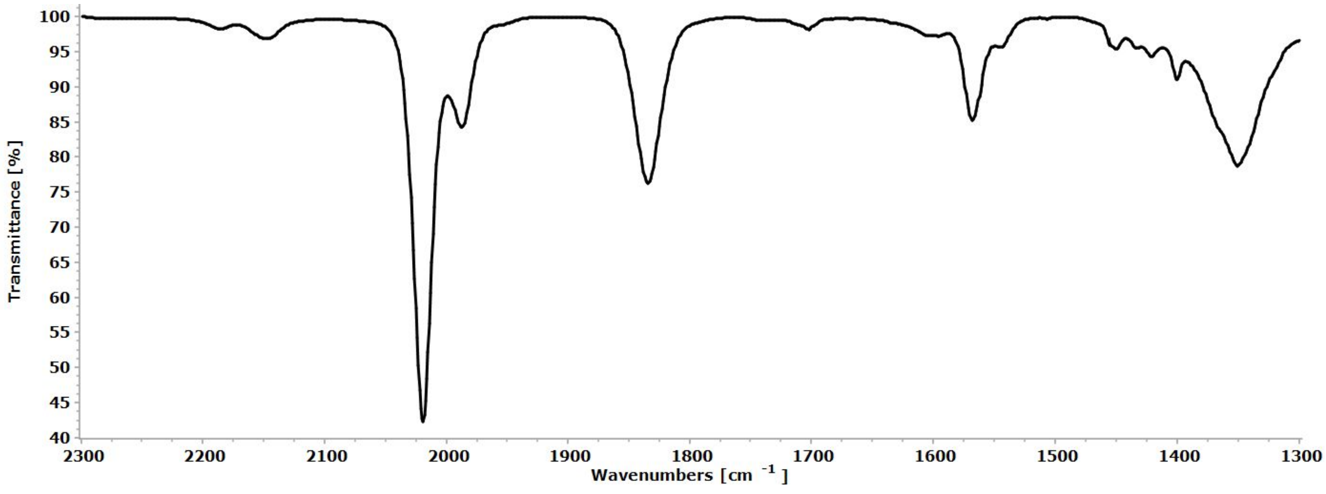

Figure S5. Solid-state IR spectrum (650-4000 cm^{-1}) of $[Fe_2Cp_2(CO)_2(\mu\text{-}CO)\{\mu\text{-}CNMe(Bn)\}]NO_3$, [**1c**]$NO_3$.

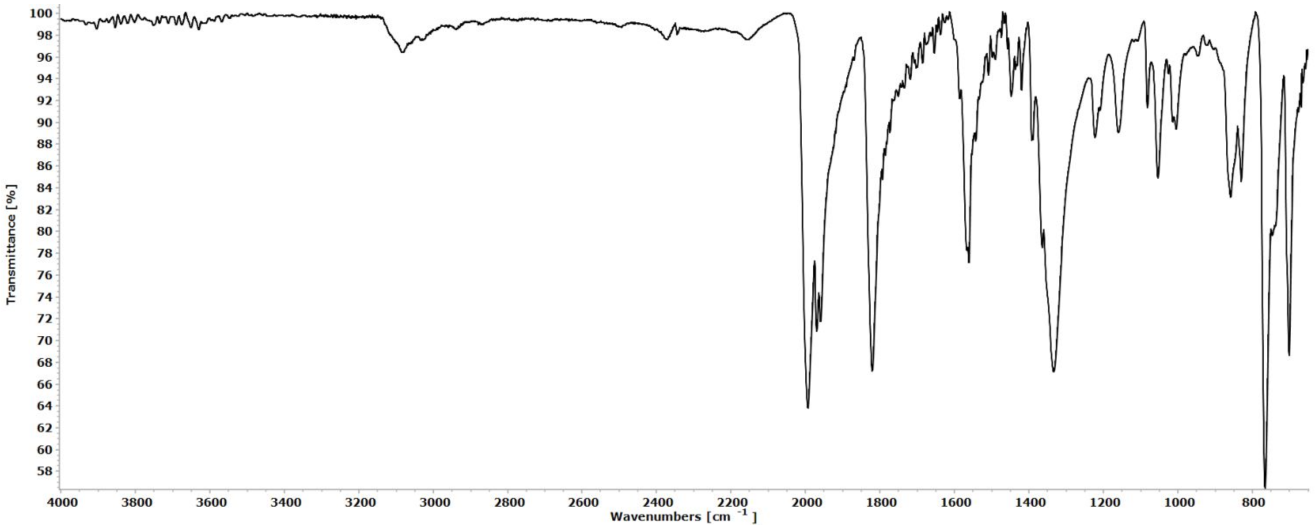

Figure S6. ^{1}H NMR spectrum (401 MHz, acetone-d$_6$) of [**1c**]NO_3.

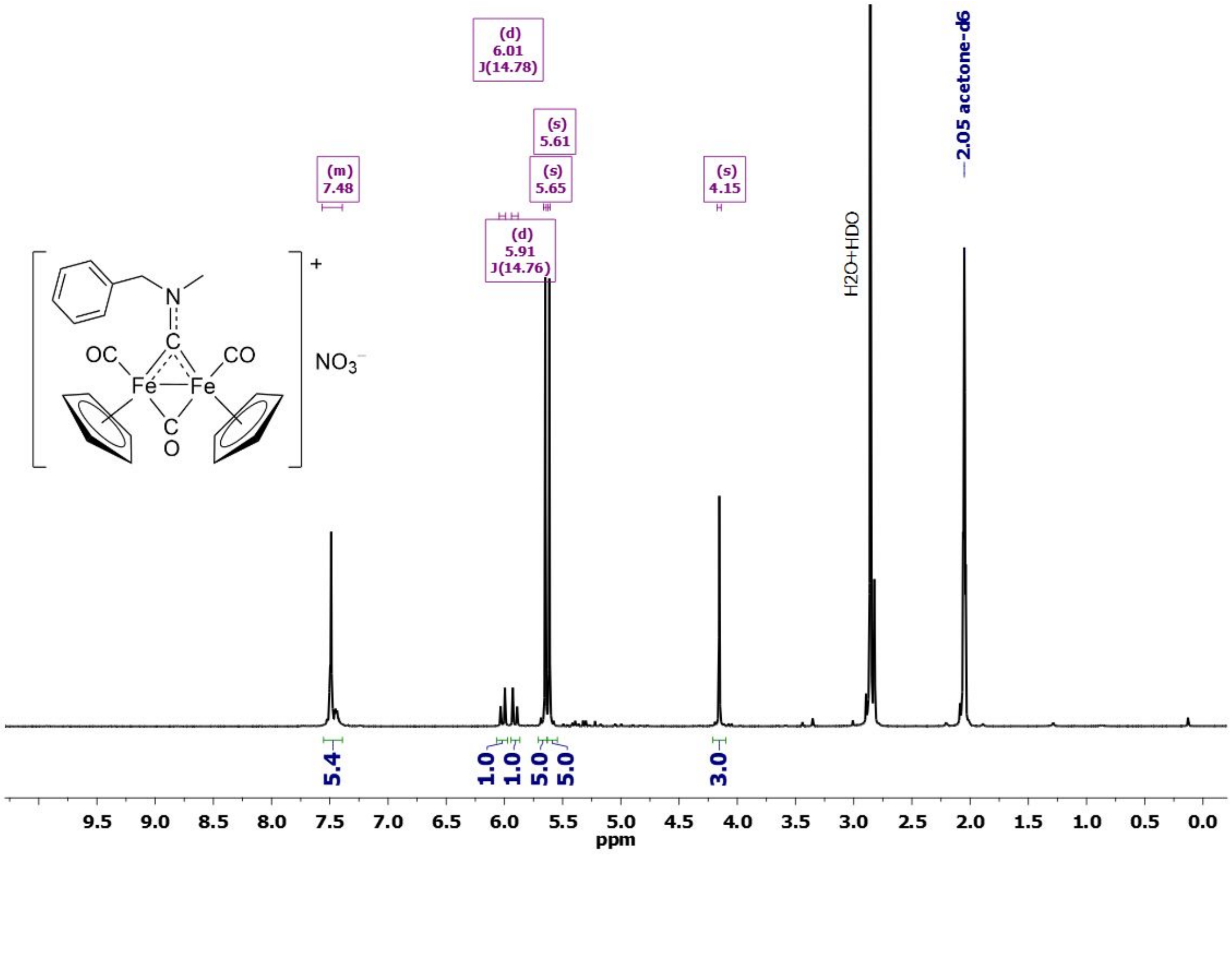

Figure S7. $^{13}C\{^{1}H\}$ NMR spectrum (126 MHz, acetone-d_6) of [**1c**]NO_3.

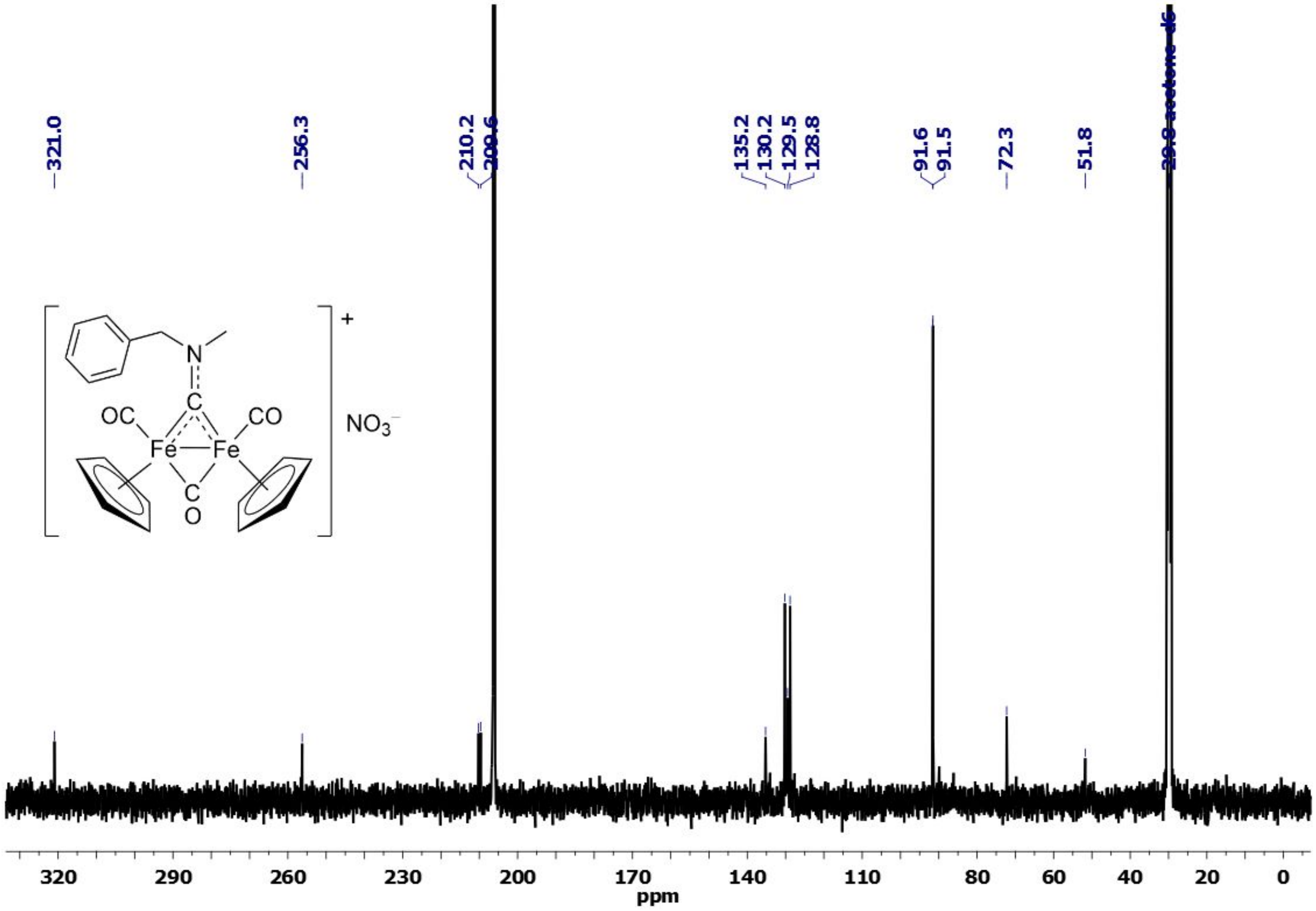

Figure S8. Solvent-subtracted IR spectrum (1300-2300 cm^{-1}) of [**1c**]NO_3 in CH_2Cl_2.

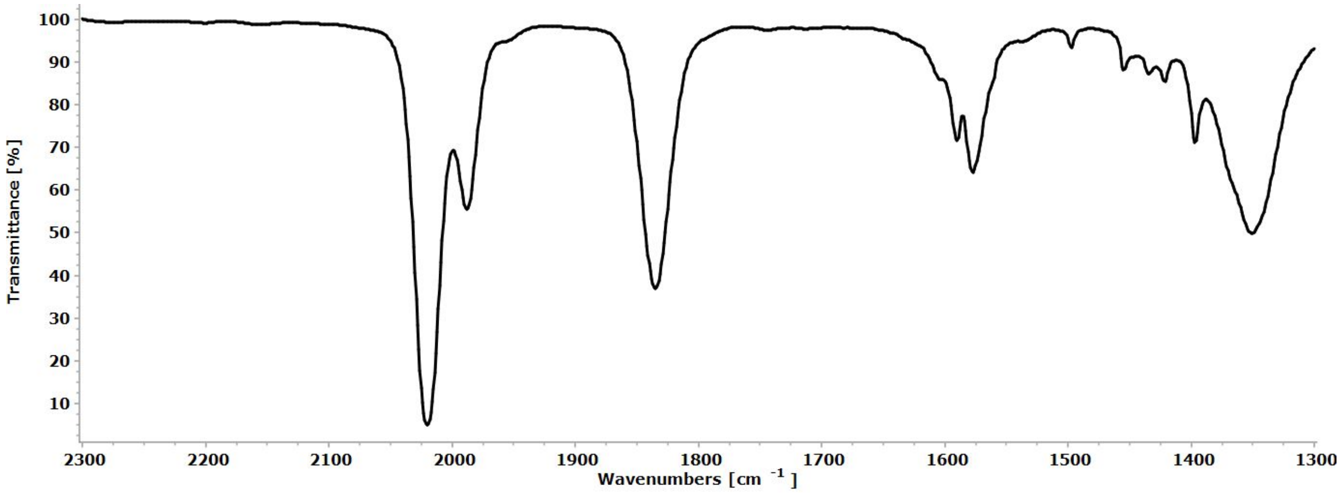

Figure S9. Solid-state IR spectrum (650-4000 cm^{-1}) of $[Fe_2Cp_2(CO)_2(\mu\text{-}CO)\{\mu\text{-}CNBn_2\}]NO_3$, [**1d**]$NO_3$.

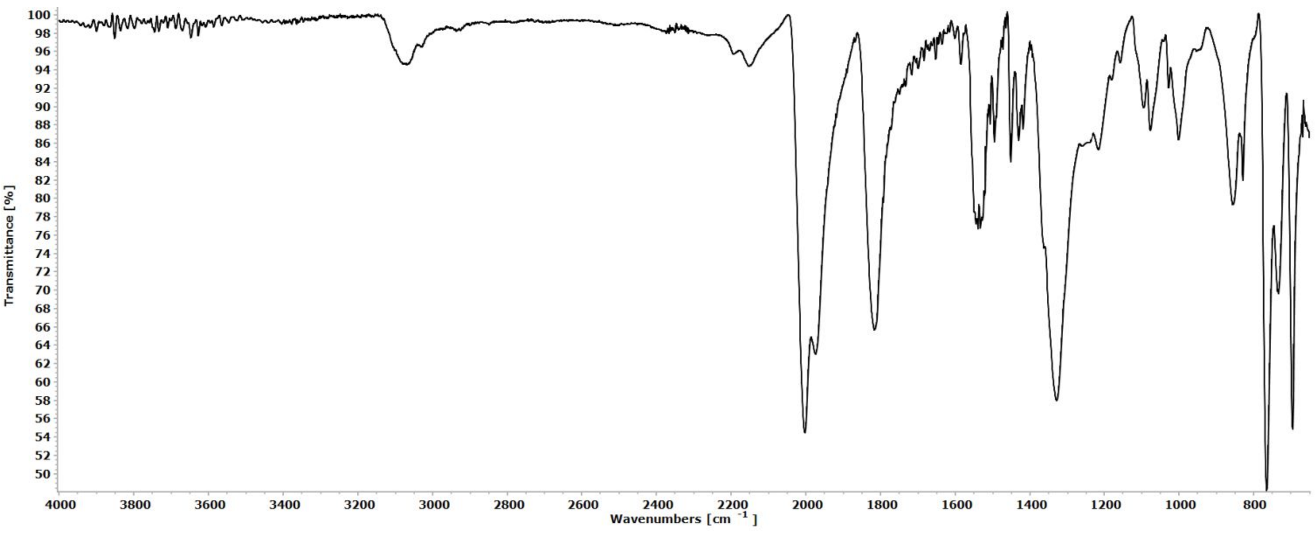

Figure S10. ^{1}H NMR spectrum (500 MHz, acetone-d$_6$) of [**1d**]NO_3.

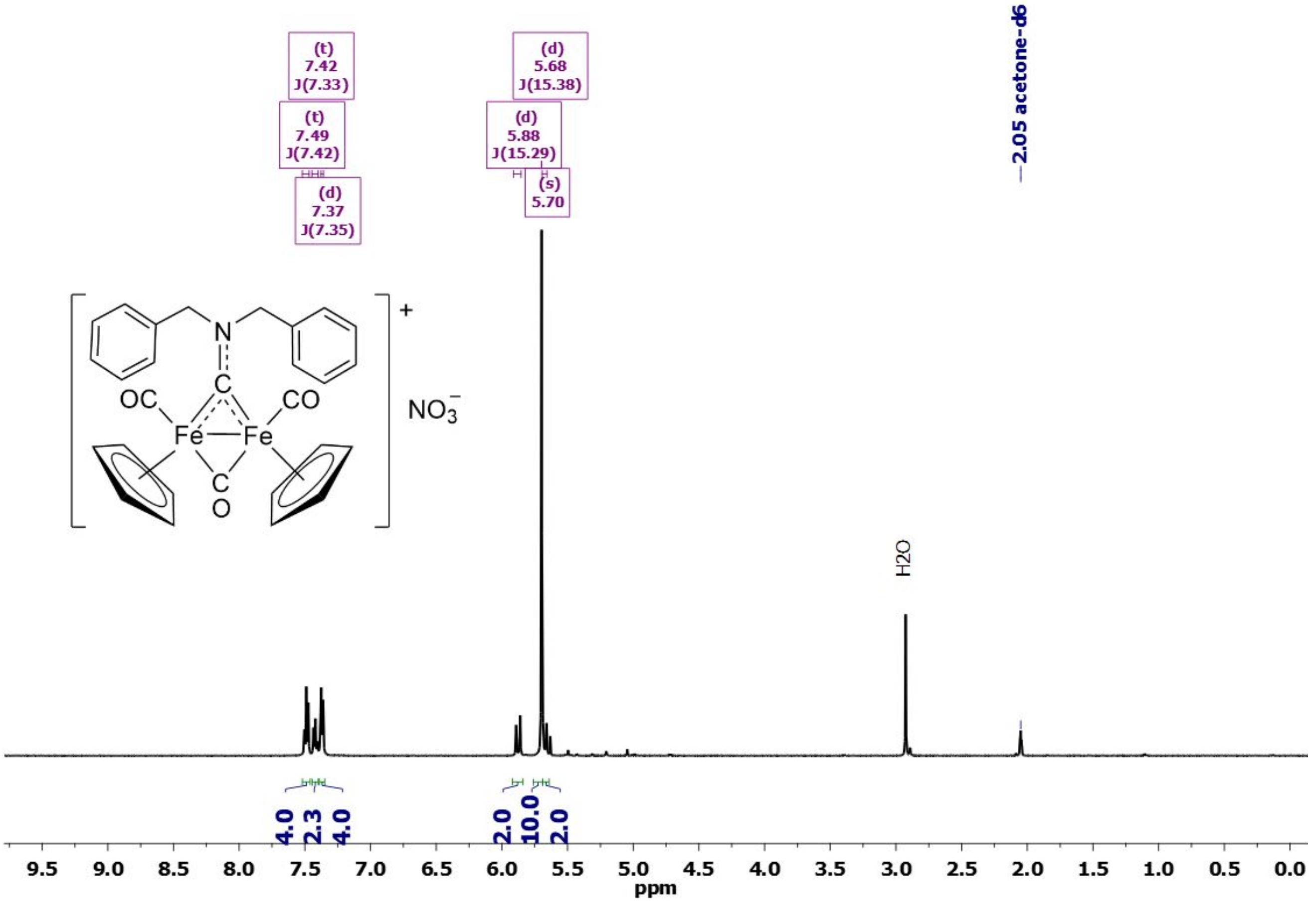

Figure S11. $^{13}C\{^1H\}$ NMR spectrum (126 MHz, acetone-d$_6$) of [**1d**]NO_3.

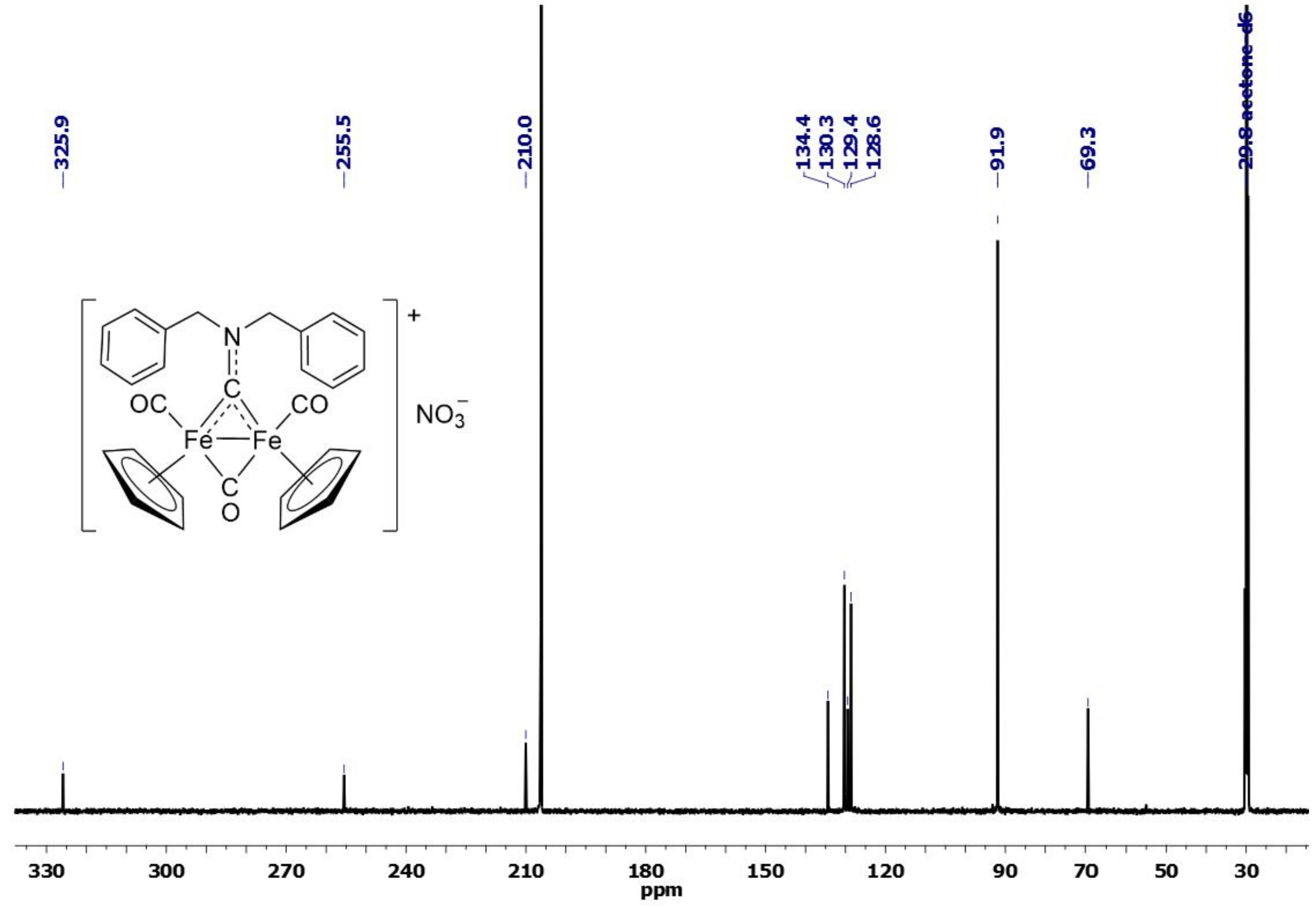

Figure S12. Solvent-subtracted IR spectrum (1300-2300 cm^{-1}) of [**1d**]NO_3 in CH_2Cl_2.

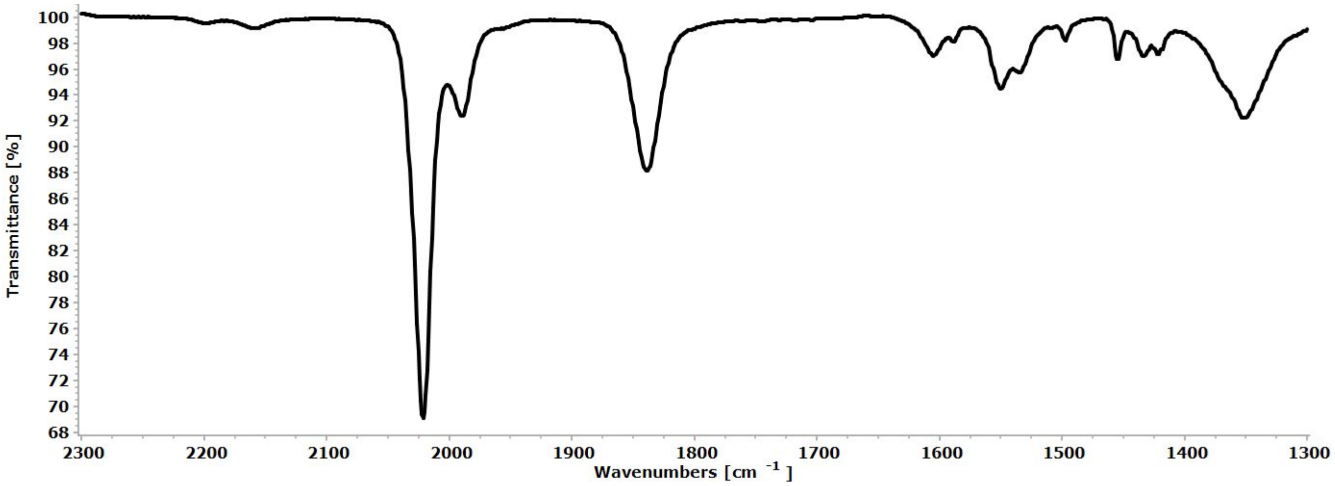

Figure S13. Solid-state IR spectrum (650-4000 cm^{-1}) of [$Fe_2Cp_2(CO)_2(\mu$-CO){μ-CNMe(Xyl)}]NO_3, [**1e**]NO_3.

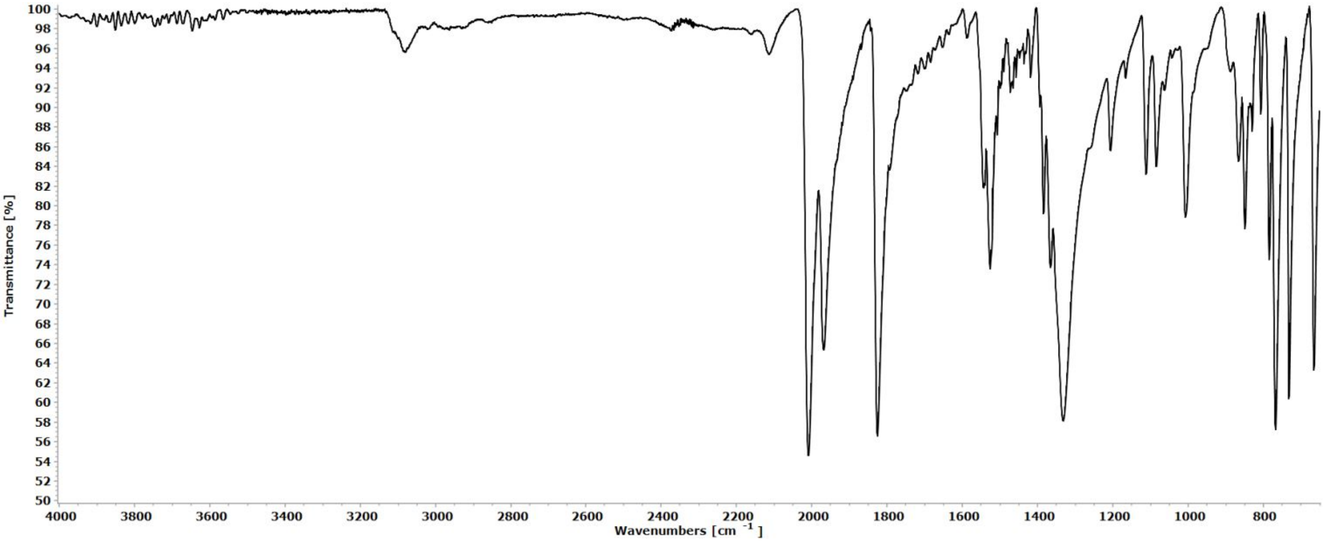

Figure S14. ^{1}H NMR spectrum (500 MHz, CD_3CN) of [**1e**]NO_3.

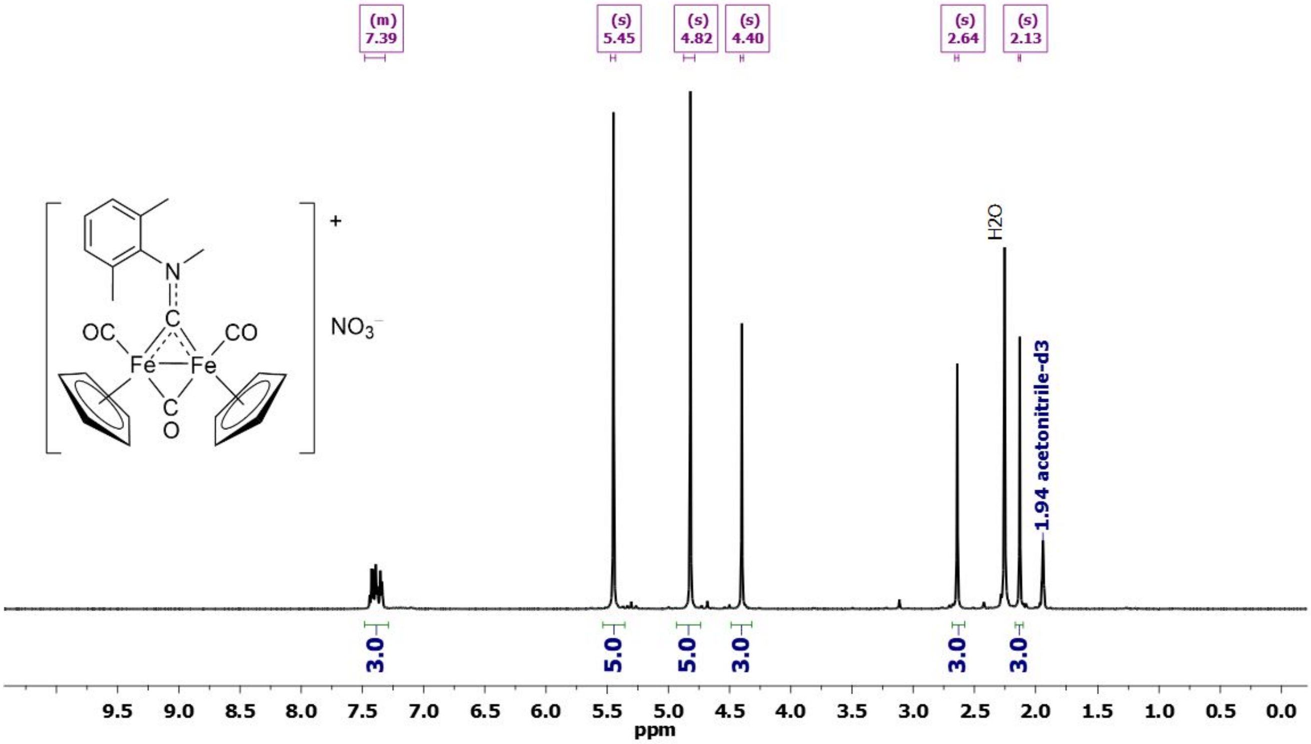

Figure S15. $^{13}C\{^{1}H\}$ NMR spectrum (126 MHz, CD_3CN) of [**1e**]NO_3.

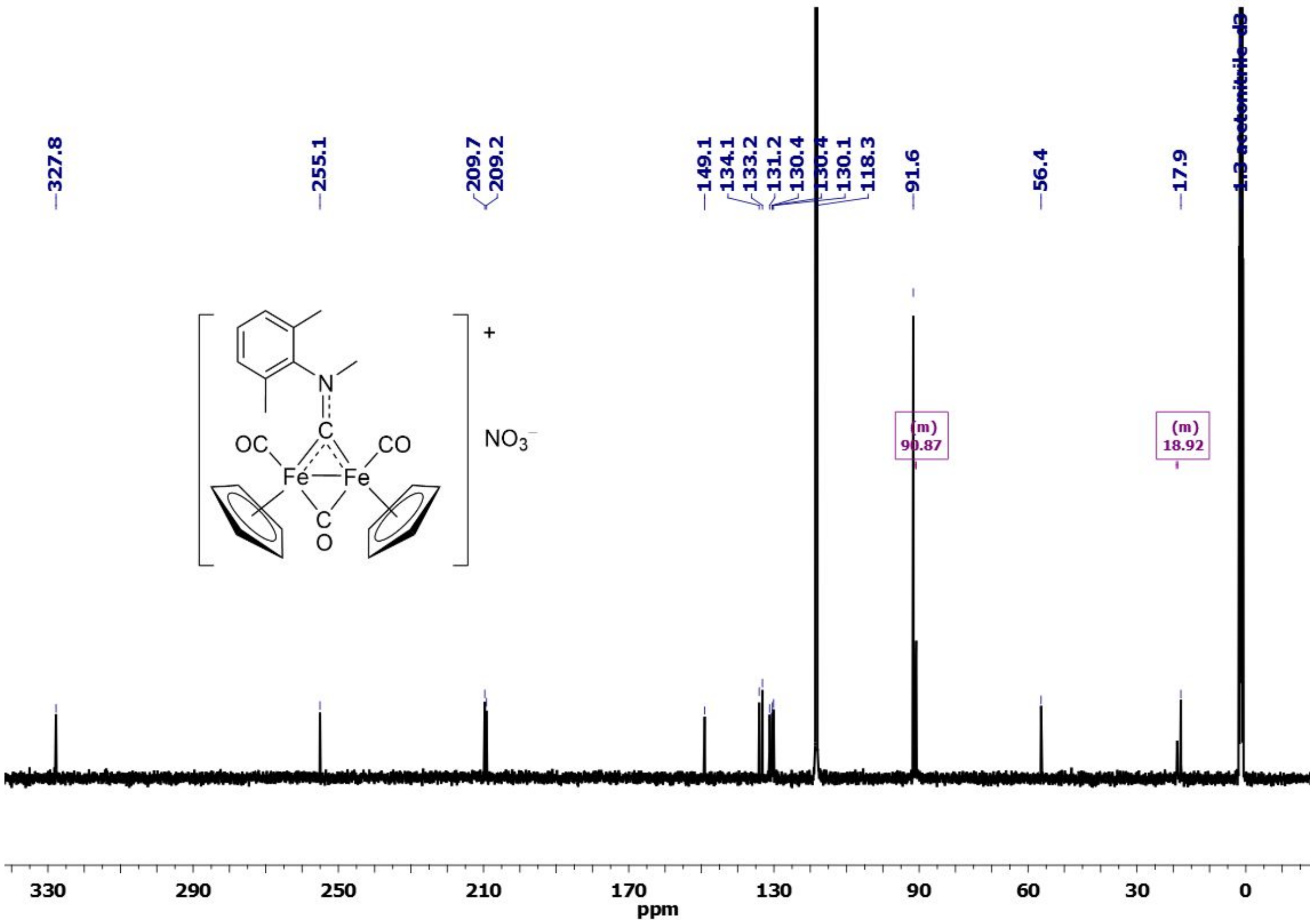

Figure S16. Solvent-subtracted IR spectrum (1300-2300 cm^{-1}) of [**1e**]NO_3 in CH_2Cl_2.

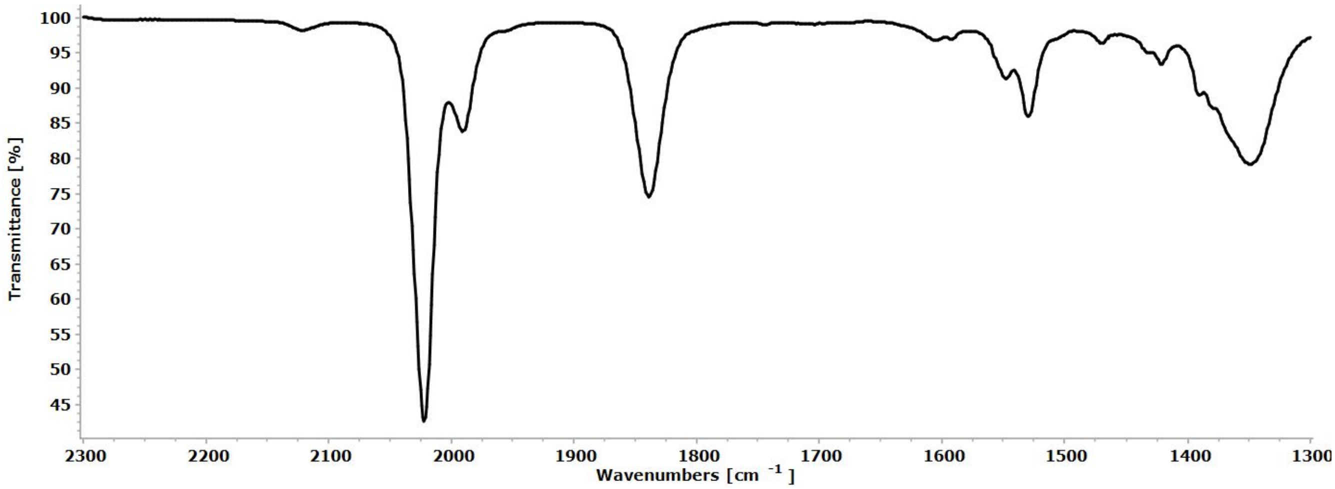

Spectroscopic characterization in aqueous solution

NMR data refer to the saturated solutions; ^{1}H NMR resonances are referenced to Me_2SO_2 (δ_H = 3.14 ppm). The chemical shifts of [**1**]$^+$ are unchanged across the concentration range explored and are coincident among nitrates and triflates (only those related to the former are listed). *Trans* isomers are not present unless otherwise specified. All UV-Vis spectra include absorptions around 284 nm (sh, $\varepsilon \approx 7$-$8 \cdot 10^3$ $M^{-1} \cdot cm^{-1}$), 415 nm (sh, $\varepsilon \approx 1.1$-$1.2 \cdot 10^3$ $M^{-1} \cdot cm^{-1}$) and 485 nm ($\varepsilon \approx 6 \cdot 10^2$ $M^{-1} \cdot cm^{-1}$) that are not listed below. Raman absorptions for [**1a**]NO_3 refer to Figure S50.

[1a]NO$_3$. ^{1}H NMR (D_2O): δ/ppm = 5.33, *5.22* (s, 10H, Cp); *4.27*, 4.19 (s, 6H, NMe_2); *cis*/*trans* isomer ratio = 25 (*ca*. 4 % *trans* isomer). UV-Vis (H_2O): λ_{max}/nm = 340 ($\varepsilon = 5.31 \cdot 10^3$ $M^{-1} \cdot cm^{-1}$). Raman (H_2O): $\tilde{\upsilon}$/cm^{-1} = 234, 361, 439, 1049, 1121, 1637br, 2028 (CO).

[1b]NO$_3$. ^{1}H NMR: δ/ppm = 5.35, 5.32 (s, 10H, Cp+Cp'); 4.05 (s, 3H, NMe); 2.33 (d, J = 12.3 Hz, 1H), 2.16-1.70 (m, 6H), 1.66-1.43 (m, 2H), 1.35-1.23 (m, 1H) (CH_2^{Cy}); the NCH^{Cy} resonance at *ca*. 4.8 ppm is hidden by HDO; a trace of *trans* isomer (ca. 3 %) is present. UV-Vis (H_2O): λ_{max}/nm = 340 (ε = $4.64 \cdot 10^3$ $M^{-1} \cdot cm^{-1}$).

[1c]NO$_3$. ^{1}H NMR: δ/ppm = 7.59–7.53 (m, 2H), 7.52–7.46 (m, 3H) (Ph); 5.85 (d, $^{2}J_{HH}$ = 15.2 Hz, 2H, NCH), 5.72 (d, $^{2}J_{HH}$ = 15.2 Hz, 2H, NCH'), 5.42 (s, 5H, Cp), 5.32 (s, 5H, Cp'), 4.07 (s, 3H, NCH_3).
UV-Vis (H_2O): λ_{max}/nm = 340 ($\varepsilon = 5.08 \cdot 10^3$ $M^{-1} \cdot cm^{-1}$).

[1d]NO$_3$. ^{1}H NMR (D_2O): δ/ppm = 7.58-7.43 (m, 6H), 7.37-7.25 (m, 4H) (Ph); 5.75 (d, $^{2}J_{HH}$ = 15 Hz, 2H, NCH); 5.63 (d, $^{2}J_{HH}$ = 15 Hz, 2H, NCH'), 5.40 (s, 10H, Cp). UV-Vis (H_2O): λ_{max}/nm = 340 (ε = $4.42 \cdot 10^3$ $M^{-1} \cdot cm^{-1}$).

[1e]NO$_3$. ^{1}H NMR: δ/ppm = 7.51–7.35 (m, 3H, C_6H_3), 5.49 (s, 5H, Cp), 4.88 (s, 5H, Cp'), 4.45 (s, 3H, NCH_3), 2.66 (s, 3H, CCH_3), 2.16 (s, 3H, CCH_3'). UV-Vis (H_2O): λ_{max}/nm = 340 ($\varepsilon = 4.75 \cdot 10^3$ $M^{-1} \cdot cm^{-1}$),

[1a]CF$_3$SO$_3$. ^{1}H NMR (D_2O): *cis*/*trans* isomer ratio *ca*. 9 in the saturated solution. UV-Vis (H_2O): λ_{max}/nm = 340 ($\varepsilon = 5.29\cdot10^3$ $M^{-1}\cdot cm^{-1}$).

***trans*-[1b]CF$_3$SO$_3$**. ^{1}H NMR: δ/ppm = 5.193, 5.189 (s, 10H, Cp+Cp'), 4.17 (s, 3H, NMe), 2.30 (d, J = 12.4 Hz, 1H); other resonances are hidden by HDO (NCH^{Cy}) or superimposed with the *cis* isomer (CH_2^{Cy}); *cis*/*trans* ratio = 2.4 in the saturated solution; unchanged after heating to 70 °C for *ca*. 4 h.

[1b]CF$_3$SO$_3$. UV-Vis (H_2O): λ_{max}/nm = 340 ($\varepsilon = 5.22\cdot10^3$ $M^{-1}\cdot cm^{-1}$).

***trans*-[1c]CF$_3$SO$_3$**. ^{1}H NMR: δ/ppm = 7.58–7.45 (Ph, superimposed with the *cis* isomer), 6.05 (d, $^2J_{HH}$ = 13.7 Hz, 2H, NCH), 5.68 (d, $^2J_{HH}$ = 13.5 Hz, 2H, NCH'), 5.28 (s, 5H, Cp), 5.20 (s, 5H, Cp'), 4.08 (s, 3H, NCH_3); *cis*/*trans* ratio = 2.5 in the saturated solution; 3.9 after heating to 70 °C for *ca*. 4 h.

[1c]CF$_3$SO$_3$. UV-Vis (H_2O): λ_{max}/nm = 340 ($\varepsilon = 4.87\cdot10^3$ $M^{-1}\cdot cm^{-1}$).

[1e]CF$_3$SO$_3$. UV-Vis (H_2O): λ_{max}/nm = 340 ($\varepsilon = 4.88\cdot10^3$ $M^{-1}\cdot cm^{-1}$).

Table S2. Solubility of diiron bis-cyclopentadienyl aminocarbyne complexes of general formula $[Fe_2Cp_2(CO)_3\{CN(R)(R')\}]^+$, as triflate or nitrate salts, in D_2O at room temperature (22 ± 2 °C).

Cation			Anion and solubility		NO_3^- *vs.* $CF_3SO_3^-$ solubility ratio
	R	R'	$CF_3SO_3^-$ [a]	NO_3^-	
**[1a]**$^+$	Me	Me	$6.27\cdot10^{-3}$ mol/L	$1.02\cdot10^{-1}$ mol/L	16
**[1b]**$^+$	Me	Cy	$3.72\cdot10^{-3}$ mol/L	$4.15\cdot10^{-2}$ mol/L	11
**[1c]**$^+$	Me	Bn	$1.82\cdot10^{-3}$ mol/L	$1.59\cdot10^{-2}$ mol/L	8.8
**[1d]**$^+$	Bn	Bn	$3.9\cdot10^{-4}$ mol/L	$4.29\cdot10^{-3}$ mol/L	11
**[1e]**$^+$	Me	Xyl	$2.86\cdot10^{-3}$ mol/L	$1.48\cdot10^{-2}$ mol/L	5.2

[a] Solubility values re-determined with respect to previously published data.

Figure S17. Solubility of diiron bis-cyclopentadienyl aminocarbyne complexes of general formula $[Fe_2Cp_2(CO)_3\{CN(R)(R')\}]^+$, as triflate or nitrate salts, in D_2O at room temperature (22 ± 2 °C). Bottom: Log plot.

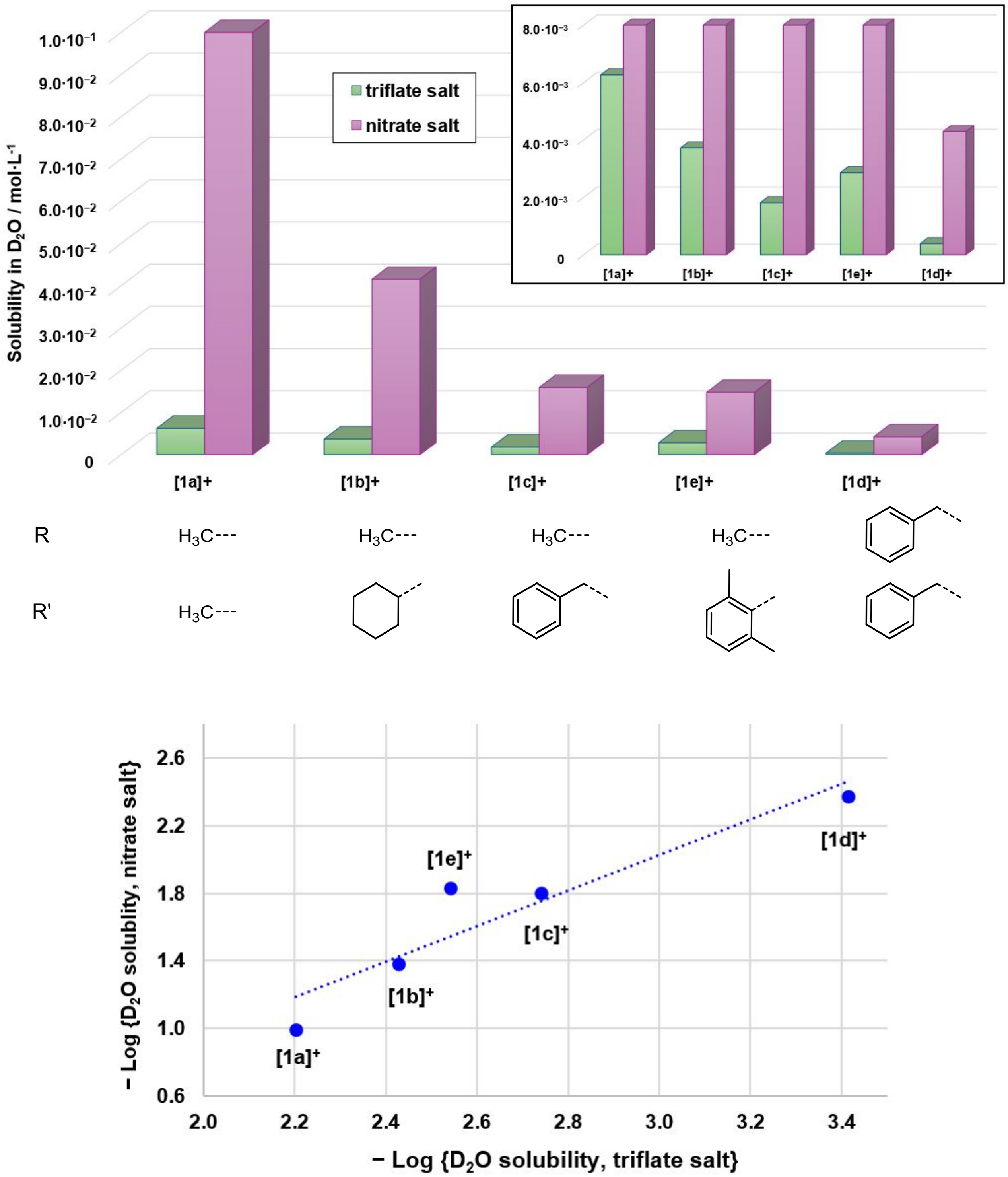

Optimization of parameters for ^{1}H NMR experiments

Figure S18. Relative integrals obtained from ^{1}H NMR spectra of D_2O solution containing DSS ($7.10 \cdot 10^{-2}$ mol/L; blue points), Me_2SO_2 ($7.57 \cdot 10^{-2}$ mol/L; green points) and [**1a**]NO_3 ($6.90 \cdot 10^{-2}$ mol/L; red points for the Cp ligands). Top image: freshly-prepared; bottom image: after 96 h at 37 °C. Spectra were acquired with a 45° pulse (filled points/continuous lines) or 90° pulse (hollow points/dotted lines) and different delay times (1-30 s). All values are normalized with respect to the fastest-relaxing NMe_2 resonance (brown crosses, I = 6).

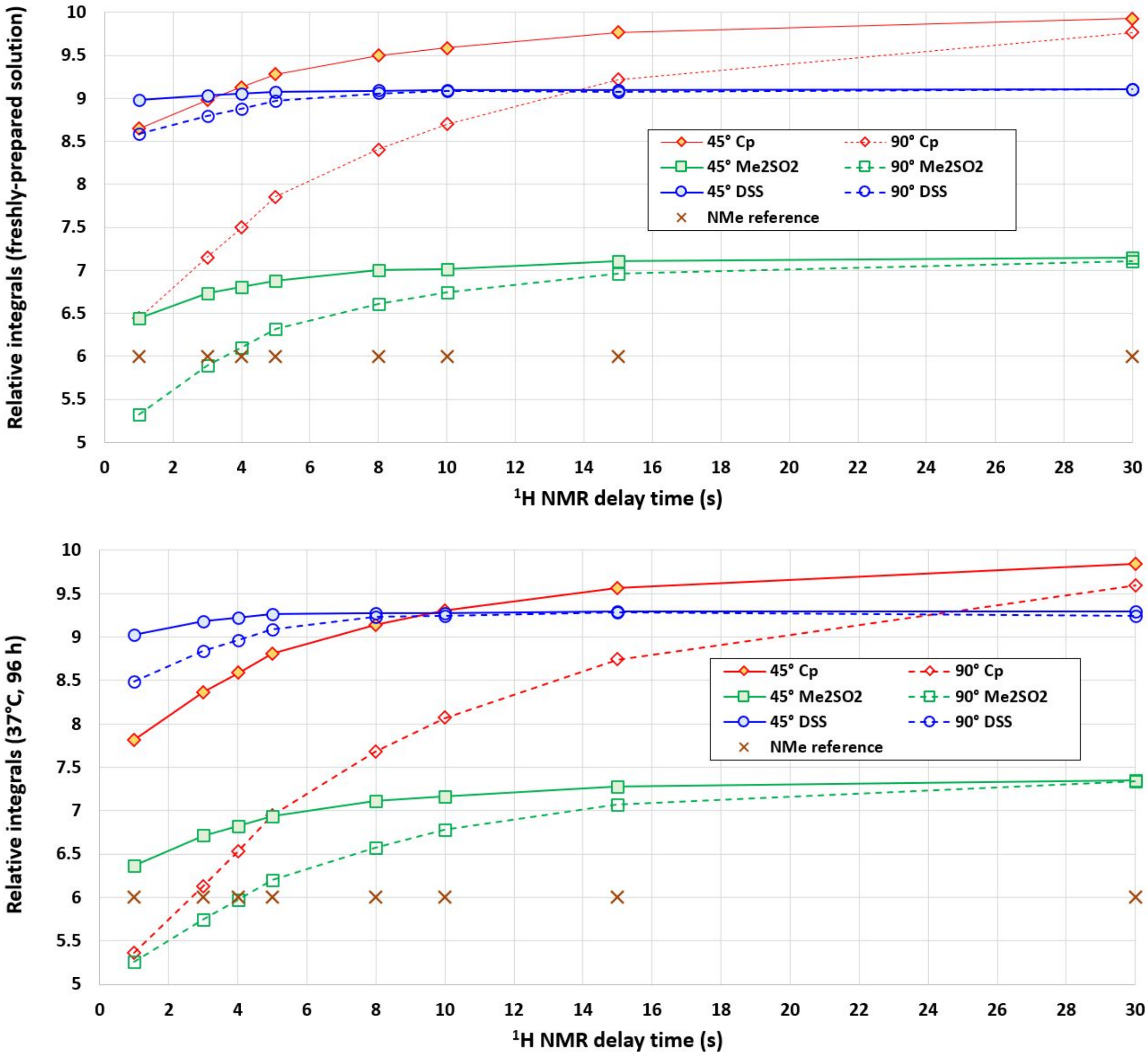

Figure S19. Relative % amount of [**1a**]$^+$ in solution after 96 h at 37 °C, with respect to the freshy-prepared solution, calculated by the relative integrals of NMe (triangles) or Cp (squares) resonances [%R = **I**(0)/**I**(96h)·100%], with respect to Me_2SO_2 or DSS, using different pulse angle (45° or 90°; colored or hollow points, respectively) and delay times (1-30 s). The dotted red line represents the "true value" calculated as the average %R of NMe and Cp resonances *vs*. DSS using 30 s delay time.

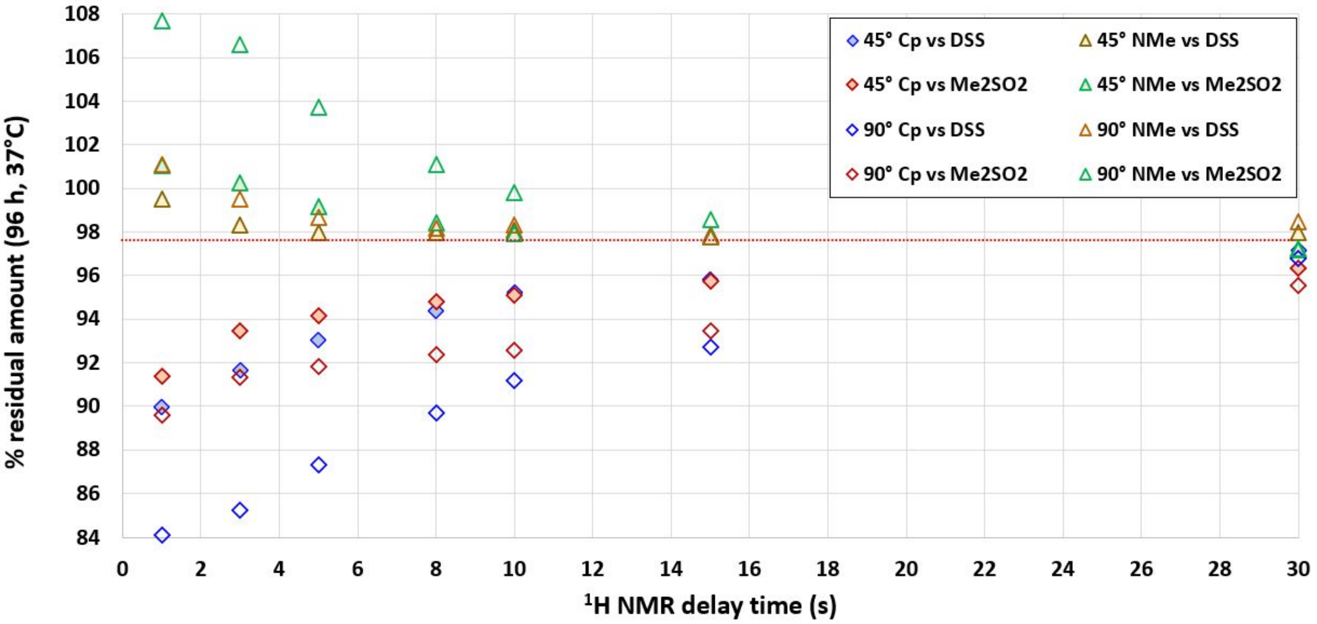

Figure S20. Error in the % residual amount of [**1a**]$^+$ with respect to the "true value" (average value calculated with the relative integrals of NMe and Cp resonances *vs*. DSS using 30 s delay time) - data in Figure S19.

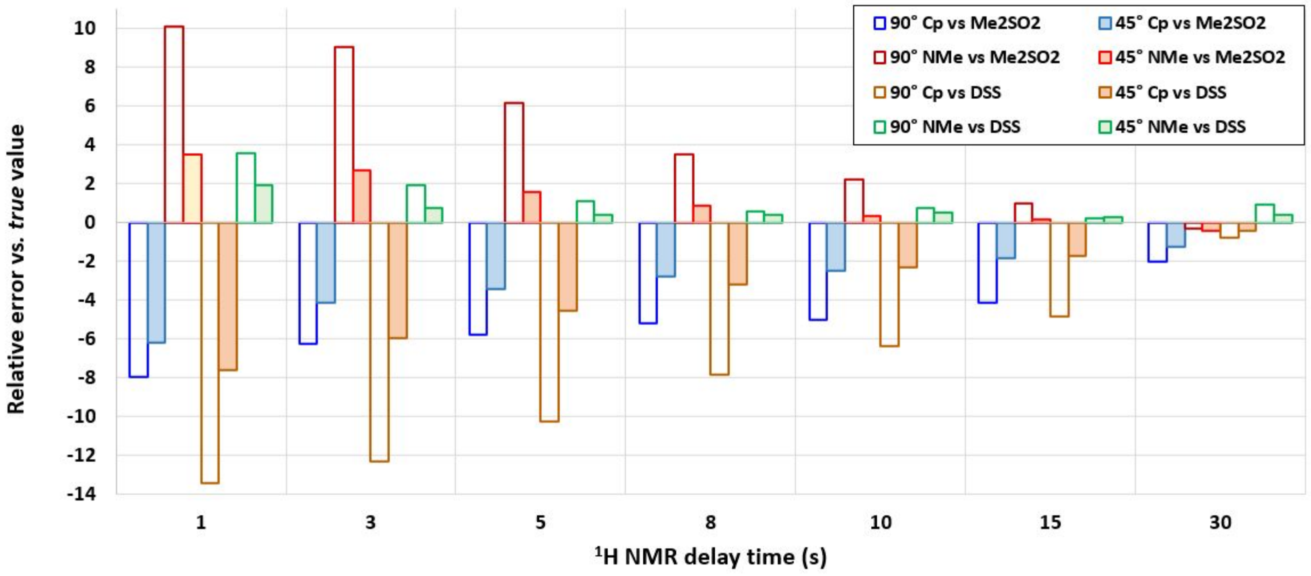

NMR and UV-Vis analyses of solutions of diiron compounds in water or DMEM

Figure S21. ^{1}H NMR spectra (401 MHz) of a freshly prepared solution of [**1a**]NO_3 in D_2O (top, dark blue line, c^0_{Fe2} = $5.38 \cdot 10^{-3}$ mol/L) and after 72 h at 37 °C (bottom, dark red line). Signals of the *trans* isomer are marked with asterisk (*).

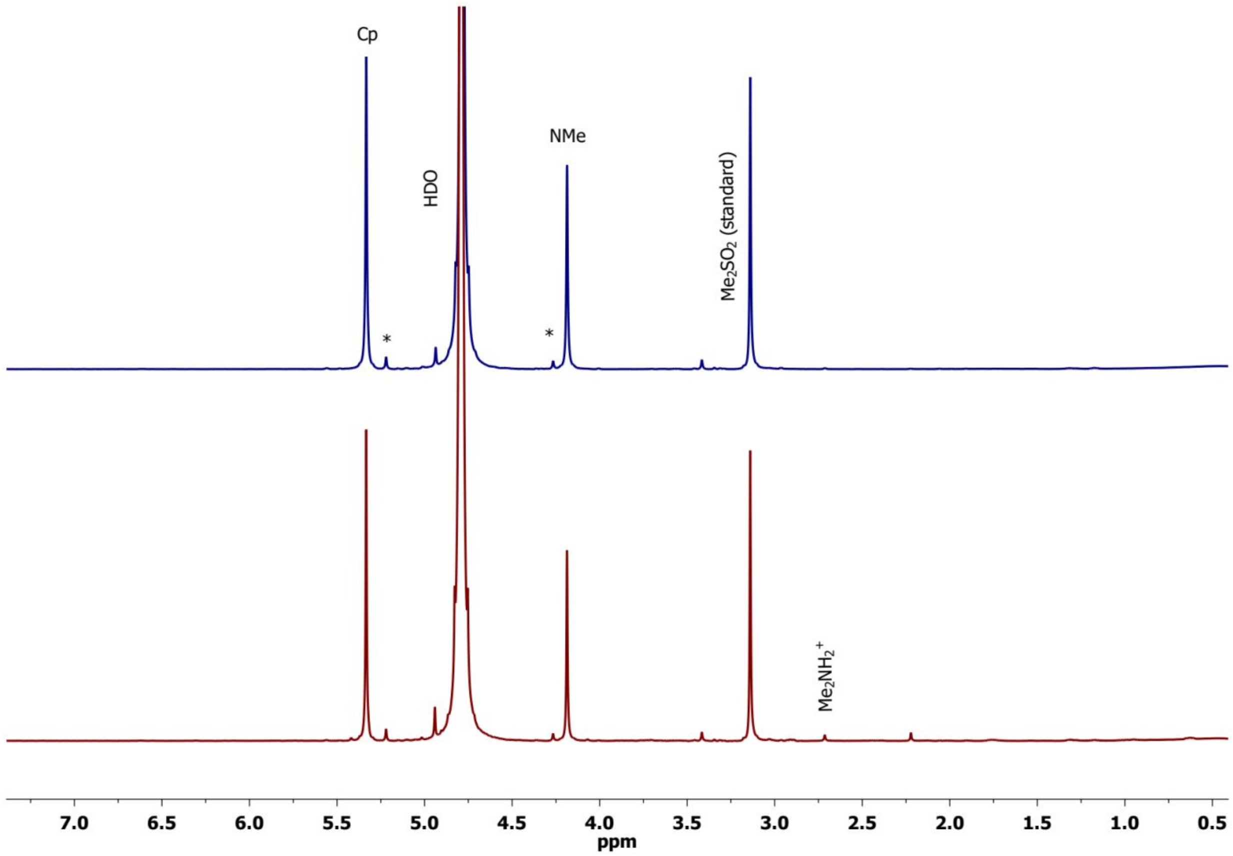

Figure S22. Blank-subtracted and baseline-corrected UV-Vis spectra (280-800 nm, 2 mm quartz cuvette) of [**1a**]NO_3 in water (c^0_{Fe2} = $1.07 \cdot 10^{-3}$ mol/L): freshly prepared solution (dark blue line) and three aliquots of the same after 72 h at 37 °C (red, brown and pink lines).

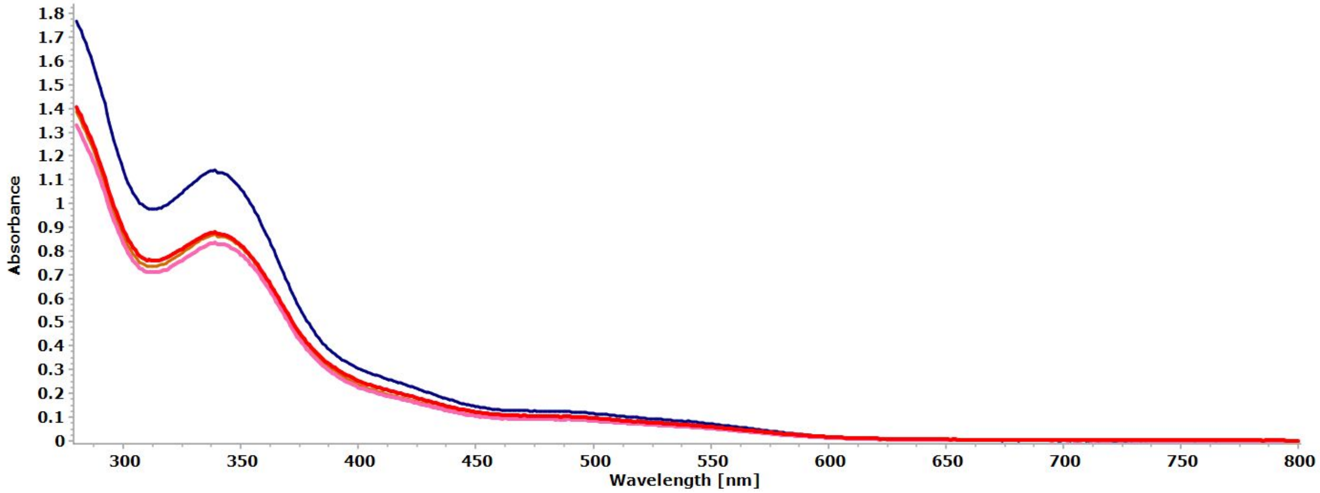

Figure S23. ^{1}H NMR spectra (401 MHz) of a freshly prepared solution of [**1b**]NO_3 in D_2O (top, dark blue line, c^0_{Fe2} = 2.71·10^{-3} mol/L) and after 72 h at 37 °C (bottom, dark red line).

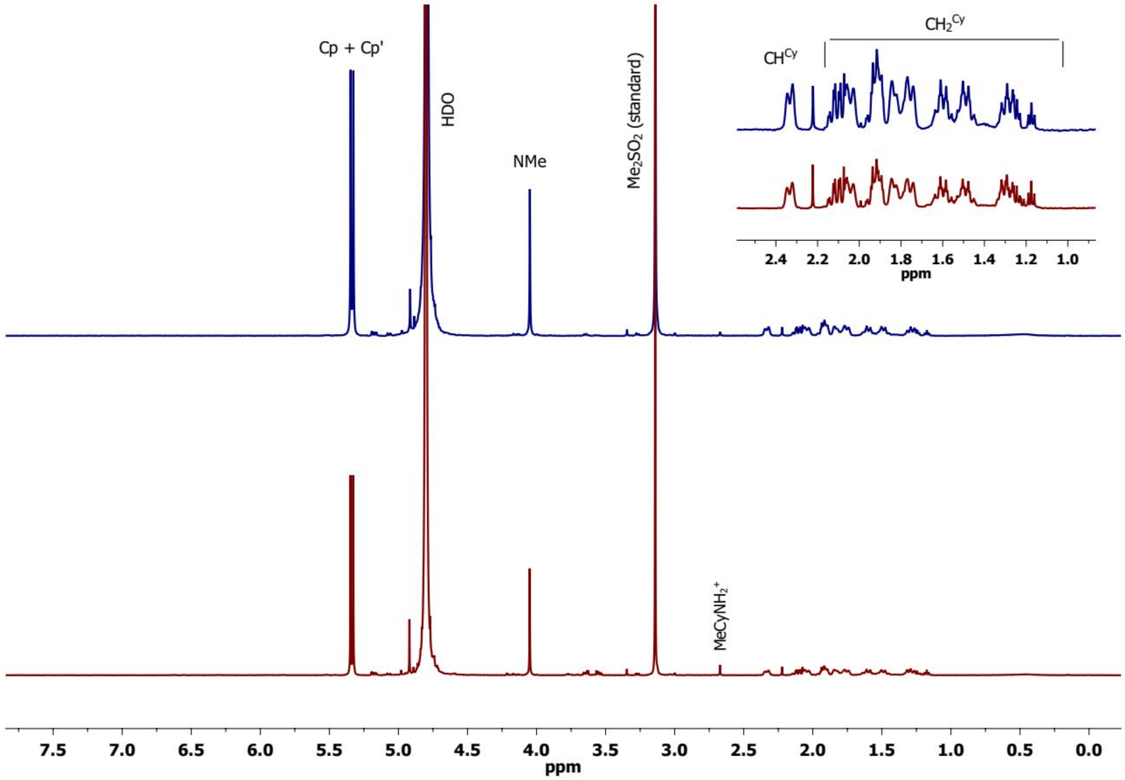

Figure S24. ^{1}H NMR spectra (401 MHz) of a freshly prepared solution of [**1c**]NO_3 in D_2O (top, dark blue line, c^0_{Fe2} = 3.93·10^{-3} mol/L) and after 72 h at 37 °C (bottom, dark red line).

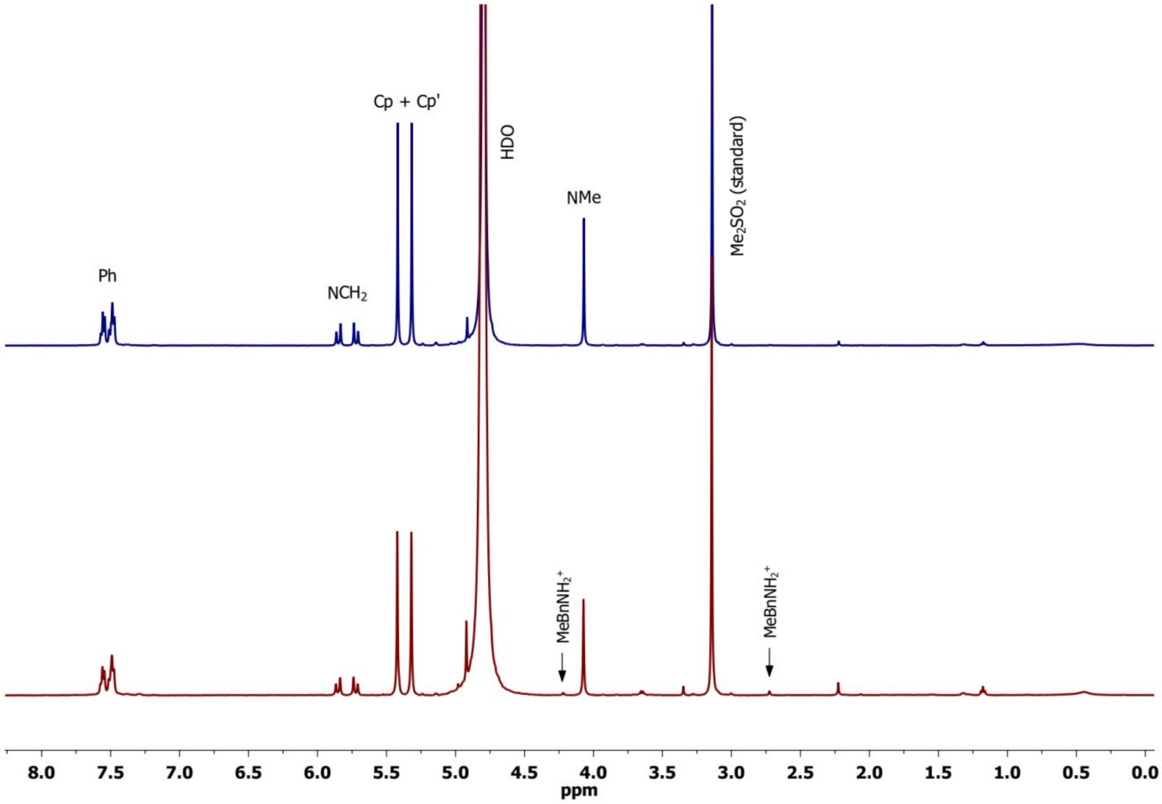

Figure S25. ^{1}H NMR spectra (401 MHz) of a freshly prepared solution of [**1d**]NO_3 in D_2O (top, dark blue line, c^0_{Fe2} = 4.01·10^{-3} mol/L) and after 72 h at 37 °C (bottom, dark red line).

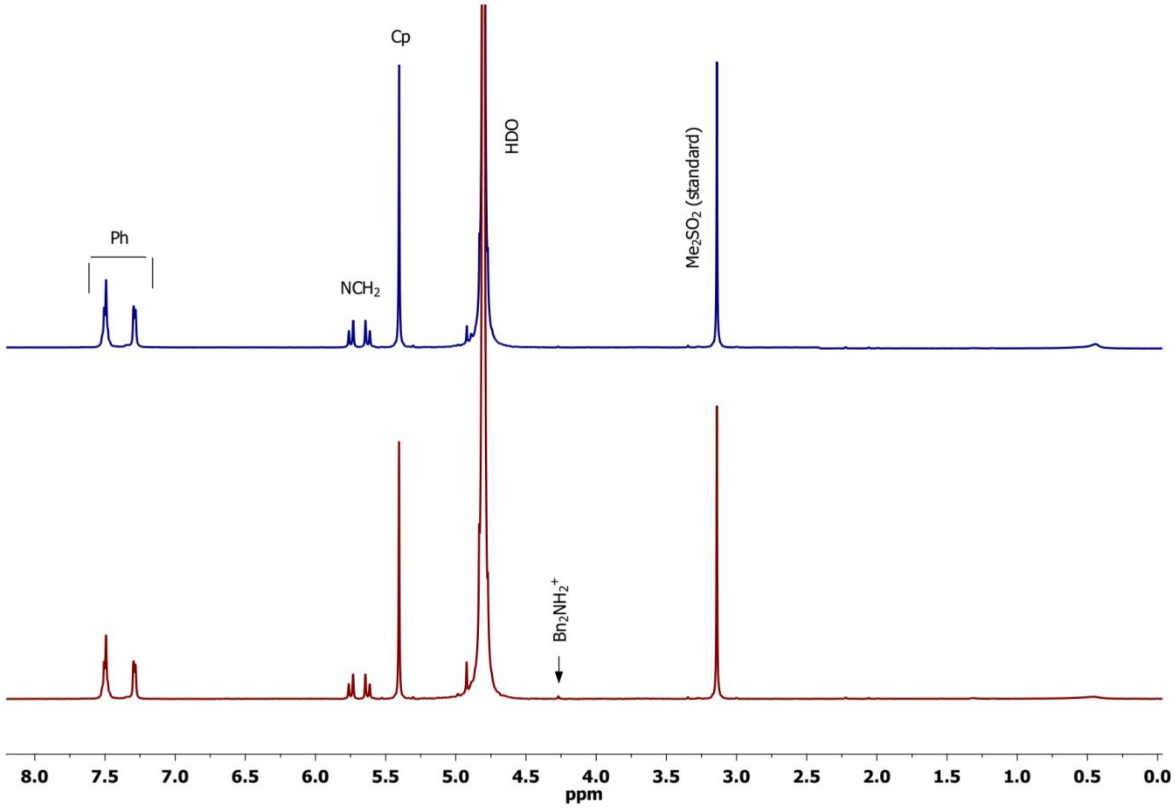

Figure S26. ^{1}H NMR spectra (401 MHz) of a freshly prepared solution of [**1e**]NO_3 in D_2O (top, dark blue line, c^0_{Fe2} = 5.91·10^{-3} mol/L) and after 72 h at 37 °C (bottom, dark red line).

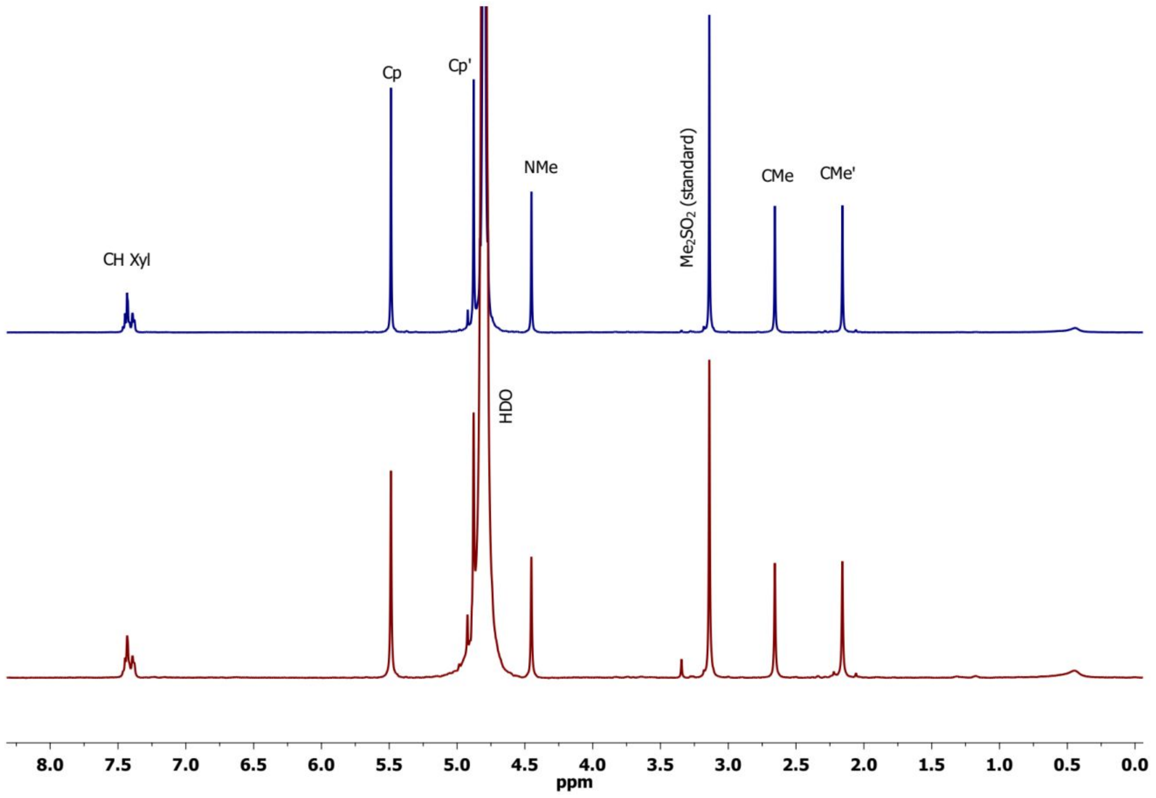

Table S3. ^{1}H NMR or UV-Vis analysis of solutions of [**1a**]$^+$ in water or cell culture medium kept at 37 °C for 72 h. All experiments were carried out without protection from ambient light/air.

Entry	Starting material	Solution, technique	Initial concentration [a]		compounds detected in the final solution [a] (% amount respect to the starting material)			Notes
			c^0_{Fe2} / mol·L^{-1}	pFe^0	[1a]$^+$ [b]	$Me_2NH_2^+$ (% *vs* consumed [**1a**]$^+$)	CpH (% *vs* consumed [**1a**]$^+$)	
S3-1	[**1a**]NO_3	D_2O, NMR	$6.90 \cdot 10^{-2}$	1.16	97.8	0.5 (23)	trace	
S3-2	[**1a**]NO_3	D_2O, NMR	$2.28 \cdot 10^{-2}$	1.64	97.0	0.2 (8)	< LOD	
S3-3	[**1a**]NO_3	D_2O, NMR	$2.25 \cdot 10^{-2}$	1.65	95.6	0.9 (20)	< LOD	[c]
S3-4	[**1a**]NO_3	D_2O, NMR	$2.13 \cdot 10^{-2}$	1.67	94.9	1.5 (30)	0.2 (4)	
S3-5	[**1a**]NO_3	D_2O, NMR	$1.00 \cdot 10^{-2}$	2.00	93.6	1.2 (19)	< LOD	
S3-6	[**1a**]NO_3	D_2O, NMR	$9.93 \cdot 10^{-3}$	2.00	93.7	1.3 (20)	0.3 (5)	
S3-7	[**1a**]CF_3SO_3	D_2O, NMR	$6.27 \cdot 10^{-3}$	2.20	86.1	8.1 (58)	trace	[d]
S3-8	[**1a**]NO_3	D_2O, NMR	$5.99 \cdot 10^{-3}$	2.22	89.6	0.9 (8)	< LOD	
S3-9	[**1a**]NO_3	D_2O, NMR	$5.38 \cdot 10^{-3}$	2.27	89.0	2.2 (20)	0.6 (6)	
S3-10	[**1a**]NO_3	D_2O, NMR	$5.13 \cdot 10^{-3}$	2.29	86.1	1.3 (9)	trace	
S3-11	[**1a**]NO_3	D_2O, NMR	$4.95 \cdot 10^{-3}$	2.31	88.9	2.4 (21)	0.8 (7)	
S3-12	[**1a**]CF_3SO_3	D_2O, NMR	$3.97 \cdot 10^{-3}$	2.40	82.6	6.9 (40)	trace	
S3-13	[**1a**]NO_3	D_2O, NMR	$3.05 \cdot 10^{-3}$	2.52	87.0	1.8 (13)	trace	
S3-14	[**1a**]NO_3	D_2O, NMR	$3.04 \cdot 10^{-3}$	2.52	83.5	3.9 (23)	< LOD	
S3-15	[**1a**]NO_3	D_2O, NMR	$2.48 \cdot 10^{-3}$	2.61	82.4	3.2 (18)	0.5 (3)	
S3-16	[**1a**]NO_3	D_2O, NMR	$2.40 \cdot 10^{-3}$	2.62	81.8	3.3 (18)	< LOD	
S3-17	[**1a**]NO_3	D_2O, NMR	$1.26 \cdot 10^{-3}$	2.90	74.2	3.5 (14)	trace	
S3-18	[**1a**]NO_3	H_2O, UV-Vis	$1.07 \cdot 10^{-3}$	2.97	75.6 ± 1.4	/	/	
S3-19	[**1a**]NO_3	D_2O, NMR	$9.85 \cdot 10^{-4}$	3.01	71.1	6.3 (22)	trace	
S3-20	[**1a**]NO_3	H_2O, UV-Vis	$8.40 \cdot 10^{-4}$	3.08	72.5 ± 5.0	/	/	
S3-21	[**1a**]CF_3SO_3	H_2O, UV-Vis	$5.52 \cdot 10^{-4}$	3.26	70.4 ± 6.5	/	/	
S3-22	[**1a**]NO_3	H_2O, UV-Vis	$4.95 \cdot 10^{-4}$	3.31	68.7 ± 2.1	/	/	
S3-23	[**1a**]NO_3	H_2O, UV-Vis	$2.74 \cdot 10^{-4}$	3.56	63.6 ± 0.4	/	/	
S3-24	[**1a**]CF_3SO_3	H_2O, UV-Vis	$2.44 \cdot 10^{-4}$	3.61	69.0 ± 2.1	/	/	
S3-25	[**1a**]NO_3	H_2O, UV-Vis	$1.57 \cdot 10^{-4}$	3.80	58.0 ± 4.4	/	/	
S3-26	[**1a**]NO_3	H_2O, UV-Vis	$1.37 \cdot 10^{-4}$	3.86	56.8 ± 3.7	/	/	
S3-27	[**1a**]NO_3	DMEM-d, NMR	$2.01 \cdot 10^{-2}$	1.70	90.2	2.3 (*24*)	0.7 (*7*)	
S3-28	[**1a**]NO_3	DMEM-d, NMR	$1.99 \cdot 10^{-2}$	1.70	90.4	1.5 (*15*)	0.8 (*8*)	
S3-29	[**1a**]NO_3	DMEM-d, NMR	$1.43 \cdot 10^{-2}$	1.85	88.5	2.7 (*24*)	1.0 (*9*)	
S3-30	[**1a**]NO_3	DMEM-d, NMR	$9.94 \cdot 10^{-3}$	2.00	83.8	2.8 (*18*)	1.4 (*8*)	
S3-31	[**1a**]NO_3	DMEM-d, NMR	$9.48 \cdot 10^{-3}$	2.02	82.9	4.3 (*25*)	1.9 (*11*)	
S3-32	[**1a**]NO_3	DMEM-d, NMR	$6.98 \cdot 10^{-3}$	2.16	79.8	3.7 (*18*)	1.7 (*9*)	
S3-33	[**1a**]NO_3	DMEM-d, NMR	$5.53 \cdot 10^{-3}$	2.26	79.1	5.6 (*27*)	2.3 (*11*)	

S3-34	[**1a**]NO_3	DMEM-d, NMR	$5.48{\cdot}10^{-3}$	2.26	77.3	5.4 (*24*)	1.7 (*7*)
S3-35	[**1a**]CF_3SO_3	DMEM-d, NMR	$4.36{\cdot}10^{-3}$	2.36	72.8	4.9 (*18*)	2.9 (*11*)
S3-36	[**1a**]CF_3SO_3	DMEM-d, NMR	$3.27{\cdot}10^{-3}$	2.49	74.8	6.0 (*24*)	3.0 (*12*)
S3-37	[**1a**]NO_3	DMEM-d, NMR	$2.22{\cdot}10^{-3}$	2.65	74.4	6.3 (*24*)	2.5 (*10*)
S3-38	[**1a**]CF_3SO_3	DMEM-d, NMR	$1.02{\cdot}10^{-3}$	2.99	63.3	12.9 (*35*)	< LOD

[a] ^{1}H NMR experiments: the initial concentration and % relative amount of compounds with respect to the freshly prepared solution were calculated using Me_2SO_2 or DSS as internal standard. UV-Vis experiments: the initial concentration was calculated from mass and volume data (volumetric solutions) or from the molar absorbance at 340 nm of the freshly-prepared solution; the % residual amount of starting material was calculated by the relative decrease of the UV-Vis peak at 340 nm (see main text for details). Data expressed with 2 or 1 decimal digits to avoid excessive rounding. [b] Including *cis* and *trans* isomers. [c] 90.2 % of [**1a**]$^+$ after 6 days at 37 °C. [d] Corresponding to a saturated solution at room temperature.

Figure S27. % Residual amount of [**1a**]$^+$ after 72 h at 37 °C *vs.* decreasing initial molar concentration (logarithmic scale, − Log c^0_{Fe2} = pFe^0) in water or cell culture medium solution. Data refer to Table S3. Dotted lines represent a linear fitting of the data.

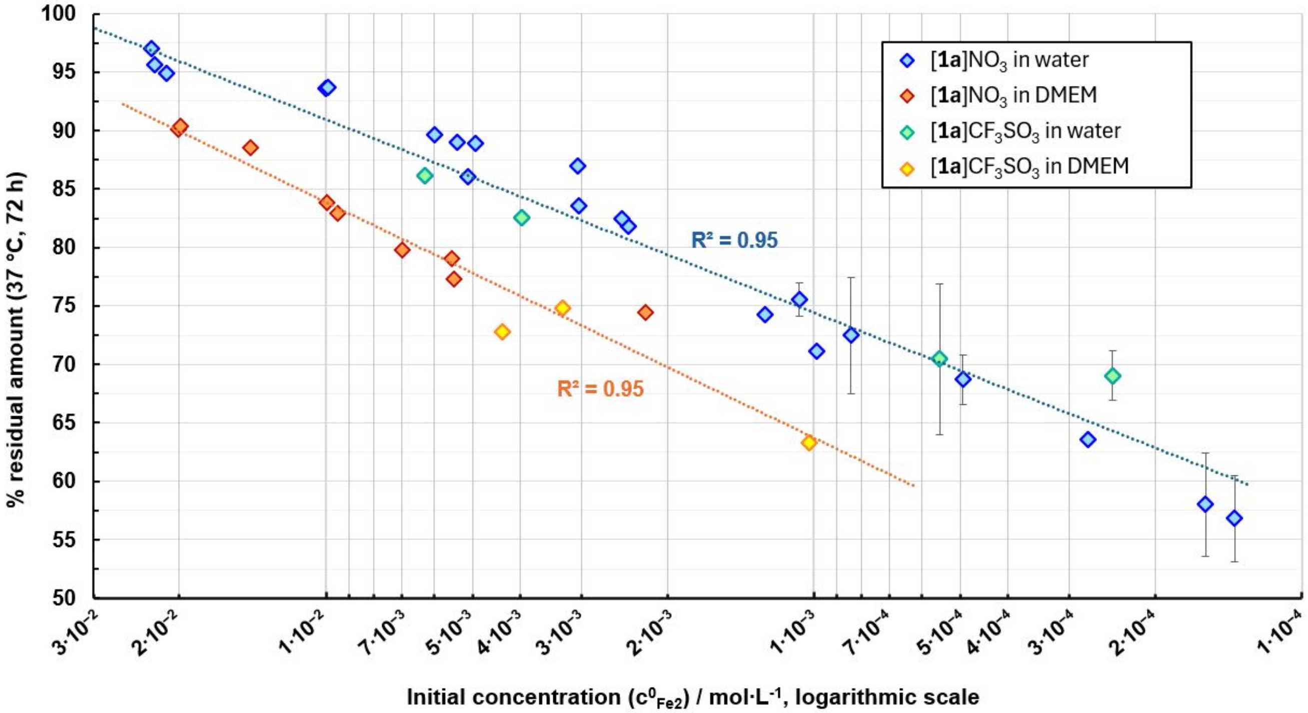

Table S4. ^{1}H NMR or UV-Vis analysis of solutions of [**1b**]$^+$ in water or cell culture medium kept at 37 °C for 72 h. All experiments were carried out without protection from ambient light/air.

Entry	Starting material	Solution, technique	Initial concentration [a]		compounds detected in the final solution [a] (% amount respect to the starting material)		
			c^0_{Fe2} / mol·L^{-1}	pFe^0	[1b]$^+$ [b]	CyMeNH$_2^+$ (% *vs* consumed [**1b**]$^+$)	CpH (% *vs* consumed [**1b**]$^+$)
S4-1	[**1b**]NO$_3$	D$_2$O, NMR	1.43·10^{-2}	1.84	92.9	4.5 (*63*)	0.8 (*12*)
S4-2	[**1b**]NO$_3$	D$_2$O, NMR	9.13·10^{-3}	2.04	90.7	3.2 (*35*)	1.7 (*18*)
S4-3	[**1b**]NO$_3$	D$_2$O, NMR	5.27·10^{-3}	2.28	90.7	2.8 (30)	< LOD
S4-4	[**1b**]NO$_3$	D$_2$O, NMR	4.82·10^{-3}	2.32	88.9	4.3 (39)	0.8 (*7*)
S4-5	[**1b**]NO$_3$	D$_2$O, NMR	2.71·10^{-3}	2.57	86.6	5.0 (37)	2.1 (*16*)
S4-6	[**1b**]NO$_3$	D$_2$O, NMR	2.55·10^{-3}	2.59	79.8	12.2 (60)	1.5 (*8*)
S4-7	[**1b**]NO$_3$	D$_2$O, NMR	2.53·10^{-3}	2.60	83.5	4.1 (25)	1.0 (*6*)
S4-8	[**1b**]CF$_3$SO$_3$	D$_2$O, NMR	2.10·10^{-3}	2.68	78.0	14.1 (64)	2.5 (*12*)
S4-9	[**1b**]NO$_3$	H$_2$O, UV-Vis	9.58·10^{-4}	3.02	69.5 ± 0.7	/	/
S4-10	[**1b**]CF$_3$SO$_3$	H$_2$O, UV-Vis	6.16·10^{-4}	3.21	73.9 ± 1.1	/	/
S4-11	[**1b**]NO$_3$	H$_2$O, UV-Vis	5.49·10^{-4}	3.26	73.4 ± 2.0	/	/
S4-12	[**1b**]CF$_3$SO$_3$	H$_2$O, UV-Vis	3.01·10^{-4}	3.52	66.2 ± 3.9	/	/
S4-13	[**1b**]NO$_3$	H$_2$O, UV-Vis	1.96·10^{-4}	3.71	59.6 ± 3.9	/	/
S4-14	[**1b**]NO$_3$	H$_2$O, UV-Vis	1.69·10^{-4}	3.78	55.4 ± 5.0	/	/
S4-15	[**1b**]CF$_3$SO$_3$	H$_2$O, UV-Vis	1.67·10^{-4}	3.78	57.7 ± 4.1	/	/
S4-16	[**1b**]NO$_3$	DMEM-d, NMR	1.38·10^{-2}	1.86	90.2	2.5 (*25*)	1.0 (*10*)
S4-17	[**1b**]NO$_3$	DMEM-d, NMR	5.28·10^{-3}	2.28	82.9	4.8 (*28*)	1.4 (*8*)
S4-18	[**1b**]NO$_3$	DMEM-d, NMR	4.49·10^{-3}	2.35	81.5	6.1 (*33*)	2.8 (*15*)
S4-19	[**1b**]NO$_3$	DMEM-d, NMR	4.09·10^{-3}	2.39	75.6	9.2 (*38*)	2.1 (*9*)
S4-20	[**1b**]NO$_3$	DMEM-d, NMR	2.56·10^{-3}	2.59	76.9	7.6 (*33*)	2.7 (*12*)
S4-21	[**1b**]CF$_3$SO$_3$	DMEM-d, NMR	2.51·10^{-3}	2.60	75.1	8.2 (*33*)	4.3 (*17*)
S4-22	[**1b**]CF$_3$SO$_3$	DMEM-d, NMR	2.07·10^{-3}	2.68	70.7	10.2 (*35*)	1.4 (*5*)
S4-23	[**1b**]CF$_3$SO$_3$	DMEM-d, NMR	1.91·10^{-3}	2.72	69.0	10.1 (*32*)	1.7 (*5*)
S4-24	[**1b**]NO$_3$	DMEM-d, NMR	9.85·10^{-4}	3.01	55.9	12.2 (*28*)	7.7 (*17*)

[a] ^{1}H NMR experiments: the initial concentration and % relative amount of compounds with respect to the freshly prepared solution were calculated using Me_2SO_2 or DSS as internal standard. UV-Vis experiments: the initial concentration was calculated from mass and volume data (volumetric solutions) or from the molar absorbance at 340 nm of the freshly-prepared solution; the % residual amount of starting material was calculated by the relative decrease of the UV-Vis peak at 340 nm (see main text for details). Data expressed with 2 or 1 decimal digits to avoid excessive rounding. [b] For [**1b**]CF_3SO_3: including *cis* and *trans* isomers.

Figure S28. % Residual amount of [**1b**]$^+$ after 72 h at 37 °C *vs.* decreasing initial molar concentration (logarithmic scale, − Log c^0_{Fe2} = pFe^0) in water or cell culture medium solution. Data refer to Table S4. Dotted lines represent a linear fitting of the data.

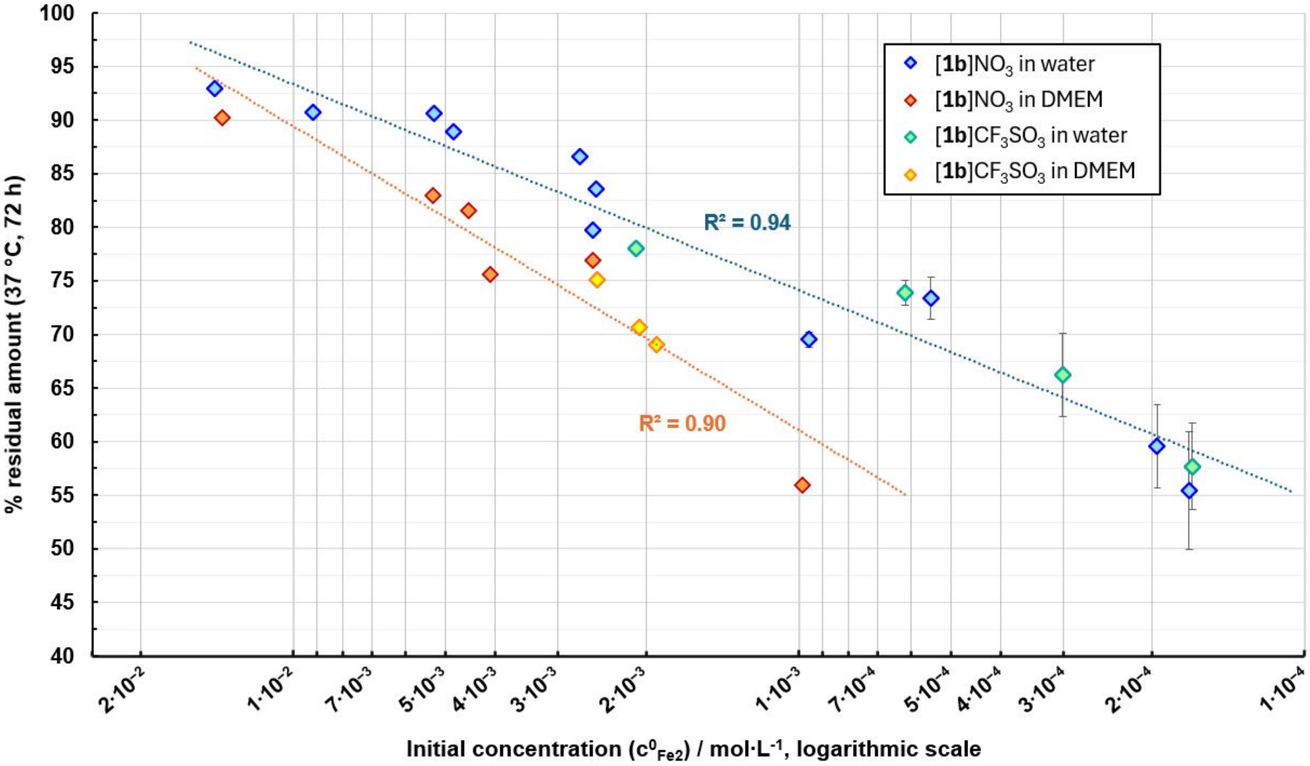

Table S5. ^{1}H NMR or UV-Vis analysis of solutions of [**1c**]$^+$ in water or cell culture medium kept at 37 °C for 72 h. All experiments were carried out without protection from ambient light/air.

Entry	Starting material	Solution, technique	Initial concentration [a]		compounds detected in the final solution [a] (% amount respect to the starting material)			Notes
			c^0_{Fe2} / $mol \cdot L^{-1}$	pFe^0	[1c]$^+$ [b]	$BnMeNH_2^+$ (% *vs* consumed [**1c**]$^+$)	CpH (% *vs* consumed [**1c**]$^+$)	
S5-1	[**1c**]NO_3	D_2O, NMR	$1.18 \cdot 10^{-2}$	2.03	93.7	2.2 (*35*)	0.6 (*10*)	
S5-2	[**1c**]NO_3	D_2O, NMR	$9.24 \cdot 10^{-3}$	2.23	87.8	3.6 (*29*)	0.5 (*4*)	
S5-3	[**1c**]NO_3	D_2O, NMR	$5.82 \cdot 10^{-3}$	2.33	90.2	0.82 (*8*)	< LOD	
S5-4	[**1c**]NO_3	D_2O, NMR	$4.70 \cdot 10^{-3}$	2.34	84.2	2.5 (*16*)[c]	< LOD	4.7 % $BnNH_3^+$
S5-5	[**1c**]NO_3	D_2O, NMR	$4.58 \cdot 10^{-3}$	2.41	82.5	3.9 (*22*)	< LOD	
S5-6	[**1c**]NO_3	D_2O, NMR	$3.93 \cdot 10^{-3}$	2.52	85.6	3.6 (*25*)	< LOD	
S5-7	[**1c**]NO_3	D_2O, NMR	$3.05 \cdot 10^{-3}$	2.53	79.8	7.6 (*37*)	1.4 (*7*)	
S5-8	[**1c**]NO_3	D_2O, NMR	$2.95 \cdot 10^{-3}$	3.06	84.8	4.9 (*32*)	1.1 (*7*)	
S5-9	[**1c**]NO_3	H_2O, UV-Vis	$8.64 \cdot 10^{-4}$	3.30	75.7 ± 0.2	/	/	
S5-10	[**1c**]NO_3	D_2O, NMR	$5.01 \cdot 10^{-4}$	3.36	66.0 ± 3.1	/	/	
S5-11	[**1c**]NO_3	H_2O, UV-Vis	$4.32 \cdot 10^{-4}$	3.40	65.7 ± 2.4	/	/	
S5-12	[**1c**]CF_3SO_3	H_2O, UV-Vis	$3.95 \cdot 10^{-4}$	3.76	66.3 ± 5.9	/	/	
S5-13	[**1c**]CF_3SO_3	H_2O, UV-Vis	$1.74 \cdot 10^{-4}$	3.90	64.1 ± 7.5	/	/	
S5-14	[**1c**]NO_3	H_2O, UV-Vis	$1.27 \cdot 10^{-4}$	1.93	55.9 ± 3.5	/	/	
S5-15	[**1c**]NO_3	DMEM-d, NMR	$1.31 \cdot 10^{-2}$	1.88	88.2	3.4 (*29*)	2.0 (*17*)	
S5-16	[**1c**]NO_3	DMEM-d, NMR	$1.12 \cdot 10^{-2}$	1.95	85.9	3.3 (*24*)	2.1 (*15*)	
S5-17	[**1c**]NO_3	DMEM-d, NMR	$6.40 \cdot 10^{-3}$	2.19	79.7	4.8 (*24*)	*trace*	
S5-18	[**1c**]NO_3	DMEM-d, NMR	$5.29 \cdot 10^{-3}$	2.28	80.6	3.1 (*16*)	1.1 (*6*)	
S5-19	[**1c**]NO_3	DMEM-d, NMR	$3.77 \cdot 10^{-3}$	2.42	70.6	7.1 (*24*)	2.7 (*9*)	
S5-20	[**1c**]NO_3	DMEM-d, NMR	$2.46 \cdot 10^{-3}$	2.61	71.2	4.6 (*16*)	< LOD	
S5-21	[**1c**]CF_3SO_3	DMEM-d, NMR	$1.86 \cdot 10^{-3}$	2.73	63.4	11.2 (*31*)	4.5 (*12*)	[d]
S5-22	[**1c**]CF_3SO_3	DMEM-d, NMR	$9.16 \cdot 10^{-4}$	3.04	49.1	15.2 (*30*)	1.3 (*3*)	

[a] ^{1}H NMR experiments: the initial concentration and % relative amount of compounds with respect to the freshly prepared solution were calculated using Me_2SO_2 or DSS as internal standard. UV-Vis experiments: the initial concentration was calculated from mass and volume data (volumetric solutions) or from the molar absorbance at 340 nm of the freshly-prepared solution; the % residual amount of starting material was calculated by the relative decrease of the UV-Vis peak at 340 nm (see main text for details). Data expressed with 2 or 1 decimal digits to avoid excessive rounding. [b] For [**1c**]CF_3SO_3: including *cis* and *trans* isomers. [c] Including $BnNH_3^+$. [d] Corresponding to a saturated solution at room temperature.

Figure S29. % Residual amount of [**1c**]$^+$ after 72 h at 37 °C *vs.* decreasing initial molar concentration (logarithmic scale, − Log c^0_{Fe2} = pFe^0) in water or cell culture medium solution. Data refer to Table S5. Dotted lines represent a linear fitting of the data.

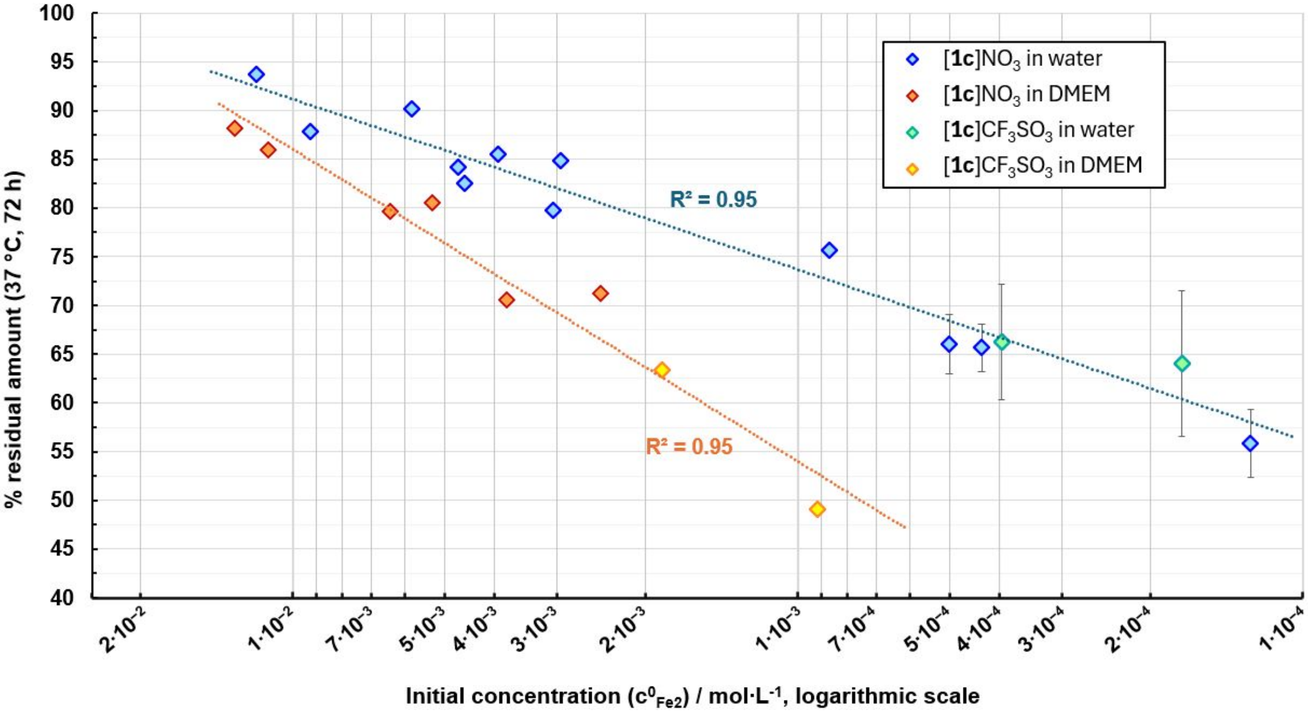

Table S6. ^1H NMR or UV-Vis analysis of aqueous solutions of [**1d**]NO_3 kept at 37 °C for 72 h. All experiments were carried out without protection from ambient light/air.

Entry	Solution, technique	Initial concentration [a]		compounds detected in the final solution [a] (% amount respect to the starting material)		
		c^0_{Fe2} / mol·L^{-1}	pFe^0	[**1d**]$^+$	$Bn_2NH_2^+$ (% *vs* consumed [**1d**]$^+$)	CpH (% *vs* consumed [**1d**]$^+$)
S6-1	D_2O, NMR	$4.01 \cdot 10^{-3}$	2.40	85.5	2.1 (*15*)	1.1 (*8*)
S6-2	D_2O, NMR	$3.68 \cdot 10^{-3}$	2.43	81.9	3.5 (*19*)	< LOD
S6-3	D_2O, NMR	$1.67 \cdot 10^{-3}$	2.78	79.8	5.1 (*25*)	1.9 (*9*)
S6-4	D_2O, NMR	$1.38 \cdot 10^{-3}$	2.86	74.1	6.5 (*25*)	2.1 (*8*)
S6-5	H_2O, UV-Vis	$1.03 \cdot 10^{-3}$	2.99	70.3 ± 4.5	/	/
S6-6	H_2O, UV-Vis	$5.25 \cdot 10^{-4}$	3.28	66.2 ± 4.4	/	/
S6-7	H_2O, UV-Vis	$1.47 \cdot 10^{-4}$	3.83	62.3 ± 3.4	/	/

[a] ^1H NMR experiments: the initial concentration and % relative amount of compounds with respect to the freshly prepared solution were calculated using Me_2SO_2 or DSS as internal standard. UV-Vis experiments: the initial concentration was calculated from mass and volume data (volumetric solutions) or from the molar absorbance at 340 nm of the freshly-prepared solution; the % residual amount of starting material was calculated by the relative decrease of the UV-Vis peak at 340 nm (see main text for details). Data expressed with 2 or 1 decimal digits to avoid excessive rounding.

Figure S30. % Residual amount of [**1d**]$^+$ after 72 h at 37 °C *vs.* decreasing initial molar concentration of the aqueous solution (logarithmic scale, − Log c^0_{Fe2} = pFe^0). Data refer to Table S6. The dotted line represents a linear fitting of the data.

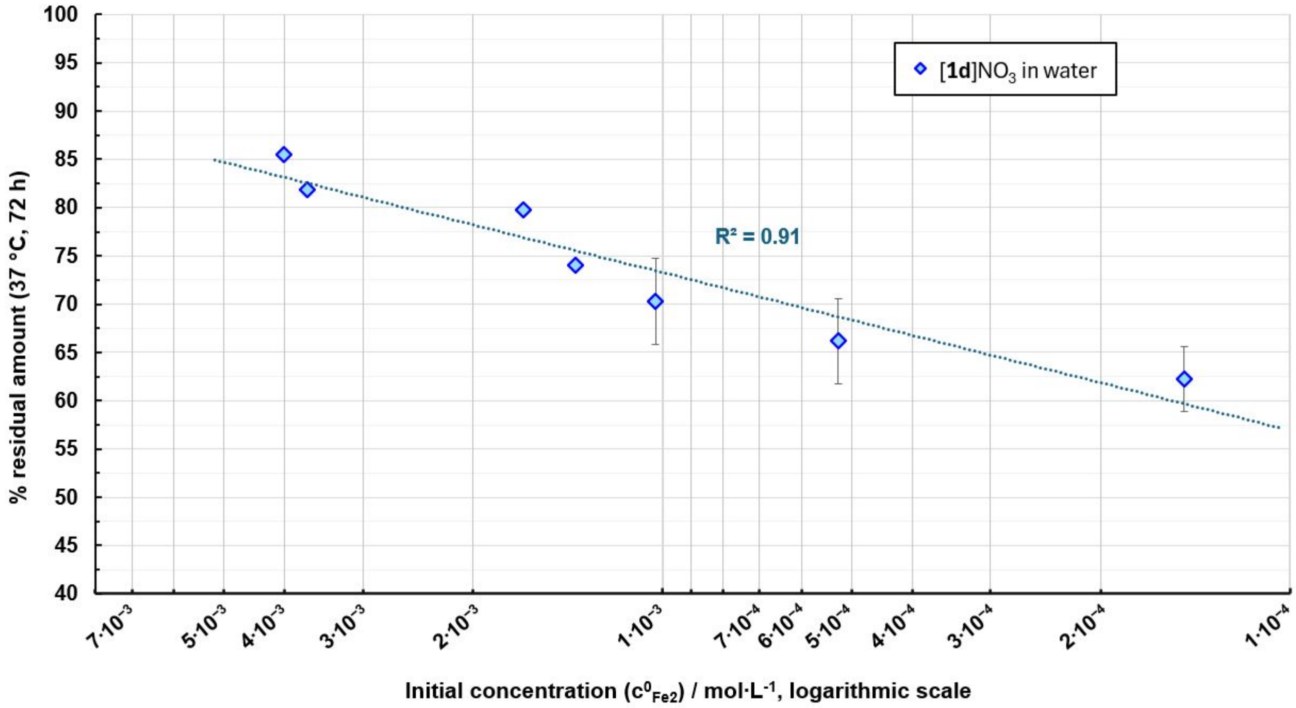

Table S7. ^{1}H NMR or UV-Vis analysis of solutions of [**1e**]$^+$ in water or cell culture medium kept at 37 °C for 72 h. All experiments were carried out without protection from ambient light/air.

Entry	Starting material	Solution, technique	Initial concentration [a]		compounds detected in the final solution [a] (% amount respect to the starting material)			Notes
			c^0_{Fe2} / mol·L^{-1}	pFe^0	[1e]$^+$	XylMeNH (% *vs* consumed [**1e**]$^+$)	CpH	
S7-1	[**1e**]NO$_3$	D$_2$O, NMR	9.86·10^{-3}	2.01	92.2	0.5 (41)[c]	0.5	XylNH$_2$ 3 %
S7-2	[**1e**]NO$_3$	D$_2$O, NMR	5.91·10^{-3}	2.23	88.1	0.31 (*3*)	< LOD	
S7-3	[**1e**]NO$_3$	D$_2$O, NMR	3.80·10^{-3}	2.42	86.2	0.2 (*1*)	< LOD	
S7-4	[**1e**]NO$_3$	D$_2$O, NMR	3.70·10^{-3}	2.43	80.1	6.0 (*30*)	< LOD	
S7-5	[**1e**]NO$_3$	D$_2$O, NMR	2.95·10^{-3}	2.53	79.8	3.1 (*15*)	trace	
S7-6	[**1e**]CF$_3$SO$_3$	D$_2$O, NMR	2.90·10^{-3}	2.54	82.6	trace	< LOD	
S7-7	[**1e**]NO$_3$	D$_2$O, NMR	2.72·10^{-3}	2.57	80.7	trace	0.9	
S7-8	[**1e**]CF$_3$SO$_3$	D$_2$O, NMR	2.36·10^{-3}	2.63	71.7	4.3 (*15*)	< LOD	XylNH$_2$ (*trace*)
S7-9	[**1e**]CF$_3$SO$_3$	H$_2$O, UV-Vis	1.30·10^{-3}	2.89	64.9 ± 4.2	x	x	
S7-10	[**1e**]CF$_3$SO$_3$	H$_2$O, UV-Vis	1.18·10^{-3}	2.93	60.1 ± 2.0	x	x	
S7-11	[**1e**]NO$_3$	H$_2$O, UV-Vis	8.82·10^{-4}	3.05	63.4 ± 4.6	x	x	
S7-12	[**1e**]CF$_3$SO$_3$	H$_2$O, UV-Vis	5.87·10^{-4}	3.23	58.1 ± 5.6	x	x	
S7-13	[**1e**]NO$_3$	H$_2$O, UV-Vis	5.04·10^{-4}	3.30	57.0 ± 4.6	x	x	
S7-14	[**1e**]CF$_3$SO$_3$	H$_2$O, UV-Vis	1.74·10^{-4}	3.75	40.1 ± 1.5	x	x	
S7-15	[**1e**]NO$_3$	H$_2$O, UV-Vis	1.05·10^{-4}	3.98	28.2 ± 2.2	x	x	
S7-16	[**1e**]NO$_3$	DMEM-d, NMR	1.00·10^{-2}	2.00	87.2	1.4 (*11*)	1.4	
S7-17	[**1e**]NO$_3$	DMEM-d, NMR	5.04·10^{-3}	2.30	80.5	1.8 (*9*)	0.5	
S7-18	[**1e**]NO$_3$	DMEM-d, NMR	3.82·10^{-3}	2.42	73.8	1.4 (*6*)	< LOD	
S7-19	[**1e**]NO$_3$	DMEM-d, NMR	3.47·10^{-3}	2.46	70.7	6.0 (*21*)	trace	
S7-20	[**1e**]NO$_3$	DMEM-d, NMR	3.35·10^{-3}	2.47	77.3	1.8 (*8*)	< LOD	
S7-21	[**1e**]CF$_3$SO$_3$	DMEM-d, NMR	2.90·10^{-3}	2.54	71.0	2.7 (*9*)	< LOD	
S7-22	[**1e**]CF$_3$SO$_3$	DMEM-d, NMR	2.74·10^{-3}	2.56	69.9	3.6 (*12*)	2.9	
S7-23	[**1e**]CF$_3$SO$_3$	DMEM-d, NMR	1.43·10^{-3}	2.84	59.6	6.3 (*16*)	< LOD	
S7-24	[**1e**]NO$_3$	DMEM-d, NMR	1.01·10^{-3}	3.00	48.1	5.3 (*10*)	< LOD	

[a] ^{1}H NMR experiments: the initial concentration and % relative amount of compounds with respect to the freshly prepared solution were calculated using Me$_2$SO$_2$ or DSS as internal standard. UV-Vis experiments: the initial concentration was calculated from mass and volume data (volumetric solutions) or from the molar absorbance at 340 nm of the freshly-prepared solution; the % residual amount of starting material was calculated by the relative decrease of the UV-Vis peak at 340 nm (see main text for details). Data expressed with 2 or 1 decimal digits to avoid excessive rounding. [c] Including XylNH$_2$.

Figure S31. % Residual amount of [**1e**]$^+$ after 72 h at 37 °C *vs.* decreasing initial molar concentration (logarithmic scale, − Log c^0_{Fe2} = pFe^0) in water or cell culture medium solution. Data refer to Table S7. Dotted lines represent a linear fitting of the data.

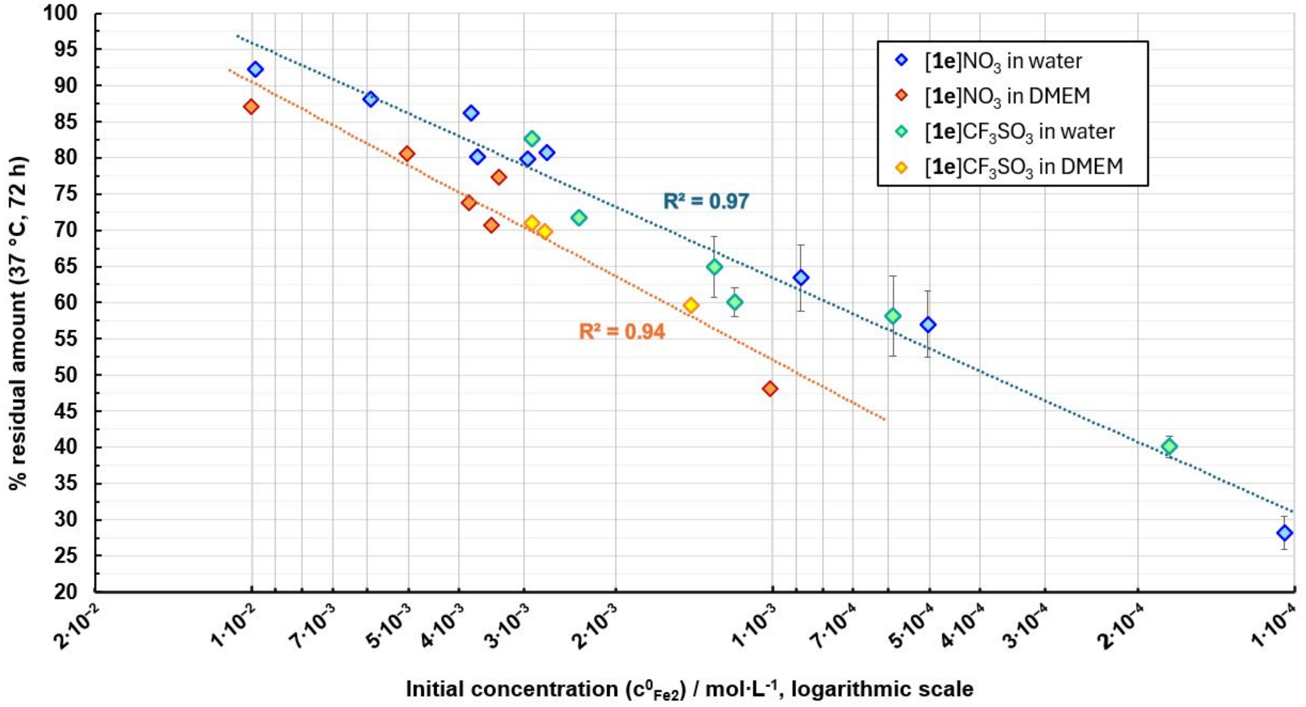

Figure S32. % residual amount of [**1a-e**]$^+$ 72 h at 37 °C *vs.* decreasing initial molar concentration (− c^0_{Fe2}) of the aqueous solution (data in Tables S3-S7). Dashed orange line: calculated data according to a zero-order kinetics with rate constant k = 7.0 μM/h.

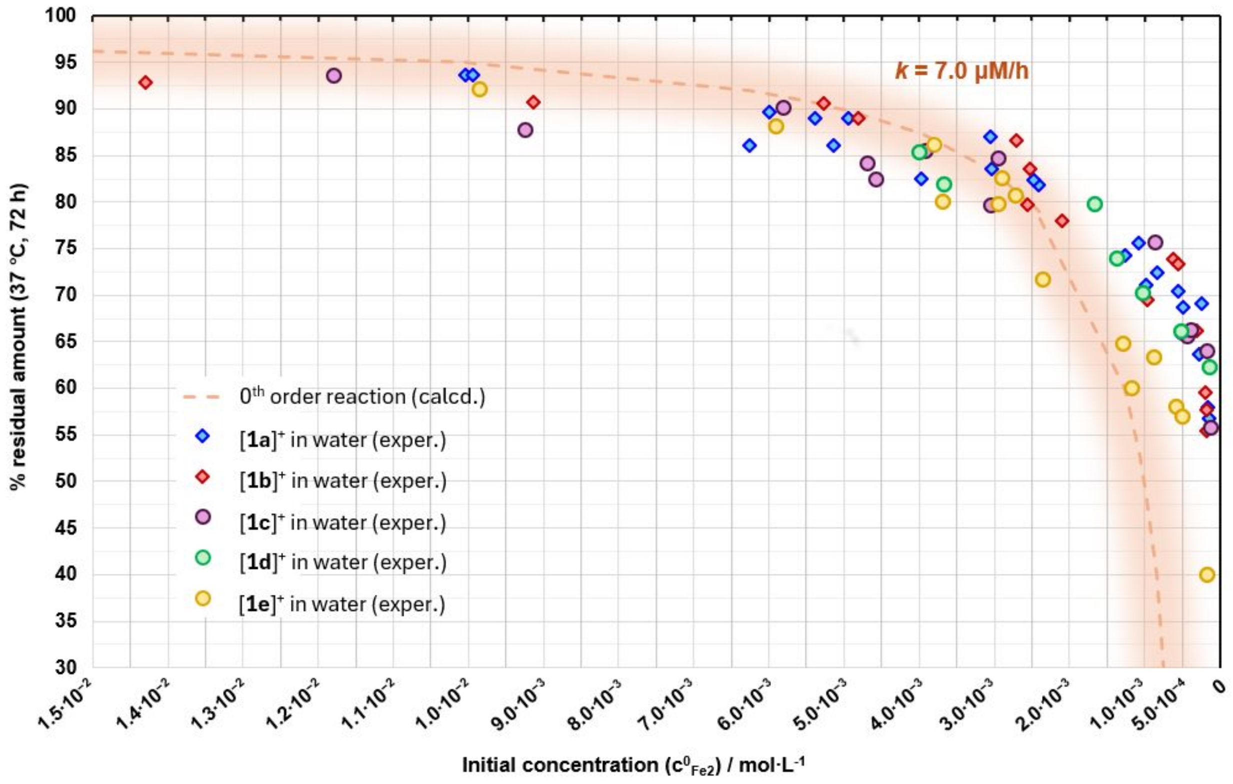

Figure S33. Blue squares: % residual amount of [**1a**]$^+$ after 72 h at 37 °C *vs.* decreasing initial molar concentration ($-\ c^0_{Fe2}$) of the aqueous solution (data in Table S3). Dashed lines: calculated data according to a zero-order kinetics with rate constant k = 7.0 µM/h (orange), 4.0 µM/h (pink), 2.5 µM/h (green). Dashed gray line: calculated data according to a first-order kinetics with rate constant $k = 4.5 \cdot 10^{-3}\ h^{-1}$.

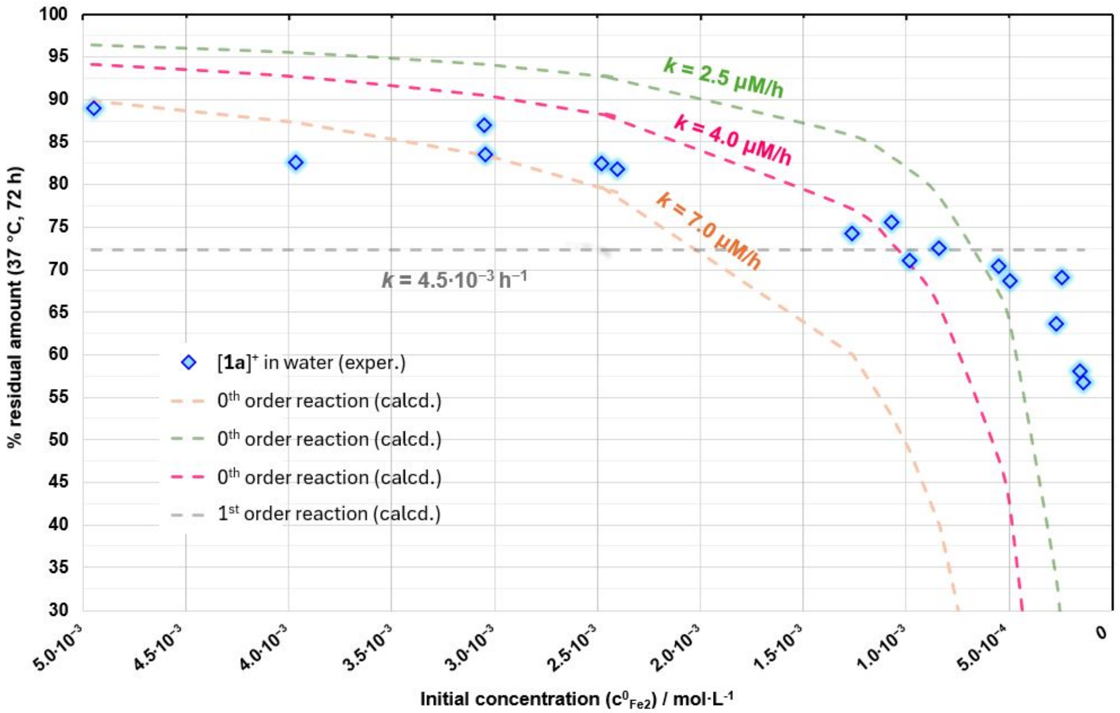

Figure S34. % Residual amount of [**1a-c,e**]$^+$ after 72 h at 37 °C *vs.* decreasing initial molar concentration of the DMEM-d solution (logarithmic scale, $-$ Log c^0_{Fe2} = pFe^0). Data refer to Tables S3-S7. Dotted lines represent a linear fitting of the data.

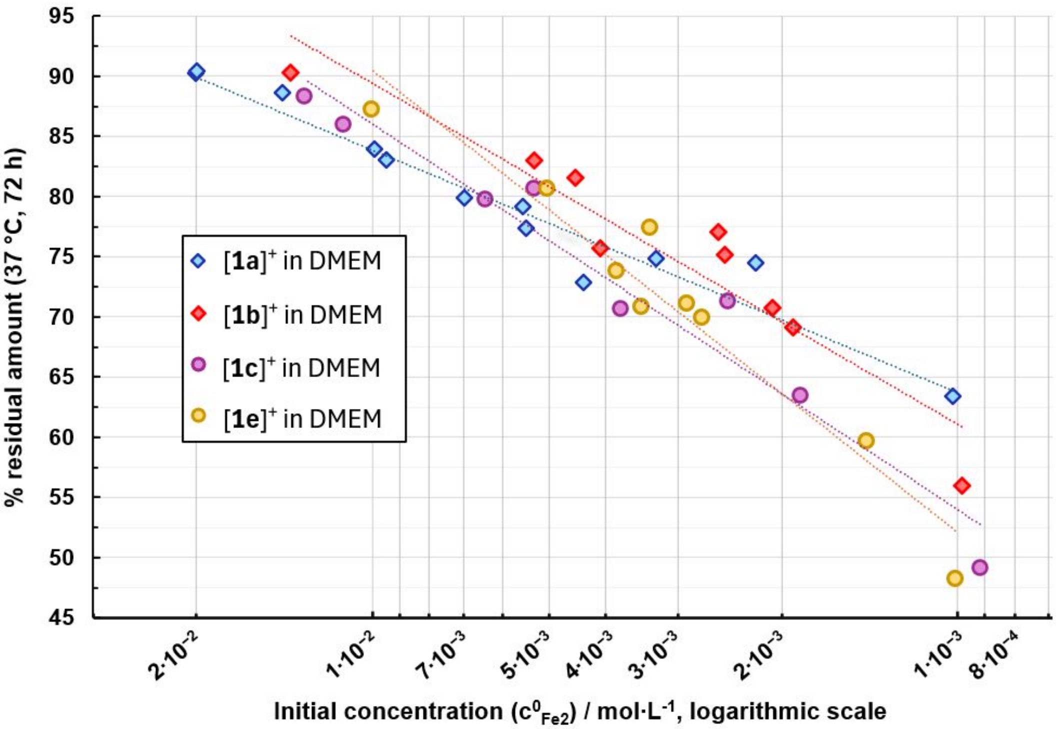

Table S8. ^{1}H NMR analyses of solutions of [**1b**]NO_3 in water (D_2O) or cell culture medium (DMEM-d) kept at 37 °C for 72 h. All experiments were carried out without protection from ambient light/air. All experiments were carried out in air under ambient light and refer to Table S4 (time course analysis was not previously reported).

Entry	Solution, technique	Time (hours)	[1b]$^+$ concentration [a] / mol·L^{-1}	compounds detected in solution [a] (% amount respect to the starting material)		
				**[1b]$^+$**	**Cy(Me)NH$_2^+$**	**CpH**
S4-4	D_2O	0	$4.82\cdot10^{-3}$ (c^0_{Fe2})	100	0	0
		24	$4.66\cdot10^{-3}$	96.8	0.7	0.6
		48	$4.52\cdot10^{-3}$	93.8	3.1	0.5
		72	$4.28\cdot10^{-3}$	88.9	4.3	0.8
S4-17	DMEM-d	0	$5.28\cdot10^{-3}$ (c^0_{Fe2})	100	0	0
		24	$5.05\cdot10^{-3}$	95.7	1.8	1.2
		48	$4.64\cdot10^{-3}$	87.8	4.0	1.2
		72	$4.38\cdot10^{-3}$	82.9	4.8	1.4

[a] ^{1}H NMR experiments: the initial concentration and % relative amount of compounds with respect to the freshly prepared solution were calculated using Me_2SO_2 or DSS as internal standard. UV-Vis experiments: the initial concentration was calculated from mass and volume data (volumetric solutions) or from the molar absorbance at 340 nm of the freshly-prepared solution; the % residual amount of starting material was calculated by the relative decrease of the UV-Vis peak at 340 nm (see main text for details). Data expressed with 2 or 1 decimal digits to avoid excessive rounding.

Figure S35. % Relative amount of compounds detected in D_2O (left) or DMEM-d (right) solutions of [**1b**]NO_3 at 37 °C over 72 h. Data refer to Table S8.

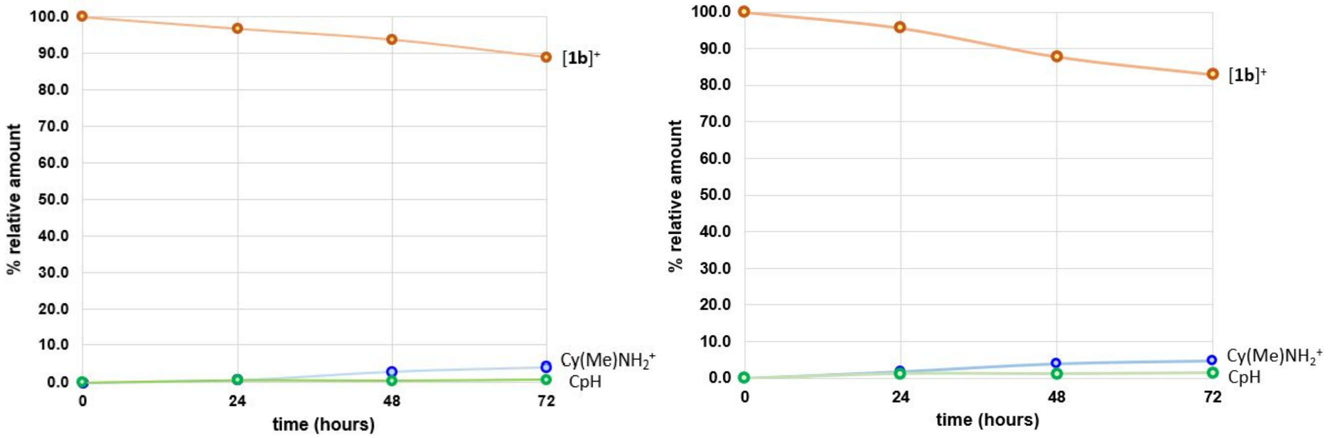

Figure S36. Molar concentration of [**1b**]$^+$ in D_2O (blue points) or DMEM-d (orange points) at 37 °C over 72 h. Dotted lines represent a linear regression of data, providing k = 7.3 μM/h and k = 13 μM/h for water and cell culture medium solution, respectively. Data refer to Table S8.

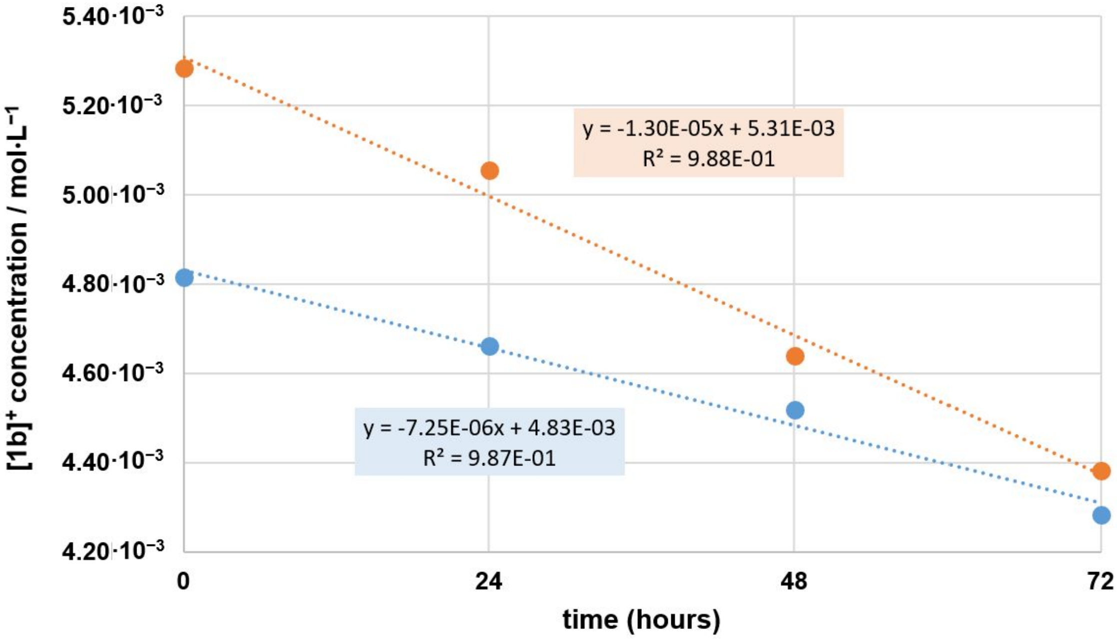

Table S9. ^{1}H NMR analyses of solutions of [**1c**]NO_3 in water (D_2O) or cell culture medium (DMEM-d) kept at 37 °C for 72 h. All experiments were carried out without protection from ambient light/air. All experiments were carried out in air under ambient light and refer to Table S5 (time course analysis was not previously reported).

Entry	Solution, technique	Time (hours)	[1c]$^+$ concentration [a] / mol·L^{-1}	compounds detected in solution [a] (% amount respect to the starting material)		
				**[1c]$^+$**	**Bn(Me)NH_2^+**	**CpH**
S5-5	D_2O	0	$4.58\cdot10^{-3}$ (c^0_{Fe2})	100	0	0
		24	$4.27\cdot10^{-3}$	93.3	1.8	0.1
		48	$4.03\cdot10^{-3}$	87.9	2.8	0.4
		72	$3.78\cdot10^{-3}$	82.5	3.9	< LOD
S5-17	DMEM-d	0	$6.40\cdot10^{-3}$ (c^0_{Fe2})	100	0	0
		24	$5.93\cdot10^{-3}$	92.6	0.7	1.4
		48	$5.50\cdot10^{-3}$	85.9	2.3	2.8
		72	$5.10\cdot10^{-3}$	79.7	4.8	*trace*

[a] ^{1}H NMR experiments: the initial concentration and % relative amount of compounds with respect to the freshly prepared solution were calculated using Me_2SO_2 or DSS as internal standard. UV-Vis experiments: the initial concentration was calculated from mass and volume data (volumetric solutions) or from the molar absorbance at 340 nm of the freshly-prepared solution; the % residual amount of starting material was calculated by the relative decrease of the UV-Vis peak at 340 nm (see main text for details). Data expressed with 2 or 1 decimal digits to avoid excessive rounding.

Figure S37. % Relative amount of compounds detected in D_2O (left) or DMEM-d (right) solutions of [**1c**]NO_3 at 37 °C over 72 h. Data refer to Table S9.

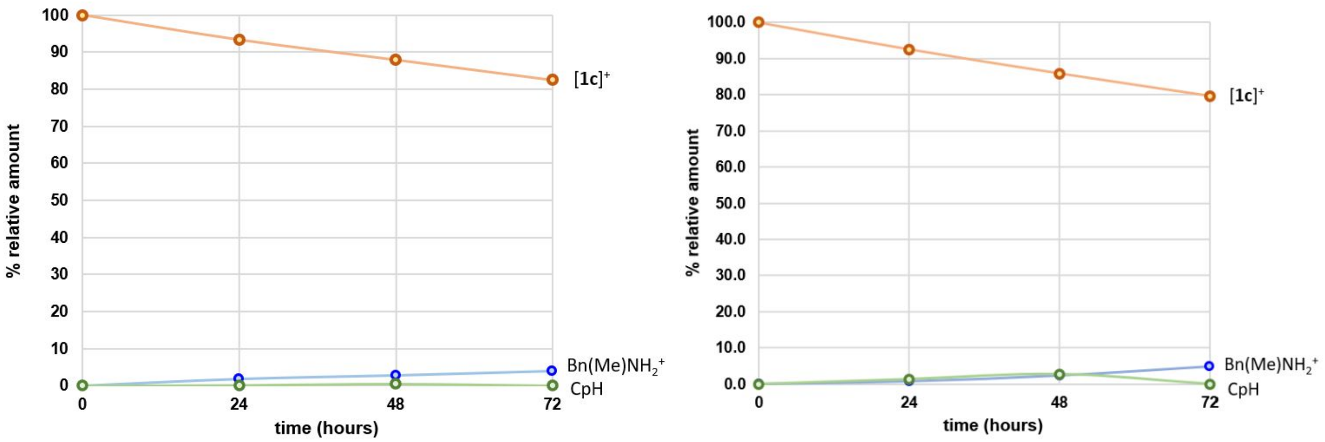

Figure S38. Molar concentration of [**1c**]$^+$ in D_2O (blue points) or DMEM-d (orange points) at 37 °C over 72 h. Dotted lines represent a linear regression of data, providing k = 11 μM/h and k = 18 μM/h for water and cell culture medium solution, respectively. Data refer to Table S9.

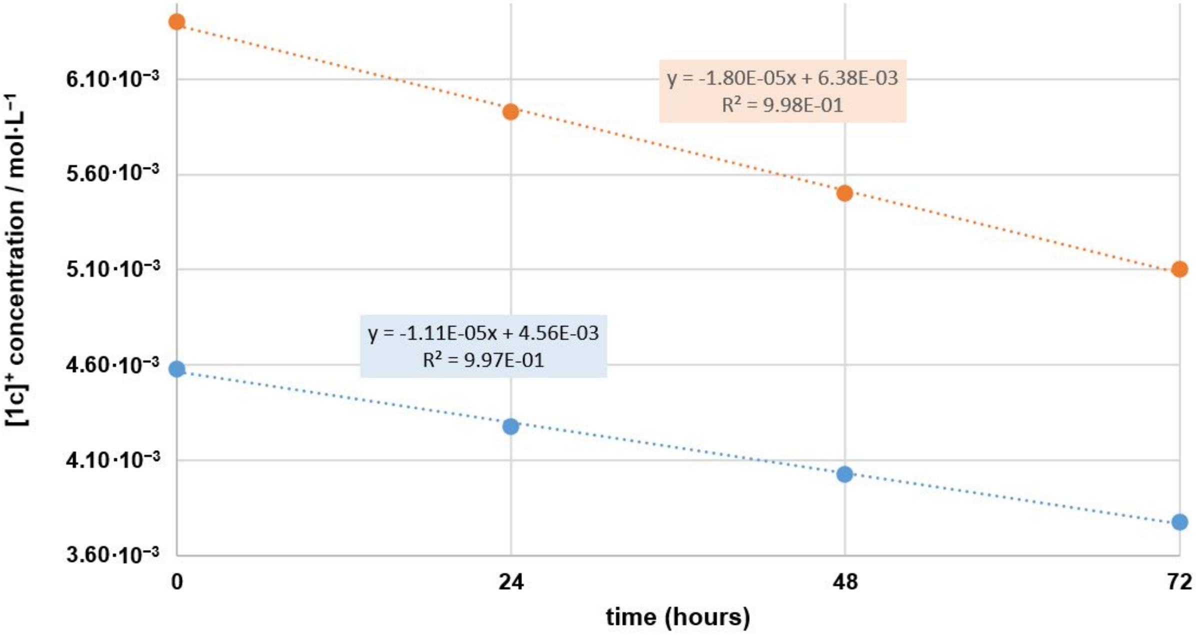

Table S10. ^{1}H NMR analyses of solutions of [**1e**]NO_3 in water (D_2O) or cell culture medium (DMEM-d) kept at 37 °C for 72 h. All experiments were carried out without protection from ambient light/air. All experiments were carried out in air under ambient light and refer to Table S7 (time course analysis was not previously reported).

**Entry**	**Solution, technique**	**Time (hours)**	**[1e]$^+$ concentration [a] / mol·L^{-1}**	**compounds detected in solution [a]** (% amount respect to the starting material)				**Notes**
				**[1e]$^+$**	**$XylNH_3^+$**	**$Xyl(Me)NH_2^+$**	**CpH**	
S7-4	D_2O	0	$3.70\cdot10^{-3}$ (c^0_{Fe2})	100	0	0	0	
		24	$3.55\cdot10^{-3}$	95.9	0.4	0	< LOD	
		48	$3.33\cdot10^{-3}$	90.2	3.2	0	< LOD	
		72	$2.96\cdot10^{-3}$	80.1	6.0	0	< LOD	
S7-19	DMEM-d	0	$3.47\cdot10^{-3}$ (c^0_{Fe2})	100	0	0	0	
		24	$3.01\cdot10^{-3}$	86.8	0	1.4	< LOD	
		48	$2.73\cdot10^{-3}$	78.8	0	2.5	0.7	
		72	$2.45\cdot10^{-3}$	70.7	0	6.0	*trace*	

[a] ^{1}H NMR experiments: the initial concentration and % relative amount of compounds with respect to the freshly prepared solution were calculated using Me_2SO_2 or DSS as internal standard. UV-Vis experiments: the initial concentration was calculated from mass and volume data (volumetric solutions) or from the molar absorbance at 340 nm of the freshly-prepared solution; the % residual amount of starting material was calculated by the relative decrease of the UV-Vis peak at 340 nm (see main text for details). Data expressed with 2 or 1 decimal digits to avoid excessive rounding.

Figure S39. % Relative amount of compounds detected in D_2O (left) or DMEM-d (right) solutions of [**1e**]NO_3 at 37 °C over 72 h. Data refer to Table S10.

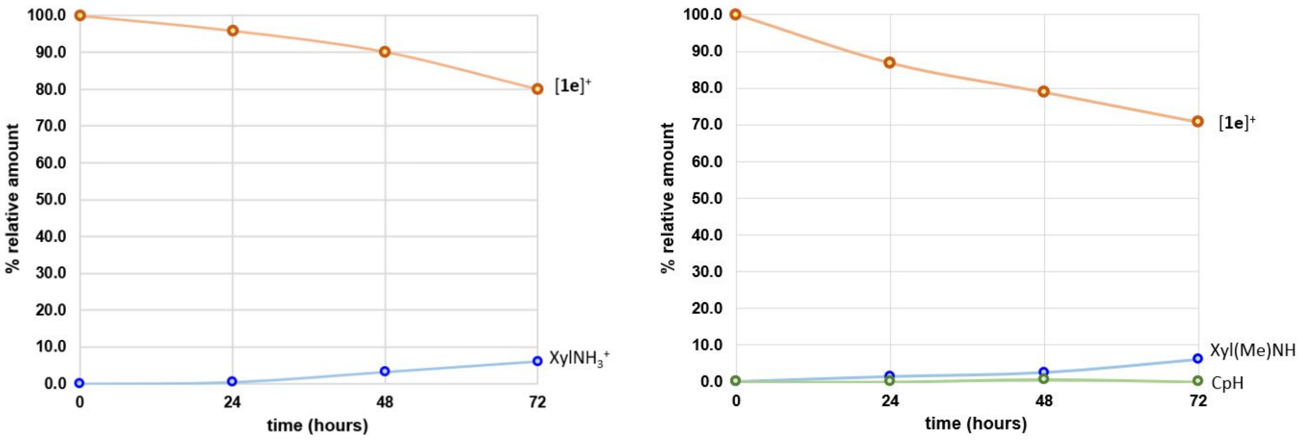

Figure S40. Molar concentration of [**1e**]$^+$ in D_2O (blue points) or DMEM-d (orange points) at 37 °C over 72 h. Dotted lines represent a linear regression of data, providing *k* = 10 μM/h and *k* = 14 μM/h for water and cell culture medium solution, respectively. Data refer to Table S10.

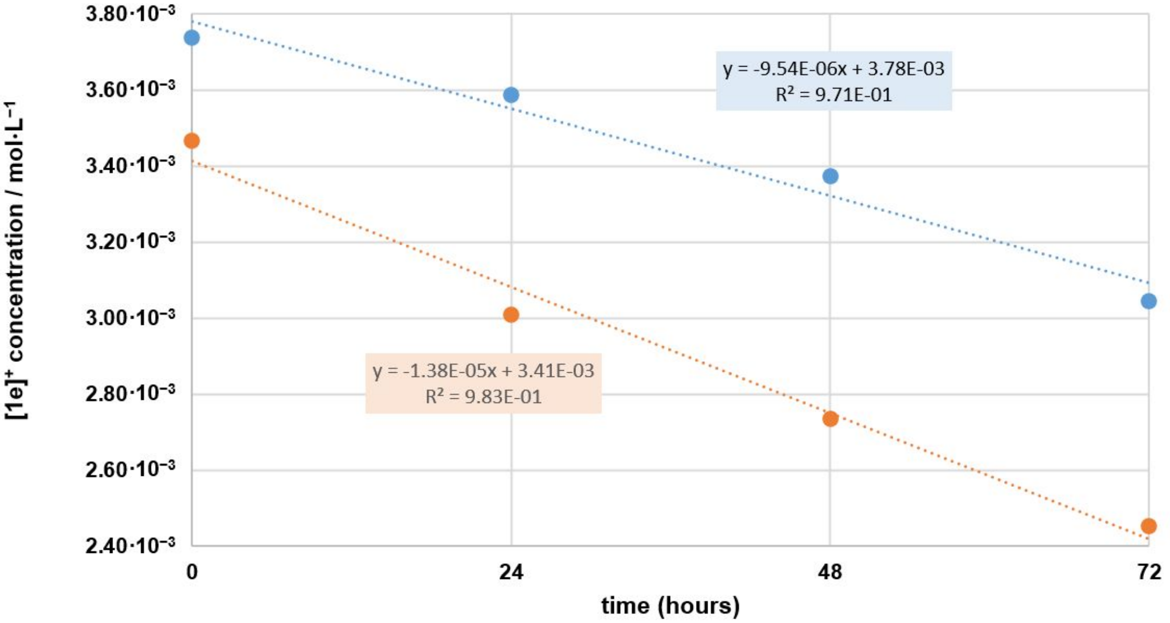

Preparation, characterization and identification of amines/ammonium salts and cyclopentadiene

The preparation of selected ammonium salts is described below; all the other compounds were commercially available. D_2O solutions of ammonium salts (3-6 mg) in D_2O were spiked with few mg of Me_2SO_2 and the ^1H NMR spectrum was recorded. Afterwards, excess $NaHCO_3$ or K_2CO_3 were added (0.10 mL of a 1.0 mol/L solution in D_2O, *ca*. 5 eq. *vs*. ammonium) to obtain a near-physiological or a more basic pH (pD), respectively. The respective ^1H NMR spectra are indicated as D_2O+$NaHCO_3$ or D_2O+K_2CO_3. Deprotonation of the ammonium ion was indicated by gas evolution. In the case of $Bn_2NH_2^+$, massive precipitation occurred due to the poor solubility of dibenzylamine at slightly basic pH. Xylyl amine, dibenzylamine and *p*-anisidine were either commercially available or obtained *in situ* by $NaHCO_3$ addition to the respective ammonium salt in D_2O. ^1H NMR data in D_2O are referenced to Me_2SO_2 (δ_H = 3.14 ppm).

Methylammonium chloride, [MeNH$_3$]Cl. *p*K$_a$ (H_2O) = 10.66. ^1H NMR (D_2O): δ/ppm = 2.59 (s, CH_3). $^{13}C\{^1H\}$ NMR (D_2O): δ/ppm = 24.6 (CH_3). ^1H NMR (D_2O+$NaHCO_3$): identical to the spectrum in D_2O.

Dimethylammonium iodide, [Me$_2$NH$_2$]I. *p*K$_a$ (H_2O) = 10.71. ^1H NMR (D_2O): δ/ppm = 2.71 (s, CH_3). $^{13}C\{^1H\}$ NMR (D_2O): δ/ppm = 34.6 (CH_3). ^1H NMR (D_2O+$NaHCO_3$): identical to the spectrum in D_2O.

Me$_2$NH/Me$_2$NH$_2^+$ equilibrium mixture. ^1H NMR (D_2O+K_2CO_3): δ/ppm = 2.59.

Cyclohexylammonium chloride, [CyNH$_3$]Cl. *p*K$_a$ (H_2O) = 10.7.[9] Prepared as described for [XylNH$_3$]Cl, using cyclohexylamine (0.15 mL, 1.3 mmol) and 2.0 mol/L $HCl_{(aq)}$ (0.70 mL, 1.4 mmol). Colorless solid. Yield = 160 mg, 90 %. ^1H NMR (D_2O): δ/ppm = *ca*. 3.14* (NCH); 2.07–1.94 (m, 2H), 1.87–1.73 (m, 2H), 1.65 (d, *J* = 12.6 Hz, 1H), 1.41–1.26 (m, 4H), 1.23–1.11 (m, 1H) (CH_2); *over Me_2SO_2 peak. ^1H NMR (D_2O+$NaHCO_3$): identical to the spectrum in D_2O.

***N*-methyl-*N*-cyclohexylammonium chloride, [CyMeNH$_2$]Cl.** *p*K$_a$ (H_2O) = 11.05.[10] Prepared as described for [XylNH$_3$]Cl, using *N*-methyl-*N*-cyclohexylamine (0.15 mL, 1.15 mmol) and 2.0 mol/L $HCl_{(aq)}$ (0.6 mL, *ca*. 1.2 mmol). Colorless solid. Yield: 132 mg, 77 %. ^1H NMR (D_2O): δ/ppm = 3.03 (m,

1H), 2.67 (s, 3H), 2.08–2.02 (m, 2H), 1.89–1.79 (m, 2H), 1.66 (d, $J = 12.5$ Hz, 1H), 1.32 (quint, $J = 9.3$ Hz, 4H), 1.24–1.09 (m, 1H). $^{13}C\{^1H\}$ NMR (D_2O): δ/ppm = 58.5 (NCH^{Cy}), 29.8 (NMe), 28.8, 24.7, 24.0. ^1H NMR (D_2O+$NaHCO_3$): identical to the spectrum in D_2O.

Reactions between cyclohexylamine and methyl iodide (1:1 ratio) at room temperature in hexane, Et_2O, THF as solvents as well as a reaction carried out with a slow, gradual addition of cyclohexylamine to a solution of methyl iodide (1:3 ratio) in Et_2O resulted in the precipitation of a colorless solid either consisting of [$CyNH_3$]I or a mixture of [$CyNH_3$]I and [$CyNMe_3$]I. No trace of [Cy(Me)NH_2]I was present. **[$CyNH_3$]I.** ^1H NMR ($CDCl_3$): δ/ppm = 3.80 (s-br, NH + H_2O), 3.11–3.00 (m, 1H, NCH), 2.10–2.02 (m, 2H), 1.84–1.73 (m, 2H), 1.64 (d, $J = 12.5$ Hz, 1H), 1.46–1.12 (m, 5H) (CH_2). $^{13}C\{^1H\}$ NMR ($CDCl_3$): δ/ppm = 51.4 (NCH); 33.7, 25.1, 24.7 (CH_2). **[$CyNMe_3$]I.** ^1H NMR ($CDCl_3$): δ/ppm = 3.30 (s, NCH_3). $^{13}C\{^1H\}$ ($CDCl_3$): δ/ppm = 75.0 (NCH), 52.2 (NMe); 26.8, 25.2, 24.6 (CH_2).

Bis(benzylammonium) sulfate, [Bn_2NH_2]$_2$$SO_4$. pK_a (H_2O) = 9.33.[11] Prepared as described for [$XylNH_3$]Cl, using benzylamine (0.12 mL, 1.1 mmol) and 1.0 mol/L $H_2SO_{4(aq)}$ (0.55 mL, 0.55 mmol). ^1H NMR (D_2O): δ/ppm = 7.53–7.43 (m, 5H, Ph), 4.19 (s, 2H, CH_2). **$BnNH_2$/$BnNH_3^+$** equilibrium mixture. ^1H NMR (D_2O+$NaHCO_3$): 7.53–7.42 (m, 5H, Ph), 4.18 (s, 2H, CH_2).

***N*-methyl-*N*-benzylammonium chloride, [$BnMeNH_2$]Cl.** pK_a (H_2O) = 9.58.[12] Prepared as described for [$XylNH_3$]Cl, using benzyl(methyl)amine (0.10 mL; 0.78 mmol) and 2.0 mol/L $HCl_{(aq)}$ (0.39 mL, *ca.* 0.78 mmol). Colorless solid. ^1H NMR ($CDCl_3$): δ/ppm = 9.73 (s-br, 2H, NH_2); 7.58–7.49 (m, 2H), 7.40–7.30 (m, 3H) (Ph); 4.03 (t, $^3J_{HH} = 4.9$ Hz, 2H, CH_2), 2.47 (t, $^3J_{HH} = 5.1$ Hz, 3H, CH_3).^1H NMR (D_2O): δ/ppm = 7.56–7.45 (m, 5H, Ph), 4.22 (s, 2H, CH_2), 2.72 (s, 3H, CH_3). **$BnMeNH_2^+$/BnMeNH** equilibrium mixture. ^1H NMR (D_2O+$NaHCO_3$): δ/ppm = 7.54–7.45 (m, 5H, Ph), 4.20 (s, 2H, CH_2), 2.70 (s, 3H, CH_3).

***N*-methyl-*N*-benzylamine, BnMeNH.** ^1H NMR (D_2O+K_2CO_3): δ/ppm = 7.48–7.38 (m, 5H, Ph), 3.82 (s, 2H, CH_2), 2.41 (s, 3H, CH_3).

Dibenzylammonium chloride, [Bn_2NH_2]Cl. pK_a (H_2O) = 8.34[13] - 8.52.[14] Prepared as described for [$XylNH_3$]Cl, using dibenzylamine (0.10 mL, 0.52 mmol) and 2.0 mol/L $HCl_{(aq)}$ (0.26 mL, 0.52 mmol).

Colorless solid. ^{1}H NMR ($CDCl_3$): δ/ppm = 7.48 (d, J = 7.0 Hz, 4H), 7.40–7.29 (m, 6H) (Ph); 3.85 (s, 4H, CH_2). ^{1}H NMR (D_2O): δ/ppm = 7.57–7.41 (m, 10H, Ph), 4.26 (s, 4H, CH_2).

Dibenzylamine, Bn_2NH. ^{1}H NMR ($CDCl_3$): δ/ppm = 7.39–7.32 (m, 8H), 7.30–7.26 (m, 2H) (Ph); 3.83 (s, 4H, CH_2), 1.71 (s-br, 1H, NH). ^{1}H NMR (D_2O+$NaHCO_3$): δ/ppm = 7.54–7.42 (m, 10H, Ph), 4.22, 4.20 (s, 4H, CH_2); massive precipitation occurred upon addition of $NaHCO_3$ to the [Bn_2NH_2]Cl solution.

Xylylammonium chloride, [$XylNH_3$]Cl. pK_a (H_2O) = 3.98.[15] A solution of 2,6-xylidine (0.15 mL, 1.2 mmol) in MeOH (4 mL) was treated with 2.0 mol/L $HCl_{(aq)}$ (0.70 mL, 1.4 mmol) and stirred at room temperature. After 1 h, volatiles were removed under vacuum (40 °C). The resulting colorless solid was triturated in Et_2O and the suspension was filtered. The solid was washed with Et_2O, hexane and dried under vacuum (40 °C). Yield: 189 mg, 98 %. ^{1}H NMR (D_2O): δ/ppm = 7.28 (dd, J = 8.8, 6.0 Hz, 1H), 7.23 (d, $^3J_{HH}$ = 7.4 Hz, 2H) (C_6H_3); 2.38 (s, 6H, CCH_3).

Xylyl amine (2,6-xylidine), $XylNH_2$. ^{1}H NMR ($CDCl_3$): δ/ppm = 6.97 (d, $^3J_{HH}$ = 7.4 Hz, 2H), 6.66 (t, $^3J_{HH}$ = 7.4 Hz, 1H) (C_6H_3); 3.58 (s-br, 2H, NH_2), 2.20 (s, 6H, CCH_3). ^{1}H NMR (D_2O+$NaHCO_3$): δ/ppm = 7.03 (d, $^3J_{HH}$ = 7.5 Hz, 2H), 6.76 (t, $^3J_{HH}$ = 7.5 Hz, 1H) (C_6H_3); 2.18 (s, 6H, CCH_3).

***N*-methyl-*N*-xylylammonium iodide, [$XylMeNH_2$]I.** pK_a (H_2O) = 6.12.[16] A solution of 2,6-xylidine (0.30 mL, 2.4 mmol) and methyl iodide (0.21 mL, 3.4 mmol) in THF (1 mL) was stirred at reflux temperature for 2 h, affording a pale orange suspension. The conversion of the amine was checked by TLC (silica gel; ethyl acetate/petroleum ether 3:1 V/V). The suspension was filtered and the resulting pale-tan solid was washed with petroleum ether and dried under vacuum (40 °C). Yield: 428 mg, 67 %. Soluble in water, DMSO. ^{1}H NMR ($CDCl_3$): δ/ppm = 7.25–7.19 (m, 1H), 7.13 (d, $^3J_{HH}$ = 7.6 Hz, 2H) (C_6H_3); 3.11 (s, 3H, NCH_3), 2.70 (s, 6H, CCH_3). ^{13}C NMR ($CDCl_3$): δ/ppm = 131.0, 130.0, 36.5, 20.4 (from ^{1}H-^{13}C HMBC). ^{1}H NMR (D_2O): δ/ppm = 7.32 (dd, $^3J_{HH}$ = 8.4, 6.7 Hz, 1H), 7.25 (d, $^3J_{HH}$ = 7.5 Hz, 2H) (C_6H_3); 3.05 (s, 3H, NCH_3), 2.43 (s, 6H, CCH_3). ***N*-methyl-*N*-xylylamine.** ^{1}H NMR (D_2O+$NaHCO_3$): δ/ppm = 7.15 (s, 3H, C_6H_3); 3.02 (s, 3H, NCH_3), 2.18 (s, 6H, CCH_3).

4-Methoxyphenylammonium chloride, [AnisNH$_3$]Cl. pK$_a$ (H_2O) = 5.36.[17] Prepared as described for [XylNH$_3$]Cl, using *p*-anisidine (110 mg, 0.895 mmol) and 2.0 mol/L $HCl_{(aq)}$ (0.50 mL, 1.0 mmol). Colorless/faint violet solid. Yield: 137 mg, 98 %. ^{1}H NMR (D_2O): δ/ppm = 7.34 (d, $^3J_{HH}$ = 8.9 Hz, 2H), 7.10 (d, $^3J_{HH}$ = 8.8 Hz, 2H) (C_6H_4); 3.85 (s, 3H, OCH_3).

4-Methoxyaniline (*p*-anisidine), *p*-C$_6$H$_4$(OMe)(NH$_2$), AnisNH$_2$. ^{1}H NMR ($CDCl_3$): δ/ppm = 6.80–6.72 (m, 2H), 6.67–6.63 (m, 2H) (C_6H_4); 3.75 (s, 3H, OMe); 3.43 (s-br, 2H, NH_2). ^{1}H NMR (D_2O+$NaHCO_3$): δ/ppm = 6.89 (d, $^3J_{HH}$ = 8.5 Hz, 2H), 6.85 (d, $^3J_{HH}$ = 8.5 Hz, 2H) (C_6H_4); 3.78 (s, 3H, OCH_3).

***N*-methyl-*N*-(4-methoxyphenyl)ammonium iodide, [AnisMeNH$_2$]I.** pK$_a$ (95 % EtOH) = 4.47.[18] A suspension of *p*-anisidine (189 mg, 1.53 mmol) and methyl iodide (0.10 mL, 1.6 mmol) in hexane (10 mL) was stirred at 45 °C in a closed 25 mL round bottom flask. After 11 h, the resulting suspension (pale yellow solution, pale pink-beige solid) was filtered (G3 sintered glass filter). The solid was washed with Et_2O, hexane and dried under vacuum (40 °C). Yield: 183 mg as a mixture of the desired compound and a byproduct containing the anisidine fragment (2:1 mol ratio). Related reactions carried out in Et_2O (room T), toluene (50°C) or THF (reflux T) gave mixtures of products not containing the title compound, while a reaction carried out in THF (room T) gave a mixture of 4 products among which the title compound in *ca*. 25 % amount. ^{1}H NMR ($CDCl_3$): δ/ppm = 7.60 (d, $^3J_{HH}$ = 8.9 Hz, 2H), 6.95 (d, $^3J_{HH}$ = 8.9 Hz, 2H) (C_6H_4); 4.79 (br, NH_2); 3.81 (s, 3H, OCH_3), 3.00 (s, 3H, NCH_3). ^{1}H NMR (D_2O): δ/ppm = 7.42 (d, $^3J_{HH}$ = 9.0 Hz, 2H), 7.13 (d, $^3J_{HH}$ = 9.1 Hz, 2H) (C_6H_4); 3.87 (s, 3H, OCH_3), 3.06 (s, 3H, NCH_3). ***N*-methyl-*N*-(4-methoxyphenyl)amine, AnisMeNH.** ^{1}H NMR (D_2O+$NaHCO_3$): δ/ppm = 6.95 (d, $^3J_{HH}$ = 8.5 Hz, 2H), 6.86 (d, $^3J_{HH}$ = 8.7 Hz, 2H) (C_6H_4); 3.78 (s, 3H, OCH_3), 2.72 (s, 3H, NCH_3). The reaction of *p*-anisidine, methyl iodide and potassium carbonate in DMF at 55 °C, as described in the literature,[19] gave a complex mixture of compounds.

Figure S41. ^{1}H NMR spectra (400 MHz, D_2O) of $[Me_2NH_2]$**I** (top, blue line), a freshly prepared solution of [**1a**]NO_3 (middle, green line) and the same solution after 1 month at room temperature (red, bottom line).

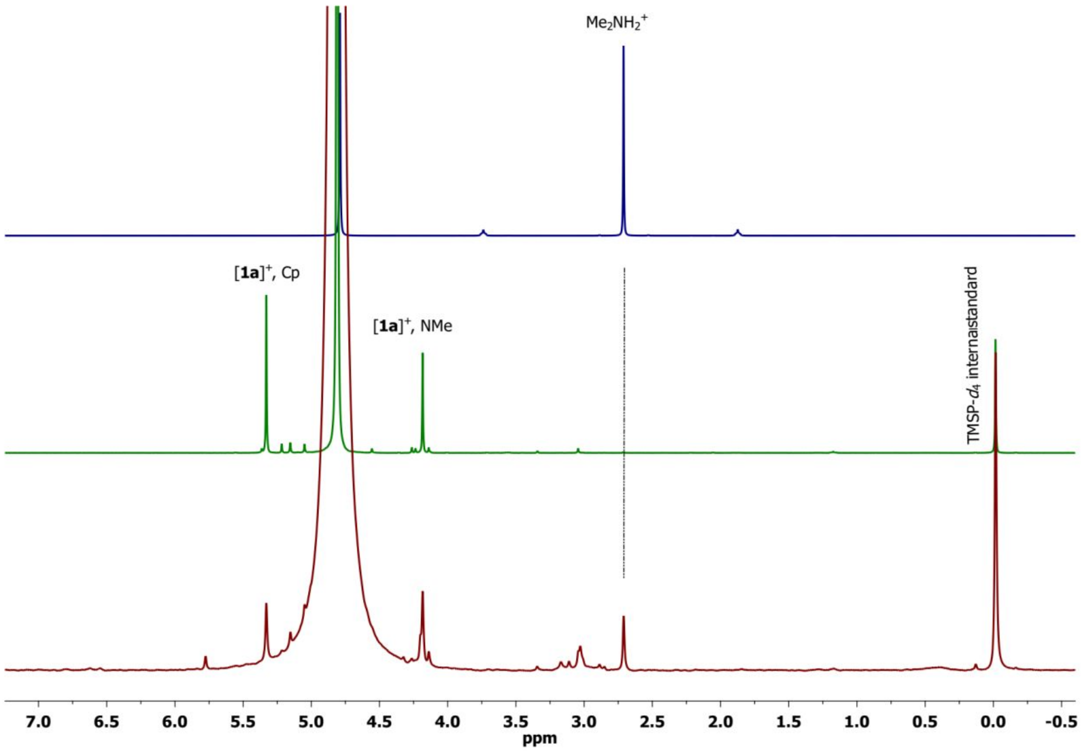

Figure S42. ^{1}H NMR spectra (400 MHz, D_2O) of $[CyMeNH_2]Cl$ (top, blue line), a freshly prepared solution of [**1b**]NO_3 (middle, green line) and the same solution after *ca*. 2 months at room temperature (red, bottom line).

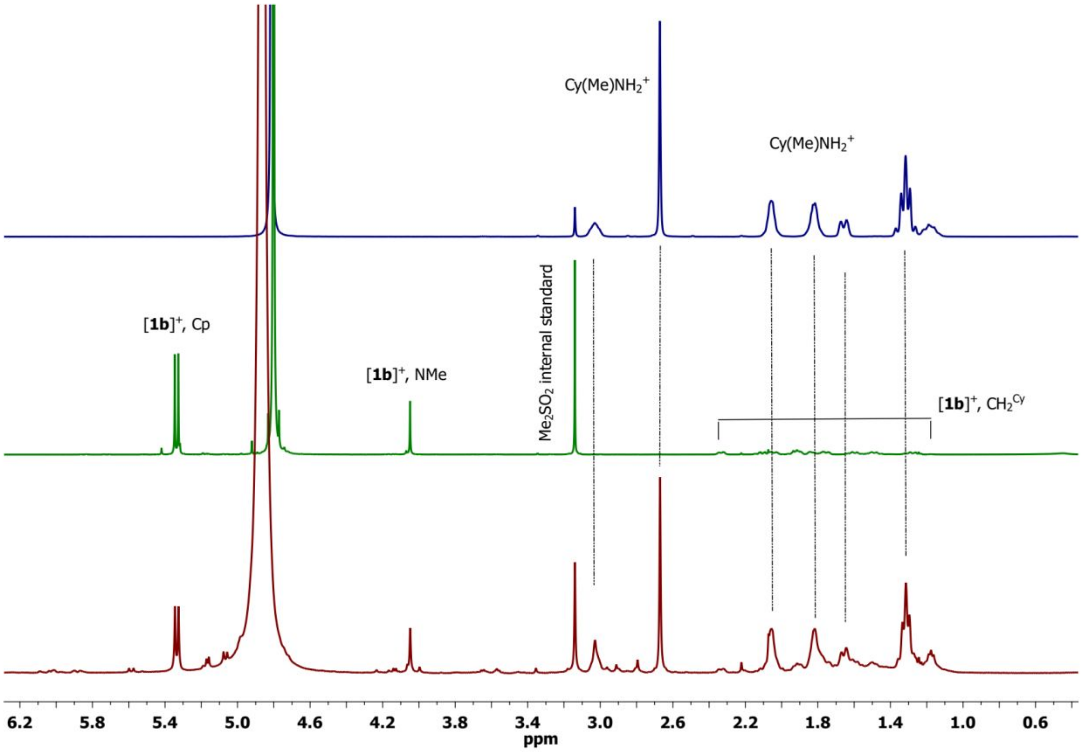

Figure S43. ^{1}H NMR spectra (400 MHz, D_2O) of [$BnMeNH_2$]Cl (top, blue line), a freshly prepared solution of [**1c**]NO_3 (middle, green line) and the same solution after *ca*. 2 months at room temperature (red, bottom line).

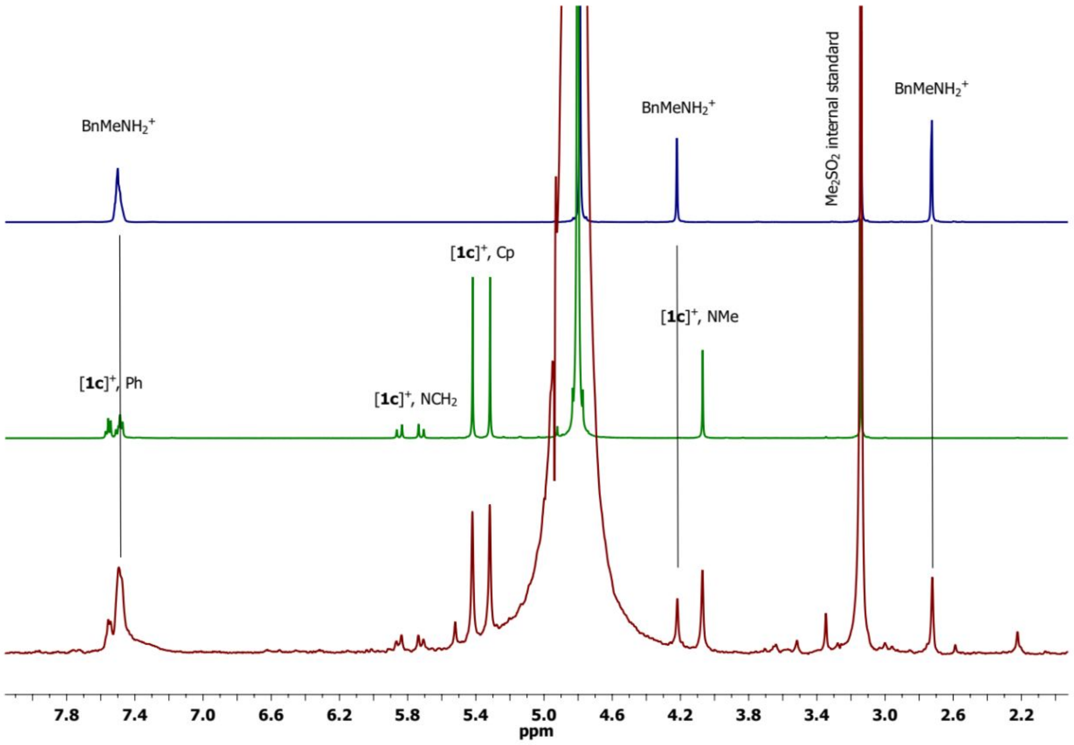

Figure S44. ^{1}H NMR spectra (400 MHz, D_2O) of [Bn_2NH_2]Cl (top, blue line), a freshly prepared solution of [**1d**]NO_3 (middle, green line) and the same solution after *ca*. 2 months at room temperature (red, bottom line).

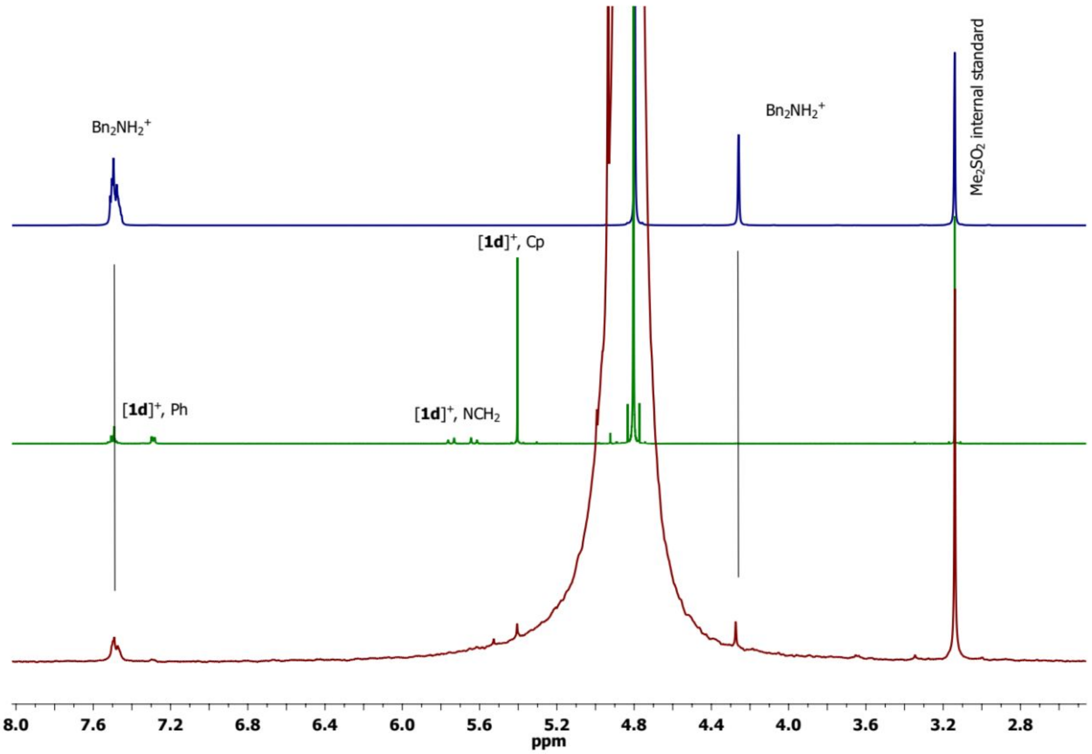

Figure S45. ^{1}H NMR spectra (400 MHz, D_2O) of [XylMeNH$_2$]**I** (top, blue line), a freshly prepared solution of [**1e**]NO_3 (middle, green line) and the same solution after *ca*. 2 months at room temperature (red, bottom line).

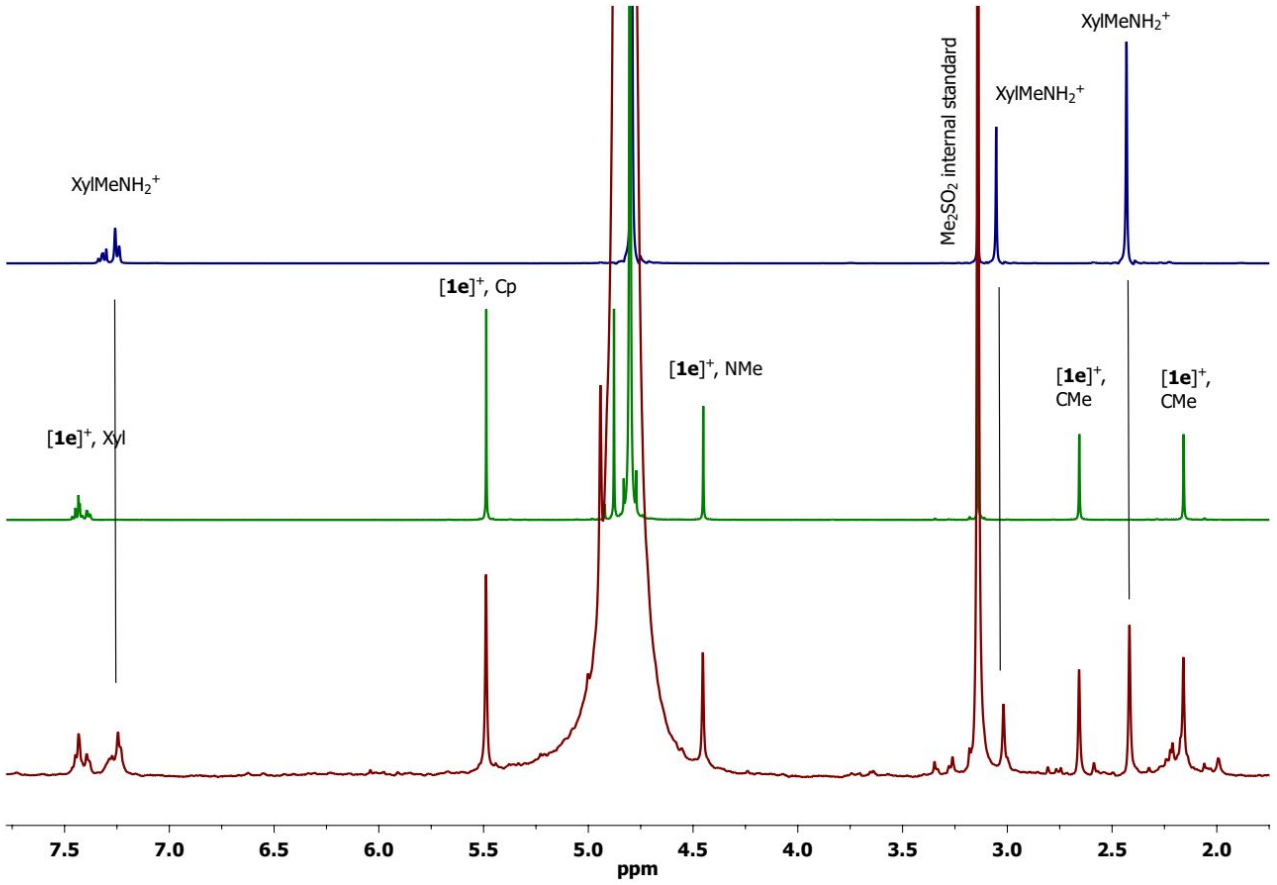

Cyclopentadiene (CpH). pK_a (H_2O) = 16.[20] ^1H NMR (D_2O): δ/ppm = 6.62 (s-br, 2H), 6.55 (s-br, 2H), 3.01 (m) as C_5H_5D formed by the reaction of Cp^- and D_2O. The observed resonances are in agreement with the literature.[21] The signal around 3 ppm is often hidden by other resonances of [**1**]$^+$, standards or other decomposition products.

Figure S46. ^1H NMR spectra (400 MHz, D_2O; 4.5-7.5 ppm) showing CpH obtained by addition of a NaCp solution in THF to D_2O (top spectrum) or from aqueous solutions of [**1a-c,e**]NO_3 after 72 h at 37 °C.

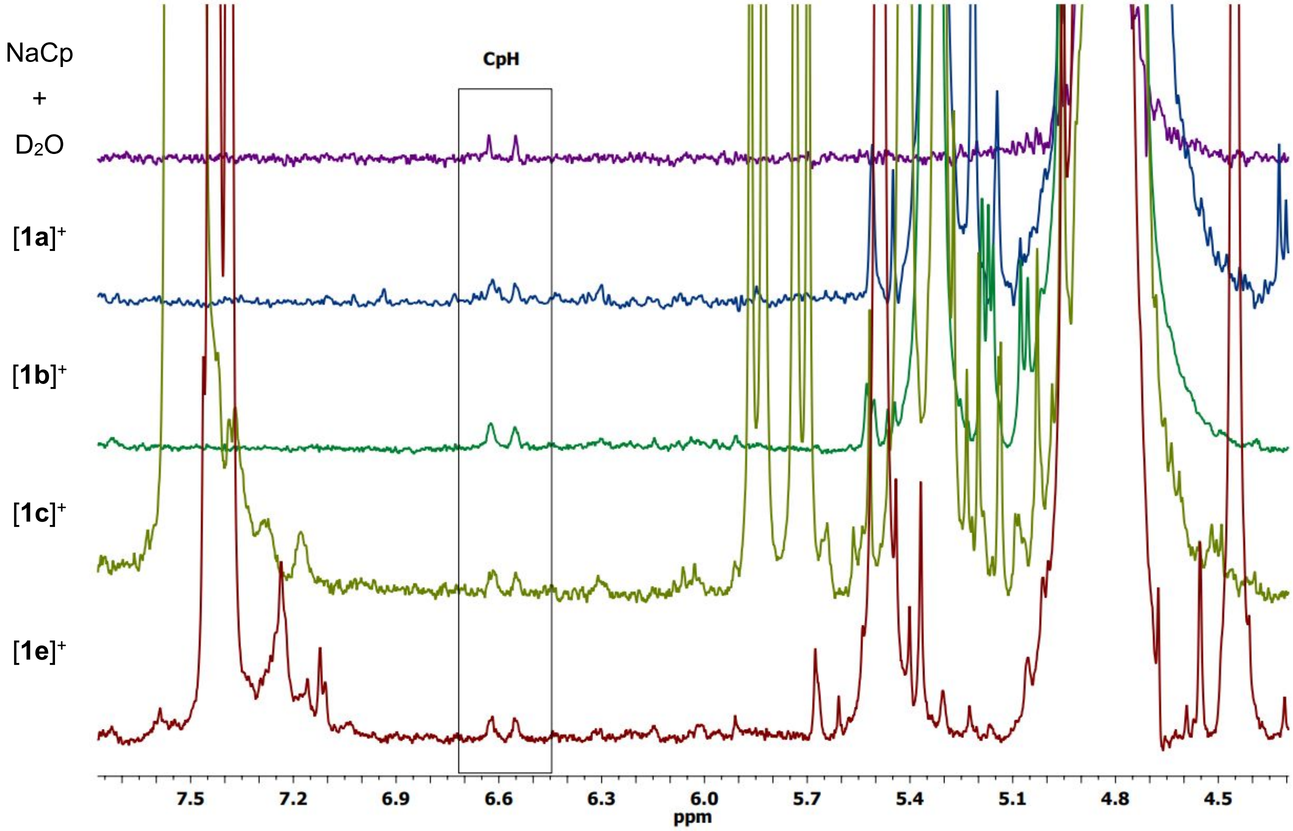

Figure S47. Plot of yield (%) of secondary amine/ammonium *vs.* initial concentration of [**1a-e**]$^+$ expressed as pFe = − Log c^0_{Fe2} (**a**). Plot of yield (%) of secondary amine (**b**) or cyclopentadiene (**c**; undistinguished) *vs.* its % ratio with respect to the % consumption (conversion) of [**1a-e**]$^+$ after 72 h. Values of 100 % and 200 %, respectively, correspond to the total decomposition reaction ([**1**]$^+$ → $R_2NH_2^+$ + 2 CpH + ..). Data refer to Tables S2-S6.

(**a**)

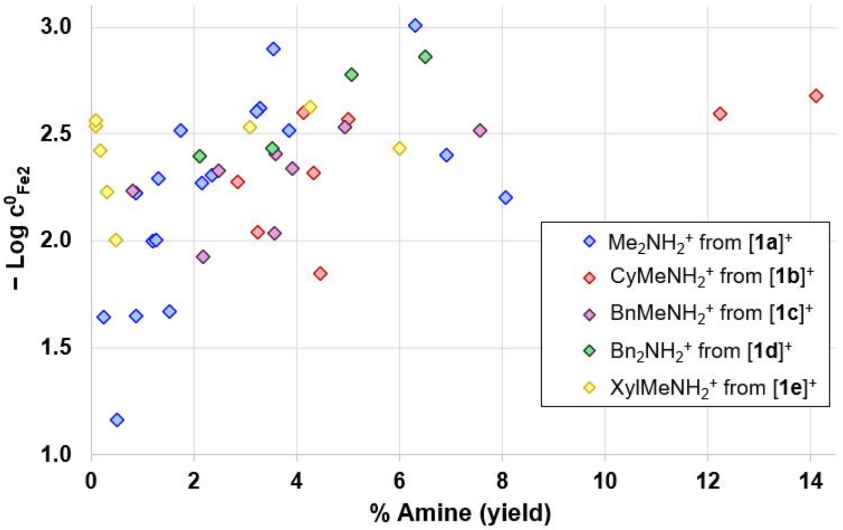

(**b**)

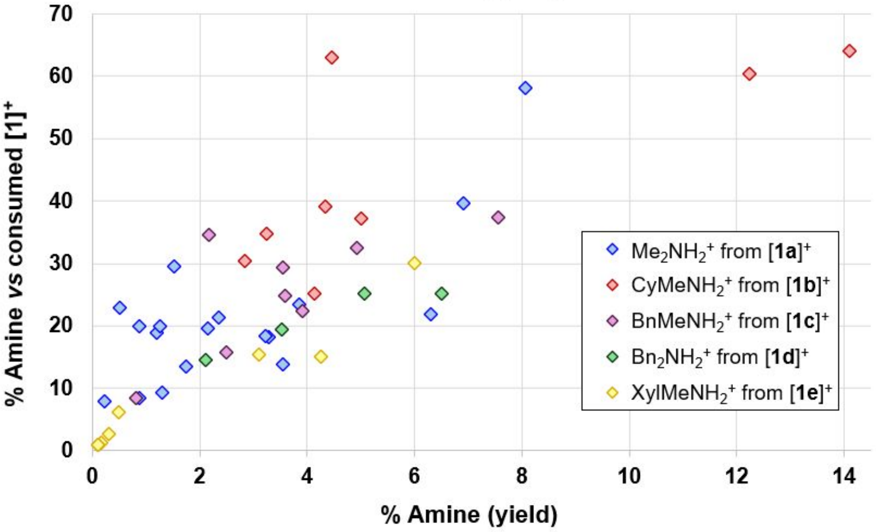

(**c**)

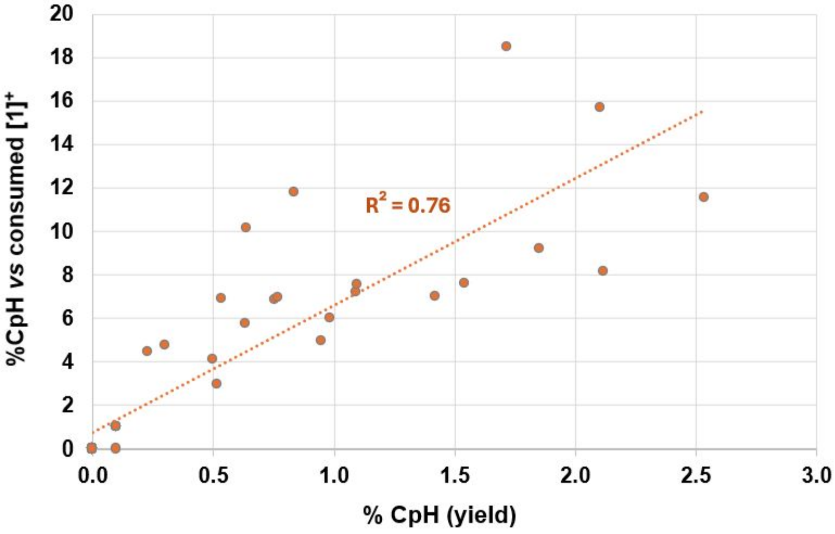

Further UV-Vis, conductivity, pH, Raman and ICP-OES analyses of solutions

Table S11. UV-Vis, pH and conductivity analyses on aqueous solutions of [**1b,c,e,f**]CF_3SO_3 kept at 37 °C for 72 h. All experiments were carried out without protection from ambient light/air. Data for reference triflate or ammonium salts is also provided.

Entry	Starting material	Time (hours)	[1]+ % residual amount (%R) [a]	[1]+ concentration / mol·L^{-1} [a]	pH	Conductivity (μS/cm)	Molar conductivity (S·cm^2·mol^{-1}) [b]	pH for net release of 1 H+ [c]
S11-1	[**1b**]CF_3SO_3	0	100	**$1.24\cdot10^{-3}$ (c^0_{Fe2})**	7.63	97 ± 1	**78**	-
		24	84.7	$1.05\cdot10^{-3}$	6.82	108 ± 2	87	*3.72*
		48	78.5	$9.74\cdot10^{-4}$	6.60	115 ± 2	93	*3.57*
		72	68.8	$8.53\cdot10^{-4}$	5.90	117 ± 1	94	*3.41*
S11-2	[**1c**]CF_3SO_3	0	100	**$1.15\cdot10^{-3}$ (c^0_{Fe2})**	7.34	93 ± 2	**81**	-
		24	86.1	$9.88\cdot10^{-4}$	6.55	93 ± 1	81	*3.80*
		48	76.1	$8.74\cdot10^{-4}$	5.77	99 ± 1	86	*3.56*
		72	68.0	$7.81\cdot10^{-4}$	5.65	101 ± 1	88	*3.44*
S11-3	[**1e**]CF_3SO_3	0	100	**$1.24\cdot10^{-3}$ (c^0_{Fe2})**	6.70	92 ± 1	**75**	-
		24	75.2	$9.31\cdot10^{-4}$	6.43	102 ± 1	82	*3.51*
		48	63.5	$7.86\cdot10^{-4}$	5.81	120 ± 1	97	*3.35*
		72	47.7	$5.91\cdot10^{-4}$	5.54	122 ± 1	98	*3.19*
S11-4	[**1f**]CF_3SO_3	0	100	**$1.16\cdot10^{-3}$ (c^0_{Fe2})**	6.69	91 ± 1	**78**	-
		24	88.2	$1.02\cdot10^{-3}$	6.20	98 ± 2	85	*3.87*
		48	80.4	$9.29\cdot10^{-4}$	6.08	106 ± 1	92	*3.65*
		72	66.5	$7.68\cdot10^{-4}$	5.81	117 ± 1	101	*3.41*
S11-5	[**1a**]CF_3SO_3	72, dark	98.5	**$1.17\cdot10^{-3}$ (c^0_{Fe2})**	6.46	102 ± 1	87	-
S11-6	[**1e**]CF_3SO_3	72, dark	88.0	**$1.26\cdot10^{-3}$ (c^0_{Fe2})**	6.41	96 ± 1	77	-
S11-7	HPLC H_2O	0	/	/	6.80	6.4	/	/
S11-8	CF_3SO_3Na	0	/	**$1.49\cdot10^{-3}$ (c^0)**	6.38	108	**70**	/
S11-9		72	/	/	6.24	114	74	/
S11-10	[Me_2NH_2]I	0	/	**$1.62\cdot10^{-3}$ (c^0)**	6.74	198	**123**	/
S11-11	[$CyMeNH_2$]Cl	0	/	**$1.44\cdot10^{-3}$ (c^0)**	6.17	132	**92**	/
S11-12	[$BnMeNH_2$]Cl	0	/	**$1.32\cdot10^{-3}$ (c^0)**	6.38	136	**103**	/
S11-13	[$XylMeNH_2$]I	0	/	**$1.19\cdot10^{-3}$ (c^0)**	5.47	128	**107**	/

[a] The initial concentration was calculated from mass and volume data (volumetric solutions) or from the molar absorbance at 340 nm of the freshly-prepared solution; the % residual amount of starting material was calculated by the relative decrease of the UV-Vis peak at 340 nm (see main text for details). Data expressed with 2 or 1 decimal digits to avoid excessive rounding. [b] Calculated with respect to the initial concentration (c^0). [c] Calculated based on the assumption that the decrease of [**1**]+ is associated to the release of one proton as – Log [10^{-pH0} + (1 − %R/100)·c^0_{Fe2}], where pH0 is the initial pH and 1 − %R/100 represents the conversion of [**1**]+.

Figure S48. Concentration of [**1b**]$^+$ (gray points), [**1c**]$^+$ (yellow points), [**1e**]$^+$ (orange points) and [**1f**]$^+$ (blue points) at 37 °C for 72 h based on UV-Vis data (Table S11). Dotted lines represent a linear regression of data, providing k = 5.2, 5.1, 8.7, 5.2 μM/h for [**1b,c,e,f**]$^+$, respectively.

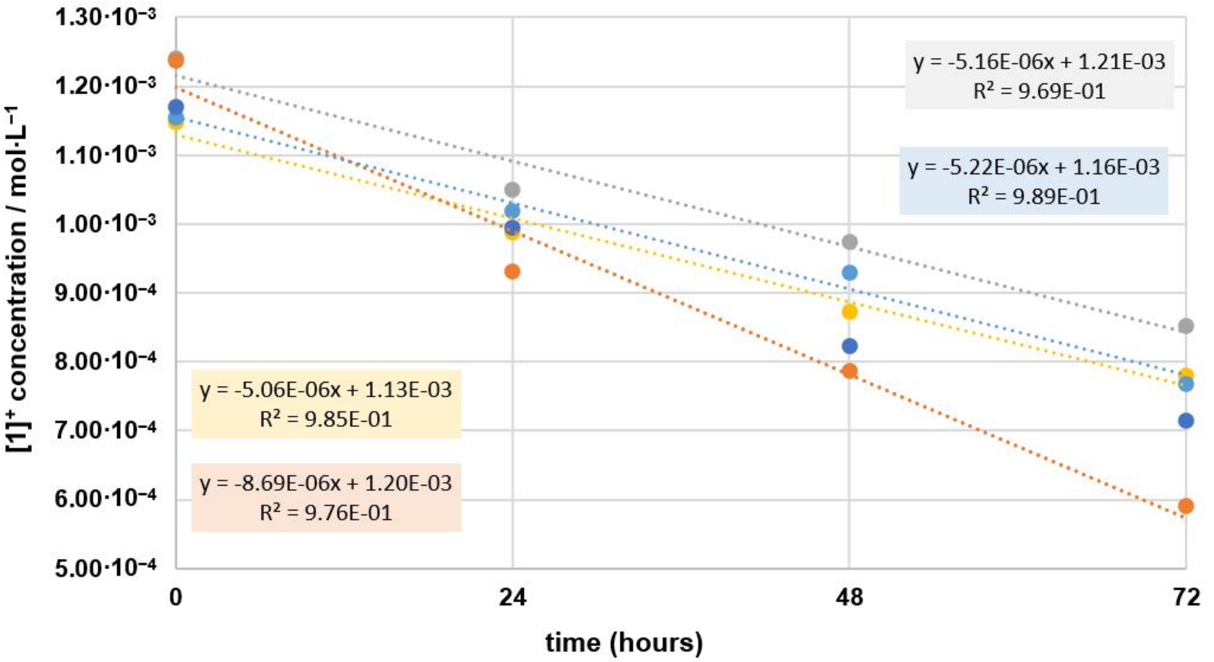

Figure S49. Monitoring pH and conductivity of aqueous solutions of [**1b,c**,**e**,**f**]CF_3SO_3 at 37 °C at 24 h intervals for up to 72 h. Data refer to Table S11.

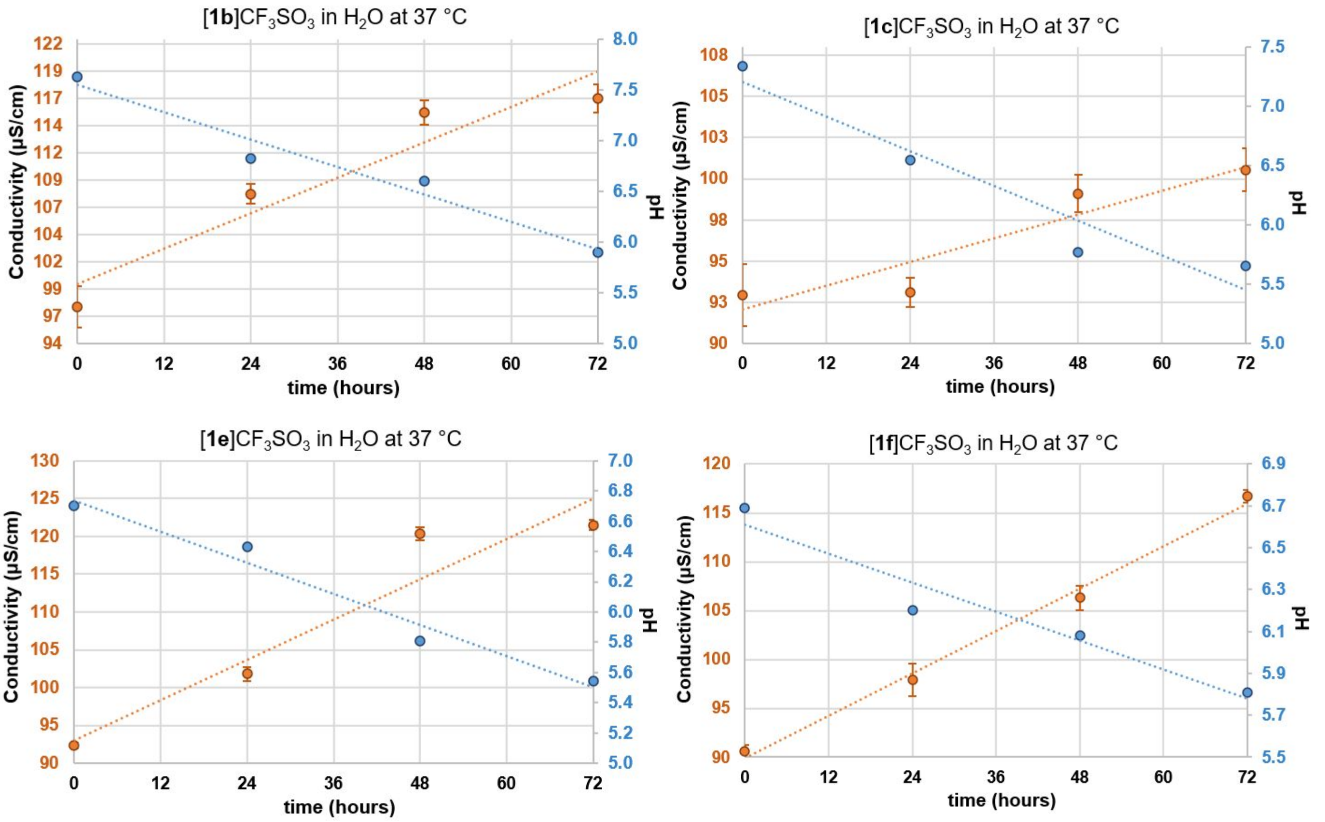

Figure S50. Raman spectrum of a *ca.* 2·10^{-2} mol/L solution of [**1a**]NO_3 in water. The inset shows a magnified view of the spectrum in the 1500–2500 cm^{-1} region.

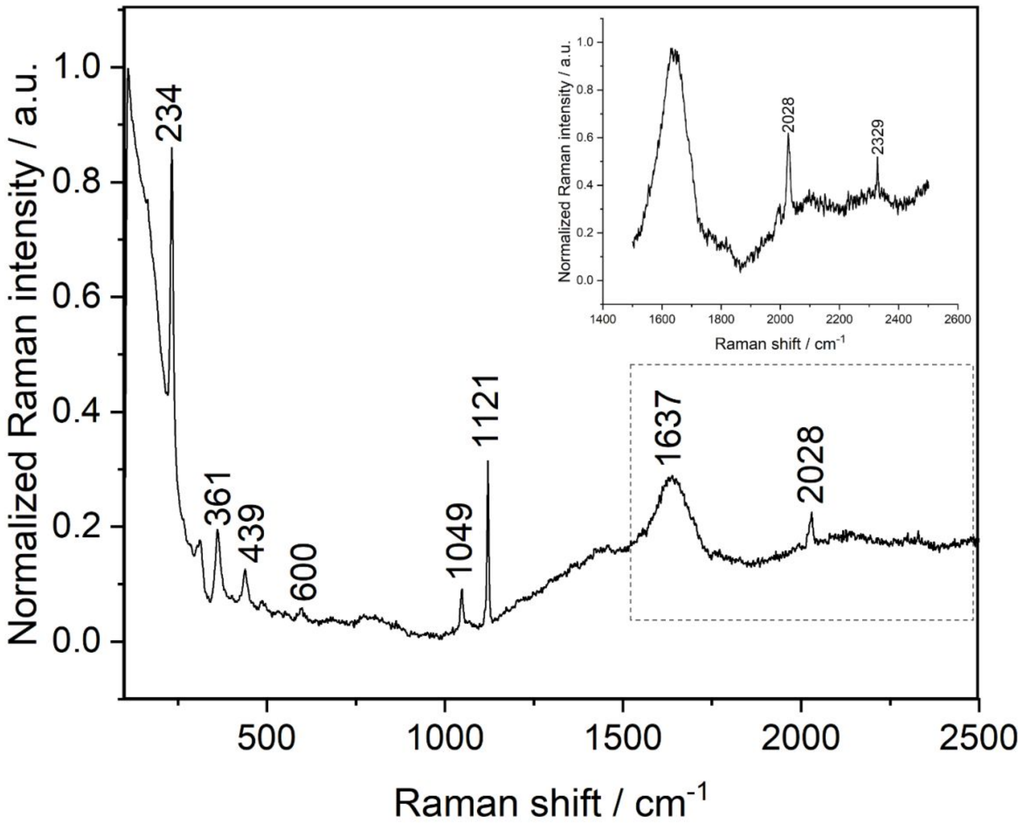

Table S12. Total soluble Fe determined via ICP-OES on solutions of [**1**]$^+$ (NO_3^- or $CF_3SO_3^-$ salts) in water or DMEM-C kept at 37 °C for 72 h. The residual % amount of iron is calculated with respect to the initial nominal amount of [**1**]$^+$. Results are compared with the residual amount of [**1**]$^+$ estimated by ^{1}H NMR and UV-Vis data.

Starting material	Solution	c^0_{Fe2} / mol·L^{-1} (freshly-prepared solution) [a]	c_{Fe}(72 h) / ppm (nominal, in the final volumetric flask)	c_{Fe}(72 h) / ppm (ICP-OES, blank-subtracted)	% residual amount [b]	Expected % residual amount based on ^{1}H NMR and UV-Vis data [c]
-	H_2O	0	0	*0.11*	-	
-	DMEM-C	2.5·10^{-7} [d]	0.014 (14 ppb) [d]	*0.18*	-	
[**1c**]NO_3	H_2O	5.77·10^{-3}	129	105.3	82	84-89
	DMEM-C	5.77·10^{-3}	129	99.2	77	76-81
[**1e**]NO_3	H_2O	3.74·10^{-3}	251	191.6	76	78-84
	DMEM-C	3.74·10^{-3}	251	178.6	71	71-76
[**1e**]CF_3SO_3	H_2O	2.41·10^{-3}	108	80.9	75	73-77
	DMEM-C	2.41·10^{-3}	108	75.4	70	65-69

[a] Correspond to the solution kept at 37 °C for 72 h. [b] Calculated with the initial mass of the diiron compound and the volume of the volumetric flask. [c] Based on residual amount *vs* pFe^0 data in Figures S29, S31 (value calculated by the linear fitting ± 2-3 % as confidence interval). [d] Corresponds to 25 µM $Fe(NO_3)_3$ as per DMEM formulation.

Characterization of water-insoluble iron compounds

Preparation and/or characterization of reference inorganic Fe compounds

General procedure. The selected iron salt, $FeSO_4$ or $Fe_2(SO_4)_3$ (100-300 mg), was dissolved in H_2O (15-20 mL) and treated with hydroxide, carbonate or phosphate salts, as specified below. The mixture was stirred at room temperature for 2-3 h then filtered on a G3 or G4 sintered glass filter. The solid was washed with H_2O and dried under vacuum (room temperature). IR bands were assigned based on the literature.[22,23]

Fe_2O_3 (commercial). Brown-red solid. IR (solid state): no absorption for $\tilde{\upsilon} > 700$ cm^{-1}. Raman (solid state): $\tilde{\upsilon}$ / cm^{-1} = 221s, 243w, 288s, 407m, 496w-br, 607w.

$FeO(OH)\cdot nH_2O$. From $Fe_2(SO_4)_3$ (393 mg, 0.983 mmol; 1.97 mmol Fe) and NaOH (6 mmol). Brown solid, 173 mg, 82 % yield as $Fe(OH)_3$. Anal. calcd. for $FeO(OH)\cdot H_2O = Fe(OH)_3$: H, 2.83 %; Anal. calcd. for $FeO(OH)\cdot(H_2O)_{0.5}$: H, 2.06 %; Found: H, 1.97 %. IR (solid state): $\tilde{\upsilon}$ / cm^{-1} = 3300-3100m-br (OH); 2979w (OH); 1640w (H_2O); 910s (OH), ≤ 650 (Fe-O). Raman (solid state): $\tilde{\upsilon}$/cm^{-1} = 712s-br.

$FeO(OH)\cdot nH_2O$. From $Fe(SO_4)$ (318 mg, 2.09 mmol) and NaOH (7 mmol). An initially green precipitate of $Fe(OH)_2$ slowly turned into brown over time. Yield: 163 mg, 55 % as FeO(OH). Anal. calcd. for $FeO(OH)\cdot H_2O = Fe(OH)_3$: H, 2.83 %; Anal. calcd. for FeO(OH): H, 1.13 % Anal. calcd. for $FeO(OH)\cdot(H_2O)_{0.1}$: H, 1.33 %; Found: H, 1.30 %. IR (solid state): qualitatively identical to the previous one.

$FePO_4\cdot nH_2O$. From $Fe_2(SO_4)_3$ (194 mg, 0.485 mmol; 0.97 mmol Fe), NaH_2PO_4 (123 mg, 1.03 mmol) and Na_2HPO_4 (155 mg, 1.09 mmol). Pale yellow solid. Yield: 129 mg, 88 % as $FePO_4$. Anal. calcd. for $FePO_4\cdot 2H_2O$: H, 2.16 %; Found: H, 2.66 %. IR (solid state): $\tilde{\upsilon}$ / cm^{-1} = 3300-3100w-br (OH); 1628w (H_2O); 991s (PO_4), 912m-sh (OH?). Raman (solid state): $\tilde{\upsilon}$/cm^{-1} = 1022s-br. A pale green solid with

identical IR spectrum was obtained from Fe(SO_4) (200 mg, 1.32 mmol), NaH_2PO_4 (160 mg, 1.33 mmol) and Na_2HPO_4 (200 mg, 1.41 mmol). Yield: 162 mg, 81 % as $FePO_4$.

FeO(OH)·nH$_2$O·Fe(CO$_3$). From Fe(SO_4) (222 mg, 1.46 mmol) and $NaHCO_3$ (617 mg, 7.34 mmol). An initially dark olive-green precipitate progressively acquired a brown shade over time. Yield: 139 mg. Anal. calcd. for $Fe_2(OH)_2(CO_3)$: C, 5.82; H, 0.97; for $[FeO(OH)]_{1.3}$·$(H_2O)_{1.7}$·$Fe(CO_3)$: C, 4.58; H, 1.81. Found: C, 4.58; H, 1.80. IR (solid state): $\tilde{\upsilon}$ / cm^{-1} = 3300-3100w-br (OH); 1630w (H_2O); 1490s-sh, 1363s (CO_3); 1073w, 853m (CO_3), ≤ 650 (Fe-O). Related reactions carried out with Na_2CO_3, either in air or under N_2, gave a brown solid characterized by a qualitatively similar IR spectrum but containing very little carbon (0.72 %). The carbonate bands disappear completely and the %C content is zero for a brown solid collected from an aqueous solution containing $FeSO_4$ and Na_2CO_3 (4:1 mol. ratio) kept at 37 °C for 11 days.

FeO(OH)·nH$_2$O with traces of an iron(III) hydroxide carbonate. From $Fe_2(SO_4)_3$ (314 mg, 0.785 mmol; 1.57 mmol Fe) and $NaHCO_3$ (650 mg, 7.73 mmol). Reddish-brow solid. Yield: 95 mg. Anal. calcd. for $[FeO(OH)]$·$(H_2O)_{0.6}$·$[Fe_2(CO_3)_3]_{0.11}$: C, 3.00; H, 1.68. Found: C, 2.91; H, 1.72. IR (solid state): similar to the previous one with weaker carbonated-related absorptions at 1479, 1330, 1070, 840 cm^{-1}.

Figure S51. IR spectra (650-4000 cm^{-1}) of FeO(OH)·nH_2O, solids obtained by addition of NaOH to solutions of $FeSO_4$ (green line) or $Fe_2(SO_4)_3$ (violet line).

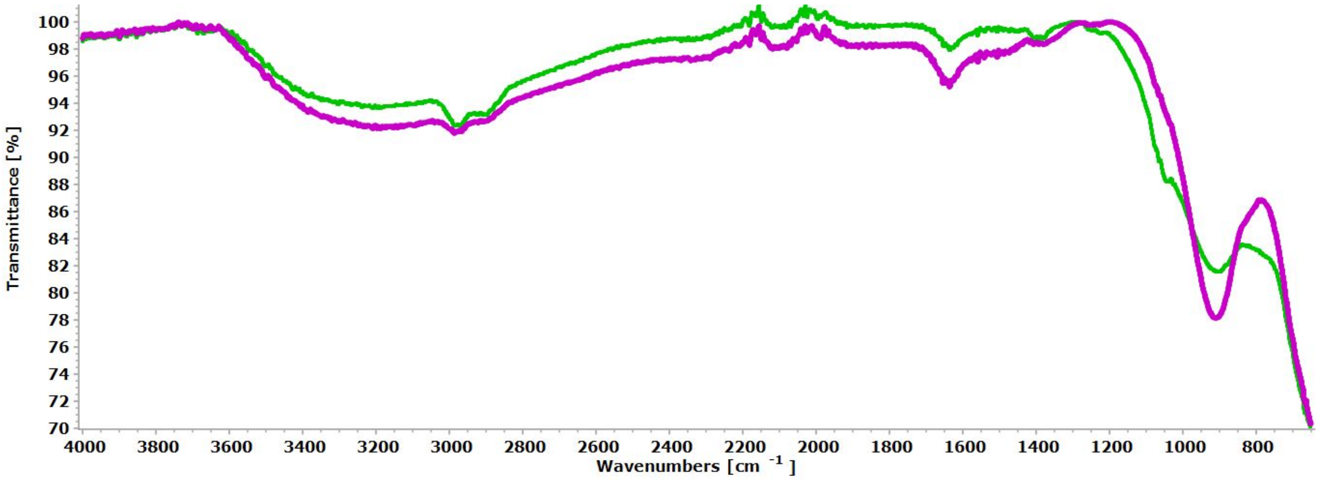

Figure S52. IR spectra (650-4000 cm^{-1}) of iron phosphates obtained by addition of $FeSO_4$ (brown line) or $Fe_2(SO_4)_3$ (dark cyan line) to a phosphate buffer solution ($NaH_2PO_4/Na_2HPO_4 \approx 1:1$; pH ≈ 7).

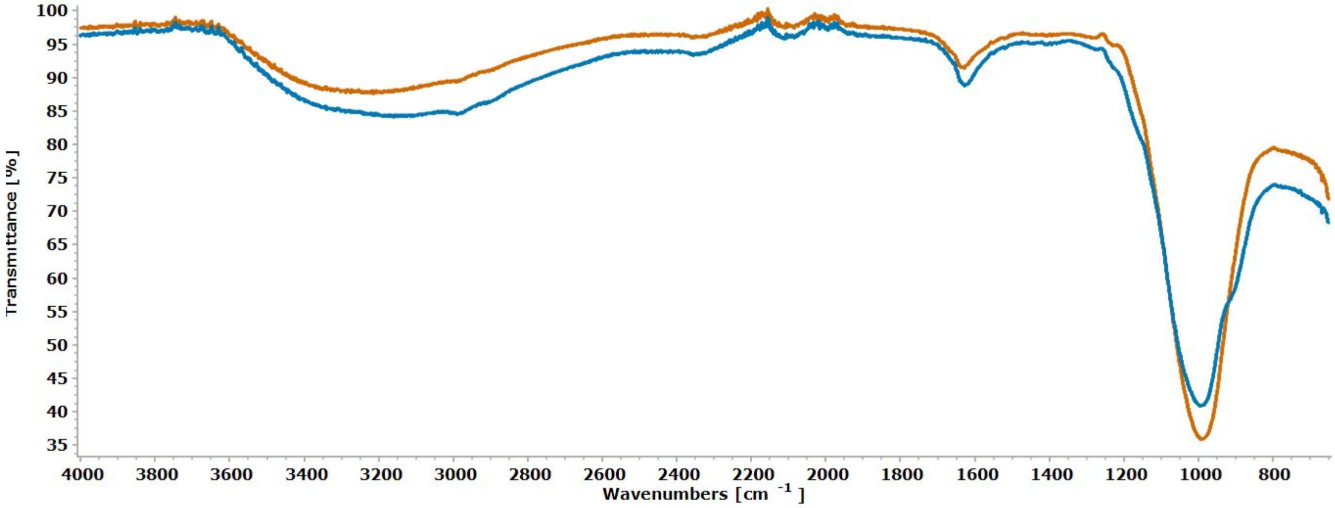

Figure S53. IR spectra (650-4000 cm^{-1}) of solids obtained by addition of $NaHCO_3$ to aqueous solutions of $FeSO_4$ (blue line, ≈ 4.6 % C) or $Fe_2(SO_4)_3$ (yellow line, ≈ 2.9 % C) or Na_2CO_3 to an aqueous solution of $FeSO_4$ (red line, ≈ 0.7 % C). Absorptions due to carbonate ligands are marked with asterisk. $FeCO_3$ contains 10 % C.

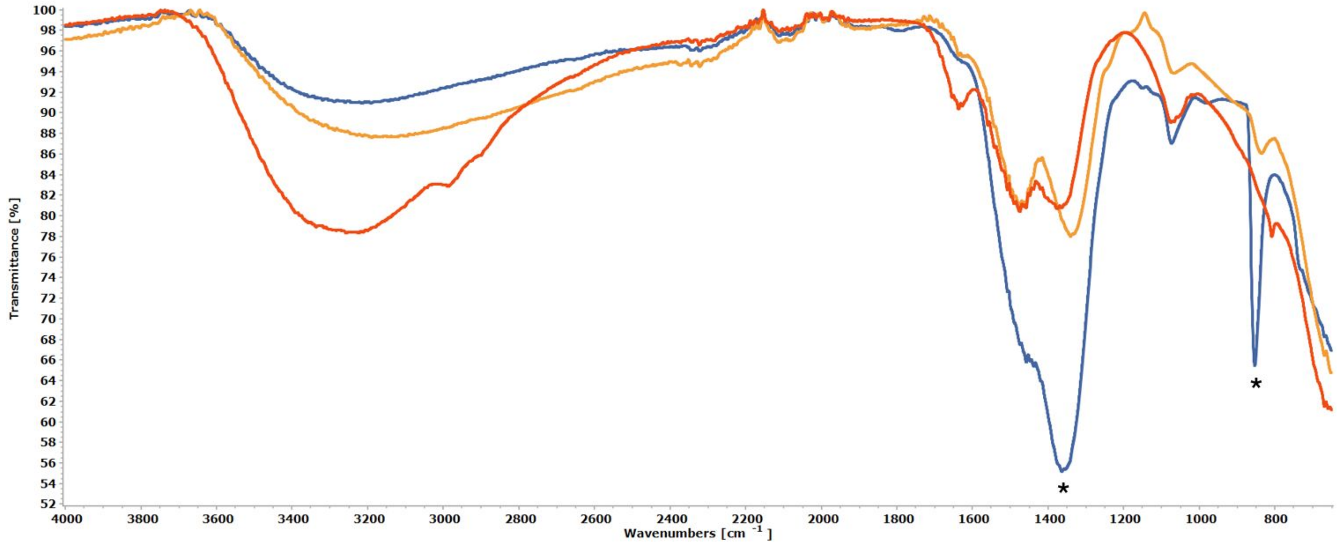

Characterization of water-insoluble products obtained from incubation of [1]$^+$ or $Fe^{2+}_{(aq)}$ in aqueous solution or cell culture media.

Sample number, experimental details and CHNS analyses are reported in Table S13. IR, Raman and ESI(+)-MS analyses are reported below. Assignments of Fe-O, Fe(OH), Fe(OH_2), Fe(PO_4) bands are based on reference samples (see above) and on the literature.[22,23] Aborptions around 1000 cm^{-1} in solids obtained from DMEM-C were tentatively assigned to Fe(PO_4) due to the residual phosphate content of the medium (0.109 g/L Na_2HPO_4, corresponding to 0.77 mM).[24] Bands due to carbonyl (CO) or isocyanide (CN) ligands are underlined. An IR band around 1600 cm^{-1} corresponding to water in CH_2Cl_2 is sometimes present (not reported).

Table S13. CHNS content and IR analyses of solids obtained from solutions of [**1**]$^+$ or $Fe^{2+}_{(aq)}$ (from $FeSO_4$ or Mohr's salt) in water or cell culture media incubated at 37 °C (except where otherwise noted) for several days.

Exp.	Starting material	Solution, concentration, incubation time: solution	c^0_{Fe2} / mM	t / days	Elemental composition [a]: %C	%H	%N	%S	% other	IR (solid):[b] CO, CNR bands (Y/N)?	IR (CH_2Cl_2):[b] CO, CNR bands (Y/N)?	Note
S13-1	$FeSO_4$	H_2O	6.1	11	/	/	/	/	/	/	*n.r.*	[c]
S13-2		DMEM-C	5.8	11	10.8	2.2	1.3	0.7	87	N	*n.r.*	
S13-3	Mohr's salt $(NH_4)_2Fe(SO_4)_2 \cdot 6H_2O$,	H_2O	3.3	7	/	/	/	/	/	/	*n.r.*	[d]
S13-4		DMEM-C-dil	3.3	7	2.6	0.54	< LOD	< LOD	97	N	*n.r.*	
S13-5		DMEM-C	3.7	11	11.9	3.20	2.29	< LOD	83	*n.r.*	*n.r.*	
S13-6			3.2	18.5	13.1	3.3	2.29	< LOD	81	N	*n.r.*	
S13-7	[**1a**]CF_3SO_3	H_2O	3.4	7	10.2	2.1	0.3	< *LOD*	87	**N**	**N**	
S13-8		H_2O	3.5	17	9.1	1.8	0.2	< *LOD*	89	**N**	**N**	[e]
S13-9		DMEM-C-dil	3.4	10	8.9	2.2	1.3	< *LOD*	88	**N**	**N**	
S13-10		DMEM-P	3.4	8	16.6	2.6	3.7	< *LOD*	77	**N**	**N**	
S13-11	[**1b**]NO_3	H_2O	3.3	10	13.8	2.3	0.3	< *LOD*	84	**N**	**N**	
S13-12		DMEM-C	3.1	12	12.2	3.0	1.5	< *LOD*	83	**Y** (very weak)	**Y** (very weak)	
S13-13	[**1b**]CF_3SO_3	H_2O	≈ 3.6 [f]	7	8.4	1.3	0.3	< *LOD*	90	**N**	**N**	
S13-14		H_2O	≈ 3.6 [f]	7	8.2	2.0	0.4	< *LOD*	89	**N**	*n.r.*	[e]
S13-15		DMEM-C-dil	≈ 3.6 [f]	10	8.1	2.3	0.4	< *LOD*	89	**N**	**N**	
S13-16		DMEM-C	≈ 3.6 [f]	10	12.7	3.2	1.9	0.5	82	**N**	**N**	
S13-17		DMEM-P	≈ 3.6 [f]	8	6.8	2.4	0.6	< *LOD*	90	**Y** (very weak)	**Y** (very weak)	
S13-18	[**1c**]CF_3SO_3	H_2O	≈ 1.8 [f]	7	6.0	0.8	0.3	< *LOD*	93	**N**	**N**	
S13-19		H_2O	≈ 1.8 [f]	10	2.6	0.5	< *LOD*	< *LOD*	97	**N**	**N**	[e]

S13-20		DMEM-C-dil	≈ 1.8 [f]	10	19.9	2.9	2.3	< *LOD*	75	Y	Y	
S13-21		DMEM-C	≈ 1.8 [f]	10	21.6	1.7	1.1	< *LOD*	83	Y	Y	
S13-22		DMEM-P	≈ 1.8 [f]	8	20.7	3.1	4.1	< *LOD*	72	Y	Y	
S13-23	[**1c**]NO_3	H_2O	3.8	17	11.4	1.8	0.4	0.5	86	N	N	
S13-24		DMEM-C-dil	12	7	18.0	2.9	1.3	< *LOD*	78	*n.r.*	Y (weak)	
S13-25	[**1e**]CF_3SO_3	H_2O	1.5	10	21.8	1.5	0.4	< *LOD*	76	Y	Y (weak)	
S13-26		DMEM-C	1.8	11	18.2	3.5	2.0	0.67	76	Y	Y	
S13-27	[**1e**]NO_3	H_2O	4.2	12	22.1	2.9	1.1	< *LOD*	74	Y	Y	
S13-28		DMEM-C-dil	4.2	12	27.3	3.5	2.0	< *LOD*	67	Y	Y	
S13-29		DMEM-C-dil	8.4	8	19.5	2.6	1.3	< *LOD*	77	Y	Y	**60 °C**
					−29 %	*−27 %*	*−32 %*					[g]
S13-30		DMEM-C	8.4	10	30.2	4.0	3.0	0.84	62	Y	Y	
S13-31	[**1f**]CF_3SO_3	H_2O	≈ 1.4 [f]	12	24.2	3.0	1.5	< *LOD*	71	Y	Y	
S13-32		DMEM-C-dil	≈ 1.4 [e]	12	18.3	3.1	1.8	< *LOD*	77	Y	Y	

[a] Data rounded to the first decimal place. [b] IR absorptions are listed below. [c] Only a trace amount of yellow precipitate was observed in the final reaction mixture, which could not be collected. [d] No precipitate was observed in the final solution. [f] Saturated solution. [g] Relative % variation in C, H or N content from 37 °C to 60 °C. *n.r.* = not recorded.

Figure S54. Raman spectra (125-1250 cm^{-1}) of commercial α-Fe_2O_3 (**A**), FeO(OH) obtained from $Fe_3(SO_4)_2$ + NaOH (**C**), $FePO_4$ obtained by $Fe_3(SO_4)_2$ + phosphate buffer (**D**) and the solids obtained from incubation of Mohr's salt (**B**), [**1b**]CF_3SO_3 (**E**), [**1c**]CF_3SO_3 (**F**), [**1a**]CF_3SO_3 (**G**) in water (black line) or in DMEM-C-dil (red line) – experimental details in Table S13.

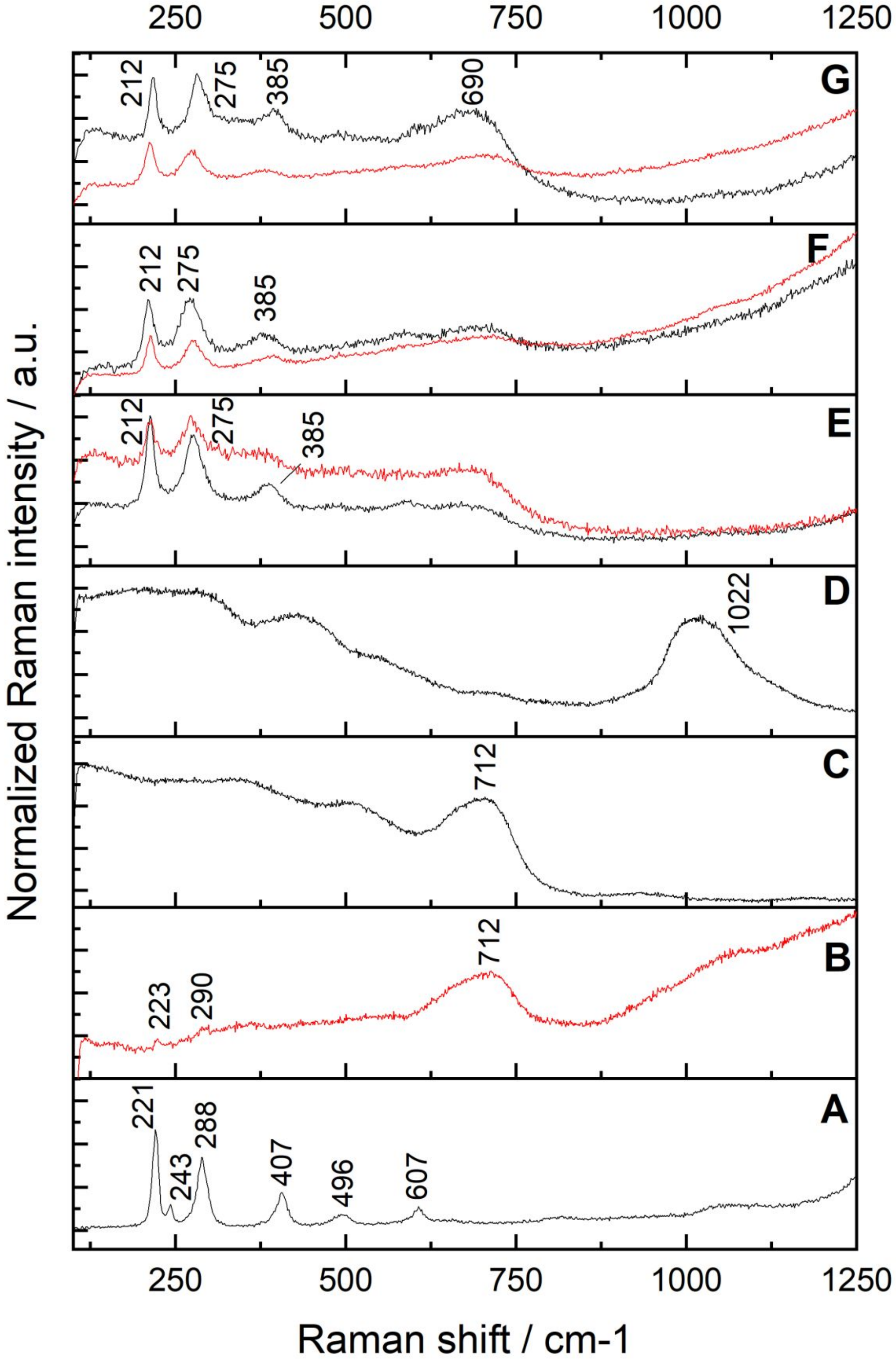

$FeSO_4$ in **DMEM-C** (exp. **S13-2**). IR (solid state): $\tilde{\upsilon}/cm^{-1}$ = 3277m-br (OH), 1629m (H_2O); 1389w, 1209m, 1151m-sh, 1006s-br (PO_4?), < 650s (Fe-O).

Mohr's salt in **DMEM-C-dil** (exp. **S13-4**). IR (solid state): $\tilde{\upsilon}/cm^{-1}$ = 3300w-br (OH), 1730w, 1645w (H_2O), 1440-1393w, 1046-982s (OH + PO_4?), 897s-sh (OH), 784s (OH), 670s (Fe-O). Raman (solid state): $\tilde{\upsilon}/cm^{-1}$ = 223vw, 290vw, 712s.

Mohr's salt in **DMEM-C** (exp. **S13-6**). IR (solid state): $\tilde{\upsilon}/cm^{-1}$ = 3209m-br (OH); 2923w, 1637m (H_2O), 1378w, 1261w, 1005s-br (PO_4?), 795m (OH), < 650s (Fe-O).

Figure S55. IR spectra (650-4000 cm^{-1}) of solids obtained from solutions of Mohr's salt in DMEM-C-dil (yellow line), Mohr's salt in DMEM-C (blue line) or $FeSO_4$ in DMEM-C (red line). The IR spectrum of $FePO_4 \cdot nH_2O$ is added for comparison (dashed black line – see also Figure S50).

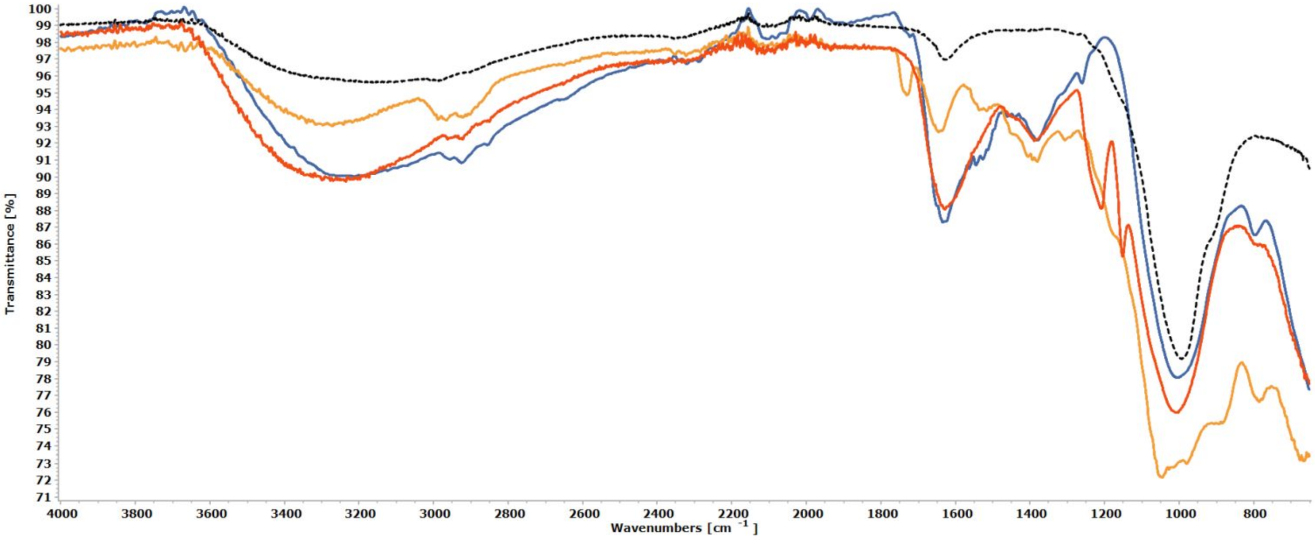

[**1a**]CF_3SO_3 in H_2O (exp. **S13-7**). IR (solid state): υ̃/cm^{-1} = 3370-3244m-br (OH), 1590m (H_2O), 1406m, 1263w; 906m-sh (OH), 794m-sh, < 650s (Fe-O). Raman (solid state): υ̃/cm^{-1} = 212s, 275s, 385m, 690m. IR (CH_2Cl_2): no absorptions.

[**1a**]CF_3SO_3 in H_2O (exp. **S13-8**). IR (solid state): identical to the previous one in the 1350-4000 cm^{-1} region; υ̃/cm^{-1} = 1308w, 1212w, 1153w, 1018w, < 650s (Fe-O). IR (CH_2Cl_2): no absorptions.

[**1a**]CF_3SO_3 in DMEM-C-dil (exp. **S13-9**). IR (solid state): υ̃/cm^{-1} = 3286m-br (OH), 1636m (H_2O), 1536m, 1368m, 1055m-sh, 1029m-sh; 947s-sh (OH), < 650s (Fe-O). Raman (solid state): υ̃/cm^{-1} = 212s, 275s. IR (CH_2Cl_2): no absorptions.

[**1a**]CF_3SO_3 in DMEM-P (exp. **S13-10**). IR (solid state): υ̃/cm^{-1} = 3278w-br (OH), 2915w, 1644m (H_2O), 1538m, 1393w, 1018s (PO_4). IR (CH_2Cl_2): no absorptions.

Figure S56. IR spectra (650-4000 cm^{-1}) of solids obtained from solutions of [**1a**]CF_3SO_3 in water (cyan line), DMEM-C-dil (yellow line) and DMEM-P (red line).

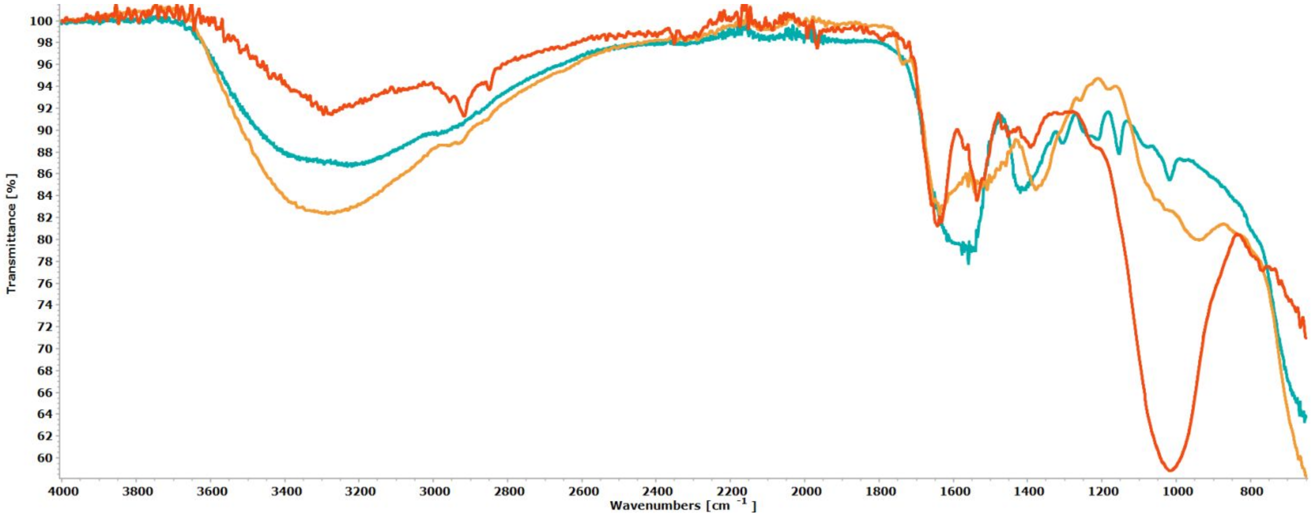

[**1b**]NO_3 in H_2O (exp. **S13-11**). IR (solid state): ṽ/cm^{-1} = 3250m-br (OH); 2923w; 2014w, 1595m-br (H_2O); 1418w, 1207s (*), 1151s (*), 1090m, 1051m, 1026m, 790m-sh (OH), < 650s (Fe-O); *PTFE impurity. IR (CH_2Cl_2): no absorptions.

[**1b**]NO_3 in DMEM-C (exp. **S13-12**). IR (solid state): ṽ/cm^{-1} = 3240w-br (OH); 2930w; 2012w, 1975vw, 1825w (CO); 1616m (H_2O); 1386m, 1085s-sh, 1008s (PO_4?), < 650s (Fe-O). IR (CH_2Cl_2): ṽ/cm^{-1} = 2017vw, 1983vw (CO).

[**1b**]CF_3SO_3 in H_2O (exp. **S13-13**). IR (solid state): ṽ/cm^{-1} = 3337m-br, 3172m-br (OH); 1624m-sh (H_2O), 1583m, 1409m, 1262w; 1019m, 906m, 801m-sh (OH). IR (CH_2Cl_2): no absorptions.

[**1b**]CF_3SO_3 in H_2O (exp. **S13-14**). IR (solid state): ṽ/cm^{-1} = 3300w-br (OH); 1617m (H_2O); 1420m, 1008m (OH). Raman (solid state): ṽ/cm^{-1} = 212s, 275s, 385m.

[**1b**]CF_3SO_3 in DMEM-C-dil (exp. **S13-15**). IR (solid state): ṽ/cm^{-1} = 3350-3250m-br (OH); 1607m (H_2O); 1376m, 1082w-sh; 1029m-sh, 943s-sh (OH). Raman (solid state): ṽ/cm^{-1} = 212s, 275s. IR (CH_2Cl_2): no absorptions.

[**1b**]CF_3SO_3 in DMEM-C (exp. **S13-16**). IR (solid state): ṽ/cm^{-1} = 3337m-br, 3205m-br (OH); 1601m (H_2O); 1379m, 1076s-sh; 989s (PO_4?), 960s-sh (OH). IR (CH_2Cl_2): no absorptions.

[**1b**]CF_3SO_3 in DMEM-P (exp. **S13-17**). IR (solid state): ṽ/cm^{-1} = 3330-3200w-br (OH); 2987w, 2926w; 2012vw, 1970vw (CO); 1637w (H_2O); 1447w, 998s (PO_4). IR (CH_2Cl_2): ṽ/cm^{-1} = 2016vw, 1979-1971vw (CO).

Figure S57. IR spectra (650-4000 cm^{-1}) of solids obtained from solutions of [**1b**]CF_3SO_3 in water (cyan line), DMEM-C-dil (yellow line), DMEM-C (blue line) and DMEM-P (red line).

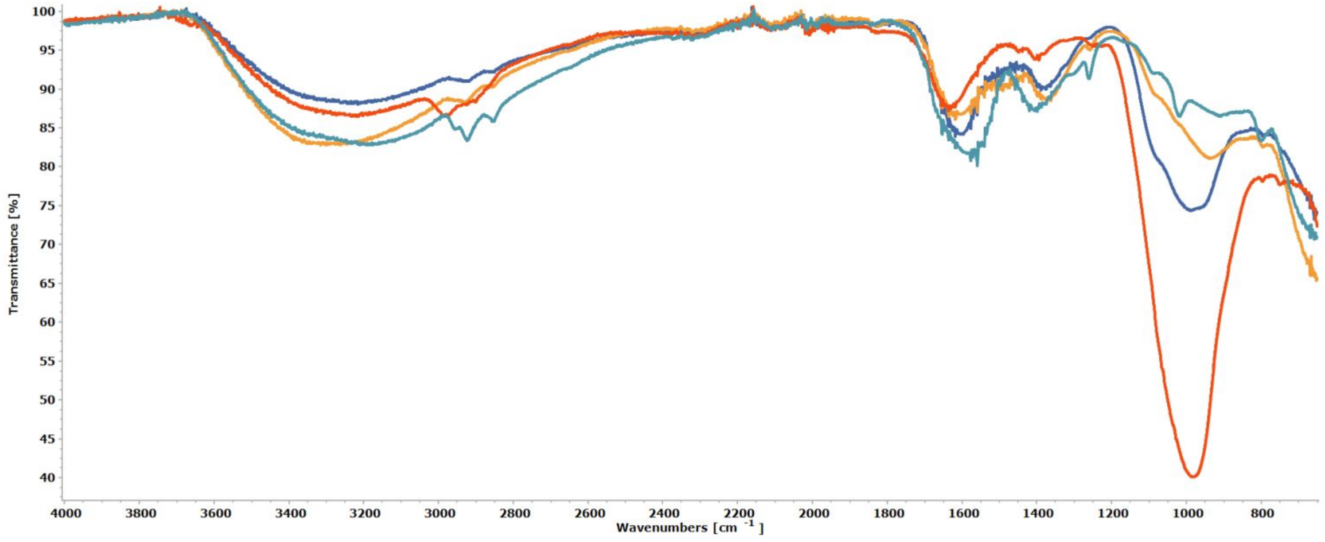

Figure S58. IR spectra (650-4000 cm^{-1}) of solids obtained from solutions of [**1b**]NO_3 (green line) or [**1b**]CF_3SO_3 (blue line) in DMEM-C.

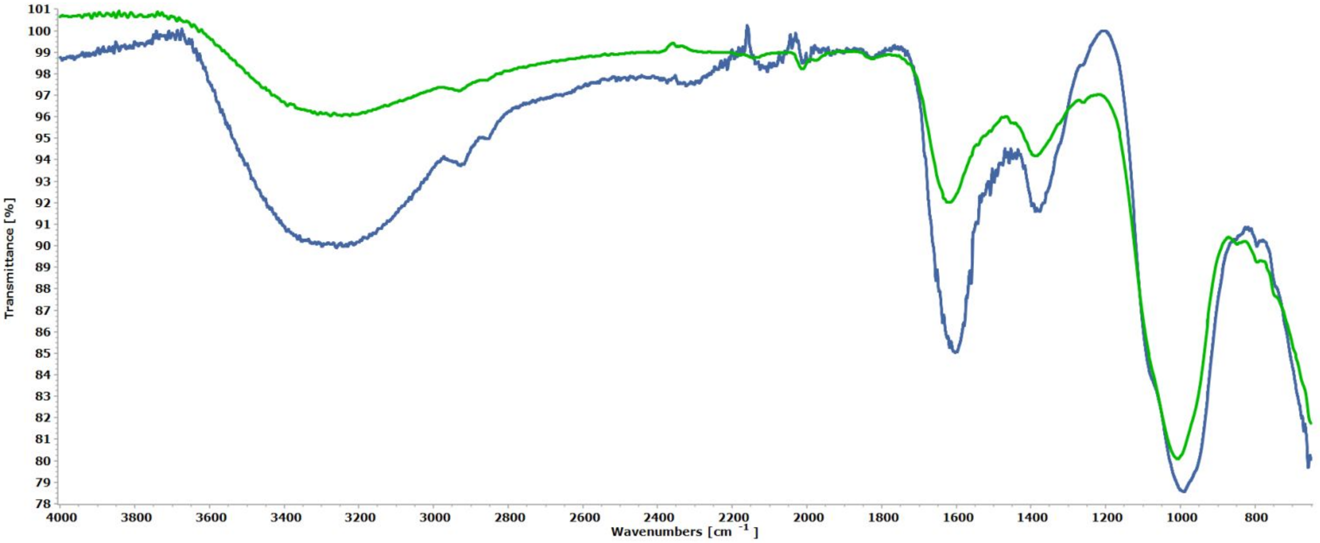

[**1c**]CF_3SO_3 in H_2O (exp. **S13-18**). IR (solid state): $\tilde{\upsilon}$/cm^{-1} = 3300-3100w-br (OH); 1622w (H_2O); 1402w, 1001s-br (OH), 794m (OH), 702m. Raman (solid state): $\tilde{\upsilon}$/cm^{-1} = 212s, 275s, 385m. IR (CH_2Cl_2): no absorptions.

[**1c**]CF_3SO_3 in H_2O (exp. **S13-19**). IR (solid state): identical to the previous one. IR (CH_2Cl_2): no absorptions.

[**1c**]CF_3SO_3 in DMEM-C-dil (exp. **S13-20**). IR (solid state): $\tilde{\upsilon}$/cm^{-1} = 3230s-br (OH); 2192w-sh, 2154w (CN); 2009w, 1977w, 1810w (CO); 1635m (H_2O); 1540m 1474m, 1386m, 1252w, 1072s, 967s (OH). Raman (solid state): $\tilde{\upsilon}$/cm^{-1} = 212s, 275s. IR (CH_2Cl_2): $\tilde{\upsilon}$/cm^{-1} = 2198w, 2159m (CN); 2013w, 1977w, 1803w-br (CO); 1646m-br.

[**1c**]CF_3SO_3 in DMEM-C (exp. **S13-21**). IR (solid state): $\tilde{\upsilon}$/cm^{-1} = 3300w-br (OH); 2193w, 2152w, 2010w, 1974vw, 1829vw, 1622m (H_2O); 1206s (*), 1150s (*), 1014s-br (PO_4?), 700m; *PTFE impurity. IR (CH_2Cl_2): $\tilde{\upsilon}$/cm^{-1} = 2199w, 2158m, 2015w, 1981w, 1825w-br, 1738m.

[**1c**]CF_3SO_3 in DMEM-P (exp. **S13-22**). IR (solid state): $\tilde{\upsilon}$/cm^{-1} = 3272m-br (OH); 2956w, 2923w, 2853w, 2188w, 2153w (CN); 2011w-sh, 1973w, 1800w (CO); 1637m (H_2O); 1534m, 1438w, 1389w, 1047s-sh, 1000s-br (PO_4), 769w, 734w, 698w. IR (CH_2Cl_2): $\tilde{\upsilon}$/cm^{-1} = 2199w, 2160m (CN); 2015w-sh, 1981w, 1813w (CO); 1667w.

[**1c**]NO_3 in H_2O (exp. **S13-23**). IR (solid state): $\tilde{\upsilon}$/cm^{-1} = 3360-3220m-br (OH); 1609m-sh (H_2O), 1560m; 1414m, 1306w, 1210w, 1153w, 1017w, 680s-sh (Fe-O). IR (CH_2Cl_2): no absorptions.

[**1c**]NO_3 in DMEM-C-dil (exp. **S13-24**). IR (CH_2Cl_2): $\tilde{\upsilon}$/cm^{-1} = 2162-2145w (CN); 2015vw, 1980w, 1809w-br (CO); 1634m-br.

Figure S59. IR spectra (650-4000 cm^{-1}) of solids obtained from solutions of [**1c**]CF_3SO_3 in water (cyan line), DMEM-C-dil (yellow line), DMEM-C (blue line*) and DMEM-P (red line). *IR absorptions at 1205 and 1150 cm^{-1} are due to a PTFE impurity.

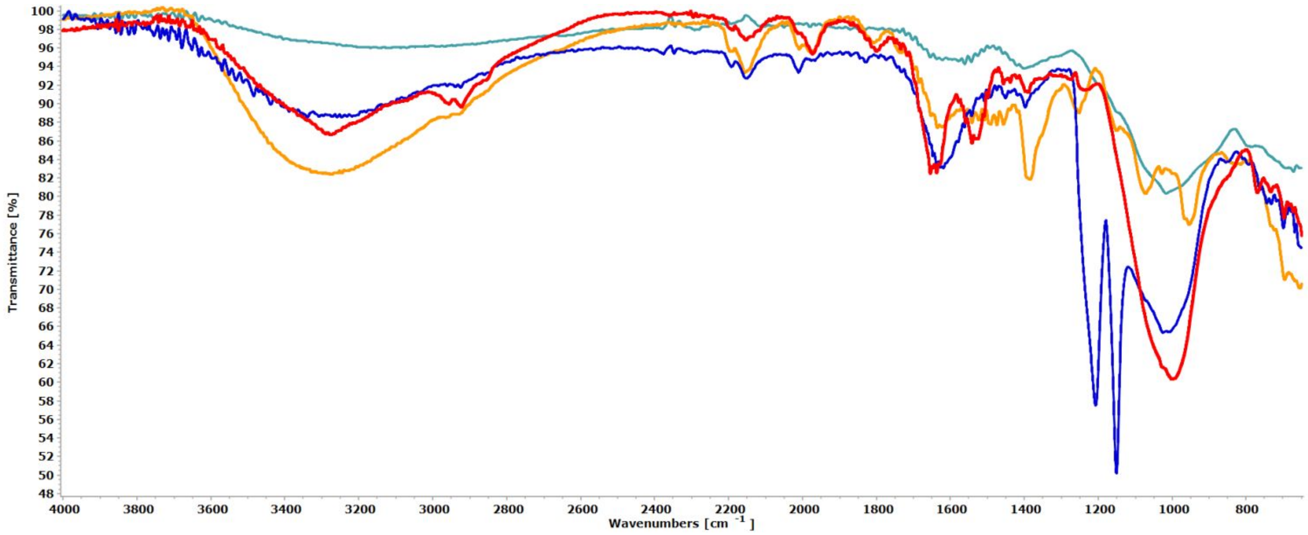

Figure S60. IR spectra (650-4000 cm^{-1}) of solids obtained from solutions of [**1c**]NO_3 (green line) or [**1c**]CF_3SO_3 (cyan line) in water.

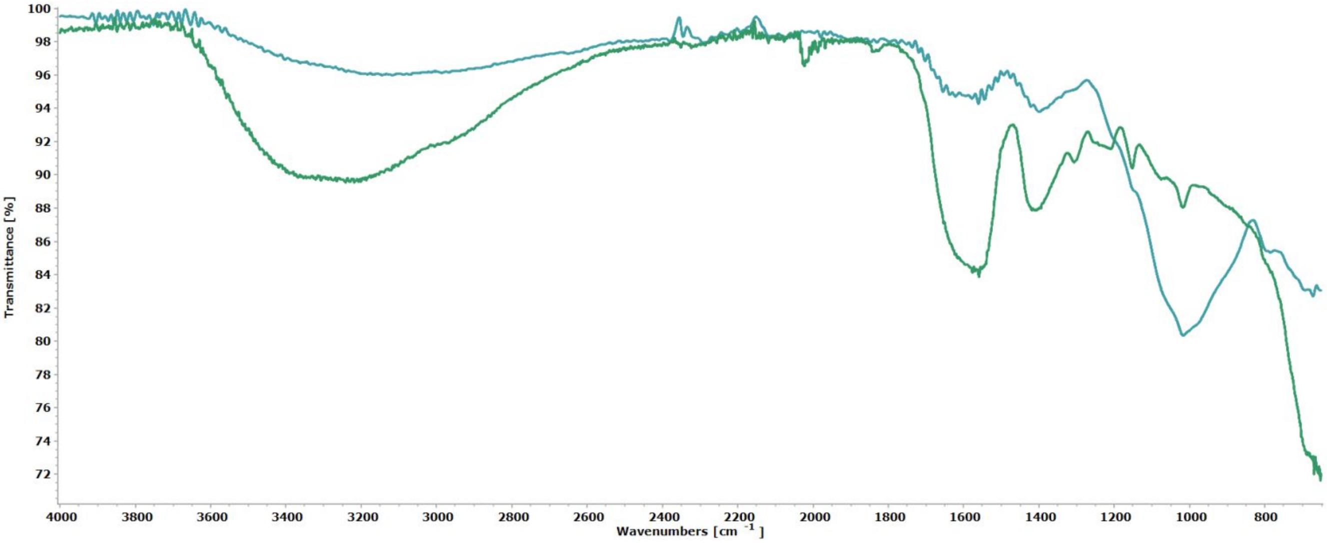

[**1e**]CF_3SO_3 in H_2O (exp. **S13-25**). IR (solid state): $\tilde{\upsilon}$/cm^{-1} = 3260w-br (OH); 2959w, 2922w; 2114w (CN); 2016m, 1988w, 1832w (CO); 1630w-br (H_2O), 797m-sh, < 650s (Fe-O)

[**1e**]CF_3SO_3 in DMEM-C (exp. **S13-26**). IR (solid state): $\tilde{\upsilon}$/cm^{-1} = 3274m-br (OH); 2979w; 2158w, 2113w (CN); 2014w, 1974w, 1830w (CO), 1618m (H_2O); 1379w, 1084s-sh, 1008s (PO_4?), 764w. IR (CH_2Cl_2): $\tilde{\upsilon}$/cm^{-1} = 2166w, 2123m (CN); 2020m, 1984m, 1830w, 1798w (CO).

[**1e**]NO_3 in H_2O (exp. **S13-27**). IR (solid state): $\tilde{\upsilon}$/cm^{-1} = 3330m-br (OH); 2161w, 2112m (CN); 2016w, 1977w, 1816w (CO); 1598s-br (H_2O); 1420m, 1377m, 1086w, 1017w, < 650s (Fe-O). IR (CH_2Cl_2): $\tilde{\upsilon}$/cm^{-1} = 2167m, 2124s (CN); 1983w (CO).

[**1e**]NO_3 in DMEM-C-dil (exp. **S13-28**). IR (solid state): $\tilde{\upsilon}$/cm^{-1} = 3370w-br (OH); 2162w, 2111m (CN); 2009m, 1975m, 1812m (CO); 1618m (H_2O), 1527m, 1474m, 1376m, 1083w; 841m, 771s; < 650s (Fe-O). IR (CH_2Cl_2): $\tilde{\upsilon}$/cm^{-1} = 2167w, 2122s (CN); 2018w, 1985m, 1824m (CO); 1708w.

[**1e**]NO_3 in DMEM-C-dil at 60 °C (exp. **S13-29**). IR (solid state): qualitatively similar to the previous one, all absorptions have a lower intensity. IR (CH_2Cl_2): $\tilde{\upsilon}$/cm^{-1} = 2167w, 2120m (CN); 2019w, 1988w, 1825w (CO), 1715m-br.

[**1e**]NO_3 in DMEM-C (exp. **S13-30**). IR (solid state): $\tilde{\upsilon}$/cm^{-1} = 3200w-br (OH); 2113w (CN); 2013m, 1973m, 1826m (CO); 1616m (H_2O); 1523m, 1473m, 1374s, 1082s, 1007s (PO_4?), 950s-sh, 771s, 734s, 664s. IR (CH_2Cl_2): $\tilde{\upsilon}$/cm^{-1} = 2168w, 2121m (CN); 2019m, 1982m, 1821-1801m (CO); 1706w, 1630-1614m.

Figure S61. IR spectra (650-4000 cm^{-1}) of solids obtained from solutions of [**1e**]NO_3 in water (cyan line), DMEM-C-dil (yellow line), DMEM-C (red line).

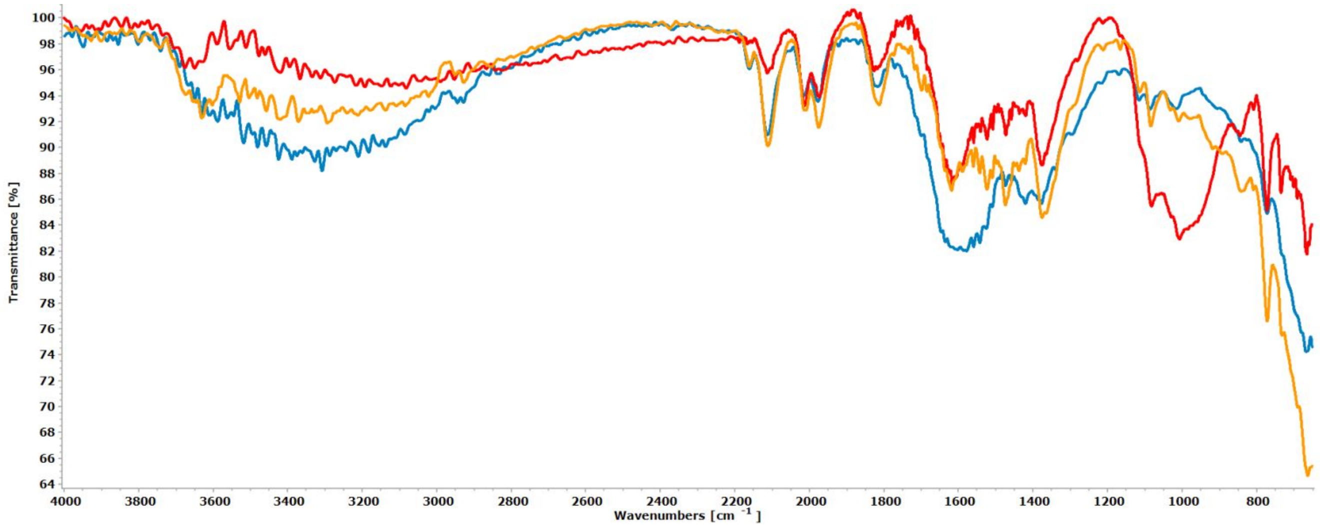

Figure S62. IR spectra (650-4000 cm^{-1}) of solids obtained from solutions of [**1e**]NO_3 (green line) or [**1e**]CF_3SO_3 (red line) in DMEM-C.

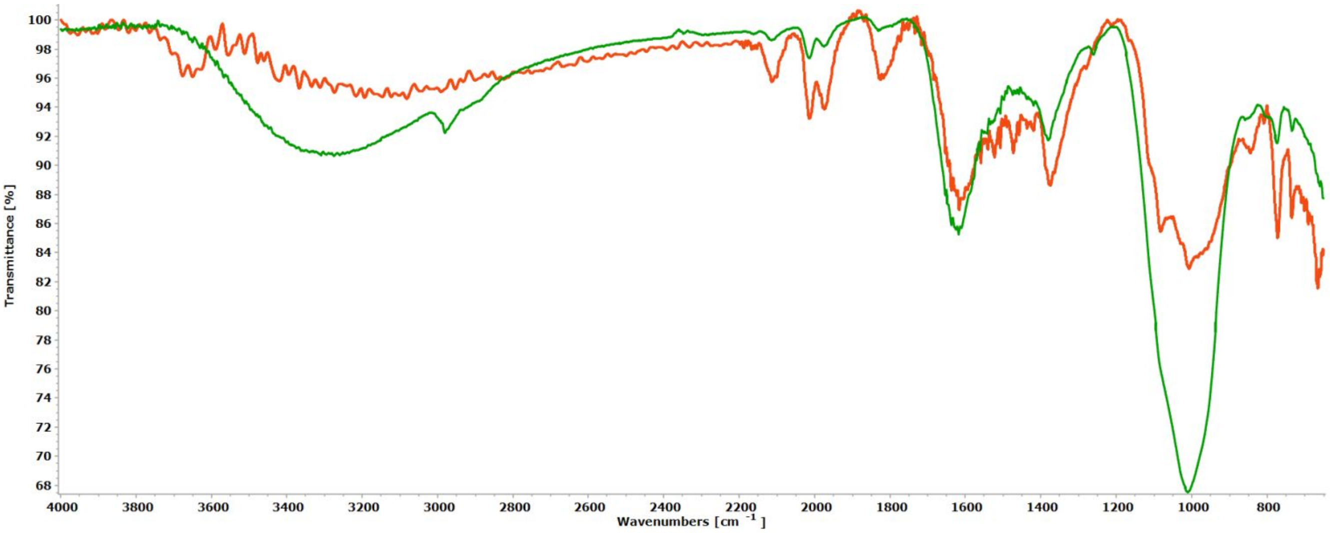

[**1f**]CF_3SO_3 in H_2O (exp. **S13-31**). IR (solid state): $\tilde{\upsilon}$/cm^{-1} = 3400-3200w-br (OH); 2170w, 2130m (CN); 2018m, 1981w, 1825w (CO); 1617m-br (H_2O), 1508s, 1396m-br, 1249m, 1173w, 1106w, 1032m, 834m, ≤ 690s (Fe-O). IR (CH_2Cl_2): $\tilde{\upsilon}$/cm^{-1} = 2173w, 2133s (CN); 2021m, 1984m, 1834w (CO); 1709w, 1510s, 1466w.

[**1f**]CF_3SO_3 in DMEM-C-dil (exp. **S13-32**). IR (solid state): $\tilde{\upsilon}$/cm^{-1} = 3400-3200w-br (OH); 2168w, 2127w (CN); 2010m, 1980w, 1826w (CO); 1635m-br (H_2O), 1509m, 1379m-br, 1246m, 1077m, 1028m, 962m-sh, 942m, 836m, < 650s (Fe-O). IR (CH_2Cl_2): $\tilde{\upsilon}$/cm^{-1} = 2175w, 2133s (CN); 2019m, 1984m, ≈ 1835w (CO); 1742w, 1709w, 1512s, 1467w, 1443w, 1424w.

Figure S63. IR spectra (650-4000 cm^{-1}) of solids obtained from solutions of [**1f**]CF_3SO_3 in water (cyan line) or DMEM-C-dil (orange line).

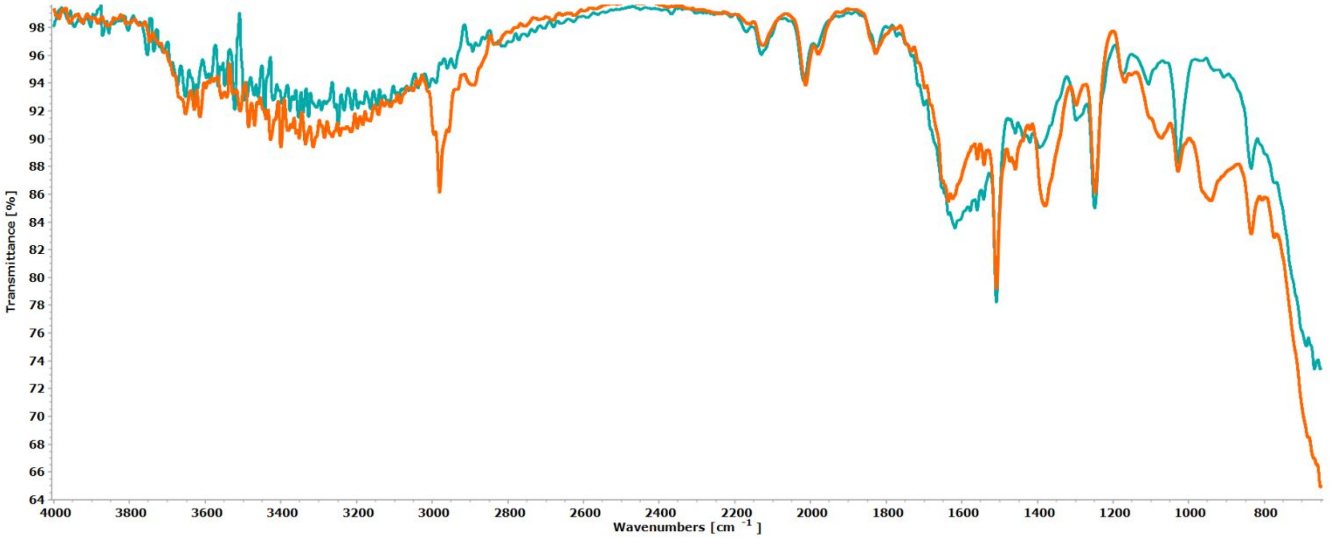

Figure S64. Comparison of IR spectra (CH_2Cl_2, solvent-subtracted, 1500-2275 cm^{-1}): solids obtained from solutions of [**1c**]CF_3SO_3 in water at 37 °C (cyan line), DMEM-C-dil (orange line), DMEM-C (blue line), DMEM-P (red line); [**1c**]CF_3SO_3 (dashed black line), **3c** (dashed green line). The absorption at 1600 cm^{-1} is due to water.

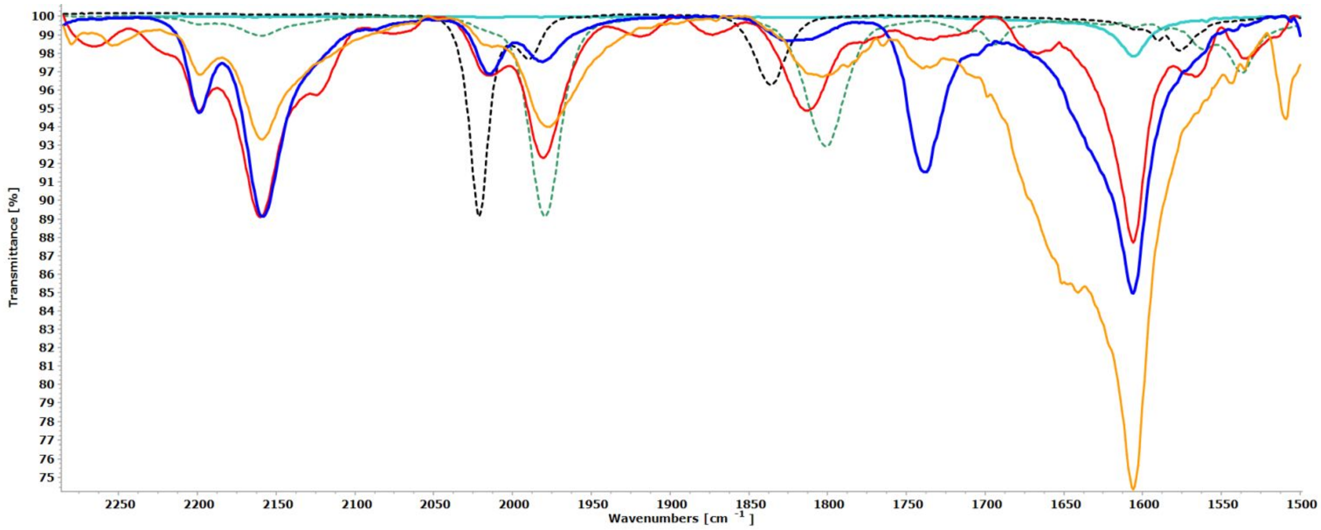

Figure S65. Comparison of IR spectra (CH_2Cl_2, solvent-subtracted, 1500-2275 cm^{-1}): solids obtained from solutions of [**1e**]NO_3 in water at 37 °C (cyan line), DMEM-C-dil at 37 °C (yellow line) or 60 °C (bordeaux line), DMEM-C (red line); [**1e**]NO_3 (dashed black line), **3e** (dashed green line). The absorption at 1600 cm^{-1} is due to water.

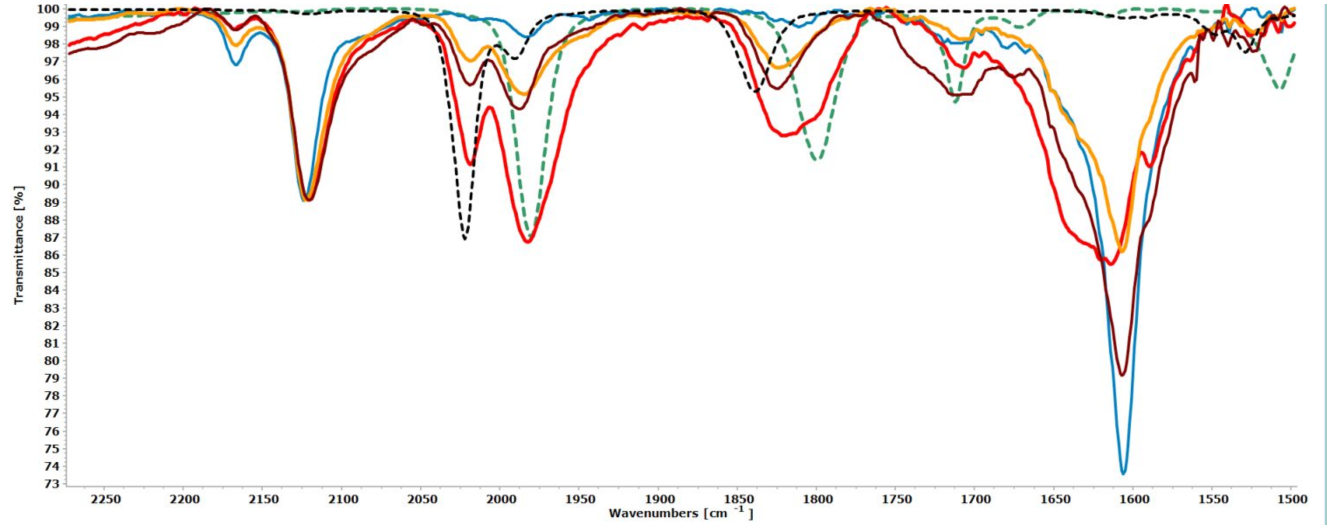

Figure S66. Comparison of IR spectra (CH_2Cl_2, solvent-subtracted, 1500-2275 cm^{-1}): solid obtained from a solution of [**1f**]CF_3SO_3 in water (cyan line) or DMEM-C-dil at 37 °C (orange line); [**1f**]CF_3SO_3 (dashed black line), **3f** (dashed green line). The absorption at 1600 cm^{-1} is due to water.

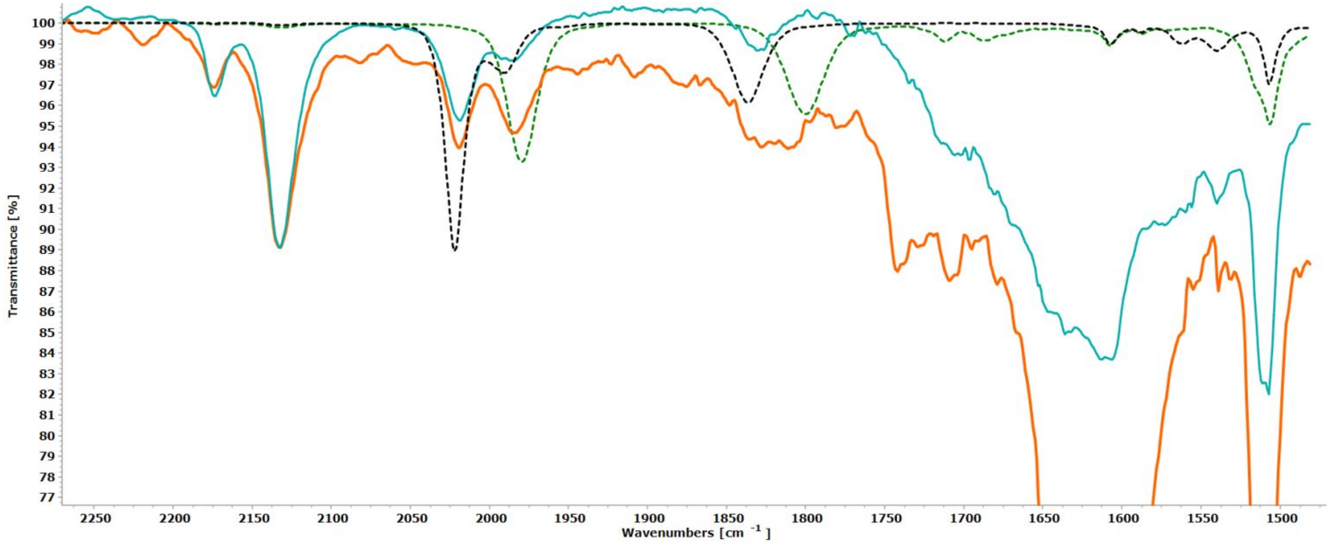

Figure S67. FIA-ESI(+)-MS spectra (MeOH, 100-1000 m/Z range) of the CH_2Cl_2-soluble fraction of the precipitate formed from solutions of [**1c**]NO_3 (top, blue line), [**1e**]NO_3 (middle, green line) or [**1f**]CF_3SO_3 (bottom, red line) in DMEM-C-dil over several days at 37 °C.

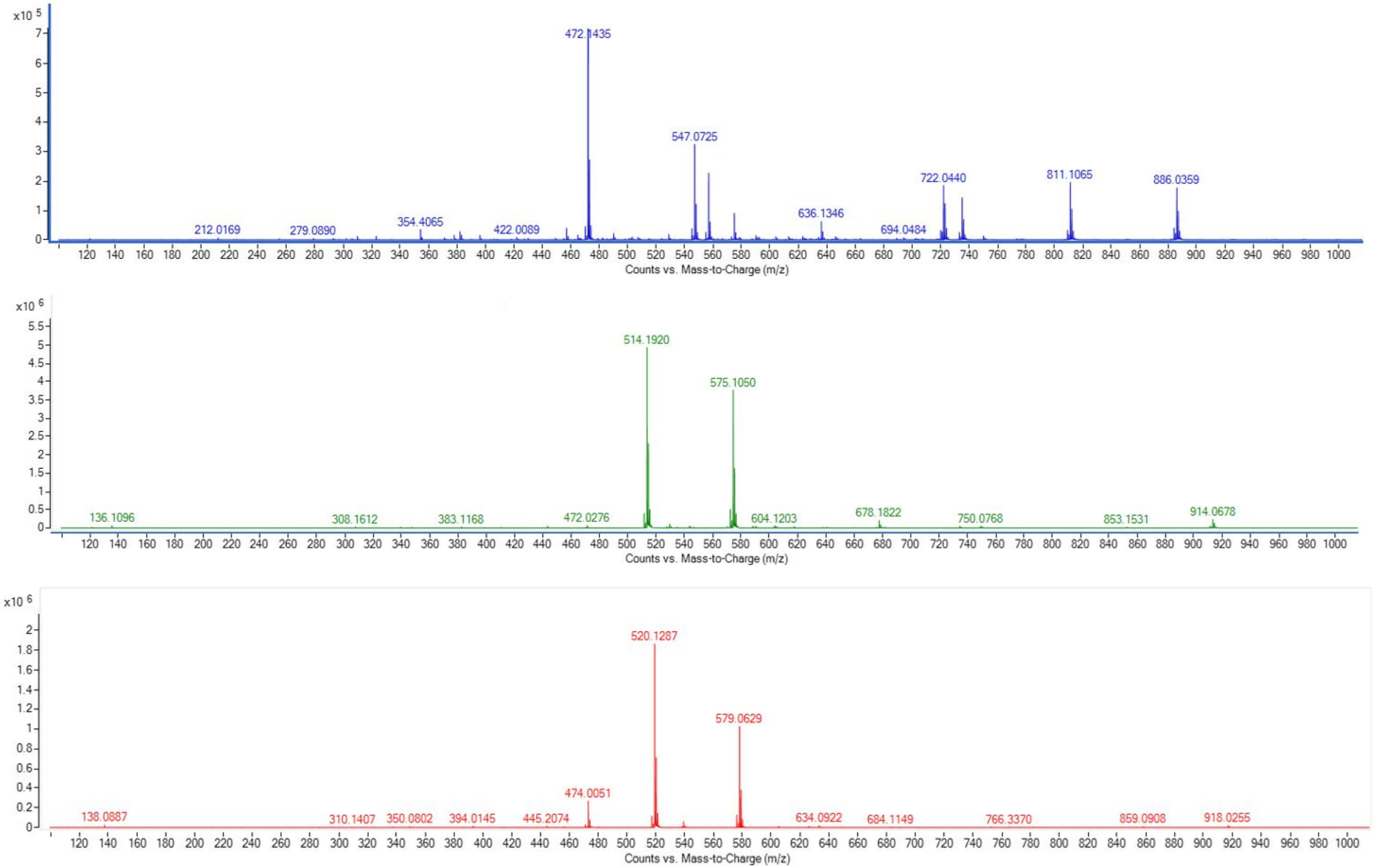

Figure S68. Comparison between experimental MS spectrum (left) and simulated isotopic patterns (right) of the CH_2Cl_2-soluble fraction of the precipitate formed over several days at 37 °C from a solution of [**1c**]NO_3 in DMEM-C-dil. Major species in **bold** (m/Z: **472.143**, **547.073**, **556.919**, 574.953, 636.135, **722.044**, 735.076, **811.107**, **886.036**).

Calcd. base peak for $[FeCp(CNBn)_3]^+$ = $C_{29}H_{26}FeN_3$: **472.148** Da.

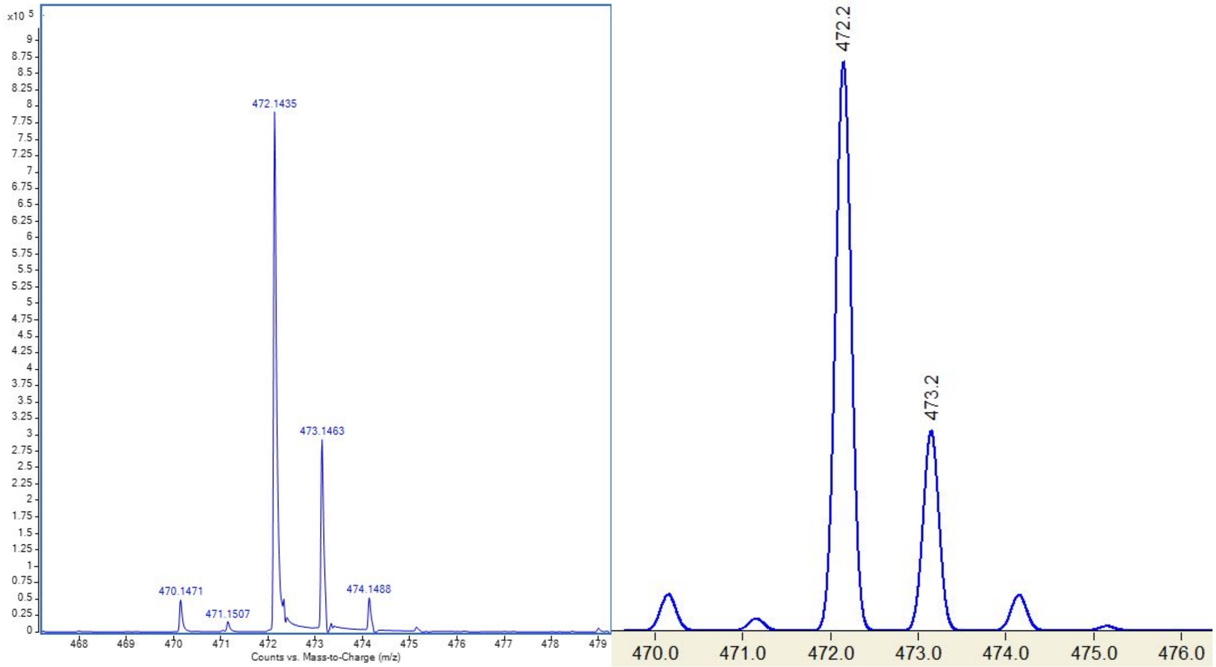

Calcd. base peak for $[Fe_2Cp_2(CO)_2(CNBn)(CNMeBn)]^+$ = $C_{29}H_{27}Fe_2N_2O_2$: **547.086** Da.

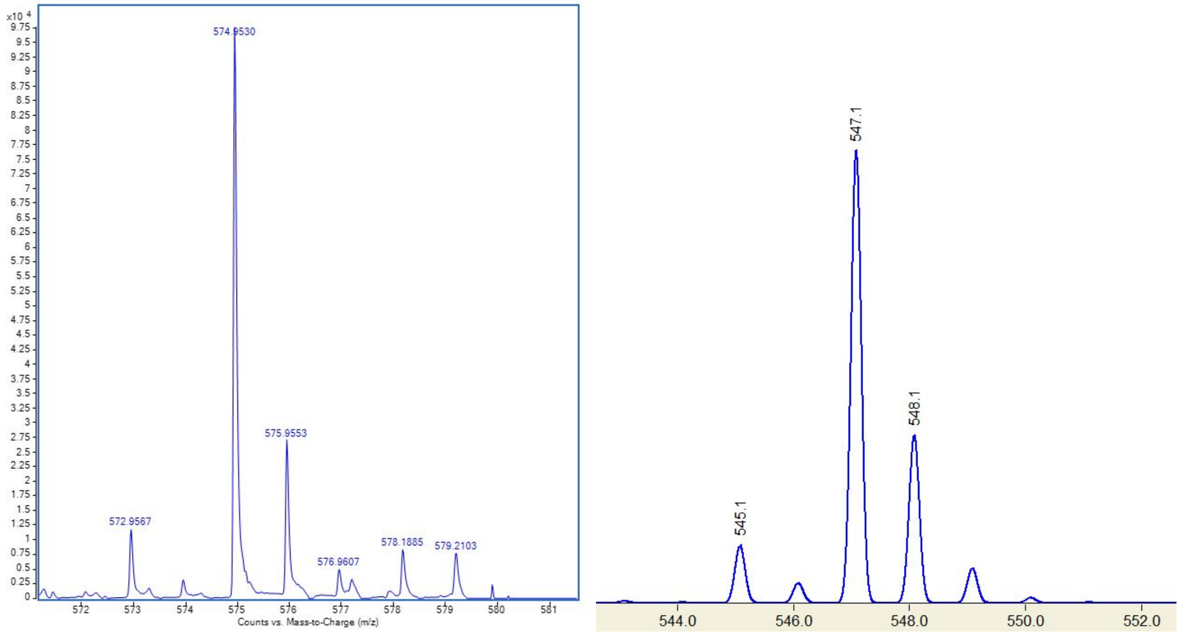

Calcd. base peak for [$Fe_2Cp_2(CO)(CNBn)_2(CNMeBn)]^+$ = $C_{36}H_{34}Fe_2N_3O$: 636.149 Da.

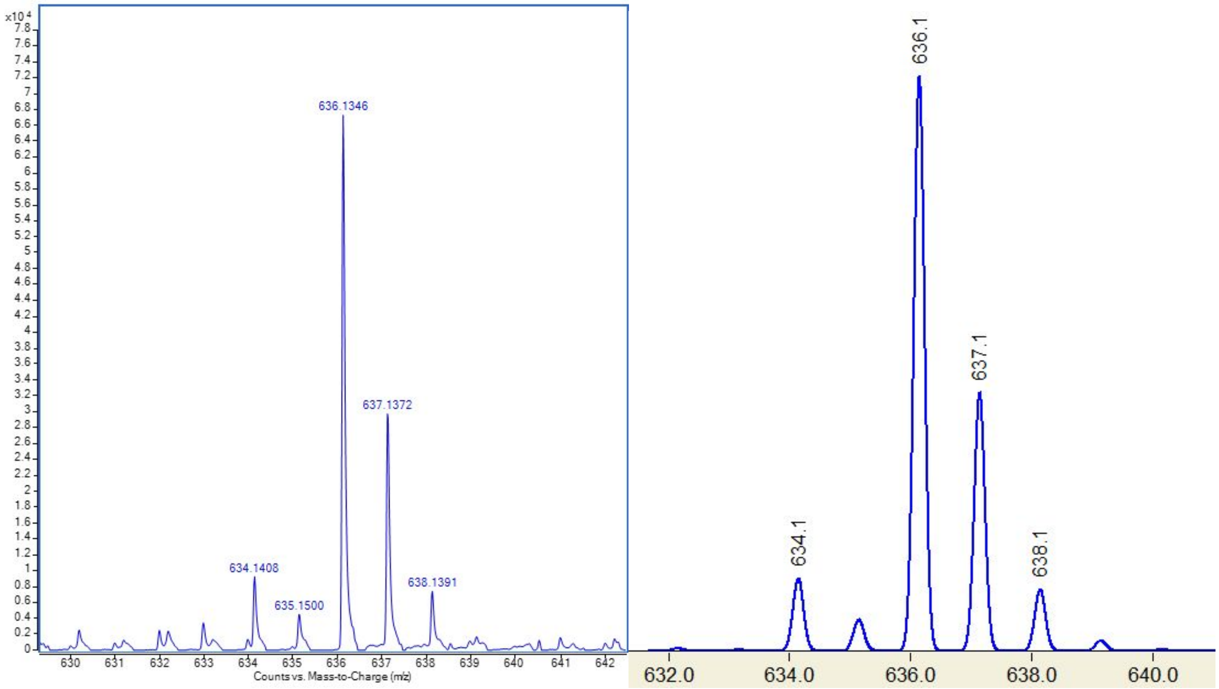

Calcd. base peak for [$Fe_4Cp_4H_2(CO)(CNBn)_2(CNMeBn)]^+$ = $C_{46}H_{52}Fe_4N_3O$: 886.169 Da.

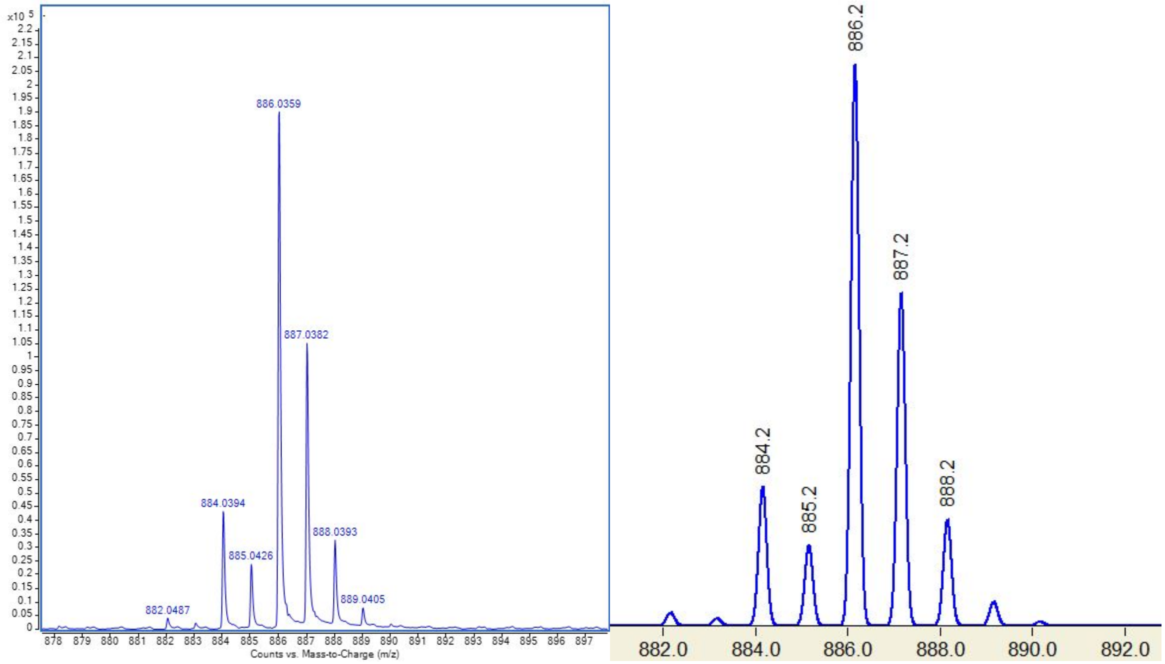

Unidentified clusters (base peak): 556.919; 722.044; 735.075 Da.

Calcd. base peak for [$Fe_2Cp_2(CO)_3(CNMeBn)]^+$, [**1c**]$^+$ = $C_{22}H_{20}Fe_2NO_3$: 458.014 Da (not present), [$Fe_2Cp_2Cl(CO)_2(CNMeBn)$], **3c** = $C_{21}H_{20}ClFe_2NO_2$: 464.988 Da (not present, as H^+ adduct)

Figure S69. Comparison between experimental MS spectrum (left) and simulated isotopic patterns (right) of the CH_2Cl_2-soluble fraction of the precipitate formed over several days at 37 °C from a solution of [**1e**]NO_3 in DMEM-C-dil. Major species in **bold** (m/Z = **514.192**, **575.105**, 678.182, 914.068).

Calcd. base peak for $[Fe_2Cp_2(CO)_3(CNMeXyl)]^+$, $[$**1e**$]^+$ = $C_{23}H_{22}Fe_2NO_3$: 472.030 Da.

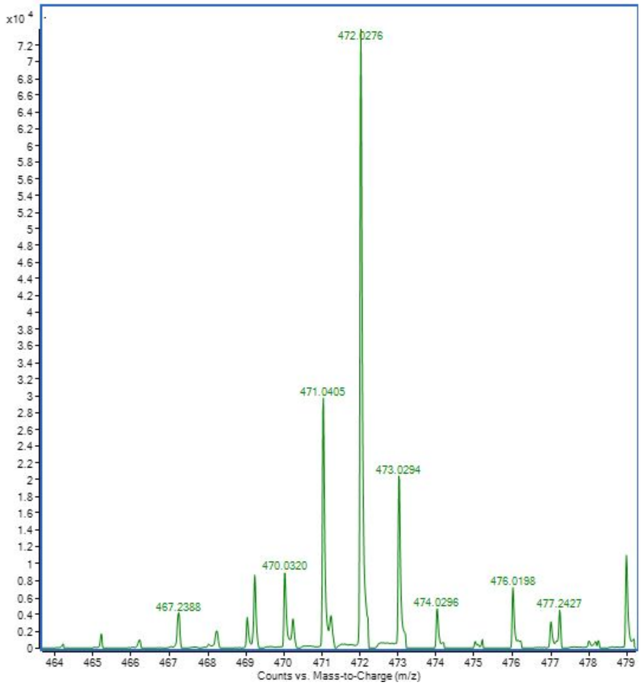

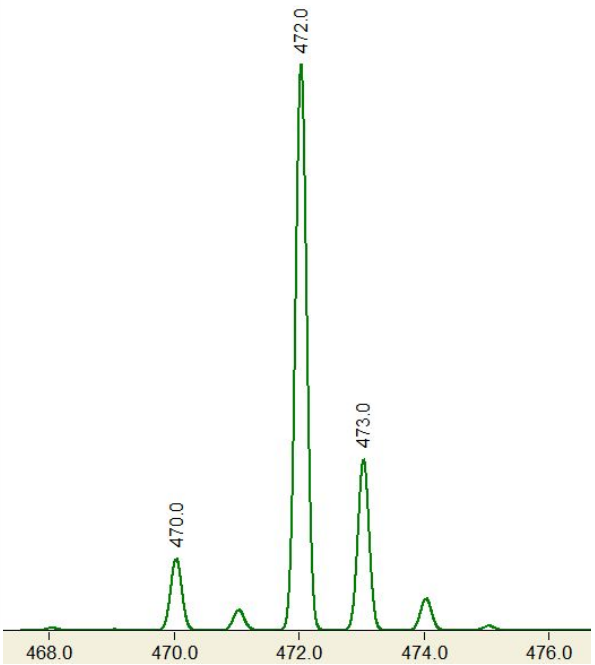

Calcd. base peak for $[FeCp(CNXyl)_3]^+$ = $C_{32}H_{32}FeN_3$: **514.462** Da.

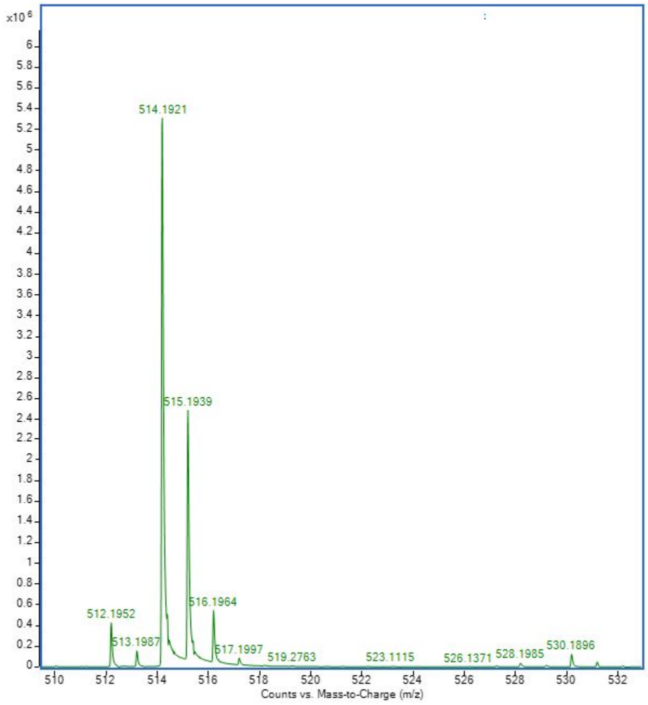

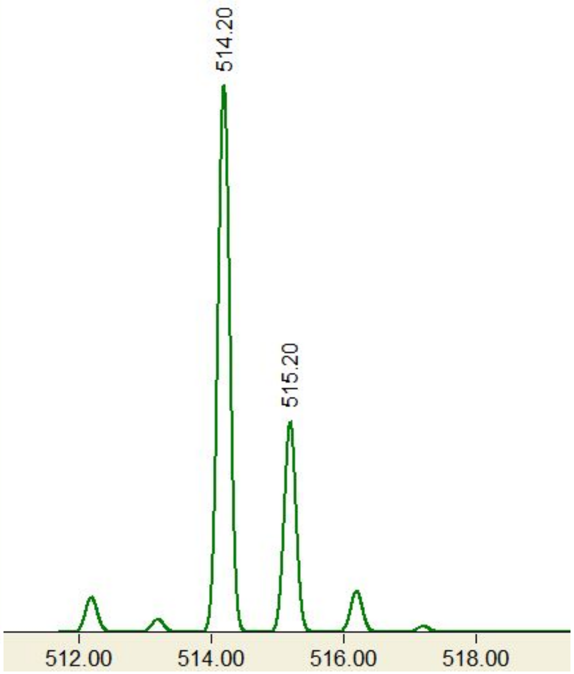

Calcd. base peak for $[Fe_2Cp_2(CO)_2(CNXyl)(CNMeXyl)]^+$ = $C_{31}H_{31}Fe_2N_2O_2$: **575.118** Da.

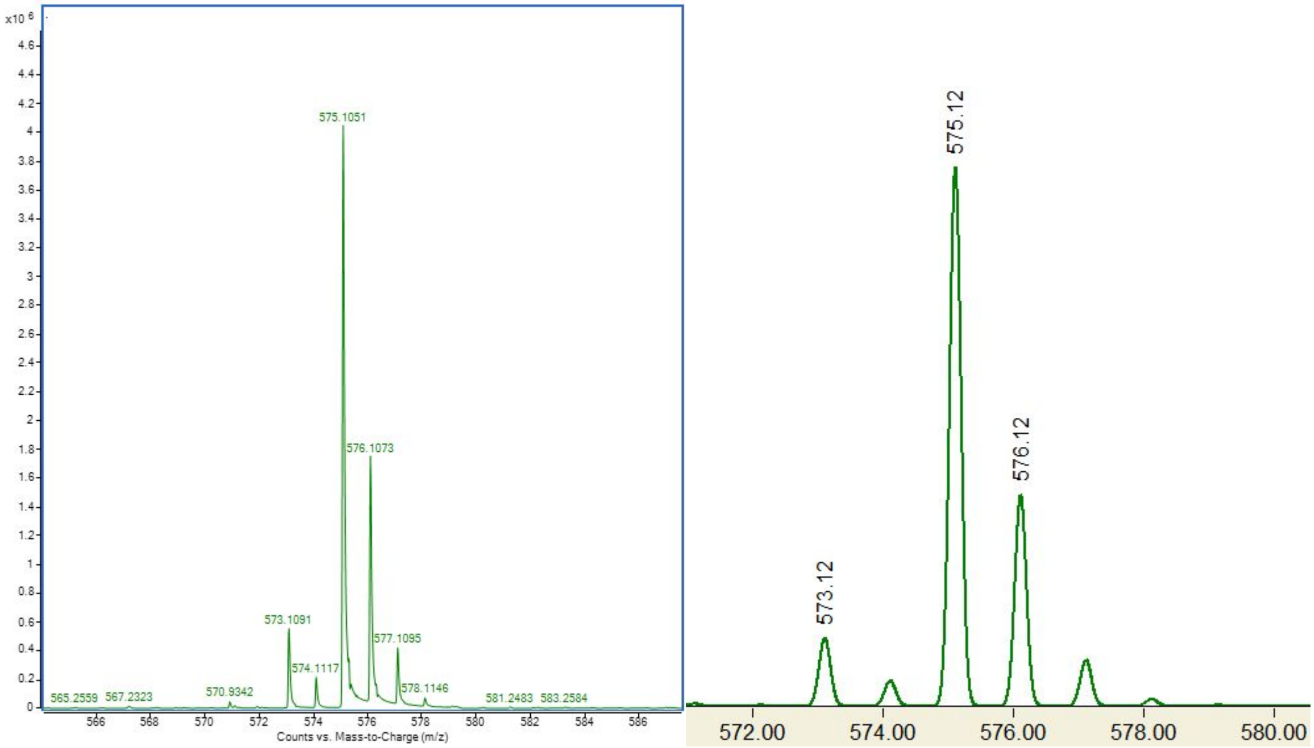

Calcd. base peak for $[Fe_2Cp_2(CO)(CNXyl)_2(CNMeXyl)]^+$ = $C_{39}H_{40}Fe_2N_3O$: 678.196 Da.

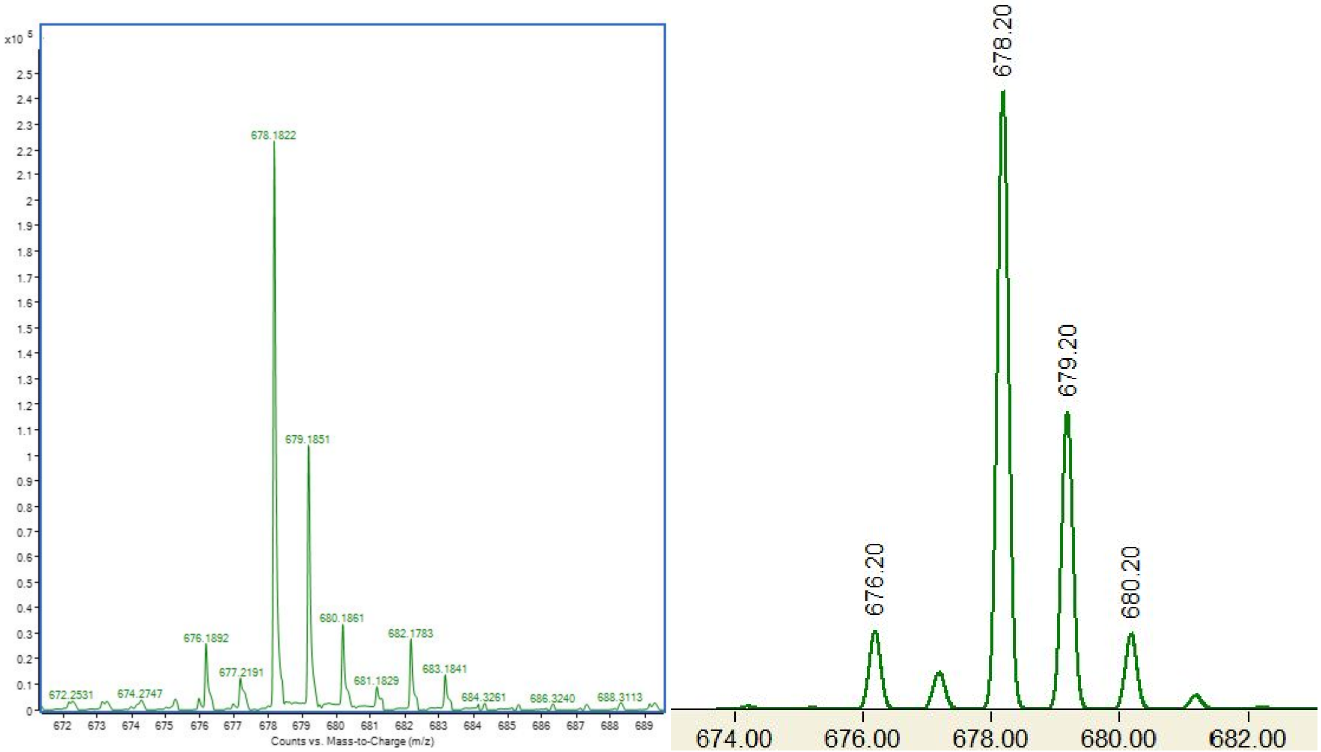

Calcd. base peak for $[Fe_3Cp_3(CNXyl)_3(CNMeXyl)''C'']^+$ = $C_{53}H_{54}Fe_3N_4$: 914.254 Da.

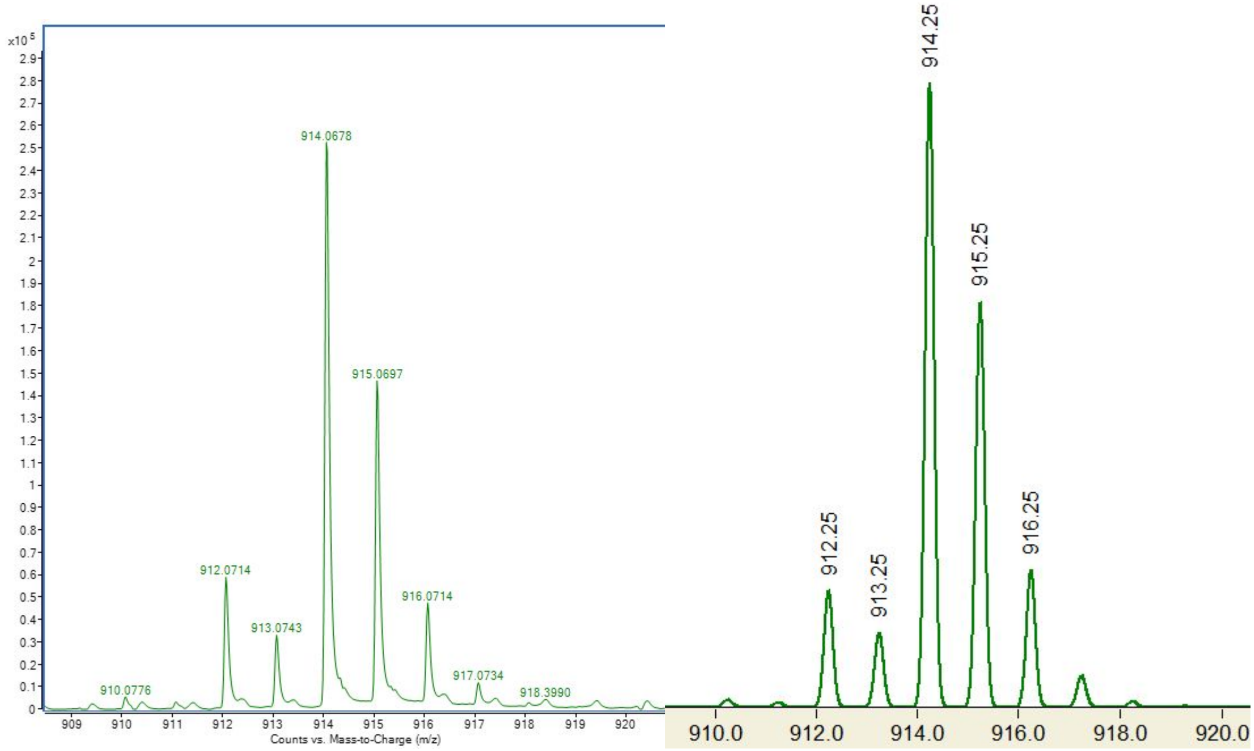

Calcd. base peak for $[Fe_2Cp_2Cl(CO)_2(CNMeXyl)]$, **3e** = $C_{22}H_{22}ClFe_2NO_2$: 479.558 Da (not present, as H^+ adduct).

Figure S70. Comparison between experimental MS spectrum (left) and simulated isotopic patterns (right) of the CH_2Cl_2-soluble fraction of the precipitate formed over several days at 37 °C from a solution of [**1f**]CF_3SO_3 in DMEM-C-dil. Major species in **bold** (m/Z = 474.005, **520.129**, **579.063**)

Calcd. base peak for $[Fe_2Cp_2(CO)_3\{CNMe(4\text{-}C_6H_4OMe)\}]^+$, [**1f**]$^+$ = $C_{22}H_{20}Fe_2NO_4$: 474.018 Da.

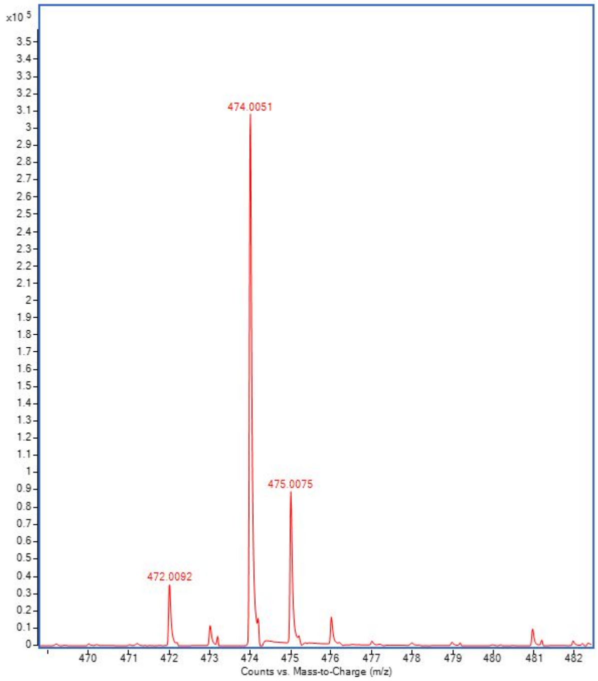

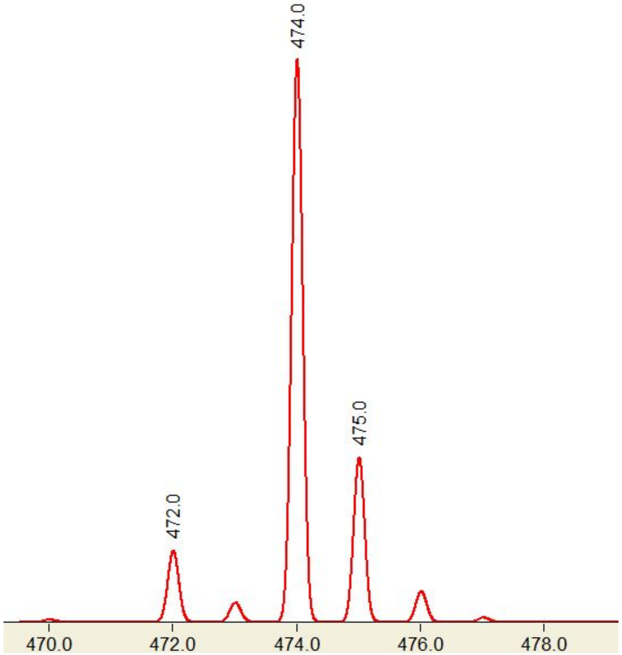

Calcd. base peak for $[FeCp\{CN(4\text{-}C_6H_4OMe)\}_3]^+$ = $C_{29}H_{26}FeN_3O_3$: **520.132** Da.

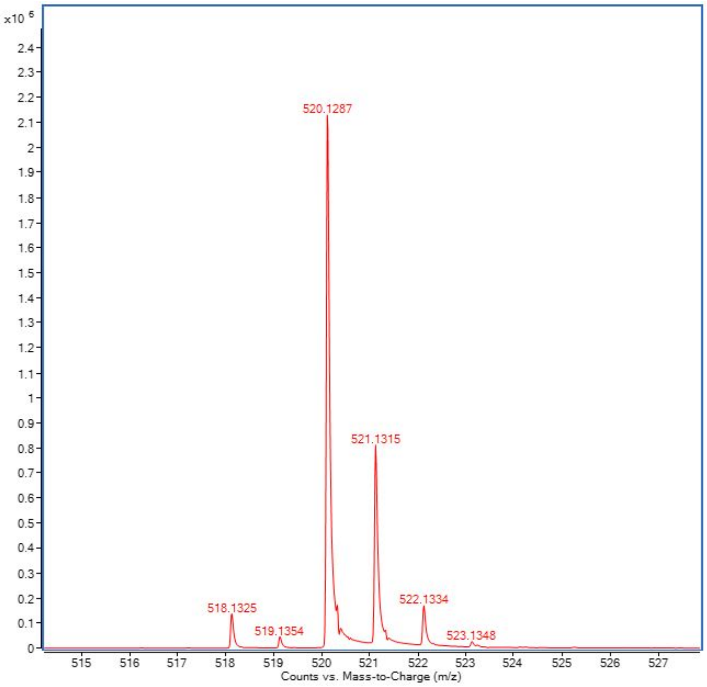

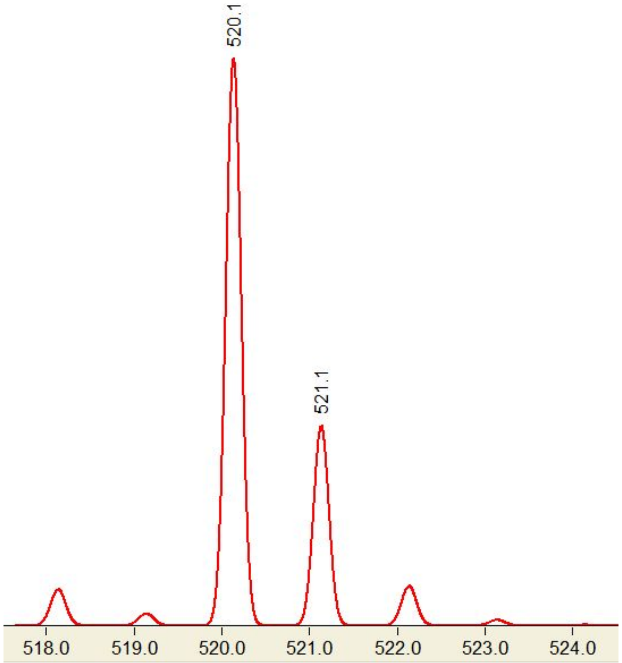

Calcd. base peak for $[Fe_2Cp_2(CO)_2\{CN(4\text{-}C_6H_4OMe)\}\{CNMe(4\text{-}C_6H_4OMe)\}]^+$ = $C_{29}H_{27}Fe_2N_2O_4$: **579.076** Da.

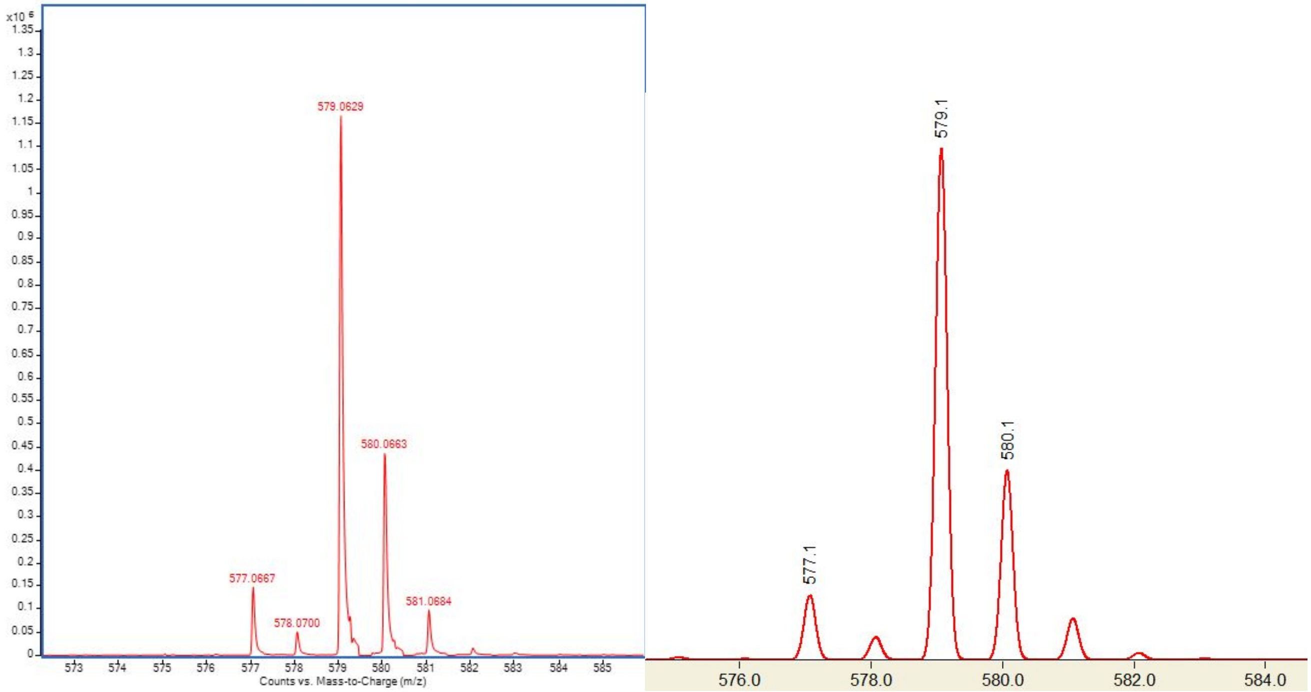

Calcd. base peak for $[Fe_2Cp_2Cl(CO)_2\{CNMe(4\text{-}C_6H_4OMe)\}$, **3f** = $C_{21}H_{20}ClFe_2NO_3$: 481.531 Da (not present, as H^+ adduct)

NMR and UV-Vis analyses of aqueous solutions of diiron compounds in various conditions

Table S14. ^{1}H NMR or UV-Vis analysis of aqueous solutions of [**1a**]$^+$ kept for 72 h with additional reagents / in other conditions than 37 °C. All experiments were carried out in air under ambient light except those highlighted in blue (under Ar or N_2 atmosphere).

Entry	Starting material	Solution, technique	Initial concentration [a] c^0_{Fe2} / mol·L^{-1}	Different conditions / additional reagent(s) (relative molar amount vs starting material)	compounds detected in the final solution [a] (% amount respect to the starting material)		
					[**1a**]$^+$ [b]	$Me_2NH_2^+$ (% *vs* consumed [**1a**]$^+$)	CpH
S14-1	[**1a**]NO_3	H_2O, UV-Vis	$1.17\cdot10^{-3}$	N_2 atmosphere	94.4	x	x
S14-2	[**1a**]NO_3	H_2O, UV-Vis	$5.12\cdot10^{-4}$	N_2 atmosphere	92.9	x	x
S14-3	[**1a**]NO_3	H_2O, UV-Vis	$1.49\cdot10^{-4}$	N_2 atmosphere	86.5	x	x
S14-4	[**1a**]NO_3	D_2O, NMR	$2.03\cdot10^{-2}$	25 °C	98.8	1.0 (*87*)	0.5
S14-5	[**1a**]NO_3	D_2O, NMR	$5.56\cdot10^{-3}$	25 °C	88.8	2.5 (*22*)	trace
S14-6	[**1a**]CF_3SO_3	D_2O, NMR	$5.50\cdot10^{-3}$	25 °C	84.1	5.3 (*34*)	1.5
S14-7	[**1a**]NO_3	D_2O, NMR	$2.03\cdot10^{-2}$	50 °C	97.5	1.9 (*75*)	0.2
S14-8	[**1a**]NO_3	D_2O, NMR	$5.53\cdot10^{-3}$	50 °C	87.5	6.4 (*51*)	0.6
S14-9	[**1a**]CF_3SO_3	D_2O, NMR	$5.50\cdot10^{-3}$	50 °C	83.7	10.2 (*63*)	0.8
S14-10	[**1a**]CF_3SO_3	D_2O, NMR	$3.74\cdot10^{-3}$	50 °C	81.0	11.7 (*62*)	< LOD
S14-11	[**1a**]NO_3	D_2O, NMR	$2.03\cdot10^{-2}$	70 °C	93.8	4.3 (*69*)	0.2
S14-12	[**1a**]NO_3	D_2O, NMR	$5.53\cdot10^{-3}$	70 °C	85.1	11.0 (*74*)	1.1
S14-13	[**1a**]NO_3	D_2O, NMR	$4.16\cdot10^{-3}$	H_2O_2 (2.3 eq)	44.3	17.7 (*32*)	1.3
S14-14	[**1a**]NO_3	H_2O, UV-Vis	$1.05\cdot10^{-3}$	H_2O_2 (2.0 eq)	47.0 ± 1.3	x	x
S14-15	[**1a**]NO_3	H_2O, UV-Vis	$9.14\cdot10^{-4}$	H_2O_2 (1.0 eq)	43.3 ± 5.3	x	x
S14-16	[**1a**]CF_3SO_3	D_2O, NMR	$3.53\cdot10^{-3}$	HCl (7 eq)	87.2 [c]	9.0 (*70*)	< LOD
S14-17	[**1a**]NO_3	D_2O, NMR	$5.53\cdot10^{-3}$	AcOH (10 eq)	85.6	7.1 (*50*)	2.7
S14-18	[**1a**]NO_3	H_2O, UV-Vis	$8.63\cdot10^{-4}$	AcOH (22 eq)	79.8 ± 2.3	x	x
S14-19	[**1a**]NO_3	D_2O, NMR	$5.56\cdot10^{-3}$	$NaHCO_3$ (10 eq)	90.6	3.1 (*33*)	1.2
S14-20	[**1a**]NO_3	H_2O, UV-Vis	$8.98\cdot10^{-4}$	$NaHCO_3$ (14 eq)	69.1	x	x
S14-21	[**1a**]NO_3	D_2O, NMR	$6.76\cdot10^{-3}$	Et_3N/[Et_3NH]Cl (12/12 eq)	68.8	9.0 (*29*)	< LOD
S14-22	[**1a**]NO_3	D_2O, NMR	$5.53\cdot10^{-3}$	Et_3N (10 eq)	≈ 63 [d]	12 (*32*) [d,e]	0.5
S14-23	[**1a**]NO_3	H_2O, UV-Vis	$8.65\cdot10^{-4}$	K_2CO_3 (9.0 eq)	< 32	x	x
S14-24	[**1a**]NO_3	H_2O, UV-Vis	$8.64\cdot10^{-4}$	Et_3N (13 eq)	43.5 ± 3.3	x	x
S14-25	[**1a**]NO_3	D_2O, NMR	$4.16\cdot10^{-3}$	$Me_3NO\cdot2H_2O$ (2.3 eq)	63.1	27.3 (*74*)	2.0
S14-26	[**1a**]CF_3SO_3	D_2O, NMR	$3.80\cdot10^{-3}$	$Me_3NO\cdot2H_2O$ (3.1 eq)	54.3	22.4 (*49*)	< LOD
S14-27	[**1a**]CF_3SO_3	D_2O, NMR	$3.43\cdot10^{-3}$	$Me_3NO\cdot2H_2O$ (1.7 eq)	69.3	15.9 (*52*)	< LOD
S14-28	[**1a**]CF_3SO_3	D_2O, NMR	$1.88\cdot10^{-3}$	$Me_3NO\cdot2H_2O$ (2.0 eq)	51.8	20.9 (43)	< LOD

S14-29	[**1a**]CF_3SO_3	D_2O, NMR	$1.88 \cdot 10^{-3}$	$Me_3NO \cdot 2H_2O$ (4.2 eq)	35.6	22.2 (35)	< LOD
S14-30	[**1a**]CF_3SO_3	D_2O, NMR	$1.88 \cdot 10^{-3}$	$Me_3NO \cdot 2H_2O$ (5.0 eq)	27.7	31.4 (44)	trace
S14-31	[**1a**]NO_3	H_2O, UV-Vis	$9.05 \cdot 10^{-4}$	$Me_3NO \cdot 2H_2O$ (2.0 eq)	47.5 ± 3.8	x	x
S14-32	[**1a**]NO_3	D_2O, NMR	$1.00 \cdot 10^{-2}$	PTA (3.5 eq) [f]	84.6 [f]	0.1	< LOD

[a] ^1H NMR experiments: the initial concentration and % relative amount of compounds with respect to the freshly prepared solution were calculated using Me_2SO_2 or DSS as internal standard. UV-Vis experiments: the initial concentration was calculated from mass and volume data (volumetric solutions) or from the molar absorbance at 340 nm of the freshly-prepared solution; the % residual amount of starting material was calculated by the relative decrease of the UV-Vis peak at 340 nm (see main text for details). Data expressed with 2 or 1 decimal digits to avoid excessive rounding. [b] Including *cis* and *trans* isomers. [c] Marked change in the *cis*/*trans* isomer ratio: 46 (0 h), 1.7 (72 h). [d] Precise quantitation not possible due to overlap between Me_2SO_2 and Et_3N signals. [e] As Me_2NH or $Me_2NH/Me_2NH_2^+$ in comparable amount. [f] PTA = 1,3,5-triaza-7-phosphaadamantane. Formation of [**2a**]$^+$ (see Figure S75).

Figure S71. % Residual amount of [**1a**]$^+$ after 72 h *vs.* decreasing initial molar concentration of the aqueous solution (logarithmic scale, − Log c^0_{Fe2} = pFe^0) in different conditions, compared to standard system (H_2O, air/ambient light, 37 °C; blue points and linear fitting). Data refer to Tables S14 and S18.

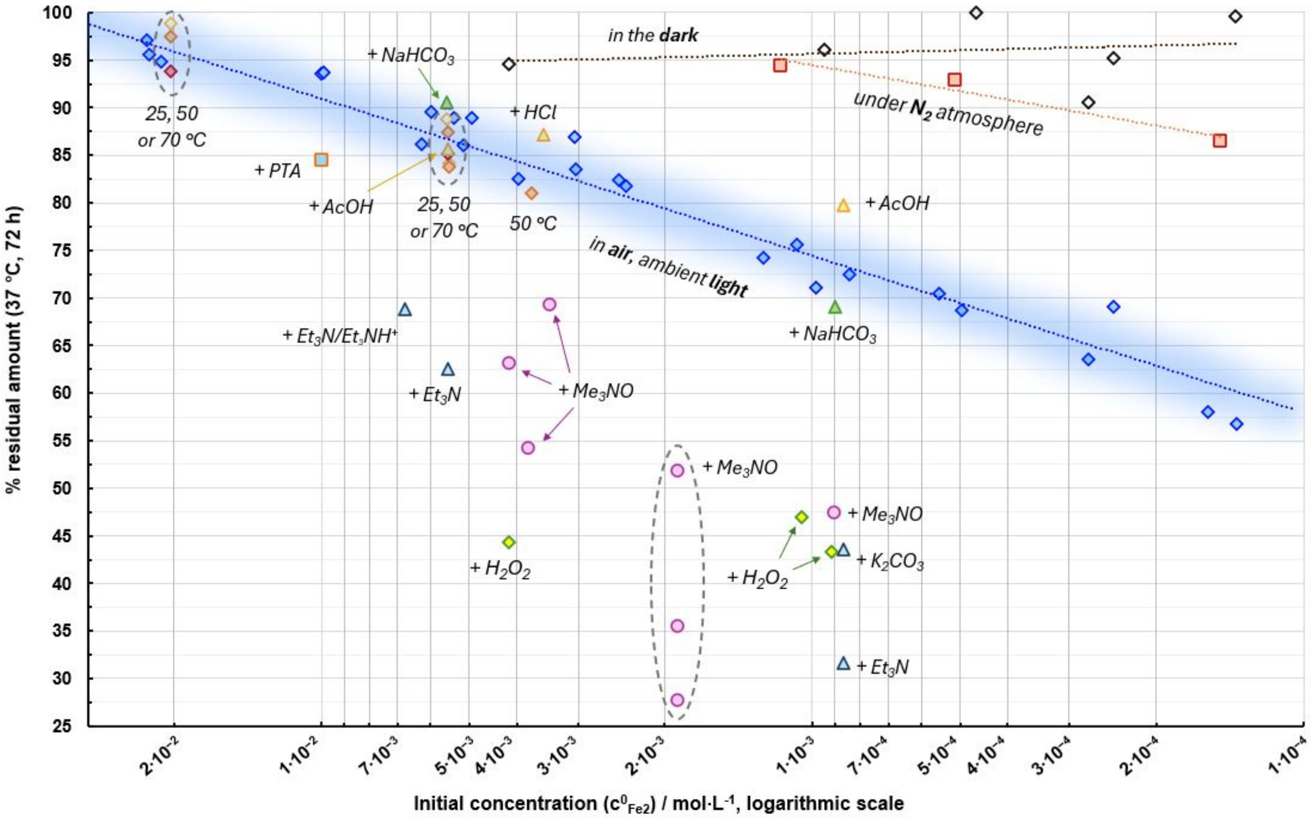

Table S15. ^{1}H NMR or UV-Vis analysis of solutions of [**1c**]$^+$ in water or cell culture medium kept for 72 h with additional reagents / in other conditions than 37 °C. All experiments were carried out in air under ambient light except those highlighted in blue (under Ar atmosphere).

Entry	Starting material	Solution, technique	Initial concentration [a] c^0_{Fe2} / mol·L^{-1}	Different conditions / additional reagent(s) (relative molar amount vs starting material)	compounds detected in the final solution [a] (% amount respect to the starting material)		
					[**1c**]$^+$ [b]	$BnMeNH_2^+$ (% *vs* consumed [**1c**]$^+$)	CpH
S15-1	[**1c**]NO_3	D_2O, NMR	$1.06 \cdot 10^{-2}$	Ar atmosphere	99.4	< LOD	< LOD
S15-2	[**1c**]NO_3	D_2O, NMR	$1.66 \cdot 10^{-3}$	H_2O_2 (2.0 eq)	57.4	7.3 (*16*)	trace
S15-3	[**1c**]NO_3	H_2O, UV-Vis	$1.02 \cdot 10^{-3}$	H_2O_2 (2.0 eq)	55.1 ± 2.0	/	/
S15-4	[**1c**]CF_3SO_3	D_2O, NMR	$1.14 \cdot 10^{-3}$	HCl (21 eq)	66.6 [c]	17.5 (52)	4.7
S15-5	[**1c**]NO_3	D_2O, NMR	$6.41 \cdot 10^{-3}$	AcOH/AcONa (15/15 eq)	92.2	3.0 (*38*)	1.7
S15-6	[**1c**]NO_3	H_2O, UV-Vis	$9.83 \cdot 10^{-4}$	AcOH (16 eq)	71.7 ± 4.4	/	/
S15-7	[**1c**]NO_3	D_2O, NMR	$6.41 \cdot 10^{-3}$	$NaHCO_3$ (20 eq)	90.2	4.6 (*48*)	2.1
S15-8	[**1c**]NO_3	H_2O, UV-Vis	$9.92 \cdot 10^{-4}$	$NaHCO_3$ (14 eq)	75.9 ± 5.2	/	/
S15-9	[**1c**]NO_3	D_2O, NMR	$6.49 \cdot 10^{-3}$	Et_3N/[Et_3NH]Cl (15/15 eq)	50.9	12.4 (*25*)	< LOD
S15-10	[**1c**]NO_3	H_2O, UV-Vis	$1.04 \cdot 10^{-3}$	K_2CO_3 (14 eq)	33.9 ± 2.3	/	/
S15-11	[**1c**]NO_3	D_2O, NMR	$4.12 \cdot 10^{-3}$	$Me_3NO \cdot 2H_2O$ (2.1 eq)	65.8	14.3 (*40*)	3.2
S15-12	[**1c**]NO_3	D_2O, NMR	$2.35 \cdot 10^{-3}$	$Me_3NO \cdot 2H_2O$ (4.0 eq)	52.0	17.9 (37)	4.2
S15-13	[**1c**]CF_3SO_3	D_2O, NMR	$1.30 \cdot 10^{-3}$	$Me_3NO \cdot 2H_2O$ (2.6 eq)	64.3	13.2 (*37*)	3.9
S15-14	[**1c**]CF_3SO_3	D_2O, NMR	$1.19 \cdot 10^{-3}$	$Me_3NO \cdot 2H_2O$ (5.7 eq)	50.6	23.1 (*47*)	2.8
S15-15	[**1c**]NO_3	D_2O, NMR	$6.40 \cdot 10^{-3}$	PTA (3.7 eq) [d]	87.6 [d]	< LOD	< LOD
S15-16	[**1c**]NO_3	DMEM-d, NMR	$6.40 \cdot 10^{-3}$	PTA (2.5 eq) [d]	89.5 [d]	< LOD	< LOD

[a] ^{1}H NMR experiments: the initial concentration and % relative amount of compounds with respect to the freshly prepared solution were calculated using Me_2SO_2 or DSS as internal standard. UV-Vis experiments: the initial concentration was calculated from mass and volume data (volumetric solutions) or from the molar absorbance at 340 nm of the freshly-prepared solution; the % residual amount of starting material was calculated by the relative decrease of the UV-Vis peak at 340 nm (see main text for details). Data expressed with 2 or 1 decimal digits to avoid excessive rounding. [b] For [**1c**]CF_3SO_3: including *cis* and *trans* isomers. [c] Marked change in the *cis*/*trans* isomer ratio: 7.0 (0 h), 1.6 (72 h). [d] PTA = 1,3,5-triaza-7-phosphaadamantane. Formation of [**2c**]$^+$ (Figure S75).

Figure S72. % Residual amount of [**1c**]$^+$ after 72 h at 37 °C (*y* axis, linear scale) *vs.* decreasing initial molar concentration of the aqueous solution (*x* axis, logarithmic scale, − Log c^0_{Fe2} = pFe^0) in different conditions, compared to standard system (H_2O, air/ambient light, 37 °C; blue points and linear fitting). Data refer to Tables S15 and S18.

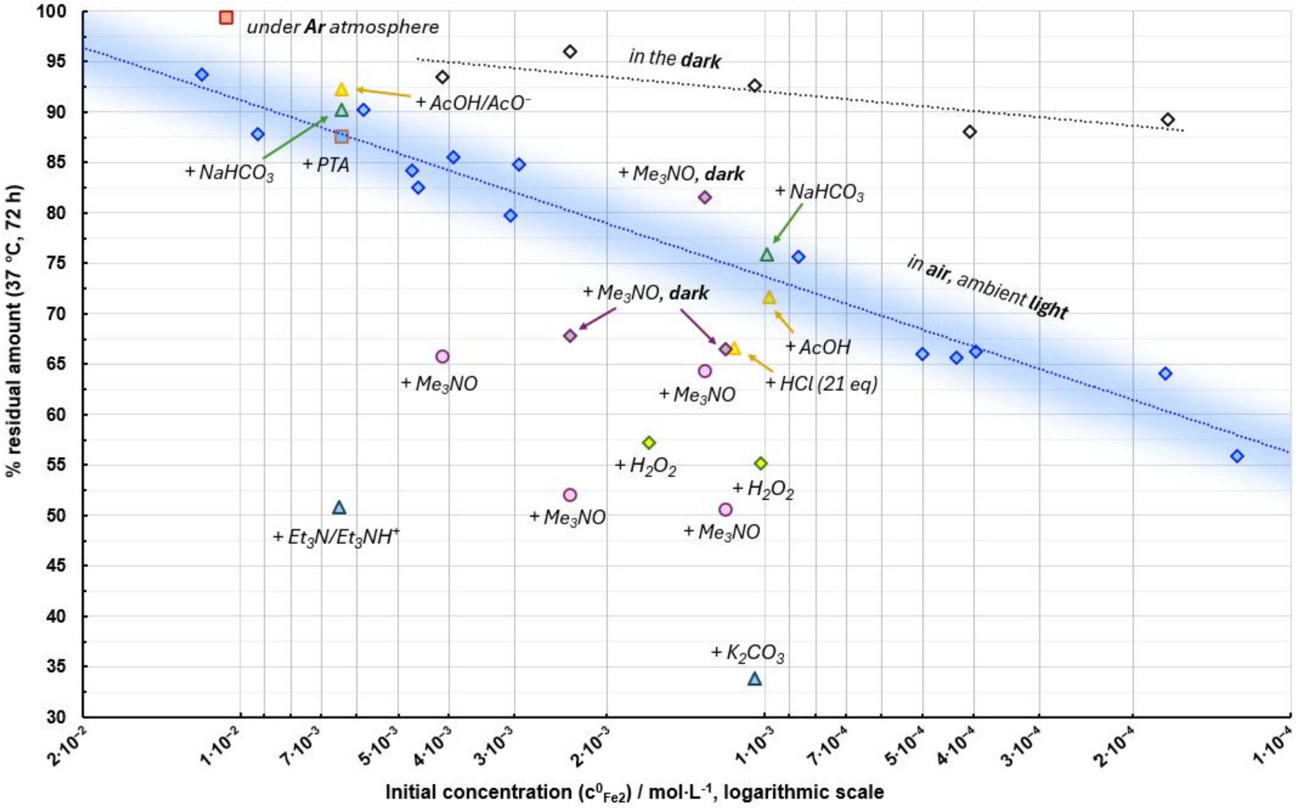

Table S16. ^{1}H NMR or UV-Vis analysis of solutions of [**1e**]$^+$ in water or cell culture medium kept for 72 h with additional reagents / in other conditions than 37 °C. All experiments were carried out in air under ambient light except those highlighted in blue (under Ar atmosphere) or in gray (in the dark). Data are plotted in Figure 9 (main text).

Entry	Starting material	Solution, technique	Initial concentration [a] c^0_{Fe2} / mol·L^{-1}	Different conditions / additional reagent(s) (relative molar amount vs starting material)	compounds detected in the final solution [a] (% amount respect to the starting material)		
					[**1e**]$^+$	XylMeNH$_2^+$ (% *vs* consumed [**1e**]$^+$)	CpH
S16-1	[**1e**]NO$_3$	D$_2$O, NMR	5.20·10^{-3}	Ar atmosphere	97.0	< LOD	< LOD
S16-2	[**1e**]NO$_3$	DMEM-d, NMR	5.20·10^{-3}	Ar atmosphere	96.8	< LOD	< LOD
S16-3	[**1e**]NO$_3$	H$_2$O, NMR [b]	3.74·10^{-3}	Ar atmosphere	99.5	< LOD	< LOD
S16-4	[**1e**]NO$_3$	D$_2$O, NMR	2.75·10^{-3}	Ar atmosphere	94.5	< LOD	< LOD
S16-5	[**1e**]NO$_3$	DMEM, UV-Vis	1.05·10^{-3}	Ar atmosphere	97.6	/	/
S16-6	[**1e**]NO$_3$	H$_2$O, UV-Vis	4.42·10^{-4}	Ar atmosphere	96.4	/	/
S16-7	[**1e**]NO$_3$	H$_2$O, UV-Vis	4.97·10^{-4}	Ar atmos. + Me$_3$NO (2 eq)	75.7	/	/
S16-8	[**1e**]NO$_3$	H$_2$O, UV-Vis	4.69·10^{-4}	Ar atmos. + H$_2$O$_2$ (1 eq)	76.6	/	/
S16-9	[**1e**]NO$_3$	H$_2$O, UV-Vis	4.39·10^{-4}	Ar atmos. + Me$_3$NO (2 eq) + H$_2$O$_2$ (1 eq)	67.8	/	/
S16-10	[**1e**]NO$_3$	H$_2$O, UV-Vis	8.79·10^{-4}	Ar atmos. + H$_2$O$_2$ (5 eq)	33.0	/	/
S16-11	[**1e**]NO$_3$	H$_2$O, UV-Vis	9.70·10^{-4}	AgNO$_3$ (2 eq)	50.7	/	/
S16-12	[**1e**]NO$_3$	H$_2$O, UV-Vis	5.45·10^{-4}	AgNO$_3$ (2 eq)	56.3 ± 4.8	/	/
S16-13	[**1e**]NO$_3$	D$_2$O, NMR	4.16·10^{-3}	H$_2$O$_2$ (2.5 eq)	38.1	7.1	1.6
S16-14	[**1e**]NO$_3$	H$_2$O, UV-Vis	7.72·10^{-4}	H$_2$O$_2$ (2 eq)	39.3	/	/
S16-15	[**1e**]NO$_3$	H$_2$O, UV-Vis	9.26·10^{-4}	Ce(SO$_4$)$_2$ (2 eq)	0	/	/
S16-16	[**1e**]NO$_3$	H$_2$O, UV-Vis	7.97·10^{-4}	Ce(SO$_4$)$_2$ (4 eq)	0	/	/
S16-17	[**1e**]CF$_3$SO$_3$	D$_2$O, NMR	2.75·10^{-3}	HCl (9 eq)	67.3	8 (24)	1.4
S16-18	[**1e**]CF$_3$SO$_3$	D$_2$O, NMR	2.56·10^{-3}	HCl (9 eq)	64.3	10 (27)	1.7
S16-19	[**1e**]NO$_3$	H$_2$O, UV-Vis	5.25·10^{-4}	AcOH (16 eq)	56.6 ± 3.1	/	/
S16-20	[**1e**]NO$_3$	D$_2$O, NMR	2.75·10^{-3}	AcOH/AcONa (15/15 eq)	67.5	11	< LOD
S16-21	[**1e**]NO$_3$	H$_2$O, UV-Vis	8.64·10^{-4}	KHSO$_4$ (13 eq)	57.9 ± 2.0	/	/
S16-22	[**1e**]NO$_3$	H$_2$O, UV-Vis	4.52·10^{-4}	KHSO$_4$ (12 eq)	51.8 ± 4.0	/	/
S16-23	[**1e**]NO$_3$	H$_2$O, UV-Vis	3.09·10^{-4}	AcOH/AcONa (15/15 eq)	37.1	/	/
S16-24	[**1e**]NO$_3$	D$_2$O, NMR	2.75·10^{-3}	NaHCO$_3$ (20 eq)	73.3	9.1	< LOD
S16-25	[**1e**]NO$_3$	H$_2$O, UV-Vis	9.83·10^{-4}	NaHCO$_3$ (20 eq)	55.9	/	/
S16-26	[**1e**]NO$_3$	H$_2$O, UV-Vis	5.20·10^{-4}	NaHCO$_3$ (14 eq)	50.0 ± 3.5	/	/
S16-27	[**1e**]NO$_3$	D$_2$O, NMR	2.75·10^{-3}	Et$_3$N/Et$_3$NHCl (15/15 eq)	56.0	5.8	trace
S16-28	[**1e**]NO$_3$	H$_2$O, UV-Vis	9.19·10^{-4}	K$_2$CO$_3$ (10 eq)	40.6 ± 4.1	/	/
S16-29	[**1e**]NO$_3$	H$_2$O, UV-Vis	5.27·10^{-4}	Et$_3$N/Et$_3$NHCl (15/15 eq)	*0 ?*	/	/
S16-30	[**1e**]NO$_3$	H$_2$O, UV-Vis	3.09·10^{-4}	Et$_3$N/Et$_3$NHCl (15/15 eq)	*0 ?*	/	/

S16-31	[**1e**]NO_3	D_2O, NMR	$5.18\cdot10^{-3}$	PTA (4.1 eq) [c]	77.9 [c]	< LOD	< LOD
S16-32	[**1e**]NO_3	DMEM-d, NMR	$4.82\cdot10^{-3}$	PTA (4.2 eq) [c]	79.0 [c]	< LOD	< LOD
S16-33	[**1e**]NO_3	D_2O, NMR	$4.16\cdot10^{-3}$	$Me_3NO\cdot2H_2O$ (2.2 eq)	63.7	6.4	4.0
S16-34	[**1e**]NO_3	D_2O, NMR	$3.57\cdot10^{-3}$	$Me_3NO\cdot2H_2O$ (3.0 eq)	60.2	8.8	4.1
S16-35	[**1e**]NO_3	D_2O, NMR	$3.57\cdot10^{-3}$	$Me_3NO\cdot2H_2O$ (5.1 eq)	49.8	11	5.0
S16-36	[**1e**]NO_3	D_2O, NMR	$2.75\cdot10^{-3}$	$Me_3NO\cdot2H_2O$ (12 eq)	3.5	?	19.0
S16-37	[**1e**]NO_3	H_2O, UV-Vis	$9.40\cdot10^{-4}$	$Me_3NO\cdot2H_2O$ (1 eq)	41.8	/	/
S16-38	[**1e**]NO_3	H_2O, UV-Vis	$8.71\cdot10^{-4}$	$Me_3NO\cdot2H_2O$ (2 eq)	42.1	/	/
S16-39	[**1e**]NO_3	H_2O, UV-Vis	$5.03\cdot10^{-4}$	$Me_3NO\cdot2H_2O$ (2 eq)	40.3	/	/
S16-40	[**1e**]NO_3	D_2O, NMR	$3.57\cdot10^{-3}$	H_2O_2 (12 eq), dark	0	0	0

[a] ^1H NMR experiments: the initial concentration and % relative amount of compounds with respect to the freshly prepared solution were calculated using Me_2SO_2 or DSS as internal standard. UV-Vis experiments: the initial concentration was calculated from mass and volume data (volumetric solutions) or from the molar absorbance at 340 nm of the freshly-prepared solution; the % residual amount of starting material was calculated by the relative decrease of the UV-Vis peak at 340 nm (see main text for details). Data expressed with 2 or 1 decimal digits to avoid excessive rounding. [b] A known amount of Me_2SO_2 was added to the aqueous solution. The final solution was taken to dryness under vacuum and the solid was analyzed by ^1H NMR ($CDCl_3$). [c] PTA = 1,3,5-triaza-7-phosphaadamantane. Formation of [**2e**]$^+$ (Figure S75).

Figure S73. Residual amount of [**1e**]$^+$ starting from 7.7-9.8·10^{-4} mol/L aqueous solutions at 37 °C in the presence of $NaHCO_3$, $AgNO_3$, Me_3NO, H_2O_2 or $Ce(SO_4)_2$. Values at 72 h correspond to entries 11, 14, 15, 25, 35 in Table S16.

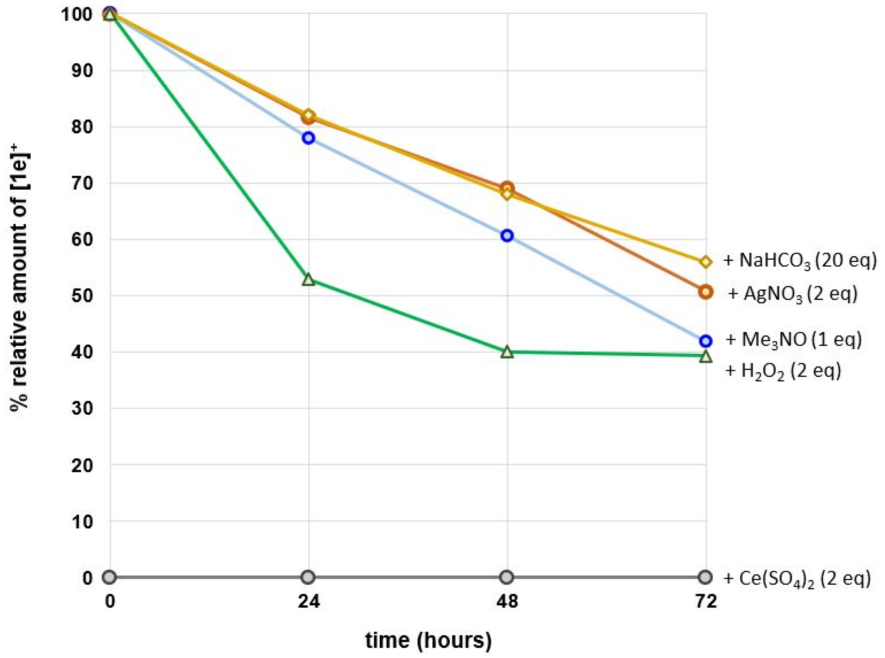

Figure S74. % residual amount of [**1e**]$^+$ in aqueous solutions at 37 °C under Ar atmosphere. Values at 72 h correspond to entries 6-10 in Table S16. Initial [**1e**]$^+$ concentration 4.4-5.0·10^{-4} mol/L except for the green points (8.8·10^{-4} mol/L).

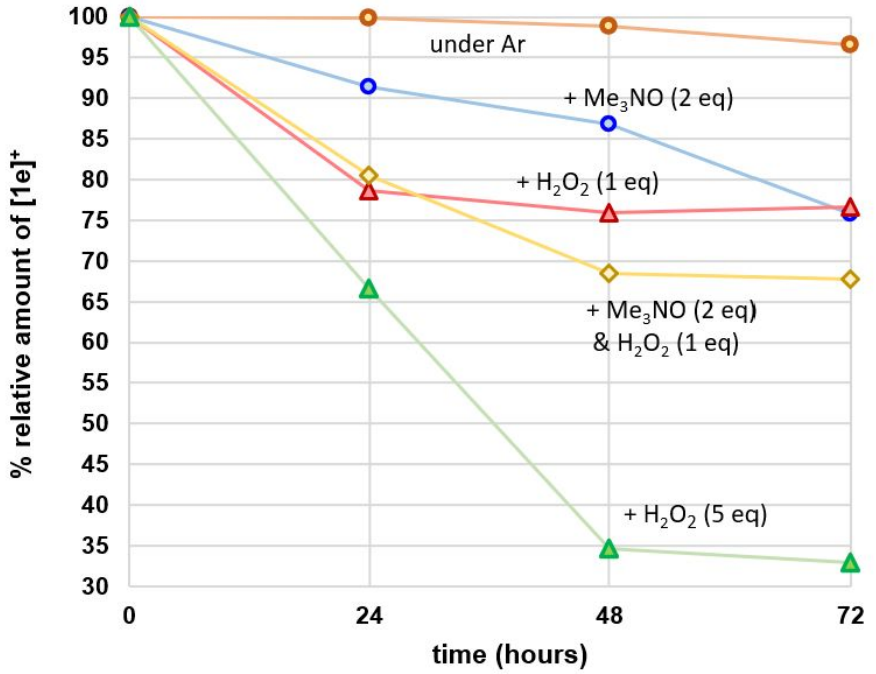

Table S17. ^{1}H NMR analysis of aqueous (D_2O) solutions of [**1**]$^+$ and $Me_3NO{\cdot}2H_2O$ at 37 °C over 72 h. % Amount of compounds are calculated with respect to Me_2SO_2 as internal standard and are referred to the freshly prepared solution. All experiments were carried out in air under ambient light and refer to Tables S14-S16 (time course analysis and Me_3N amount were not previously reported), except those highlighted in gray (in the dark).

Exp	Conditions				% amount of compounds in solution (*ratio with decomposed [1]$^+$*)			
	**Compound**	**Initial concentration (c^0_{Fe2})**	**Initial Me_3NO/[1]$^+$ molar ratio**	**time (h)**	**[1]$^+$ [b]**	**$RMeNH_2^+$**	**CpH**	**Me_3NH^+**
S14-25	[**1a**]NO_3	$4.16{\cdot}10^{-3}$	2.3	72	63.1	27.3 (*0.74*)	2.0	71.0 (***1.93***)
S14-26	[**1a**]CF_3SO_3	$3.80{\cdot}10^{-3}$	3.1	24	84.2	7.8 (*0.50*)	< LOD	11.4 (***0.73***)
				48	68.7	15.4 (*0.49*)	< LOD	21.3 (***0.68***)
				72	54.3	22.4 (*0.49*)	< LOD	31.2 (***0.68***)
S14-27	[**1a**]CF_3SO_3	$3.43{\cdot}10^{-3}$	1.7	24	89.3	5.4 (*0.51*)	< LOD	5.4 (***0.51***)
				48	79.0	9.6 (*0.46*)	< LOD	10.9 (***0.52***)
				72	69.3	15.9 (*0.52*)	< LOD	15.3 (***0.50***)
S14-28	[**1a**]CF_3SO_3	$1.88{\cdot}10^{-3}$	2.0	72	51.8	23.4 (*0.49*)	< LOD	50.1 (***1.04***)
S14-29	[**1a**]CF_3SO_3	$1.88{\cdot}10^{-3}$	4.2	72	35.6	22.2 (*0.35*)	< LOD	51.5 (***0.80***)
S14-30	[**1a**]CF_3SO_3	$1.88{\cdot}10^{-3}$	5.0	72	27.7	31.4 (0.*44*)	trace	53.0 (***0.85***)
S15-11	[**1c**]NO_3	$4.12{\cdot}10^{-3}$	2.1	72	65.8	14.3 (0.*40*)	3.2	63.6 (***1.86***)
S15-12	[**1c**]NO_3	$2.35{\cdot}10^{-3}$	4.0	72	52.0	17.9 (0.37)	4.2	74.6 (***1.55***)
S15-13	[**1c**]CF_3SO_3	$1.30{\cdot}10^{-3}$	2.6	72	64.3	13.2 (0.*37*)	3.9	28.0 (***0.78***)
S15-14	[**1c**]CF_3SO_3	$1.19{\cdot}10^{-3}$	5.7	72	50.6	23.1 (0.47)	2.8	43.3 (***0.88***)
S16-33	[**1e**]NO_3	$4.16{\cdot}10^{-3}$	2.2	72	63.7	6.4 (*0.18*)	4.0	38.3 (***1.05***)
S16-34	[**1e**]NO_3	$3.57{\cdot}10^{-3}$	3.0	72	60.2	8.8 (*0.22*)	4.1	45.5 (***1.14***)
S16-35	[**1e**]NO_3	$3.57{\cdot}10^{-3}$	5.1	72	49.8	11 (*0.21*)	5.0	79.9 (***1.56***)
S16-36	[**1e**]NO_3	$2.75{\cdot}10^{-3}$	12	24	34.4	?	8.8	60.2 (***0.92***)
				48	14.0	?	10.7	77.9 (***0.91***)
				72	3.5	?	19.1	85.0 (***0.88***)
S17-1	[**1c**]NO_3	$2.35{\cdot}10^{-3}$	4.0	72	67.8	11.0 (*0.34*)	3.0	60.2 (***1.87***)
S17-1	[**1c**]CF_3SO_3	$1.30{\cdot}10^{-3}$	2.6	72	81.5	6.8 (*0.37*)	< LOD	16.7 (***0.90***)
S17-1	[**1c**]CF_3SO_3	$1.19{\cdot}10^{-3}$	5.7	72	66.4	16.9 (*0.50*)	< LOD	39.4 (***1.17***)

Note: The ^{1}H NMR spectra of a solution of Me_3NO, KNO_3, Me_2SO_2 and TMS in D_2O underwent no changes (specifically, no Me_3NO consumption and no Me_3N formation) after 72 h at 37 °C. [b] For [**1a,c**]CF_3SO_3: including *cis* and *trans* isomers.

Me_3NO. ^{1}H NMR (D_2O): δ/ppm = 3.26. $^{13}C\{^1H\}$ NMR (D_2O): δ/ppm = 45.0.

Me_3N. ^{1}H NMR (D_2O): δ/ppm = 2.89. $^{13}C\{^1H\}$ NMR (D_2O): δ/ppm = 59.9.

Figure S75. % amount of [**1a**]$^+$, Me_3NH^+ and $Me_2NH_2^+$ in solution (left vertical axis) and ratio between Me_3NH^+ and the conversion of [**1a**]$^+$ (red crosses, right vertical axis) over time. Data are included in Table S17 (exp. S14-26 and S14-27).

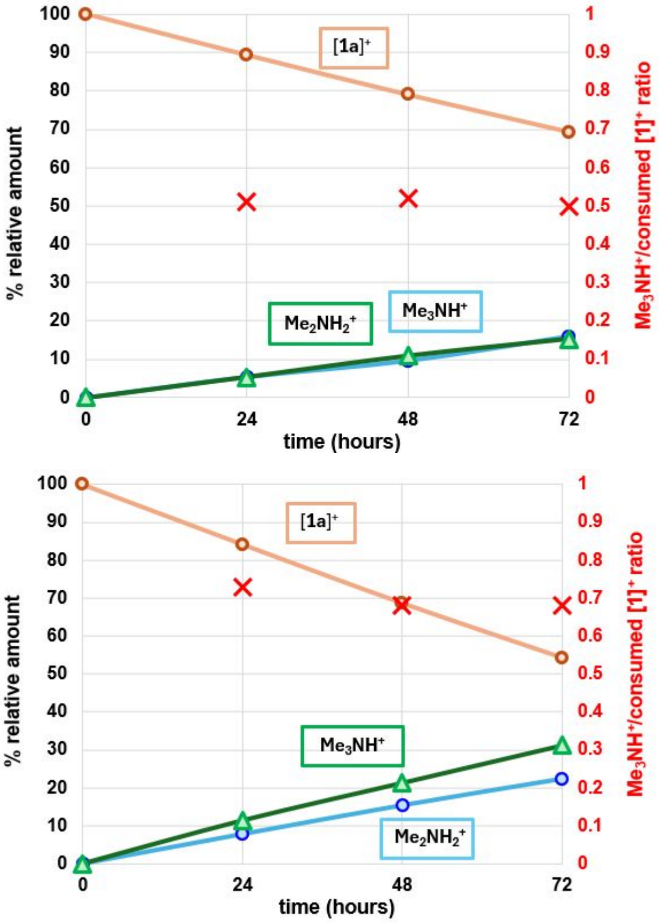

Activation of tricarbonyl complexes in presence of PTA and characterization of a dicarbonyl PTA complex

Table S18. % amount of diiron complexes in solutions of [**1a,c,e**]NO_3 and PTA in D_2O or deuterated cell culture medium kept at 37 °C. Values at 72 h are also included in Tables S14-S16.

Exp	Starting material	c^0_{Fe2} [a] / $mol \cdot L^{-1}$	PTA (eq.)	Solution	% relative amount in solution (^1H NMR) [a]					
					72 h (3 days)			144 h (6 days)		
					[**1**]$^+$	[**2**]$^+$	*total*	[**1**]$^+$	[**2**]$^+$	*total*
S14-32	[**1a**]NO_3	$1.00 \cdot 10^{-2}$	3.5	D_2O	84.6	13.9	*98.4*	56.7	33.4	*90.2*
S15-15	[**1c**]NO_3	$6.40 \cdot 10^{-3}$	3.7	D_2O	87.6	10.6	*98.2*	72.5	24.9	*97.4*
S15-16			2.5	DMEM-d	89.5	9.2	*98.7*	73.0	21.8	*94.8*
S16-31	[**1e**]NO_3	$5.18 \cdot 10^{-3}$	4.1	D_2O	77.9	17.1	*95.0*	46.4	43.8	*90.3*
S16-32		$4.82 \cdot 10^{-3}$	4.2	DMEM-d	79.0	19.0	*98.0*	46.7	43.8	*90.5*

[a] The initial concentration and % relative amount of compounds with respect to the freshly prepared solution were calculated by ^1H NMR using Me_2SO_2 as internal standard. Data expressed with 2 or 1 decimal digits to avoid excessive rounding.

[2a]$^+$. ^1H NMR (D_2O): δ/ppm = 5.20, *5.12* (s, 5H, Cp); 5.02 (d, $^3J_{HP}$ = 1.1 Hz, 5H, Cp^P); 4.36 (m), 4.27 (d, J = 12.2 Hz) (6H, NCH_2); 4.15 (s, 3H, NCH_3); 3.75 (s, 6H, PCH_2); *cis*/*trans* isomer ratio ≈ 10 (72-144 h). ^{31}P NMR (D_2O): δ/ppm = − 14.9, − *15.4*. Signals due to the *trans* isomer are italicized.

[2c]$^+$. ^1H NMR (D_2O): δ/ppm = 5.66, 5.71 (d, $^2J_{HH}$ = 15 Hz, 1H, $PhCH_2$); 5.31, 5.19 (s, 5H, Cp); 5.09, 4.99 (d, $^3J_{HP}$ = 1.0 Hz, 5H, Cp^P); *cis*-*Z*/*E* isomer ratio: 1.0 (72-144 h). ^{31}P NMR (D_2O): δ/ppm = − 13.6, − *16.4*. Signals due to the *cis*-*E* isomer are italicized.

[2e]$^+$. ^1H NMR (D_2O): δ/ppm = 5.37, *4.65* (s, 5H, Cp); *5.15* (d, $^3J_{HP}$ = 1.5 Hz, Cp^P); *4.40*, 4.38 (s, 3H, NCH_3); 4.41-4.35 (m, NCH_2); 2.69, 2.31 (s, 6H, CCH_3); *cis*-*Z*/*E* isomer ratio: 1.4 (72 h), *cis*-*E*/*Z* isomer ratio: 4.4 (144 h). ^{31}P NMR (D_2O): δ/ppm = − 12.3, − *16.2*. Signals due to the *cis*-*E* isomer are italicized.

PTA. ^1H NMR (D_2O): δ/ppm = 4.53 (app. q, J = 13.0 Hz, 6H, NCH_2), 3.97 (d, J = 8.9 Hz, 6H, PCH_2). $^{31}P\{^1H\}$ NMR (D_2O): δ/ppm = – 98.6.

PTA oxide (O=PTA). ^1H NMR (D_2O): δ/ppm = 4.39, 4.26 (d, J = 13 Hz, 6H, NCH_2); 4.03 (d, J = 10 Hz, 6H, PCH_2). $^{31}P\{^1H\}$ NMR (D_2O): δ/ppm = - 2.26.

Figure S76. % amount of diiron complexes in D_2O solution at 37 °C over 144 h in the presence of PTA: [**1a,c,e**]$^+$ (orange points), [**2a,c,e**]$^+$ (blue points), total diiron compounds ([**1**]$^+$ + [**2**]$^+$; green points). Lines are added as visual guidance.

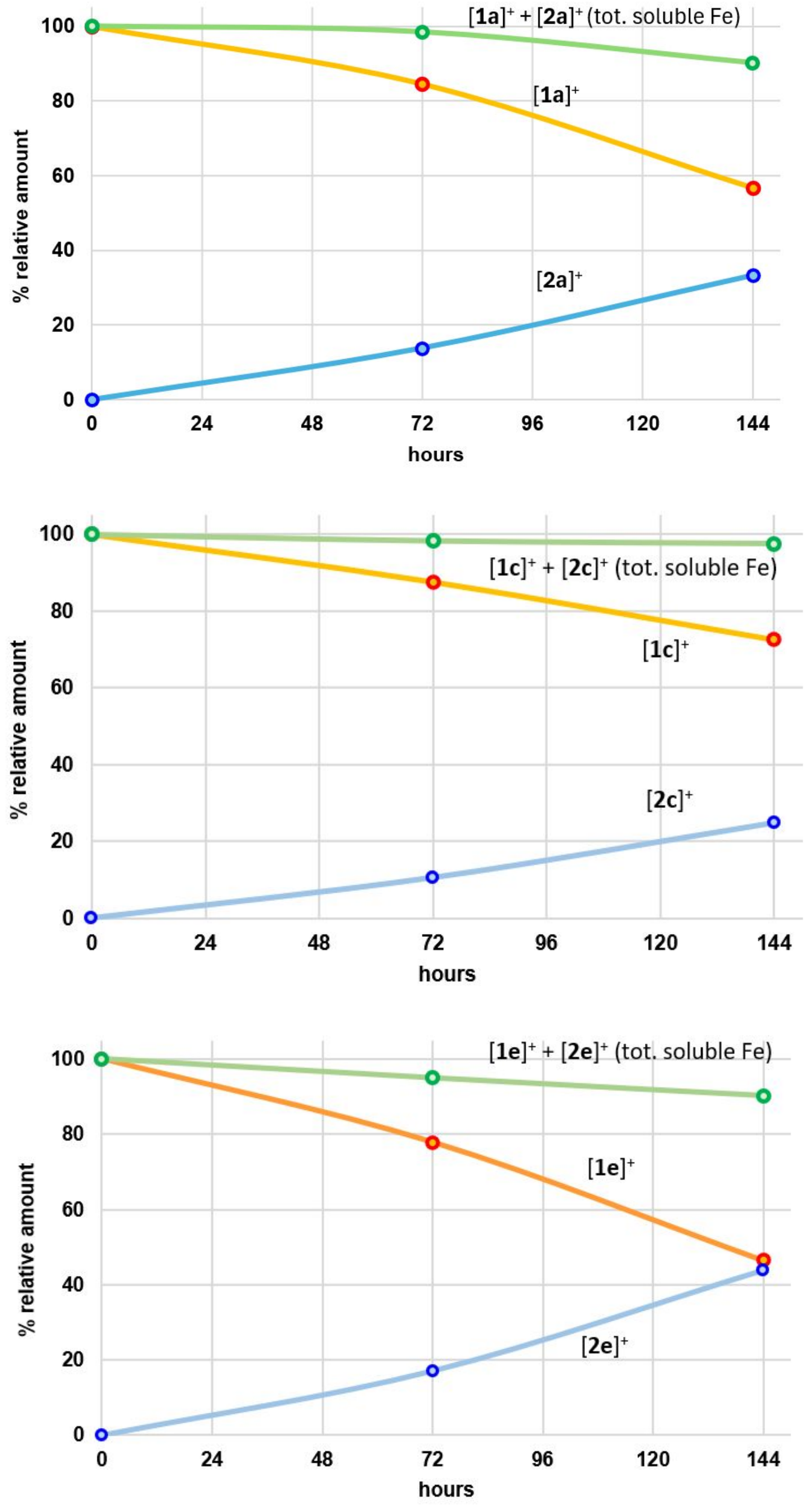

Figure S77. ^{1}H NMR spectra (400 MHz, D_2O) of the freshly prepared solution of [**1c**]NO_3 and PTA (top, blue line), after 144 at 37 °C (middle, green line) and a solution of [**2c**]CF_3SO_3 (bottom, red line).

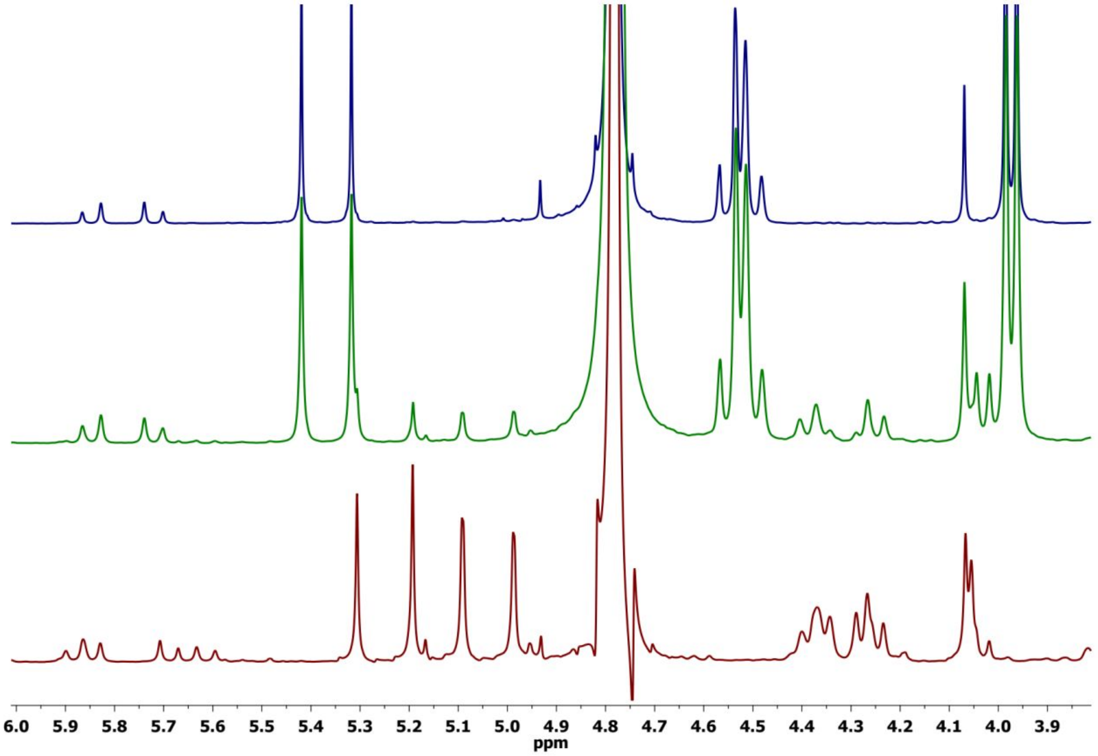

Figure S78. ^{31}P{^{1}H} NMR spectra (162 MHz, D_2O) of the freshly prepared solution of [**1c**]NO_3 and PTA (top, blue line), after 144 at 37 °C (middle, green line) and a solution of [**2c**]CF_3SO_3 (bottom, red line).

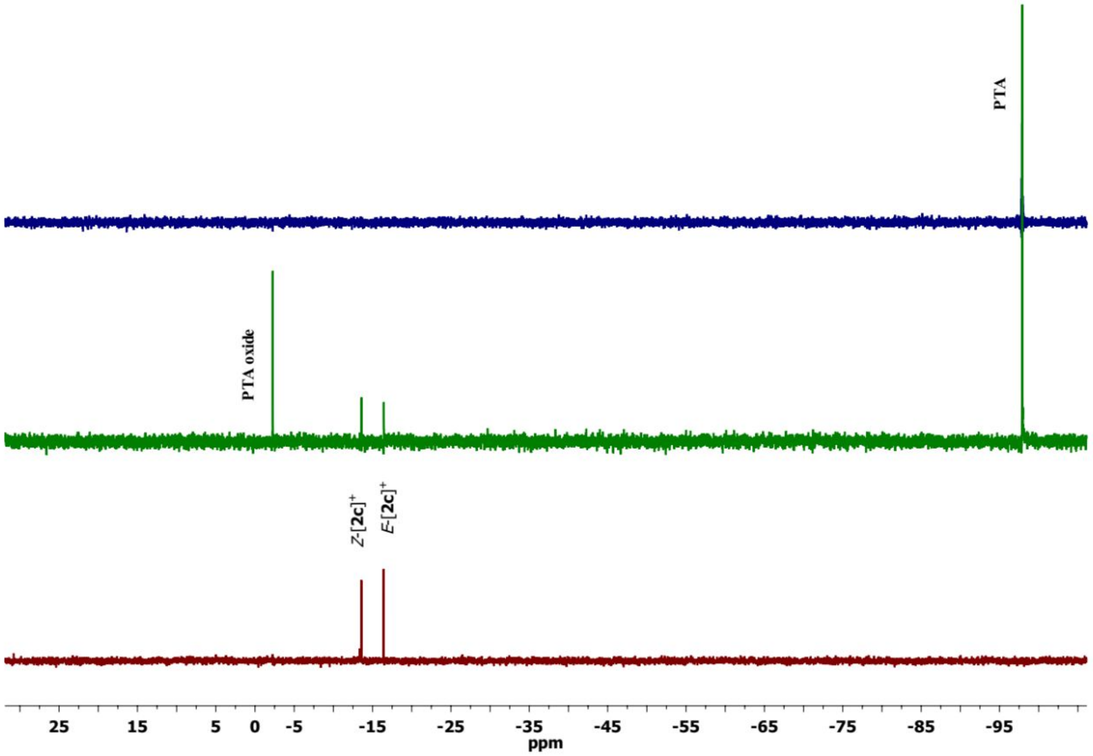

Figure S79. Comparison of ^{1}H NMR spectra (400 MHz, acetone-d_6, 3.6-6.3 ppm) of [**2c**]CF_3SO_3 as a mixture of *cis*/*trans* and *E*/*Z* isomers (top, black line) and the *cis*-*E*/*Z* mixture (bottom, red line).

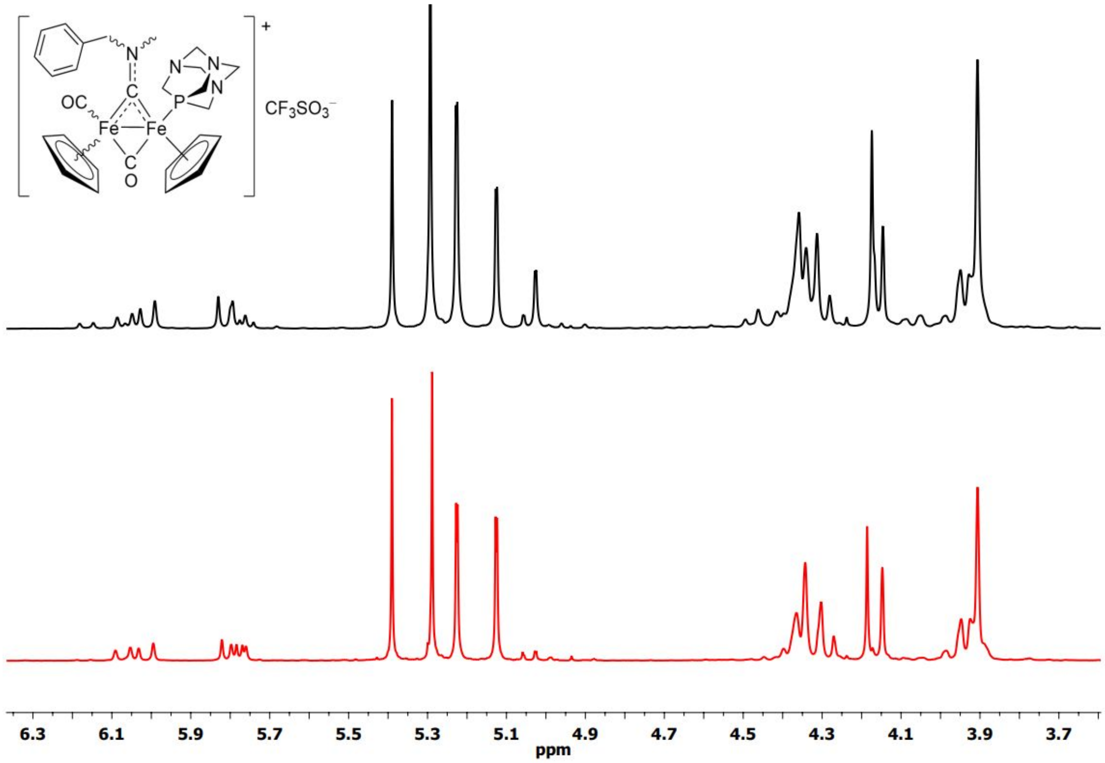

Figure S80. Comparison of ^{31}P NMR spectra (162 MHz, acetone-d_6, – 13 to – 35 ppm) of [**2c**]CF_3SO_3 as a mixture of *cis*/*trans* and *E*/*Z* isomers (top, black line) and the *cis*-*E*/*Z* mixture (bottom, red line).

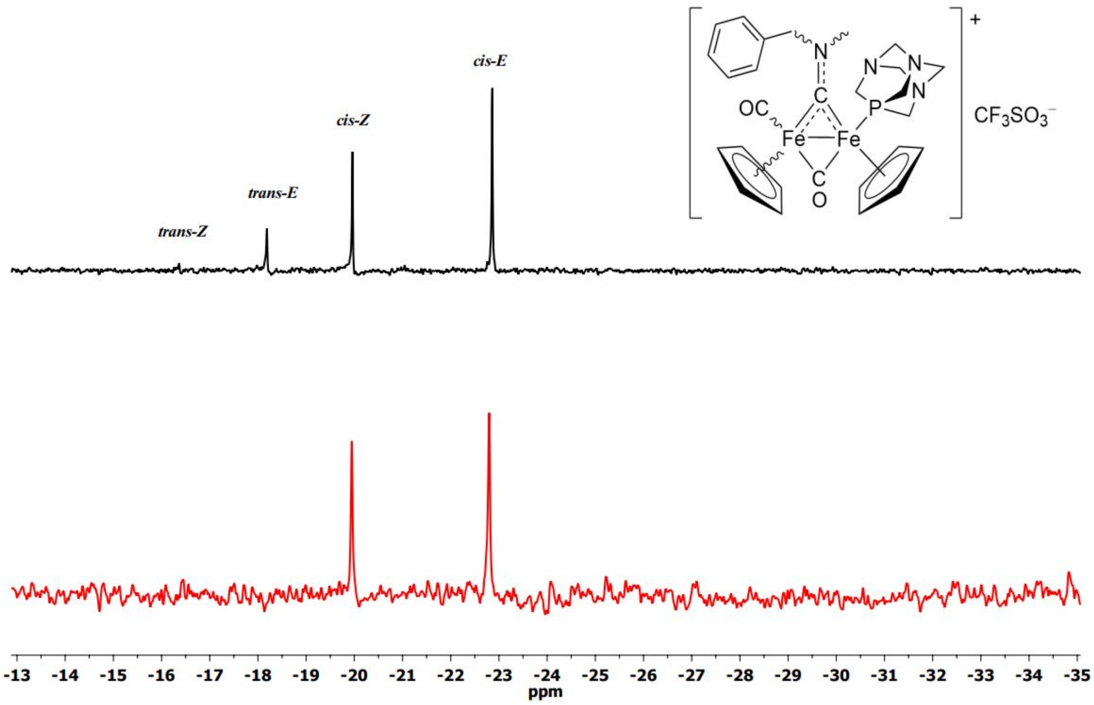

Figure S81. Solid-state IR spectrum (650-4000 cm^{-1}) of $[Fe_2Cp_2(CO)(PTA)(\mu\text{-}CO)\{\mu\text{-}CNMe(Bn)\}]CF_3SO_3$, [**2c**]$CF_3SO_3$ (mixture of *cis*-*E*/*Z* isomers).

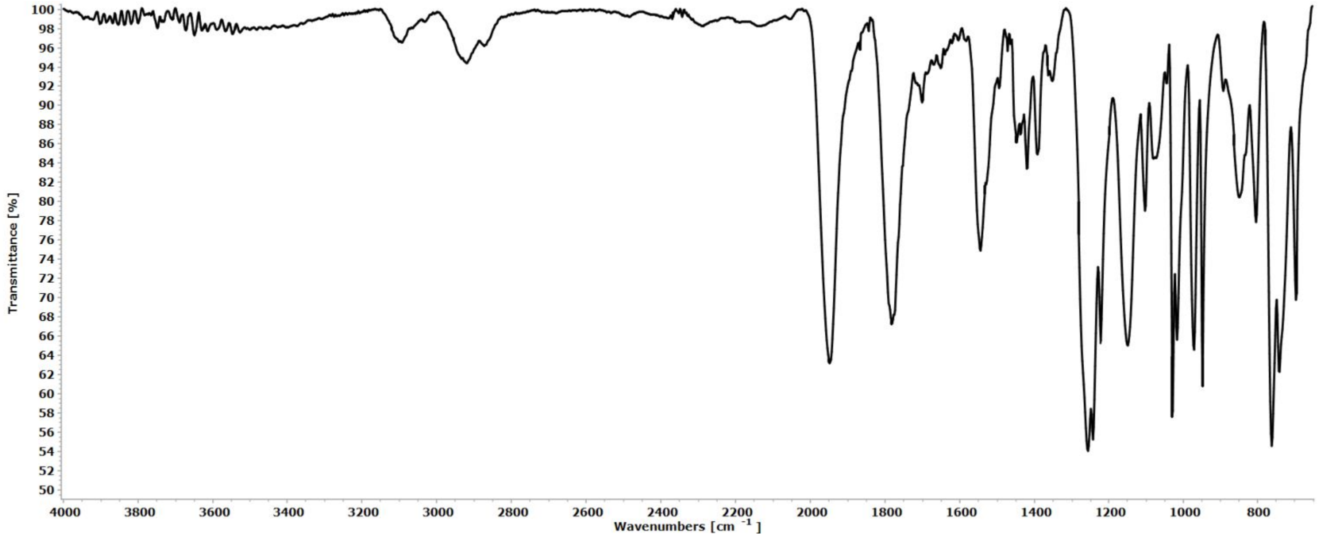

Figure S82. ^{1}H NMR spectrum (401 MHz, CD_3OD) of [**2c**]CF_3SO_3 (*cis*-*E*/*cis*-*Z* isomer ratio 1.3).

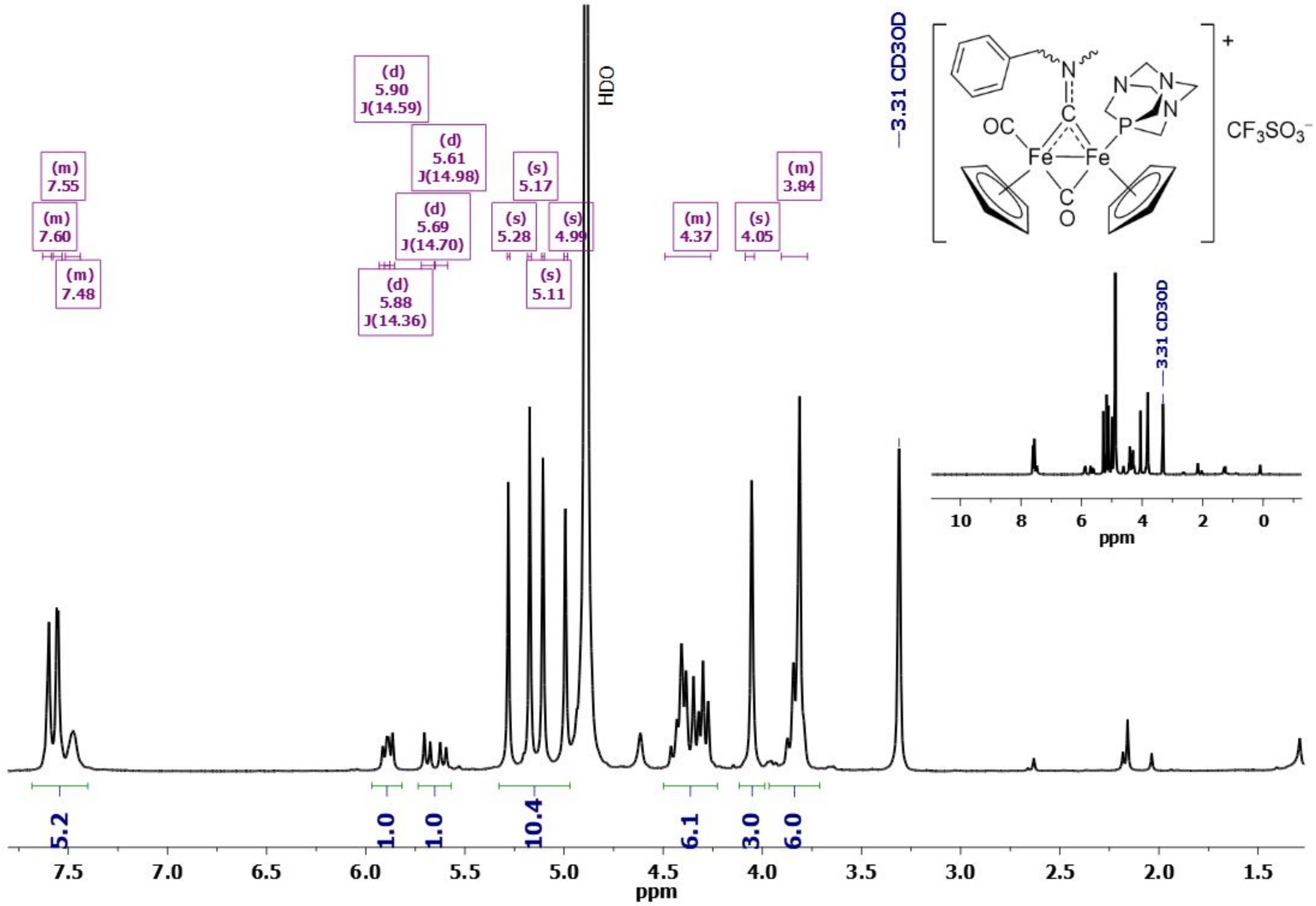

Figure S83. $^{13}C\{^1H\}$ NMR spectrum (101 MHz, CD_3OD) of [**2c**]CF_3SO_3 (*cis-E*/*cis-Z* isomer ratio 1.3).

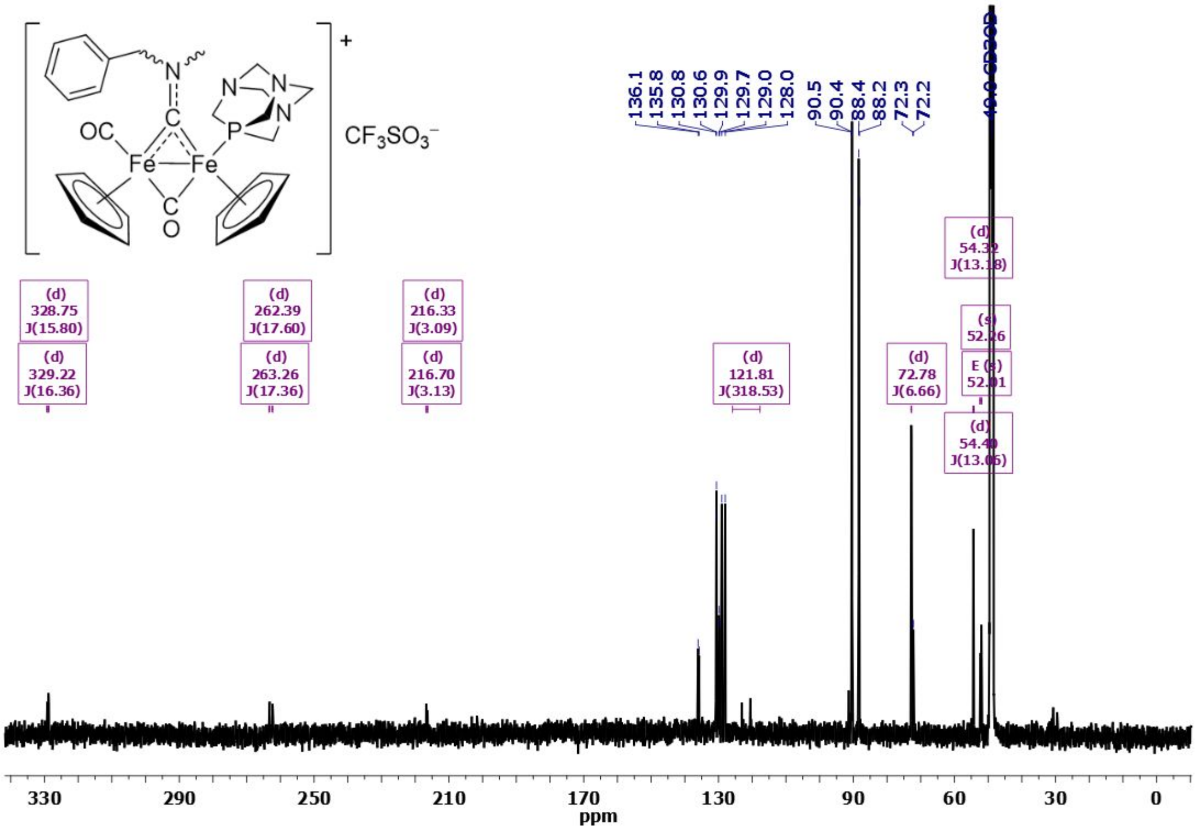

Figure S84. ^{31}P NMR spectrum (162 MHz, CD_3OD) of [**2c**]CF_3SO_3 (*cis-E*/*cis-Z* isomer ratio 1.3).

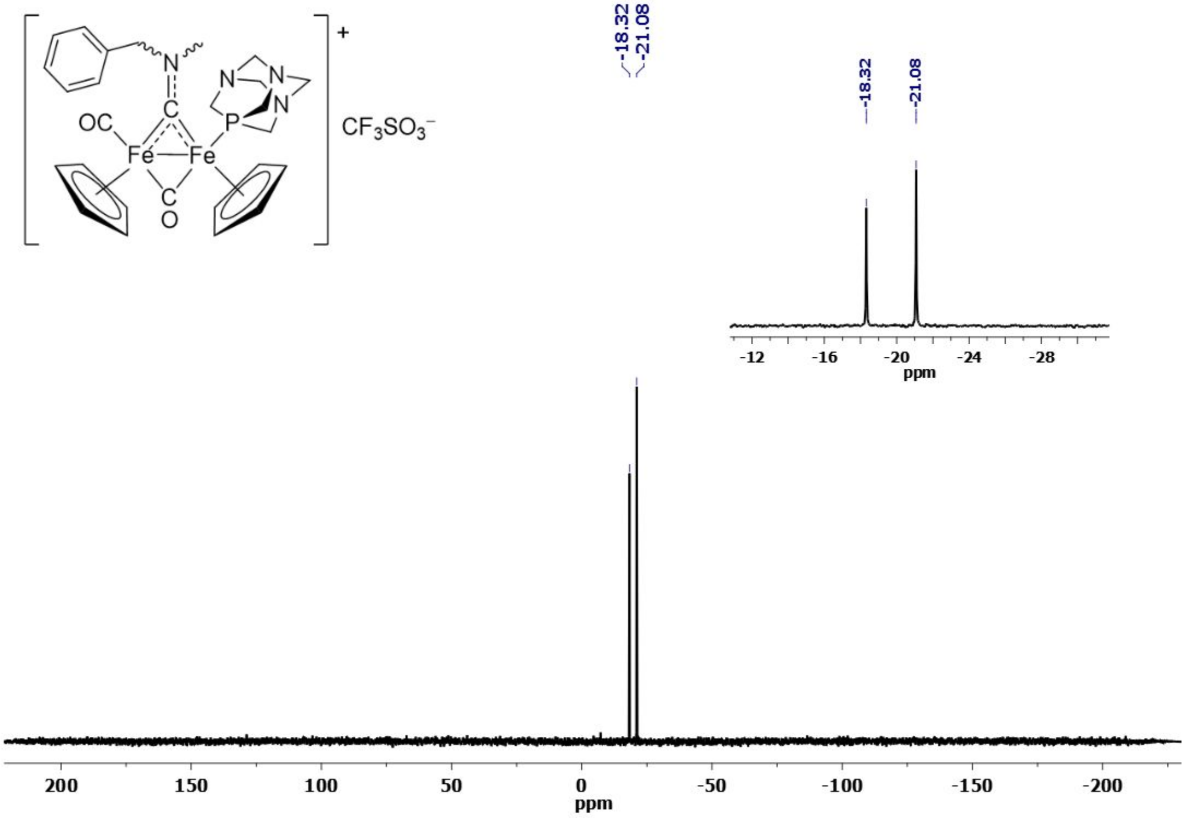

NMR and UV-Vis analyses of aqueous solutions of diiron complexes in the dark

Table S19. ^{1}H NMR or UV-Vis analysis of solutions of [**1a-e**]$^+$ in water or cell culture medium kept for 72 h in the dark at 37 °C.

Entry	Starting material	Solution, technique	Initial concentration [a] c^0_{Fe2} / mol·L^{-1}	compounds detected in the final solution [a] (% amount respect to the starting material)		
				[1]$^+$ [b]	RR'NH_2^+ (% *vs* consumed [**1**]$^+$)	CpH
S18-1	[**1a**]NO_3	D_2O, NMR	$4.16 \cdot 10^{-3}$	94.6	0.3	< LOD
S18-2	[**1a**]NO_3	H_2O, UV-Vis	$9.43 \cdot 10^{-4}$	96.1 ± 0.2	/	/
S18-3	[**1a**]CF_3SO_3	H_2O, UV-Vis	$4.65 \cdot 10^{-4}$	100 ± 0.4	/	/
S18-4	[**1a**]CF_3SO_3	H_2O, UV-Vis	$2.44 \cdot 10^{-4}$	95.2 ± 0.6	/	/
S18-5	[**1a**]NO_3	H_2O, UV-Vis	$2.74 \cdot 10^{-4}$	90.6 ± 0.3	/	/
S18-6	[**1a**]NO_3	H_2O, UV-Vis	$1.38 \cdot 10^{-4}$	99.7 ± 0.9	/	/
S18-7	[**1b**]NO_3	H_2O, UV-Vis	$1.96 \cdot 10^{-4}$	91.6 ± 2.6	/	/
S18-8	[**1b**]NO_3	DMEM-d, NMR	$1.91 \cdot 10^{-3}$	89.6	3.4 (*33*)	1.0
S18-9	[**1c**]NO_3	D_2O, NMR	$4.12 \cdot 10^{-3}$	93.5	trace	< LOD
S18-10	[**1c**]NO_3	D_2O, NMR	$2.35 \cdot 10^{-3}$	96.0	trace	< LOD
S18-11	[**1c**]NO_3	H_2O, UV-Vis	$1.04 \cdot 10^{-3}$	92.6 ± 0.6	/	/
S18-12	[**1c**]CF_3SO_3	H_2O, UV-Vis	$4.07 \cdot 10^{-4}$	88.0 ± 2.9	/	/
S18-13	[**1c**]CF_3SO_3	H_2O, UV-Vis	$1.71 \cdot 10^{-4}$	89.2 ± 2.0	/	/
S18-14	[**1d**]NO_3	H_2O, UV-Vis	$5.25 \cdot 10^{-4}$	93.3 ± 1.8	/	/
S18-15	[**1e**]NO_3	D_2O, NMR	$4.16 \cdot 10^{-3}$	88.2	?	< LOD
S18-16	[**1e**]CF_3SO_3	H_2O, UV-Vis	$1.38 \cdot 10^{-3}$	93.5 ± 2.8	/	/
S18-17	[**1e**]CF_3SO_3	H_2O, UV-Vis	$5.92 \cdot 10^{-4}$	87.7 ± 4.0	/	/
S18-18	[**1e**]NO_3	H_2O, UV-Vis	$4.17 \cdot 10^{-4}$	96.9 ± 3.0	/	/
S18-19	[**1e**]CF_3SO_3	H_2O, UV-Vis	$1.78 \cdot 10^{-4}$	88.9 ± 0.5	/	/
S18-20	[**1e**]NO_3	H_2O, UV-Vis	$1.29 \cdot 10^{-4}$	89.6 ± 2.8	/	/

[a] ^{1}H NMR experiments: the initial concentration and % relative amount of compounds with respect to the freshly prepared solution were calculated using Me_2SO_2 or DSS as internal standard. UV-Vis experiments: the initial concentration was calculated from mass and volume data (volumetric solutions) or from the molar absorbance at 340 nm of the freshly-prepared solution; the % residual amount of starting material was calculated by the relative decrease of the UV-Vis peak at 340 nm (see main text for details). Data expressed with 2 or 1 decimal digits to avoid excessive rounding. [b] For [**1a,c**]CF_3SO_3: including *cis* and *trans* isomers.

Computational studies

Table S20. DFT-optimized free Gibbs energies of the species involved in the decomposition mechanism (see Figure 11 in the main text for the structures and associated transformations).

Complex	ΔG / kcal·mol^{-1}
**MeMe_s**	− 29.1
**MeMe_t**	0
**TS1_s**	19.8
**TS1_t**	21.0
**TS1alt_t**	53.9
**A_t**	6.1
**A_s**	− 2.5
**TS2_t**	18.3
**B_t**	1
**TS3_t**	20.6
**C_t**	− 41.3
**TS4_t**	− 18.7
**D_t**	− 44

Table S21. Comparison between solid state and DFT-optimized geometries of [**1a**]$^+$. Bonds are in Å, angles in degrees.

Parameter	Experimental	Computed
Fe1-Fe2	2.5146(5)	2.494
Fe1-C1	1.772(3)	1.739
Fe2-C2	1.763(3)	1.739
Fe1-C3	1.937(3)	1.913
Fe2-C3	1.933(3)	1.921
Fe1-C4	1.880(3)	1.863
Fe2-C4	1.873(3)	1.858
C1-O1	1.144(4)	1.161
C2-O2	1.142(4)	1.162
C3-O3	1.175(4)	1.183
N1-C4	1.294(4)	1.304
N1-C5	1.468(4)	1.475
N1-C6	1.472(4)	1.476
Fe1-C1-O1	179.0(3)	177.8
Fe2-C2-O2	179.7(3)	178.0
Fe1-C3-Fe2	81.05(11)	81.1
Fe1-C4-Fe2	84.15(11)	84.1
C4-N1-C5	123.4(2)	123.1
C4-N1-C6	123.2(3)	123.1
C5-N1-C6	113.3(2)	113.8

Chlorido dicarbonyl complexes: IR and NMR characterization and silver-mediated decomposition in aqueous solution

Figure S85. Solid-state IR spectrum (650-4000 cm^{-1}) of [$Fe_2Cp_2Cl(CO)(\mu\text{-}CO)\{\mu\text{-}CNMe(Bn)\}$], **3c** (*E*/Z isomer mixture).

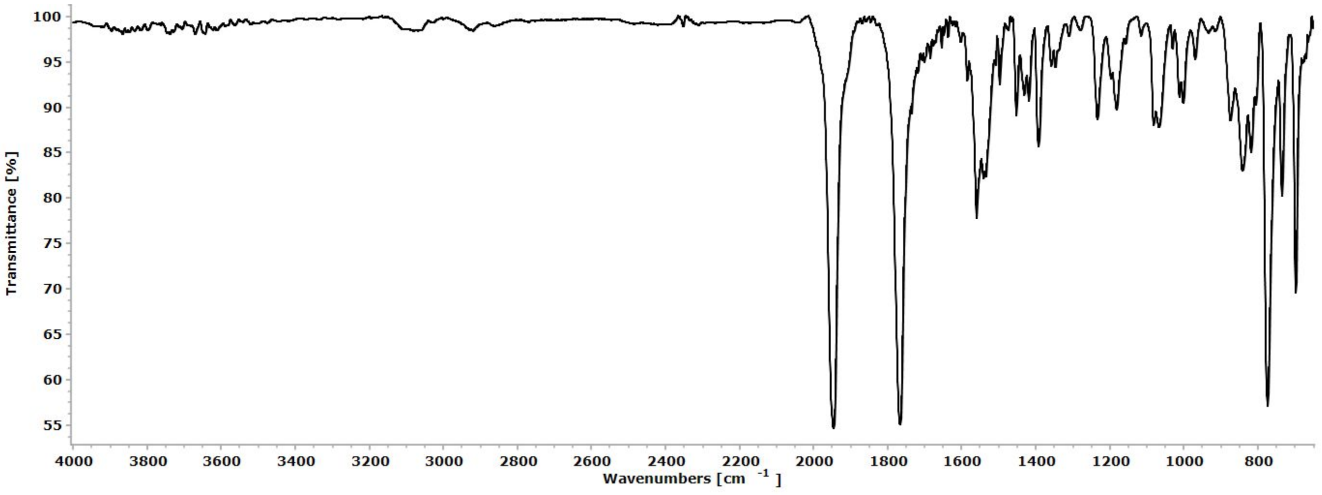

Figure S86. ^1H NMR spectrum (401 MHz, acetone-d$_6$) of **3c** (*E*/*Z* ratio 1.0).

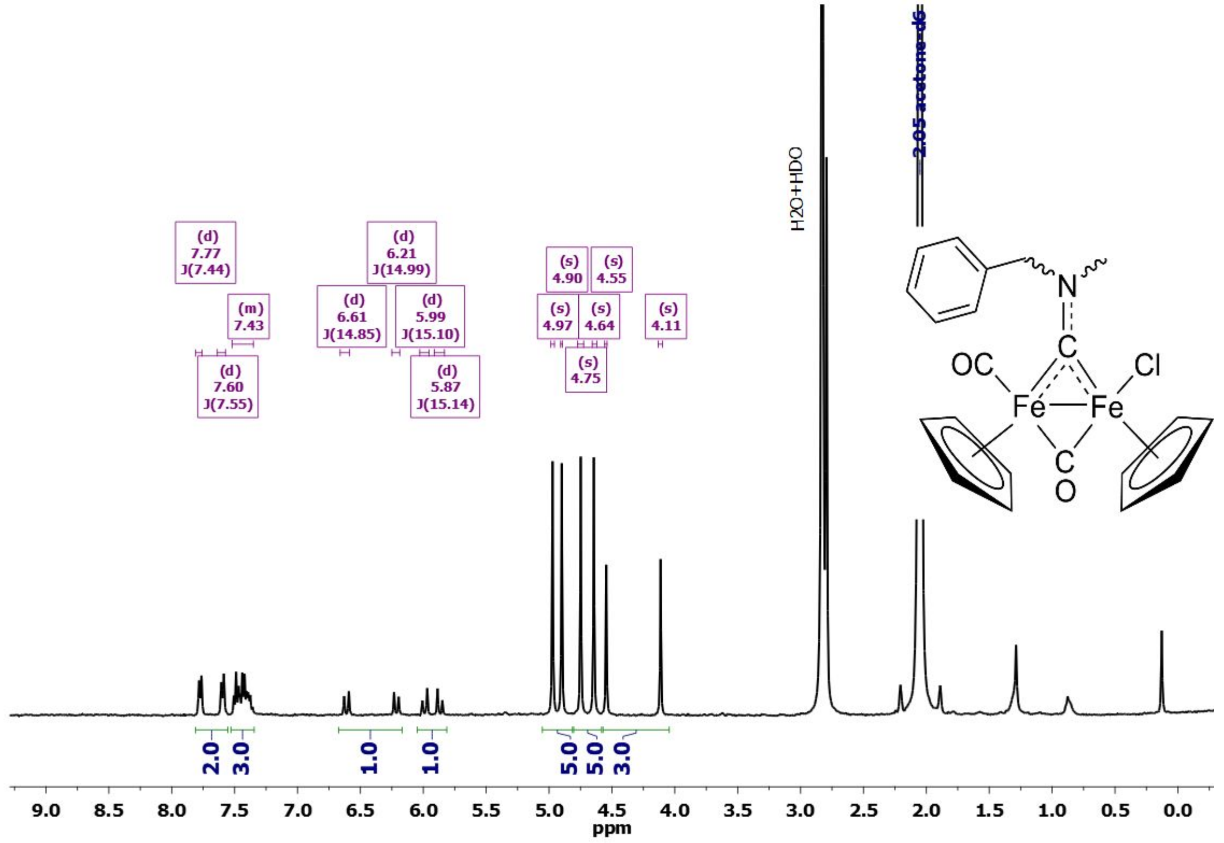

Figure S87. Solid-state IR spectrum (650-4000 cm^{-1}) of [$Fe_2Cp_2Cl(CO)(\mu\text{-}CO)\{\mu\text{-}CNBn_2\}$], **3d** (*cis*/*trans* isomer mixture).

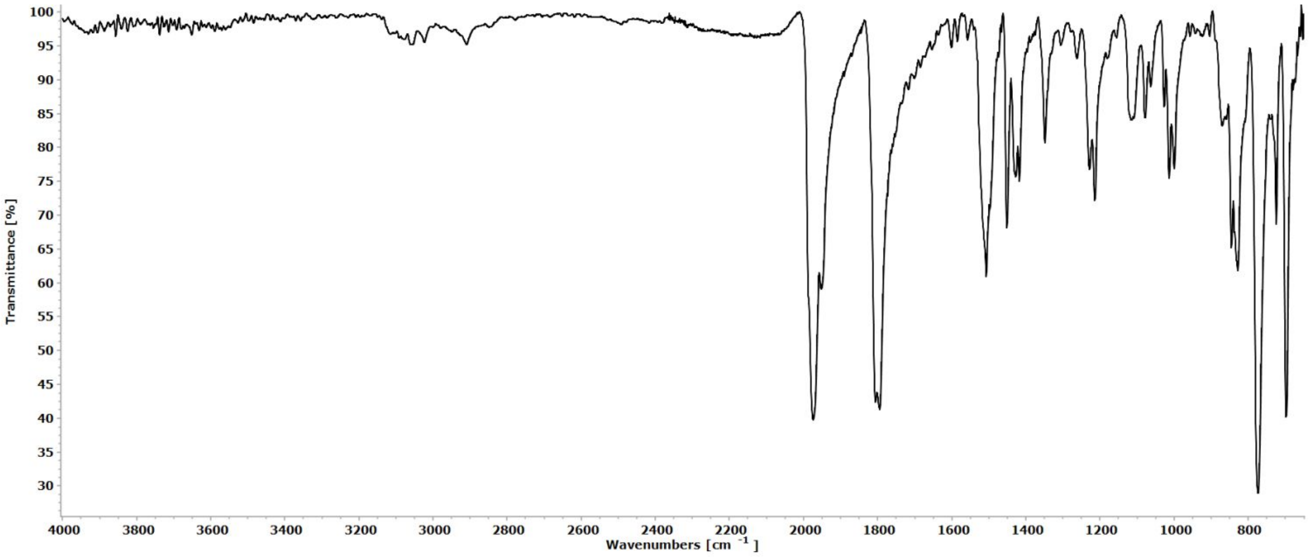

Figure S88. ^1H NMR spectrum (401 MHz, $CDCl_3$) of **3d** (pure *cis* isomer).

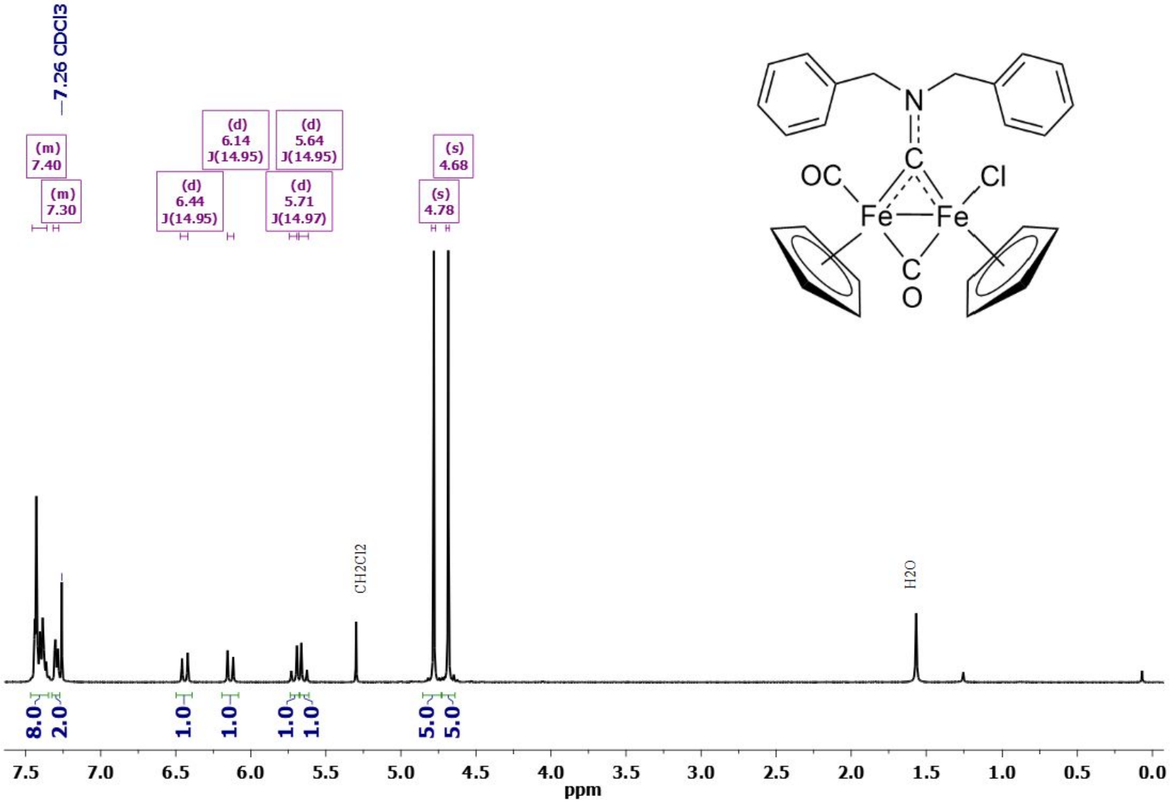

Figure S89. ^{1}H NMR spectrum (401 MHz, $CDCl_3$) of **3d** (*cis*/*trans* ratio 5.8). Only resonances due the *trans* isomer are highlighted while integrals refer collectively to all isomers.

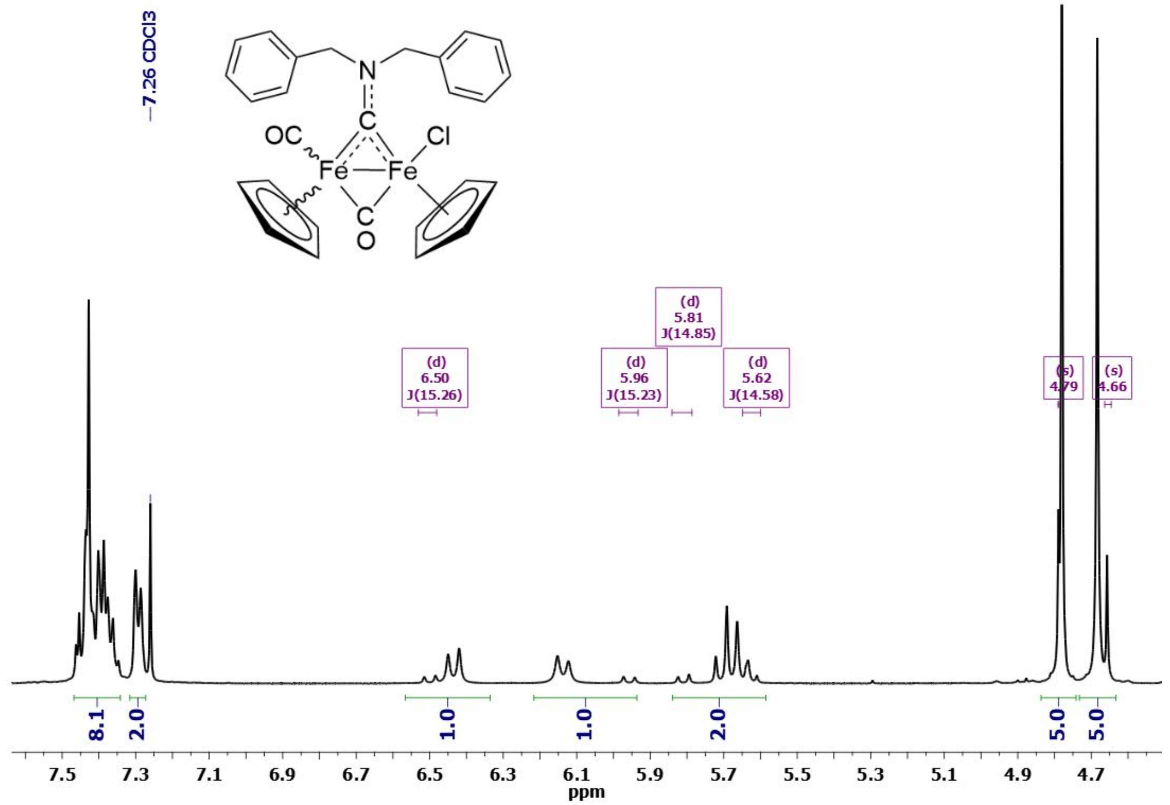

Figure S90. $^{13}C\{^{1}H\}$ NMR spectrum (101 MHz, $CDCl_3$) of **3d** (*cis*/*trans* ratio 5.8).

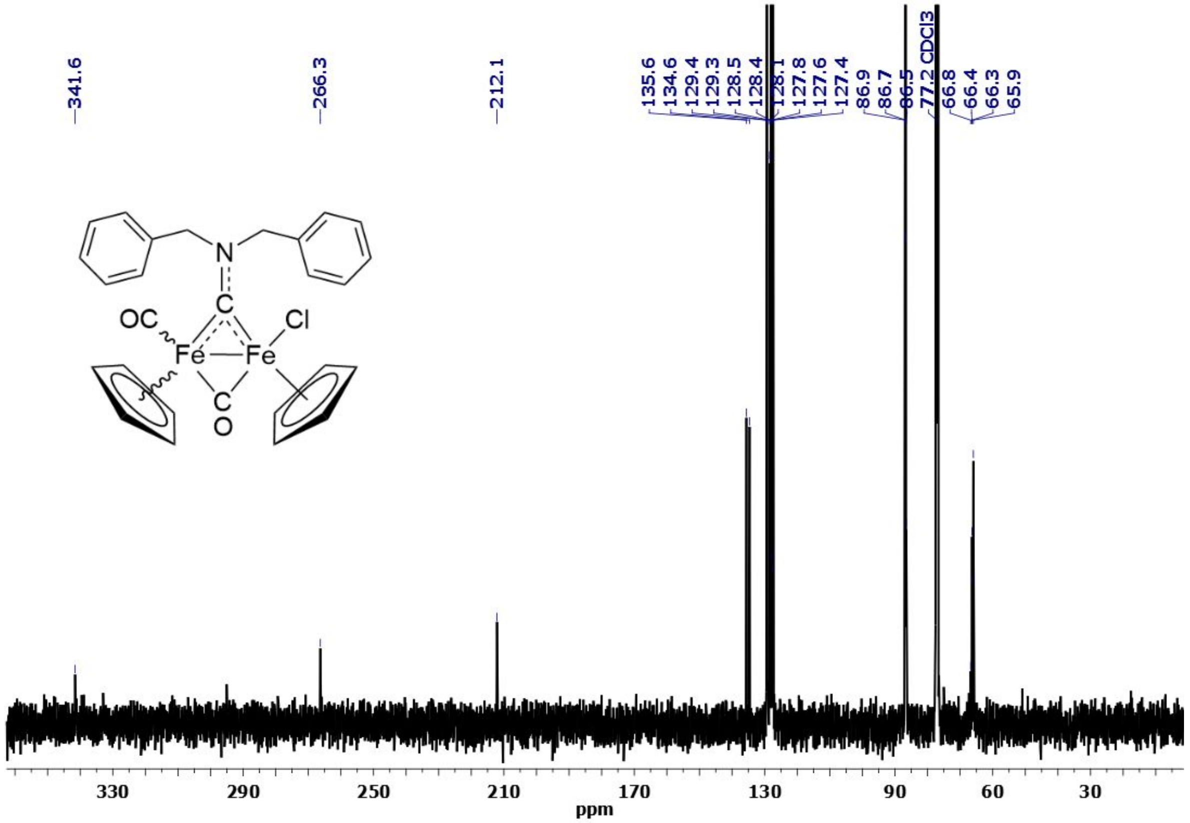

Figure S91. Solid-state IR spectrum (650-4000 cm^{-1}) of [$Fe_2Cp_2Cl(CO)(\mu\text{-}CO)\{\mu\text{-}CNMe(Xyl)\}$], **3e** (*E* isomer).

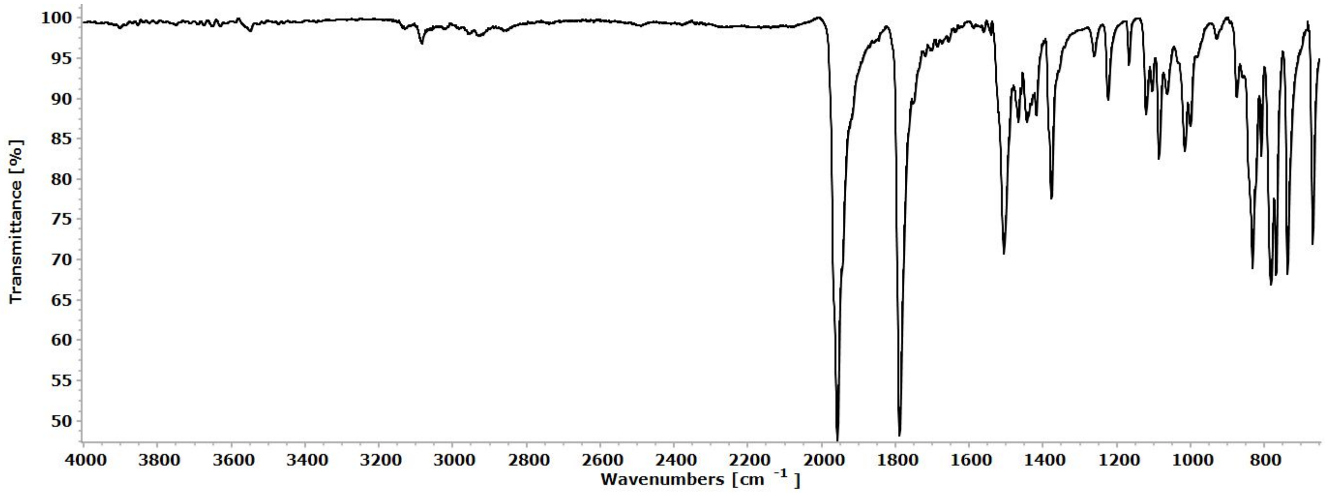

Figure S92. ^{1}H NMR spectrum (401 MHz, $CDCl_3$) of **3e** (*E* isomer).

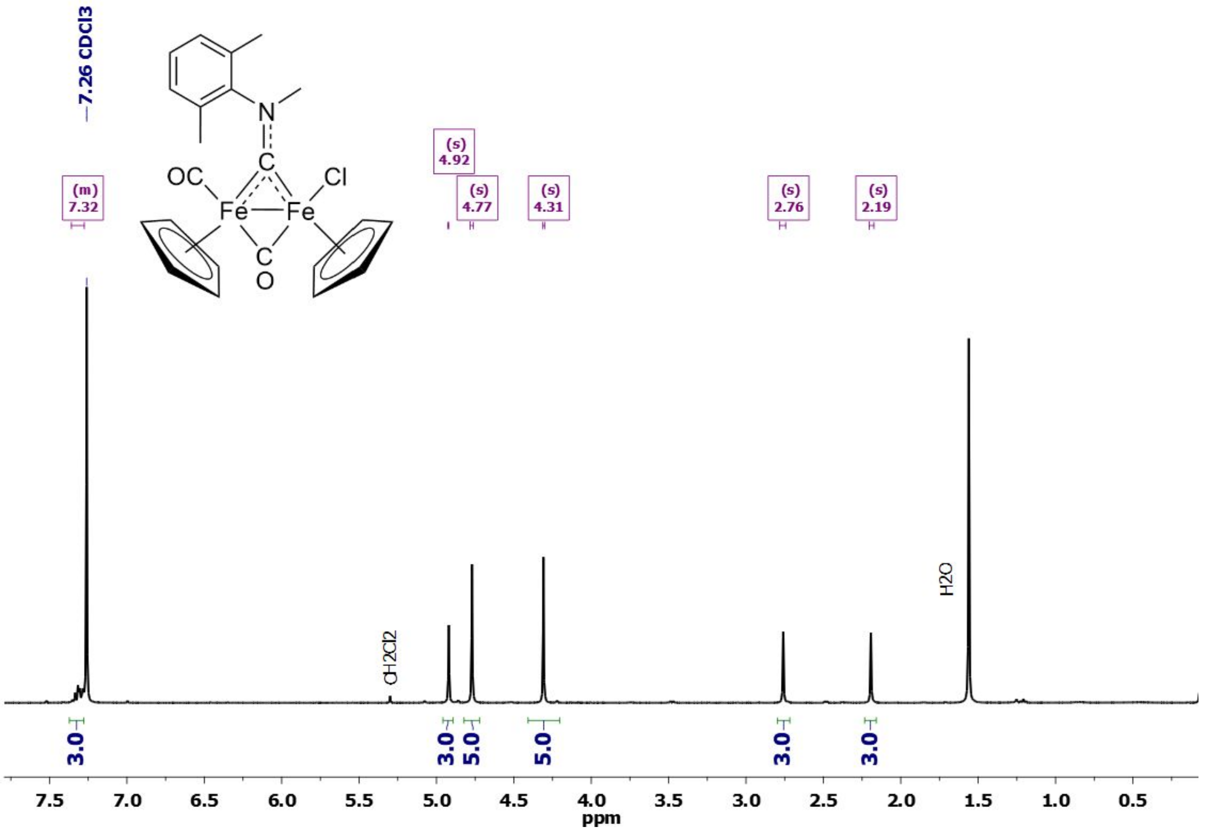

Figure S93. ^{1}H NMR spectrum (401 MHz, $CDCl_3$) of **3e** (*E/Z* isomer ratio 7.7). Only resonances of the minor (*Z*) isomer are highlighted and integrated.

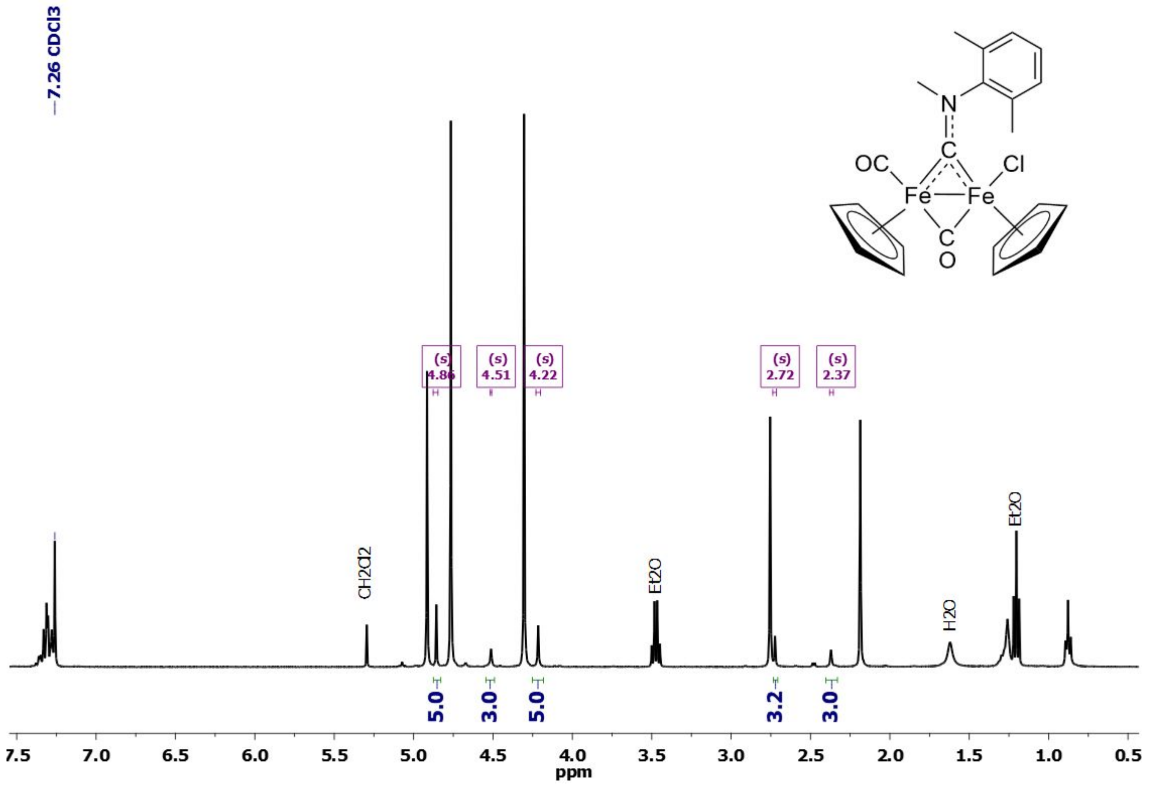

Figure S94. Solid-state IR spectrum (650-4000 cm^{-1}) of [$Fe_2Cp_2Cl(CO)(\mu\text{-}CO)\{\mu\text{-}CNMe(4\text{-}C_6H_4OMe)\}$], **3f** (*E*/*Z* isomer mixture).

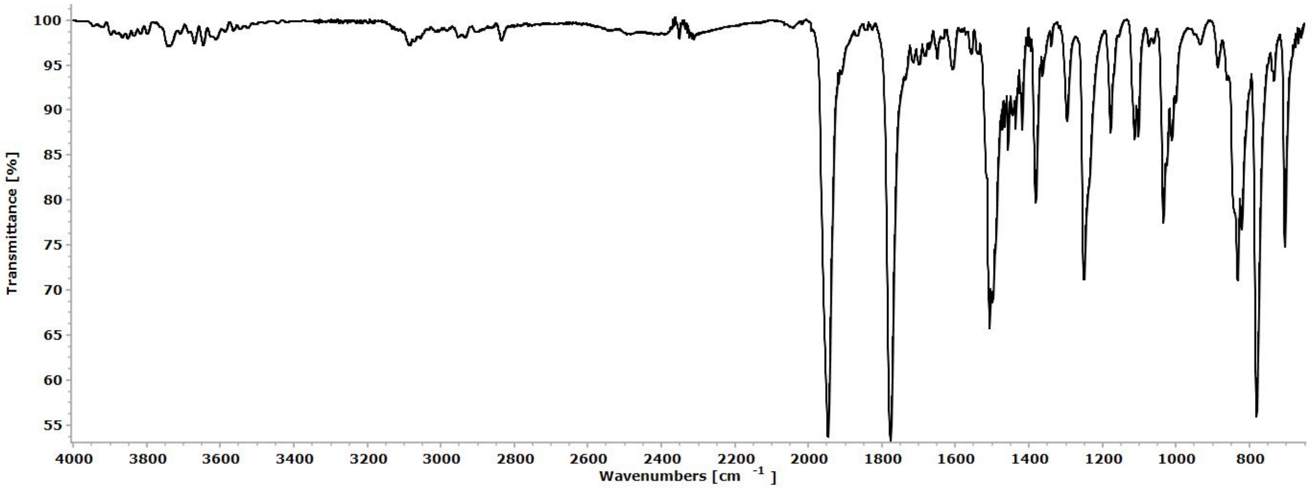

Figure S95. ^{1}H NMR spectrum (401 MHz, $CDCl_3$) of **3f** (*E*/*Z* ratio 2.0).

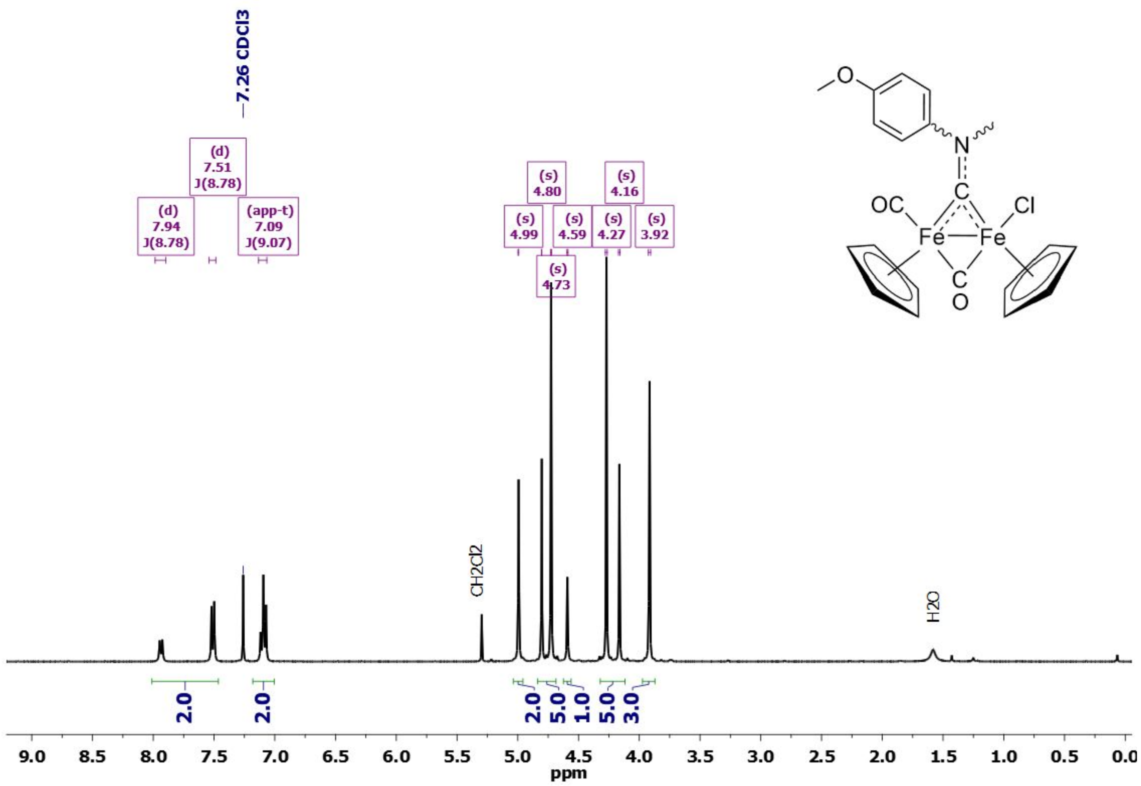

Figure S96. Solvent-subtracted IR spectra (1500-2100 cm^{-1}) of **3c** (red line), **3d** (blue line), **3e** (cyan line), **3f** (yellow line) in CH_2Cl_2.

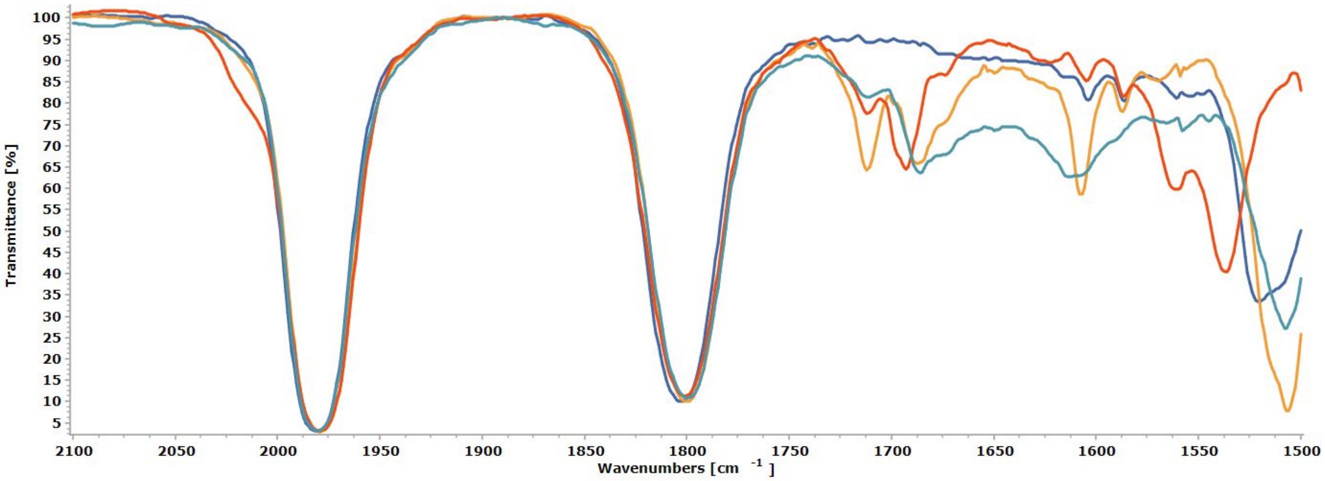

Table S22. ^{1}H NMR analyses of the water-soluble products of the reaction of **3c-f** with $Ag(CF_3SO_3)$.

Starting material	compounds detected in solution	% amount respect to the starting material [a]	Notes
**3a**	CpH		$H_2O/CDCl_3$ reaction
**3c**	$Bn(Me)NH_2^+$	33	
**3d**	$Bn_2NH_2^+$	20	
**3e**	$Xyl(Me)NH_2^+$	28	Confirmed by GC-MS (Figure S92)
**3f**	$Anis(Me)NH_2^+$	18	
**3f**	$Anis(Me)NH_2^+$	27	Treated with HCl before drying under vacuum

Abbreviation list: Anis = 4-$C_6H_4(OMe)$; Cy = cyclohexyl, Cp = cyclopentadienyl, Xyl = 2,6-$C_6H_3Me_2$

Figure S97. Top: experimental MS spectrum of a GC peak from the analysis of the aqueous solution resulting from the reaction of **3e** and $Ag(CF_3SO_3)$. Bottom: calculated MS patten for Xyl(Me)NH.

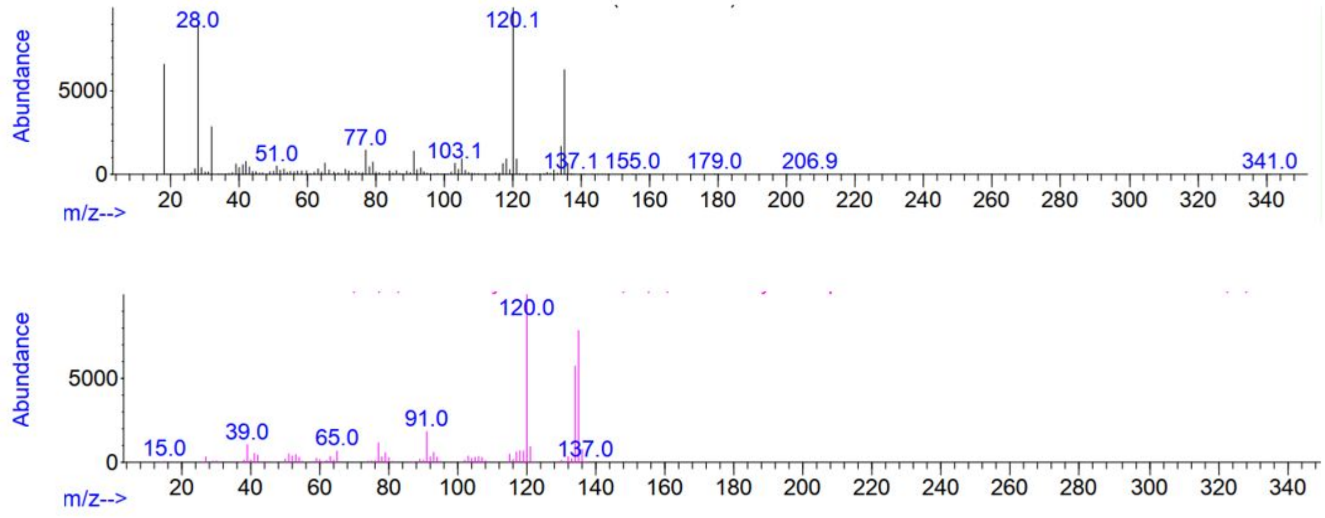

X-ray crystallography

Table S23. Crystal data and measurement details for [**1a**]NO_3, [**1b**]NO_3·0.5CH_2Cl_2, [**1e**]NO_3, *E*-[**2c**]CF_3SO_3 and **3d**.

	[**1a**]NO_3	[**1b**]NO_3·0.5CH_2Cl_2	[**1e**]NO_3	*E*-[**2c**]CF_3SO_3	**3d**
Formula	$C_{16}H_{16}Fe_2N_2O_6$	$C_{21.5}H_{25}ClFe_2N_2O_6$	$C_{23}H_{22}Fe_2N_2O_6$	$C_{28}H_{32}F_3Fe_2N_4O_5PS$	$C_{27}H_{24}ClFe_2NO_2$
FW	444.01	554.58	534.12	736.30	541.62
T, K	100(2)	100(2)	100(2)	100(2)	100(2)
λ, Å	0.71073	0.71073	0.71073	0.71073	0.71073
Crystal system	Monoclinic	Monoclinic	Monoclinic	Orthorhombic	Tetragonal
Space group	*Cc*	$P2_1/n$	*Cc*	$Pna2_1$	$P\bar{4}2_1c$
a, Å	13.6918(3)	14.3956(4)	13.5902(7)	22.1097(6)	18.5720(10)
b, Å	8.4441(2)	10.4987(3)	11.8610(6)	13.4405(4)	18.5720(10)
c, Å	15.1167(3)	15.6209(5)	15.0859(8)	9.6834(3)	13.8157(7)
β,°	107.3600(10)	103.9110(10)	114.943(2)	90	90
Cell Volume, $Å^3$	1668.11(6)	2291.62(12)	2204.9(2)	2877.57(15)	4765.3(6)
Z	4	4	4	4	8
D_c, g·cm^{-3}	1.768	1.607	1.609	1.700	1.510
μ, mm^{-1}	1.776	1.423	1.359	1.204	1.354
F(000)	904	1140	1096	1512	2224
Crystal size, mm	0.16×0.12×0.11	0.18×0.15×0.12	0.15×0.14×0.10	0.14×0.08×0.01	0.14×0.11×0.08
θ limits,°	2.824–29.999	1.728–25.096	2.383–25.098	1.773–26.996	1.551–26.991
Reflections collected	12433	24498	9759	44822	70224
Independent reflections	4857 [R_{int} = 0.0407]	4086 [R_{int} = 0.0481]	3910 [R_{int} = 0.0438]	6278 [R_{int} = 0.0593]	5205 [R_{int} = 0.0636]
Data / restraints /parameters	4857 / 2 / 238	4086 / 27 / 308	3910 / 104 / 302	6278 / 1 / 399	5205 / 0 / 299
Goodness on fit on F^2	1.035	1.088	1.139	1.100	1.146
R_1 ($I > 2\sigma(I)$)	0.0266	0.0697	0.0468	0.0348	0.0358
wR_2 (all data)	0.0646	0.1629	0.1003	0.0791	0.0896
Largest diff. peak and hole, e $Å^{-3}$	0.581 / –0.347	1.763 / –0.907	0.534 / –0.590	0.498 / –0.421	0.639 / –0.405

References

1 G. Agonigi, L. Biancalana, M. G. Lupo, M. Montopoli, N. Ferri, S. Zacchini, F. Binacchi, T. Biver, B. Campanella, G. Pampaloni, V. Zanotti, F. Marchetti, Exploring the Anticancer Potential of Diiron Bis-cyclopentadienyl Complexes with Bridging Hydrocarbyl Ligands: Behavior in Aqueous Media and In Vitro Cytotoxicity, *Organometallics* 2020, 39, 645-657

2 L. Biancalana, M. De Franco, G. Ciancaleoni, S. Zacchini, G. Pampaloni, V. Gandin, F. Marchetti, Easily Available, Amphiphilic Diiron Cyclopentadienyl Complexes Exhibit in Vitro Anticancer Activity in 2D and 3D Human Cancer Cells through Redox Modulation Triggered by CO Release, *Chem. Eur. J.* 2021, 27, 10169-10185

3 L. Biancalana, M. Kubeil, S. Schoch, S. Zacchini, F. Marchetti, Switching on Cytotoxicity of Water-Soluble Diiron Organometallics by UV Irradiation, *Inorg. Chem*. 2022, 61, 7897–7909

4 M. De Franco, L. Biancalana, C. Zappelli, S. Zacchini, V. Gandin, F. Marchetti, 1,3,5-Triaza-7-phosphaadamantane and Cyclohexyl Groups Impart to Di-Iron(I) Complex Aqueous Solubility and Stability, and Prominent Anticancer Activity in Cellular and Animal Models, *J. Med. Chem*. 2024, 67, 11138–11151

5 L. Biancalana, M. De Franco, v. Gandin, F. Marchetti, 2D and 3D anticancer activity of diiron biscyclopentadienyl complexes incorporating flurbiprofen and chlorambucil, *RSC Med. Chem*., 2025, 16, 4463

6 A. Rossi, L. Biancalana, J. Vanco, T. Malina, S. Zacchini, Z. Dvorak, Z. Travnícek, F. Marchetti, The effect of a varying pyridine ligand on the anticancer activity of Diiron(I) bis-cyclopentadienyl complexes, *Chem.-Biol. Interact*. 2025, 406, 111318

7 S. Stocchetti, J. Vanco, G. Bresciani, L. Biancalana, J. Belza, S. Zacchini, Z. Dvorak, S. Benetti, T. Biver, M. Bortoluzzi, Z. Travnícek, F. Marchetti, Anticancer diiron aminocarbyne complexes with labile N-donor ligands, *Eur. J. Med. Chem*. 2025, 286, 117304

8 C. Saviozzi, L. Biancalana, S. Zacchini, M. De Franco, V. Gandin, F. Marchetti, Isocyanide Incorporation Expands the Anticancer Potential of Diiron(I) Aminocarbyne Complexes, *Chem. Eur. J.* 2026, e03129

9 G. Garrido, V. de Nogales, C. Ràfols, E. Bosch, Acidity of several polyprotic acids, amiodarone and quetiapine hemifumarate in pure methanol, *Talanta* 2007, 73, 115-120

10 A. G. Grechin, H.-J. Buschmann, E. Schollmeyer, Complexation of gaseous guests by solid host: I. Quantitative thermodynamic approach for the reactions of β-cyclodextrin with amines using data in aqueous solution, *Thermochim. Acta* 2006, 449, 67-72

11 P. Wiczling, M. J. Markuszewski, R. Kaliszan, Determination of pKa by pH Gradient Reversed-Phase HPLC, *Anal. Chem*. 2004, 76, 3069–3077

12 F. Barbato, G. di Martino, L. Grumetto, M.I. La Rotonda, Prediction of drug-membrane interactions by IAM–HPLC: effects of different phospholipid stationary phases on the partition of bases, *Eur. J. Pharm. Sci.* 2004, 22, 261-269

13 S. L. Shapiro, E. S. Isaacs, V. Bandurco, L. Freedman, Apparent Dissociation Constants of Haloaralkylamines, *J. Med. Chem*. 1962, 5, 4, 793–799

14 J. J. Christensen, R. M. Izatt, D. P. Wrathall, L. D. Hansen, Thermodynamics of proton ionization in dilute aqueous solution. Part XI. pK, ΔH°, and ΔS° values for proton ionization from protonated amines at 25°, *J. Chem. Soc. A*, 1969, 1212-1223

15 H. Demirelli, On the Role of the Solvent and Substituent on the Protonation Equilibria of Di-Substituted Anilines in Dioxane–Water Mixed Solvents, *J. Solut. Chem*. 2005, 34, 1283–1295.

16 G. Girault-Vexlearschi, No 104. - Influence de la ramification des chaînes hydrocarbonées sur la basicité des amines. III. Étude de l'équilibre d'ionisation des amines, *Bull. Soc. Chim. Fr*. 1956, 23, 589-596

17 G. Garrido, M. Rosés, C. Ràfols, E. Bosch, Acidity of Several Anilinium Derivatives in Pure Tetrahydrofuran, *J. Solut. Chem*. 2008, 37, 689–700

18 J. Oszczapowicz, W. Krawczyk and P. Łyźwiński, Amidines. Part 30. Influence of substitution at amino nitrogen atom on pKa values of N2-phenylacetamidines and N2-phenylformamidines, *J. Chem. Soc., Perkin Trans*. 1990, 2, 311-314

19 J. Jiao, F. Xiao, C. Wang, Z. Zhang, Iodine-Promoted Metal-Free Cyclization and O/S Exchange of Acrylamides with Thiuram: One-Step Synthesis of Quinolino-2-thiones, *J. Org. Chem*. 2022, 87, 4965–4970

20 A. Streitwieser, L. L. Nebenzahl, Carbon Acidity. LII. Equilibrium Acidity of Cyclopentadiene in Water and in Cyclohexylamine, *J. Am. Chem. Soc*. 1976, 98, 2188-2190

21 F. Trentin, A. Scarso, G. Strukul, Micellar-driven substrate selectivity in Cr(salen)Cl catalytic Diels–Alder reaction in water, *Tetr. Lett*. 2011, 52, 6978-6981

22 (a) P. S. Pinto, G. D. Lanza, J. D. Ardissonb, R. M. Lago, Controlled Dehydration of $Fe(OH)_3$ to Fe_2O_3: Developing Mesopores with Complexing Iron Species for the Adsorption of β-Lactam Antibiotics, *J. Braz. Chem. Soc*., 2019, 30, 310-317. (b) A. G. Belous, E. V. Pashkova, V. A. Elshanskii, and V. P. Ivanitskii, Effect of Precipitation Conditions on the Phase Composition, Particle Morphology, and Properties of Iron(III,II) Hydroxide Precipitates, *Inorg. Mater*. 2000, 36, 343-351. (c) L. Suber, S. Foglia, D. Fiorani, H. Romero, A. Montone, A. Roig, L. Casasc, Synthesis,

morphological–structural characterization and magnetic properties of amorphous iron (III)-oxyhydroxy-phosphate nanoparticles, *J. Solid State Chem*. 2004, 177, 2440–2448. (d) M. Ristić, S. Krehula, M. Reissner, S. Musić, 57Fe Mössbauer, XRD, FT-IR, FE SEM Analyses of Natural Goethite, Hematite and Siderite, *Croat. Chem. Acta* 2017, 90, 499–507. (e) C. Rémazeilles, Ph. Refait, Fe(II) hydroxycarbonate $Fe_2(OH)_2CO_3$ (chukanovite) as iron corrosion product: Synthesis and study by Fourier Transform Infrared Spectroscopy, *Polyhedron* 2009, 28, 749–756

23 R. M. Cornell, U. Schwertmann, Characterization. The Iron Oxides: Structure, Properties, Reactions, Occurences and Uses, Second Edition, 2003. https://doi.org/10.1002/3527602097.ch7

24 DMEM formulation: https://www.sigmaaldrich.com/IT/it/technical-documents/technical-article/cell-culture-and-cell-culture-analysis/mammalian-cell-culture/dulbecco-modified-eagle-medium-formulation
